# Supplementary figures and images for: Reconstructed influenza A/H3N2 infection histories reveal variation in incidence and antibody dynamics over the life course
Source: PLoS Biol. 2024 Nov 7;22(11):e3002864. doi: 10.1371/journal.pbio.3002864 (PMC11542844; doi:10.1371/journal.pbio.3002864)

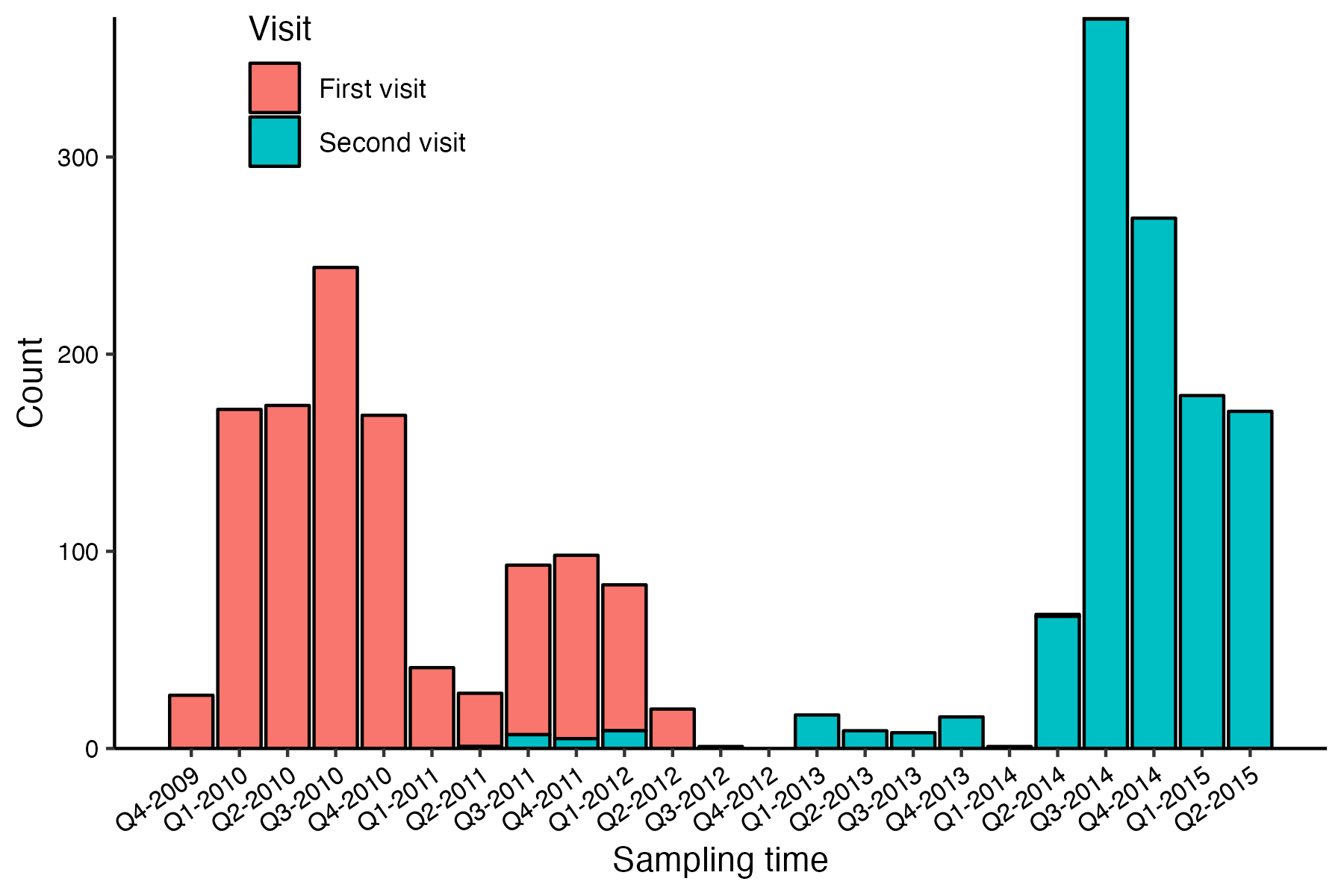

Supplement: S1 Fig — First and second visits refer to an individual’s serum sample order, which may differ from the sample collection round of the overall study. The data underlying this figure can be found at https://doi.org/10.5281/zenodo.12795911. (TIF) [file pbio.3002864.s001.tif]

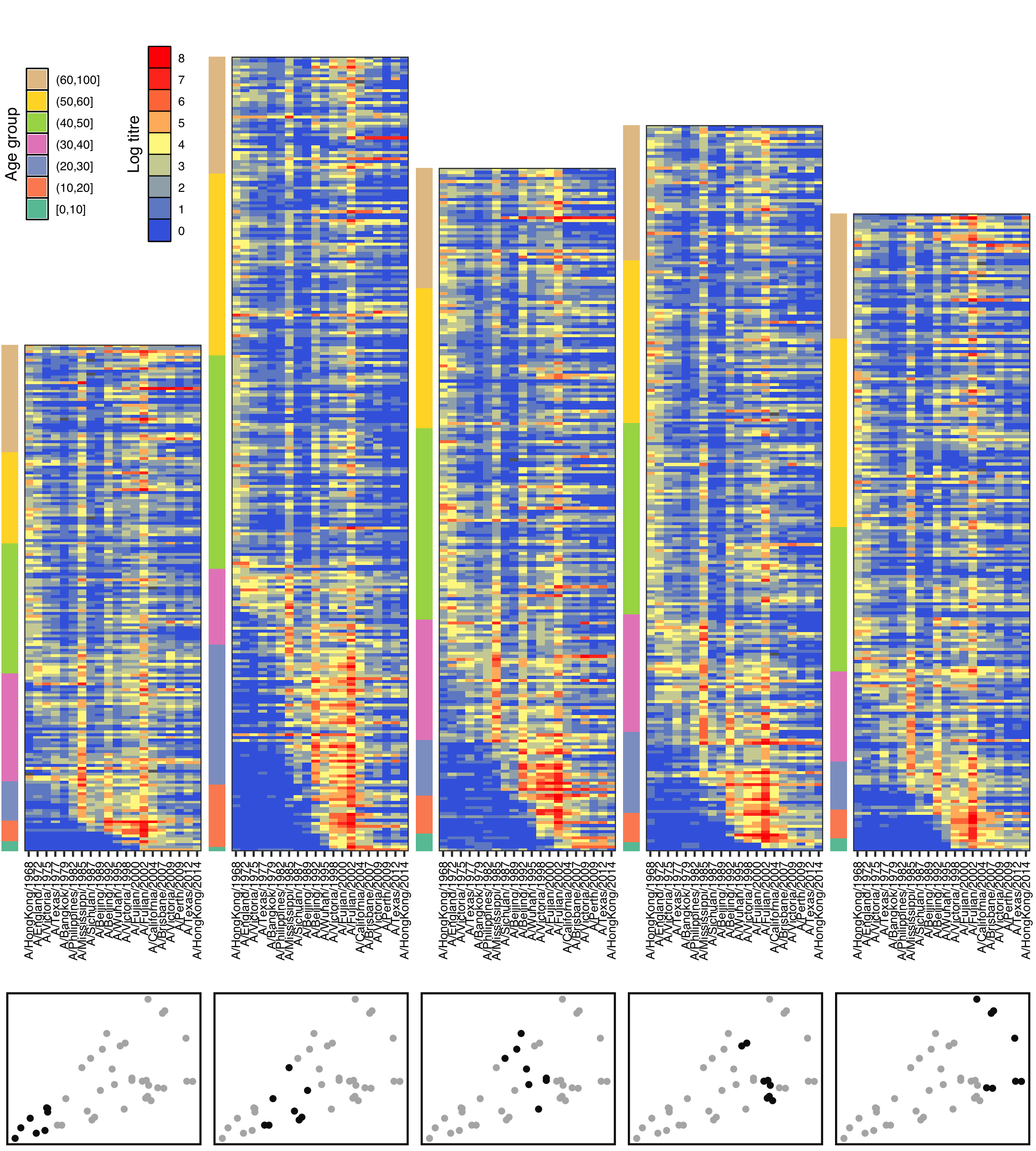

Supplement: S2 Fig — Each cell represents the log HI titre for 1 individual measured against 1 strain, shown on the x-axis. Locations were grouped into quintiles based on increasing distance from Guangzhou city center (bottom panels). Individuals were grouped by age and plotted with increasing age. Colours to the left of each subplot show age group. The data underlying this figure can be found at https://doi.org/10.5281/zenodo.12795911. (TIF) [file pbio.3002864.s002.tif]

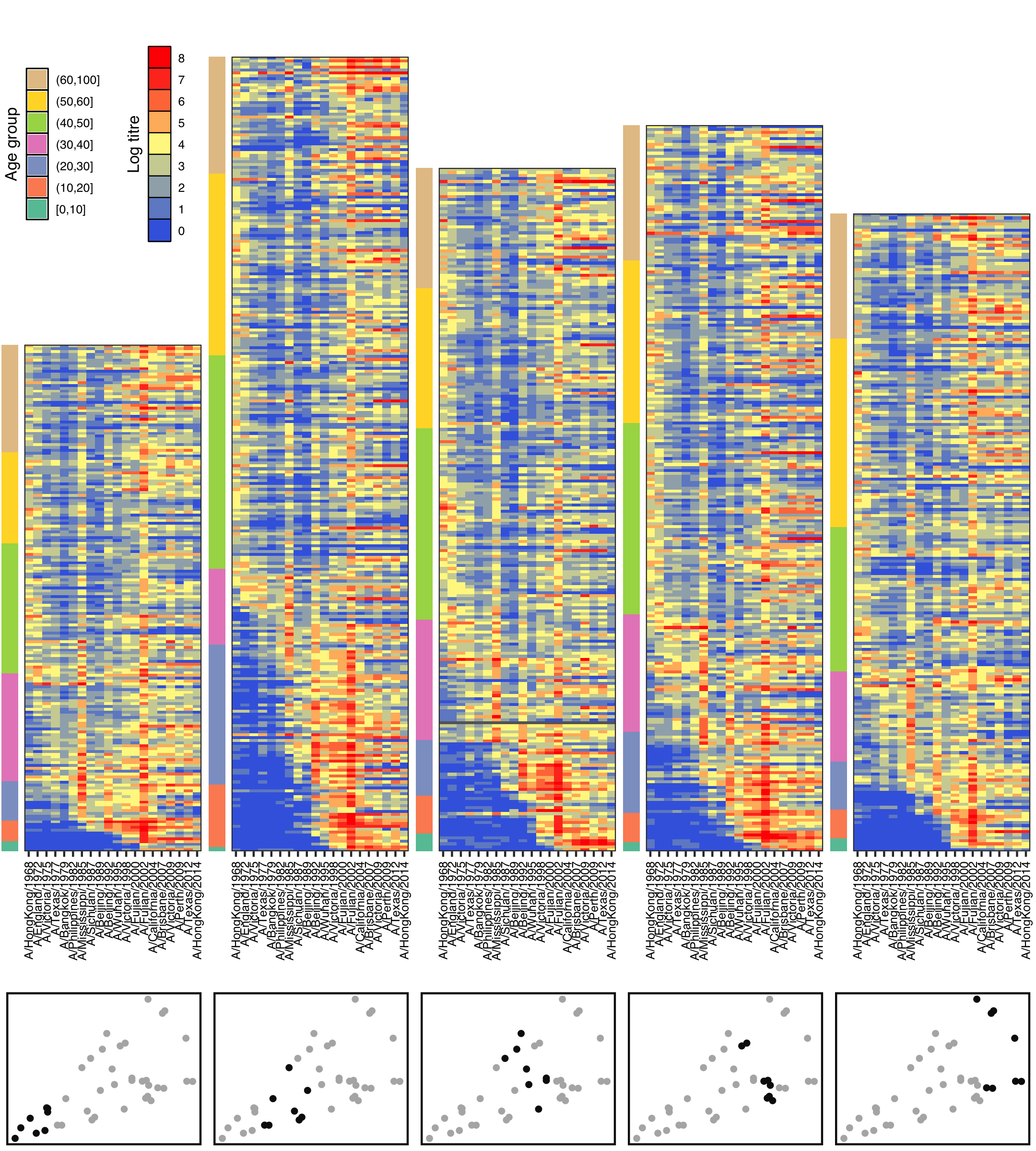

Supplement: S3 Fig — Each cell represents the log HI titre for 1 individual measured against 1 strain, shown on the x-axis. Locations were grouped into quintiles based on increasing distance from Guangzhou city center (bottom panels). Individuals were grouped by age and plotted with increasing age. Colours to the left of each subplot show age group. The data underlying this figure can be found at https://doi.org/10.5281/zenodo.12795911. (TIF) [file pbio.3002864.s003.tif]

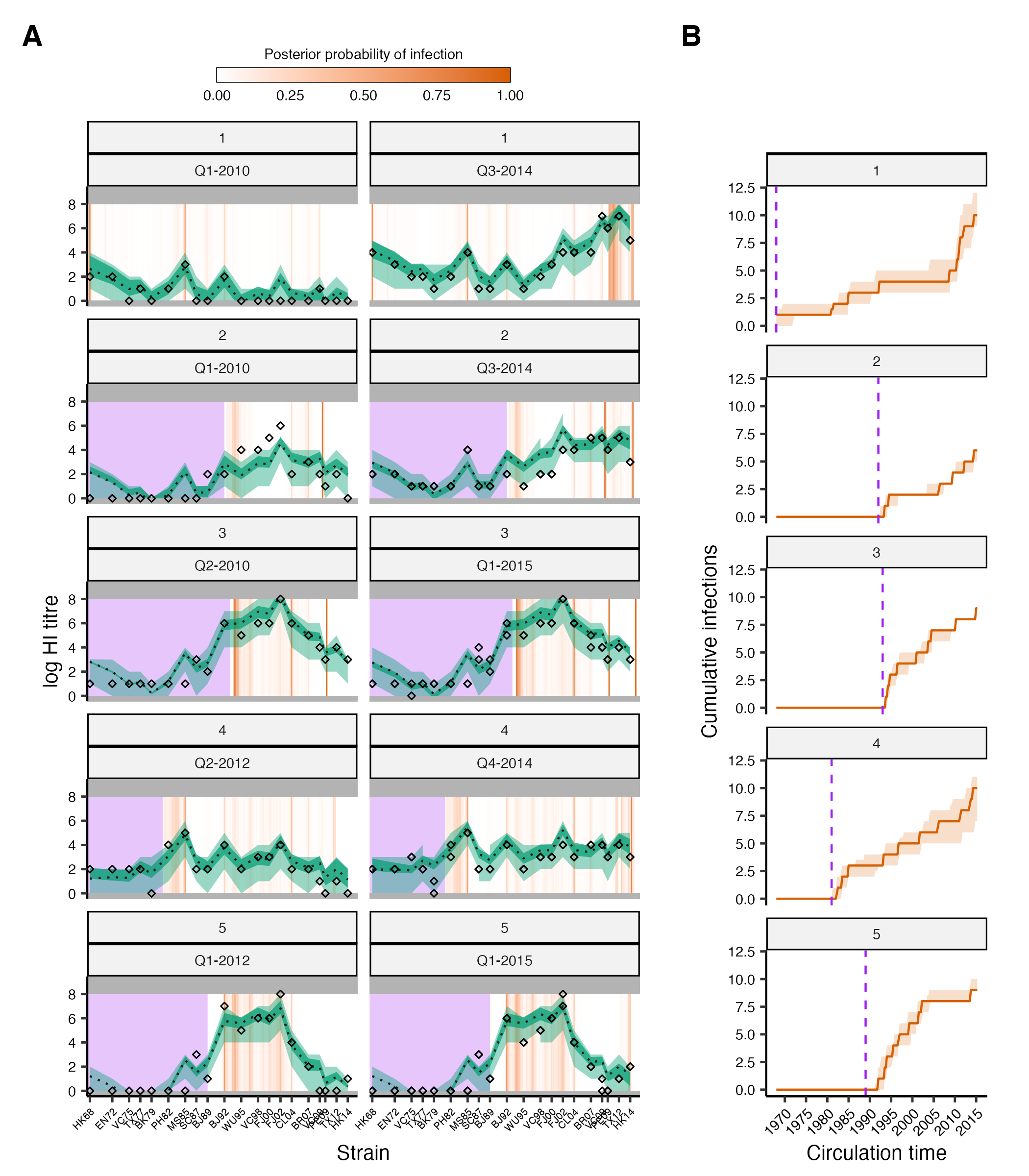

Supplement: S4 Fig — (A) Model-predicted titres compared to observed HI titres at each sampling time for 5 randomly selected individuals. Rows represent individuals. Subplots show antibody titres based on serum samples taken at that time. X-axis represents a position along the antigenic summary path. Black diamonds show observed titres. Black line and green shaded regions show posterior median and 95% credible intervals (CrIs) on model-predicted latent titres (dark green) and 95% prediction intervals (light green). Orange bars show posterior probability of infection in that 3-month window. Grey rectangles denote the limit of detection of the HI assay. Purple rectangles show time periods before birth. (B) Posterior median and 95% CrI for the cumulative number of infections over time from birth (purple dashed line). The data underlying this figure can be found at https://doi.org/10.5281/zenodo.12795911. (TIF) [file pbio.3002864.s004.tif]

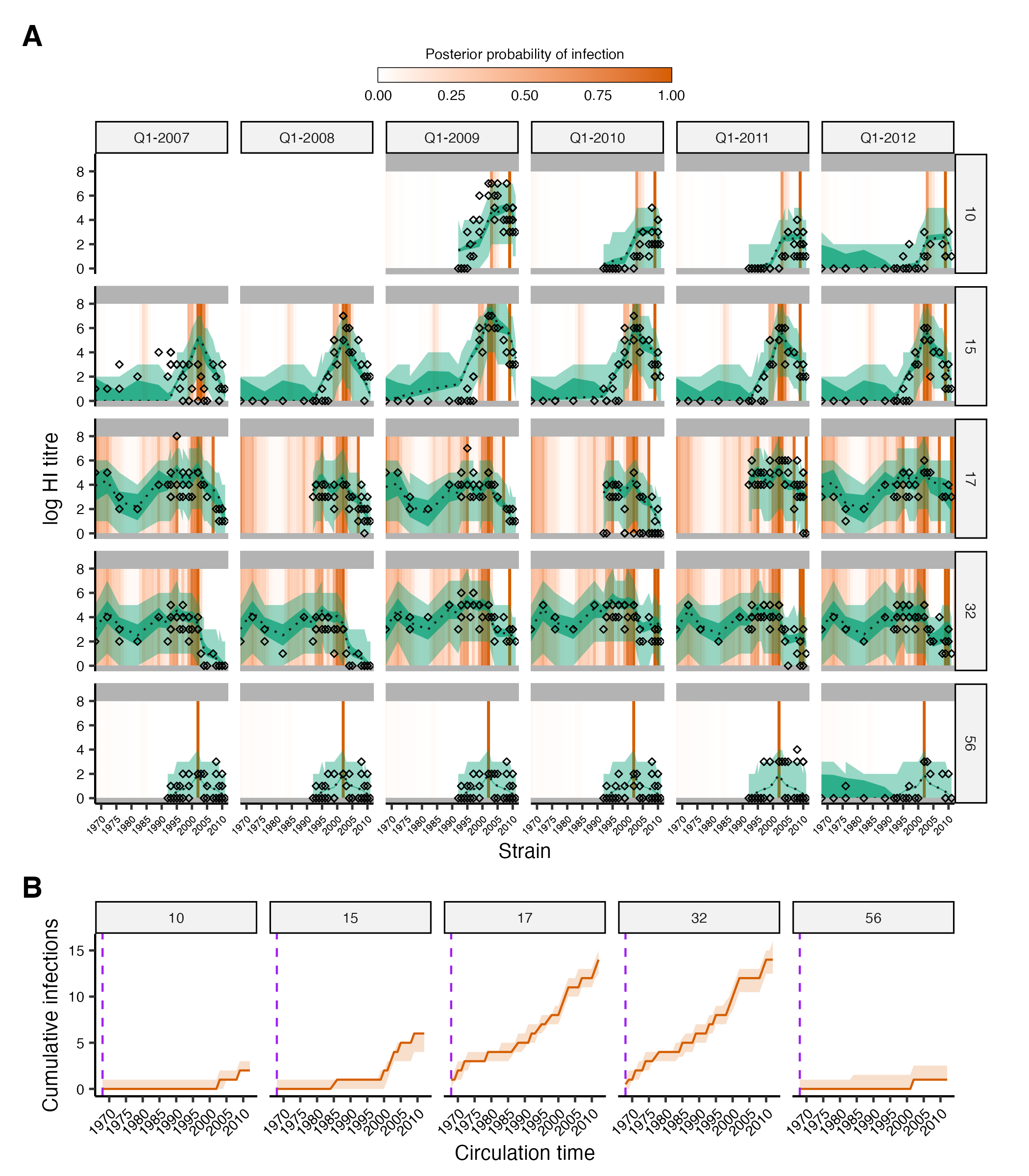

Supplement: S5 Fig — (A) Model-predicted titres compared to observed HI titres at each sampling time for 5 randomly selected individuals, as in S4 Fig. Diamonds show titre measurements; green shaded region shows 95% CrI and 95% prediction intervals; dashed line shows posterior median; orange bars show posterior probability of infection in a given time window. (B) Posterior median and 95% credible intervals (CrI) for the cumulative number of infections over time from birth (orange). Note that date of birth information was not available for these individuals. The data underlying this figure can be found at https://doi.org/10.5281/zenodo.12795911. (TIF) [file pbio.3002864.s005.tif]

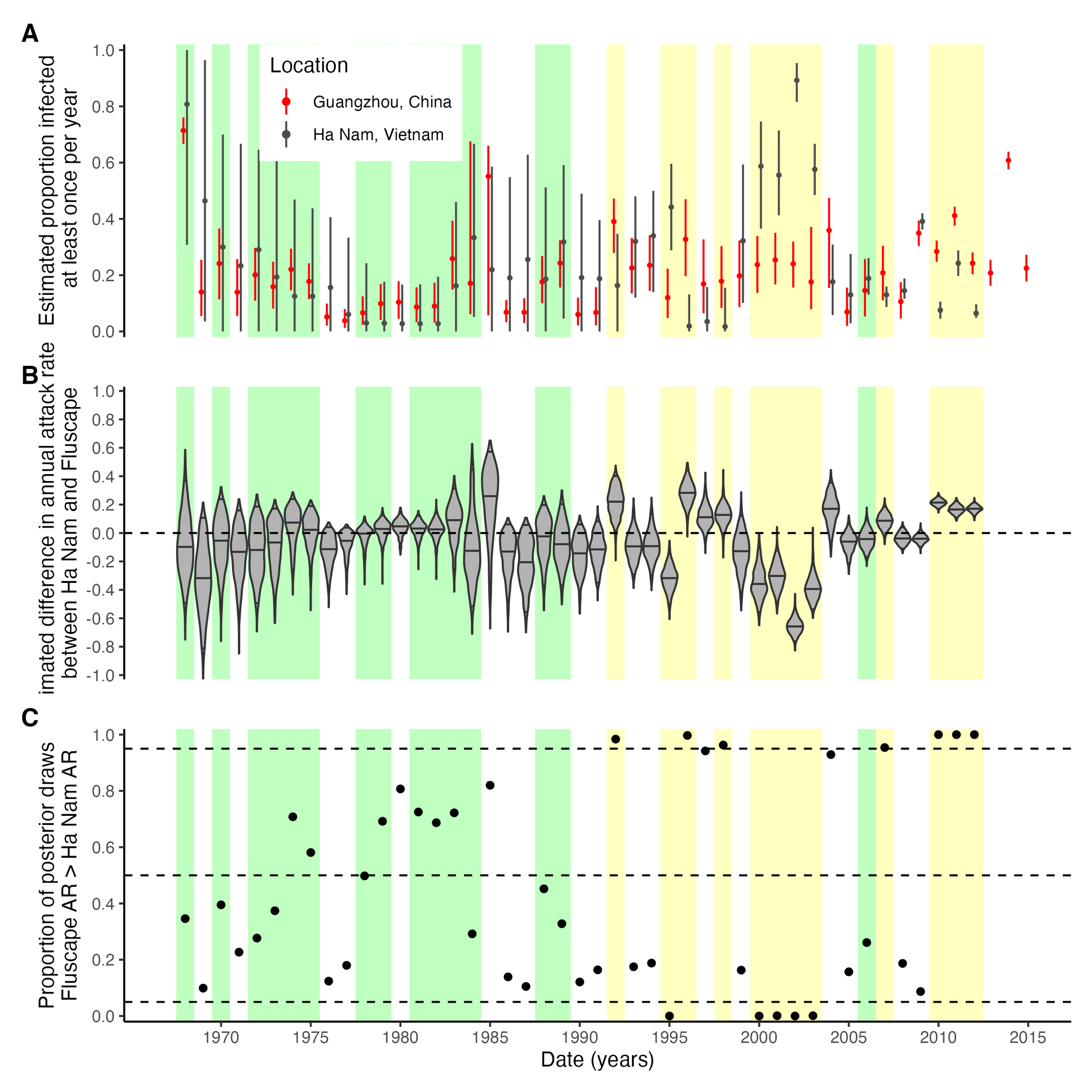

Supplement: S6 Fig — Annual attack rates were defined here as the proportion of individuals who experienced at least 1 infection per year. Yellow shaded regions show time periods where >95% or <5% of posterior samples suggested a greater attack rate in the Fluscape cohort than Ha Nam, whereas green shaded regions show time periods where between 25% and 75% of posterior draws suggested a greater attack rate in the Fluscape cohort. Some time periods showed high uncertainty for the Ha Nam data set, as few individuals in the sample were alive during that time (e.g., 1969–1980). Attack rate estimates were estimated to be higher in the Fluscape cohort from 2000 to 2003 inclusive with more than 95% posterior probability. This might reflect a genuine different in A/H3N2 epidemiology during that time, but may also be partially driven by systematic biases in titre measurements to strains isolated during that time period—the fits to the Fluscape data include a positive offset term for titres against A/Fujian/2002, which leads to lower attack rate estimates in that time period, whereas fits to the Ha Nam data do not. The time period from 2010 to 2012 also shows higher attack rates in the Fluscape cohort but with similar relative patterns. The data underlying this figure can be found at https://doi.org/10.5281/zenodo.12795911. (TIF) [file pbio.3002864.s006.tif]

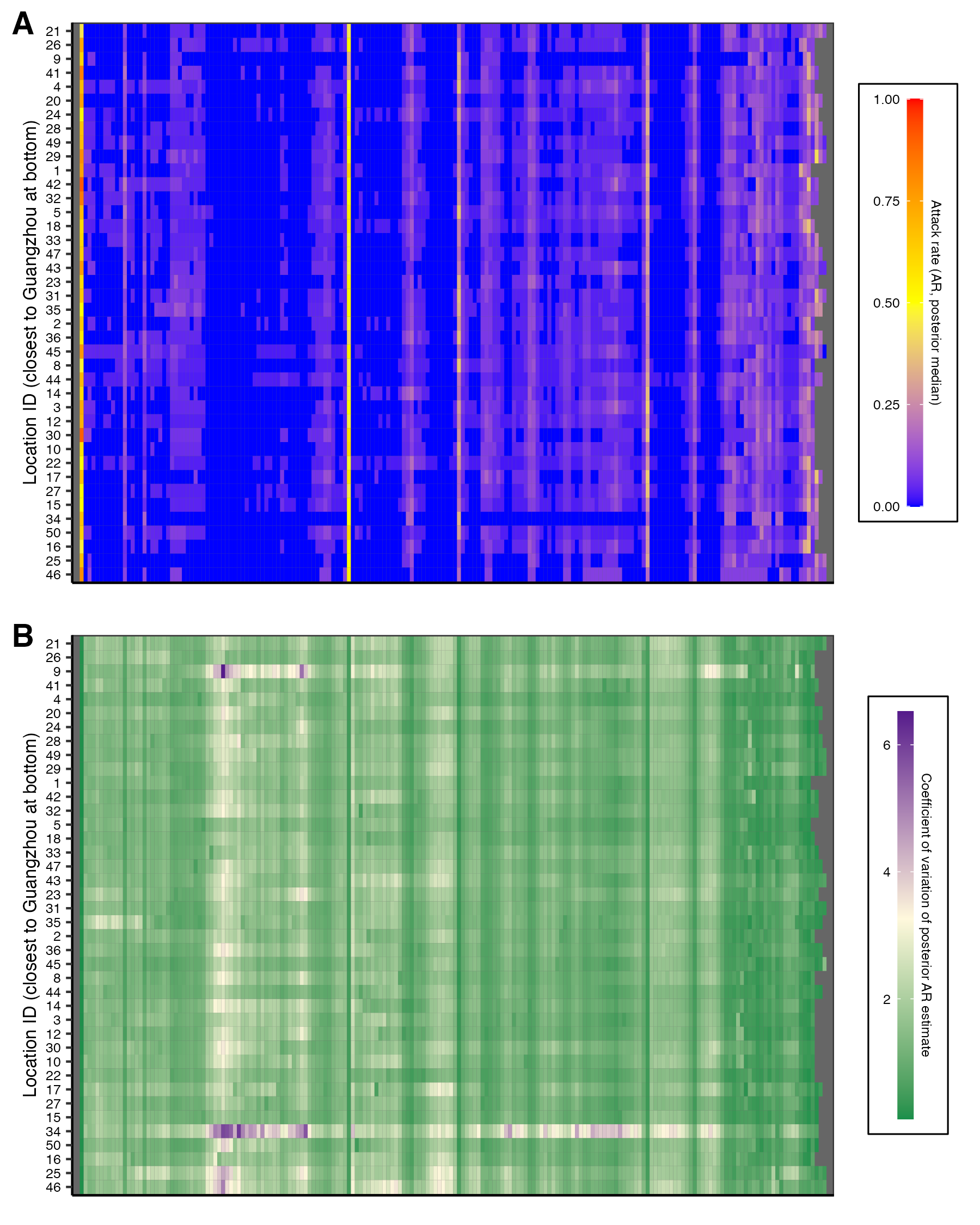

Supplement: S7 Fig — Each row represents 1 study location ordered by increasing distance from Guangzhou city center. Each column represents a 3-month period. Cells are shaded by (A) the posterior median inferred attack rate or (B) the coefficient of variation of the posterior quarterly attack rate estimate for each location. The data underlying this figure can be found at https://doi.org/10.5281/zenodo.12795911. (TIF) [file pbio.3002864.s007.tif]

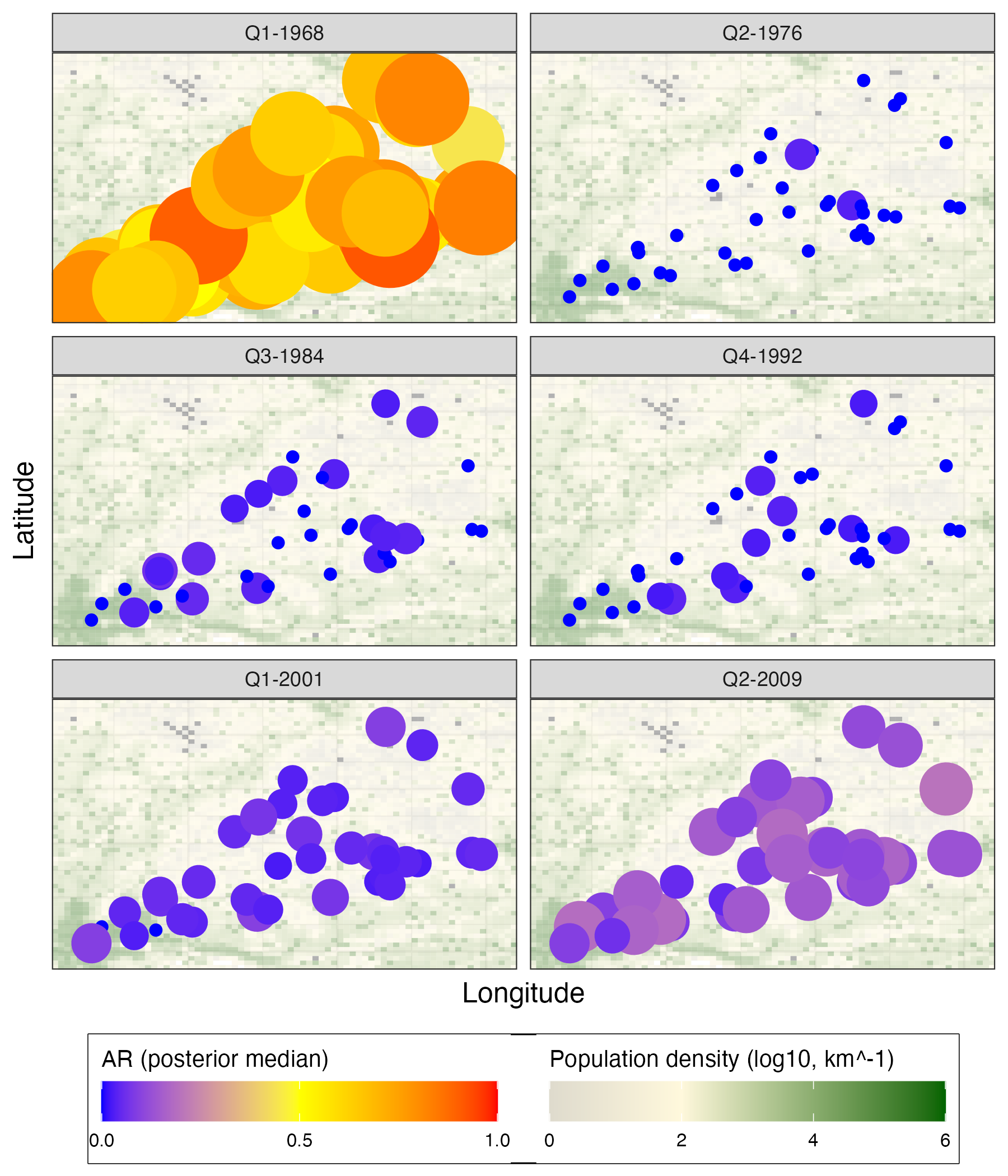

Supplement: S8 Fig — Each panel is one frame from a full animation available in S1 Video. Each coloured point shows the inferred attack rate in each of the 40 locations, with size and shading reflecting the posterior median attack rate. Underlying the plot is a map of the study area, with each grid cell shaded by its log10 population density. The data underlying this figure can be found at https://doi.org/10.5281/zenodo.12795911. (TIF) [file pbio.3002864.s008.tif]

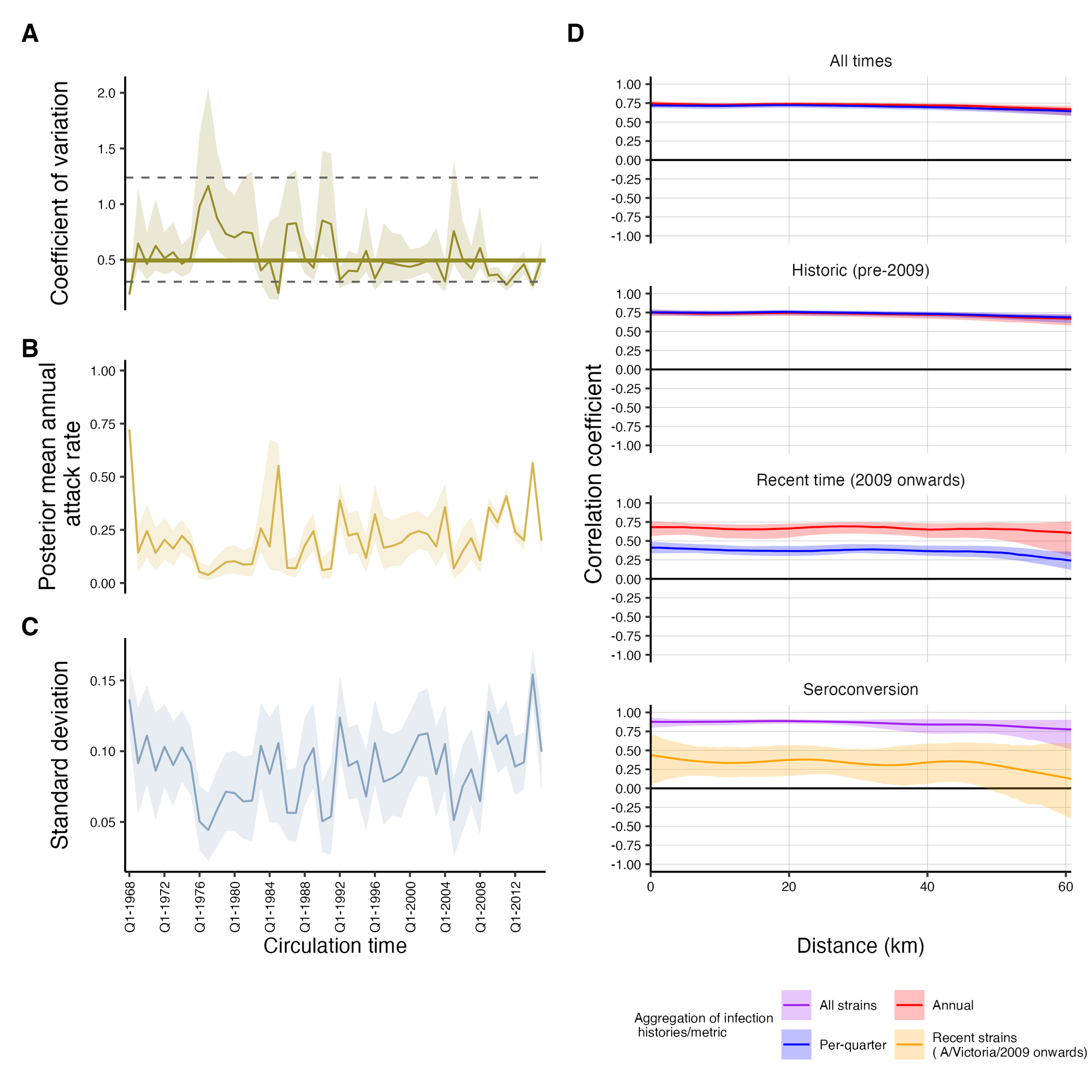

Supplement: S9 Fig — (A) Coefficient of variation, (B) overall mean, and (C) standard deviation of posterior median annual attack rate estimates from the 40 study locations. Solid lines and shaded regions show posterior medians and 95% credible intervals. In (A), the solid horizontal line shows the overall mean coefficient of variation across all time. Dashed horizontal lines show 95% quantiles of simulated coefficients of variation under the assumption that attack rates are the same across space. (D) Fitted spline correlograms showing spatiotemporal correlation in attack rates and proportion seroconverted with increasing distance. The first 3 plots show the spline correlogram calculated using the Scnf function from the ncf R-package for each of 100 posterior samples for the 40 location-specific attack rates. Solid lines and shaded regions show median and 95% quantiles of the predicted covariance function for these 100 samples, coloured by the level of aggregation used to calculate the attack rates. Each subplot shows the same calculation using either attack rates from all times, prior to Q1-2009 or Q1-2009 onwards. For the final plot (“Seroconversion”), we calculated the spatial correlation in the proportion seroconverted in each of the 40 study locations, treating strain isolation time as the time variable. Solid lines and shaded regions show median and 95% quantiles of 1,000 bootstrapped observations. We repeated the analysis using either seroconversion to all strains, or only A/Victoria/2009, A/Perth/2009, A/Texas/2012, and A/HongKong/2014. The data underlying this figure can be found at https://doi.org/10.5281/zenodo.12795911. (TIF) [file pbio.3002864.s009.tif]

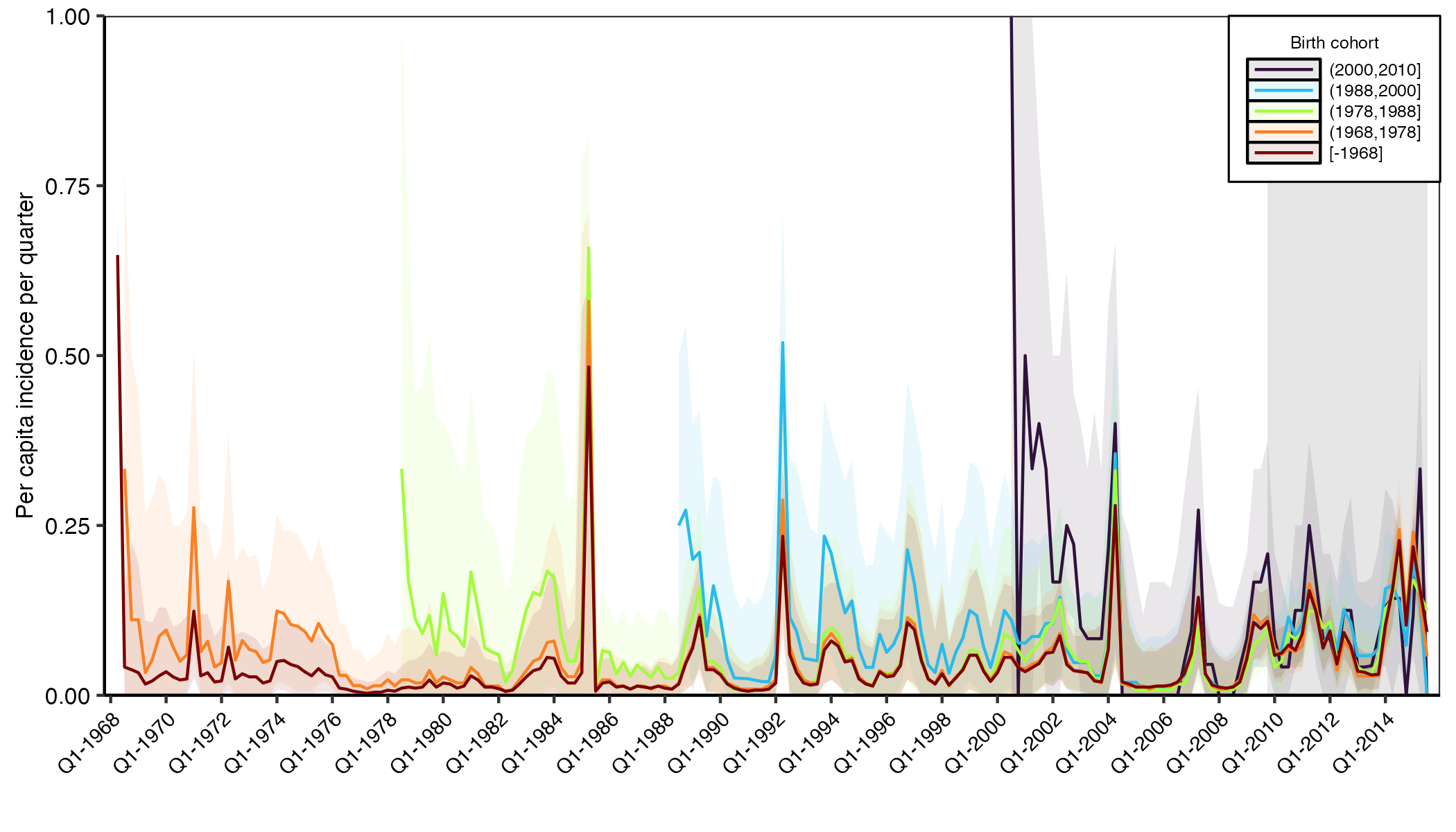

Supplement: S10 Fig — Model predicted per-capita incidence per quarter stratified into 5 birth cohorts. Attack rates were estimated by dividing the number of inferred infections by the number alive in each birth cohort in each 3-month period. Solid lines show the posterior median estimate from 1,000 posterior samples. Shaded regions show 95% credible intervals from 1,000 posterior samples. Grey shaded box shows duration of the Fluscape study. The data underlying this figure can be found at https://doi.org/10.5281/zenodo.12795911. (TIF) [file pbio.3002864.s010.tif]

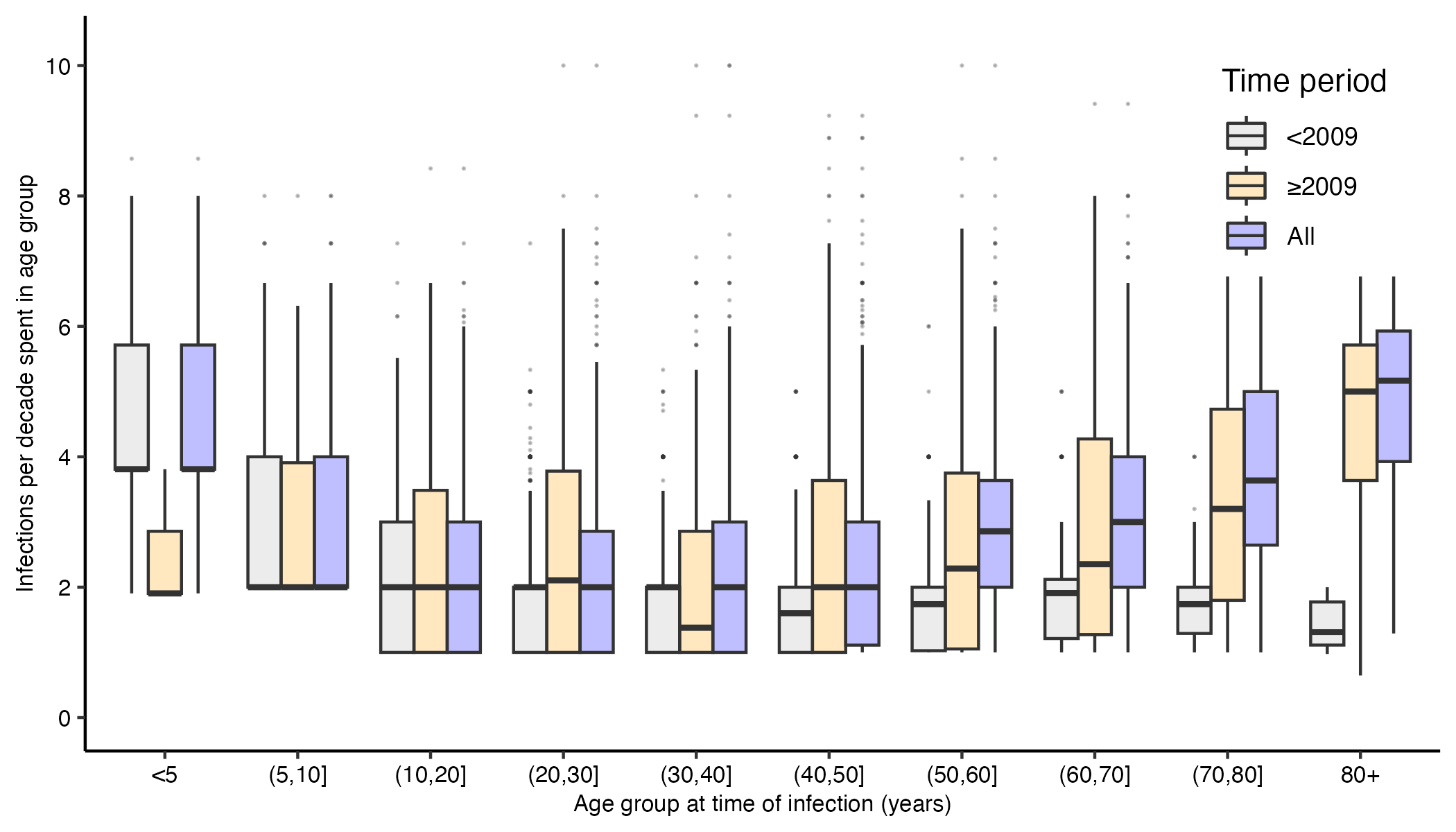

Supplement: S11 Fig — Excludes infection states for individuals who spent less than 2 years in that age group. We present infection rate estimates using only infections from time periods prior to the first serum sample in Q4-2009 in Fig 3C. This is because there are many individuals representing the oldest age group at time of infection for time periods post Q4-2009, but relatively few from pre Q4-2009 (as individuals who were very old in historical time periods are no longer alive). In contrast, younger age groups are better represented across historical time periods (as those individuals are still alive at the time of sampling). Combining this biased representation of older individuals with much higher estimated incidence rates in recent time periods weighs the infection rate estimates for the older age groups much higher simply because most of their infections come from this time period. Therefore, we present age-stratified infection rate estimates using only pre Q4-2009 infections in the main text. The data underlying this figure can be found at https://doi.org/10.5281/zenodo.12795911. (TIF) [file pbio.3002864.s011.tif]

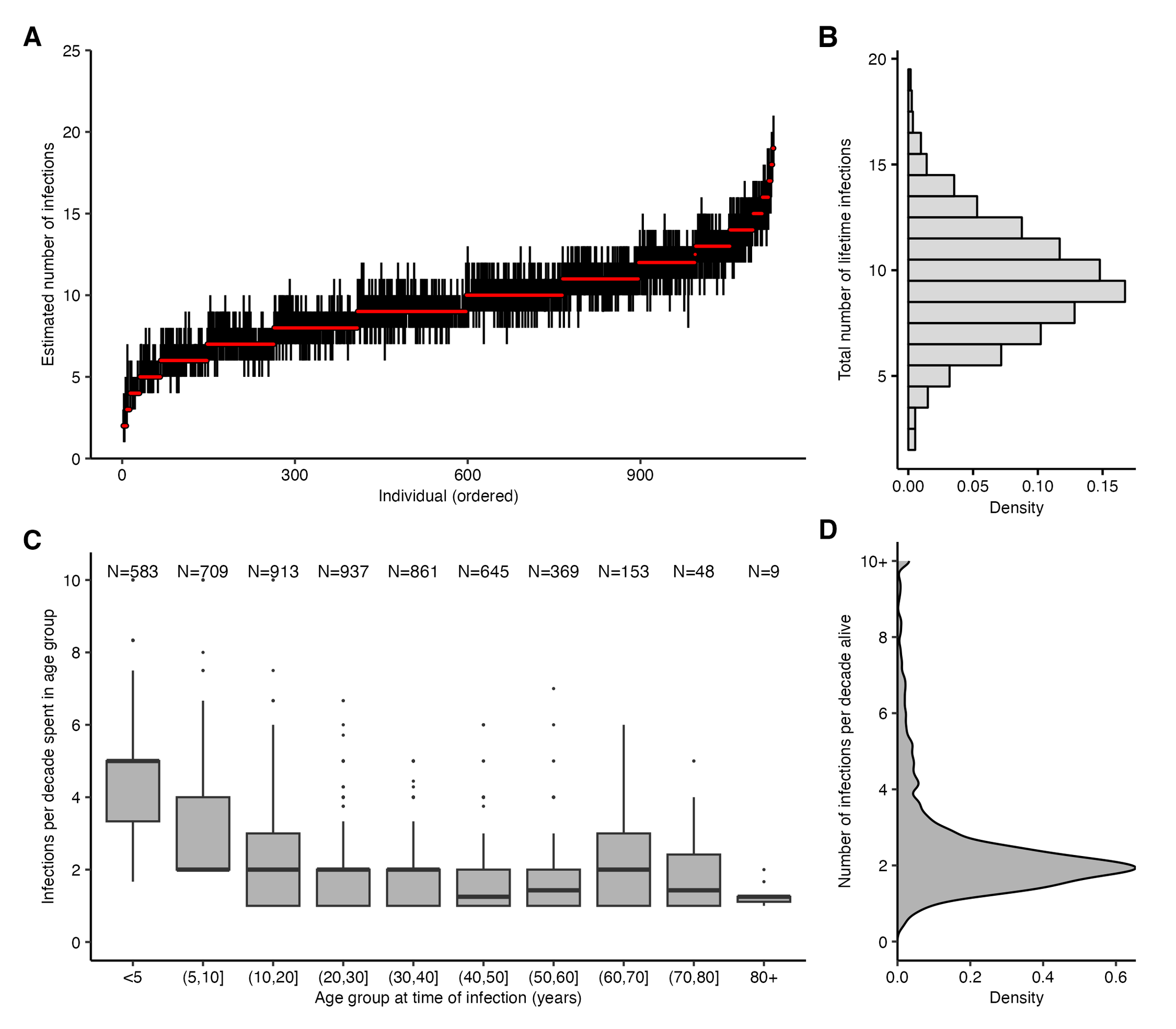

Supplement: S12 Fig — Results shown are identical to those in Fig 3, but assuming that (i) individuals can only be infected once per year (i.e., annual resolution infection histories rather than quarterly); (ii) the infection history model is placed upon an individual’s total number of lifetime infections and not their per-time probability of infection (see [35] for further detail on implications of different prior assumptions); (iii) we did not remove runs of continuous infections from the posteriors. (A) Pointrange plot shows median and 95% CrI on the total number of lifetime infections for each individual, ordered by increasing age. (B) Distribution of the total number of infections across all individuals based on the posterior median total number of infections. (C) Posterior median number of infections per 10-year period stratified by age group at the time of infection, excluding individuals who spent less than 2 years in that age group, and including only time periods prior to Q4-2009. Text shows sample size within each age group—note this does not sum to the number of individuals in the sample, as individuals contribute to multiple age groups during their lifetime. (D) Posterior median number of infections per 10 years alive across all individuals. Under this prior, we estimated that individuals are infected 2.08 times per 10-year period (posterior median; 95% CrI: 1.04–7.62). The data underlying this figure can be found at https://doi.org/10.5281/zenodo.12795911. (TIF) [file pbio.3002864.s012.tif]

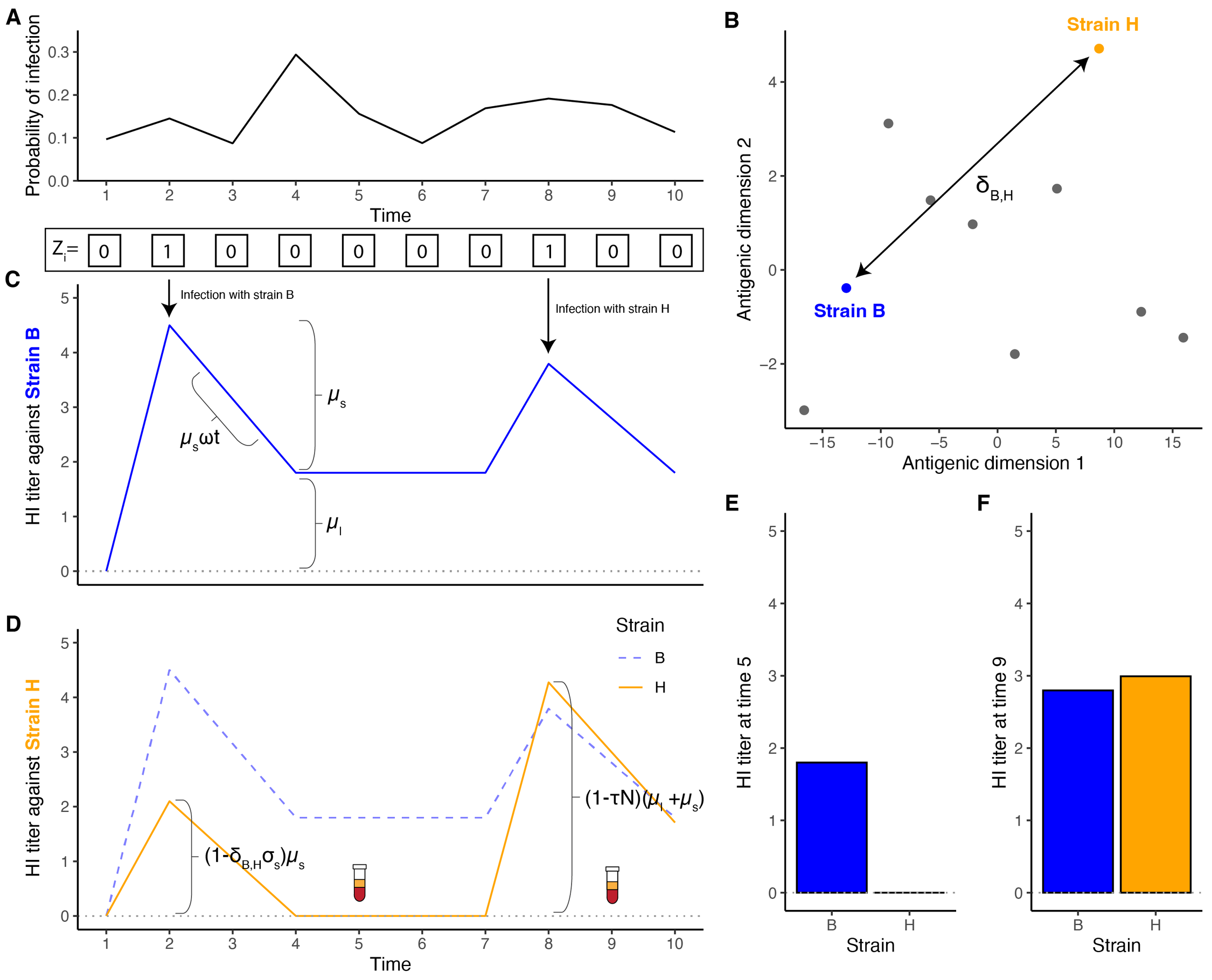

Supplement: S13 Fig — (A) Example, randomly generated population-level infection probabilities. At the population level, the model describes a per-time-period probability of infection applied to the whole population. These probabilities are used to simulate a vector of latent binary infection states, Zi, for each individual as a series of independent Bernoulli trials (shown as a vector of 1s and 0s). (B) The antigenic relatedness of A/H3N2 strains is given by an antigenic map, where the degree of cross reactivity between any 2 strains is given by their Euclidean distance on the map. (C) Antibody levels against the infecting strain (strain B) are boosted and wane, given by the summation of transient short-term boosting and persistent long-term boosting. (D) Infection with strain B also induces cross-reactive antibodies against all other strains, here showing antibody levels to strain H. The degree of cross-reactivity is proportional to the antigenic distance between the infecting and measured strain. Later on, the individual is infected again, this time with strain H, inducing further antibody boosting and waning. An antigenic seniority parameter, τ, reduces each successive boost as a function of the number of previous infections, N. Snapshots of these underlying antibody kinetics are observed through serum samples (blood vials; (E) and (F)) distributed according to a truncated, discretized normal distribution with standard deviation parameter ε. (TIF) [file pbio.3002864.s013.tif]

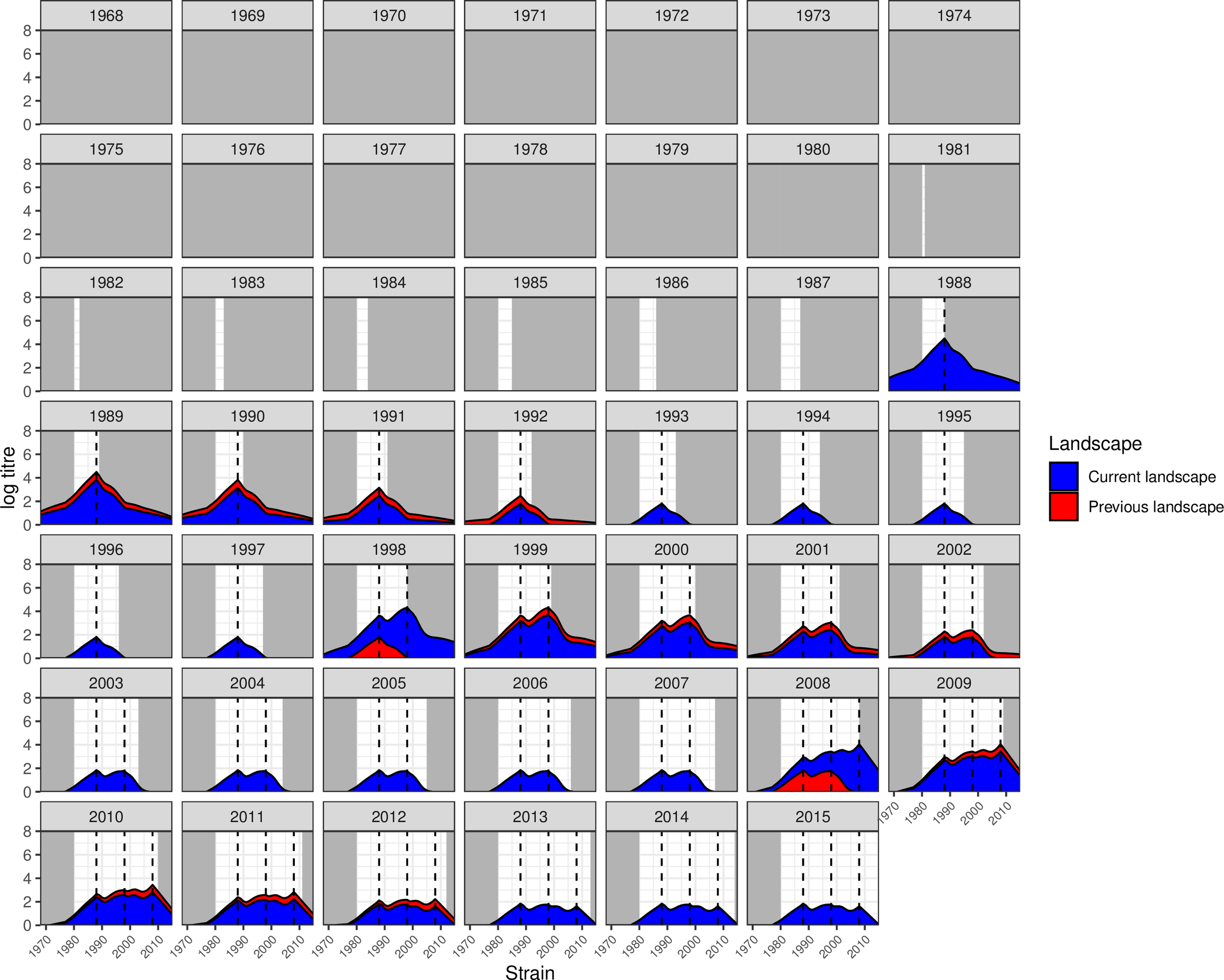

Supplement: S14 Fig — Each subplot shows the antibody landscape for that time period. The blue region gives the antibody landscape in that time period, whereas the red region gives the antibody landscape in the preceding time period. The x-axis of each subplot gives the identity of the strain assumed to be circulating in that year. The grey region shows the time period and thus strains that circulated before the individual was born. Vertical dashed lines give the timing/strains the individual was infected with. (TIF) [file pbio.3002864.s014.tif]

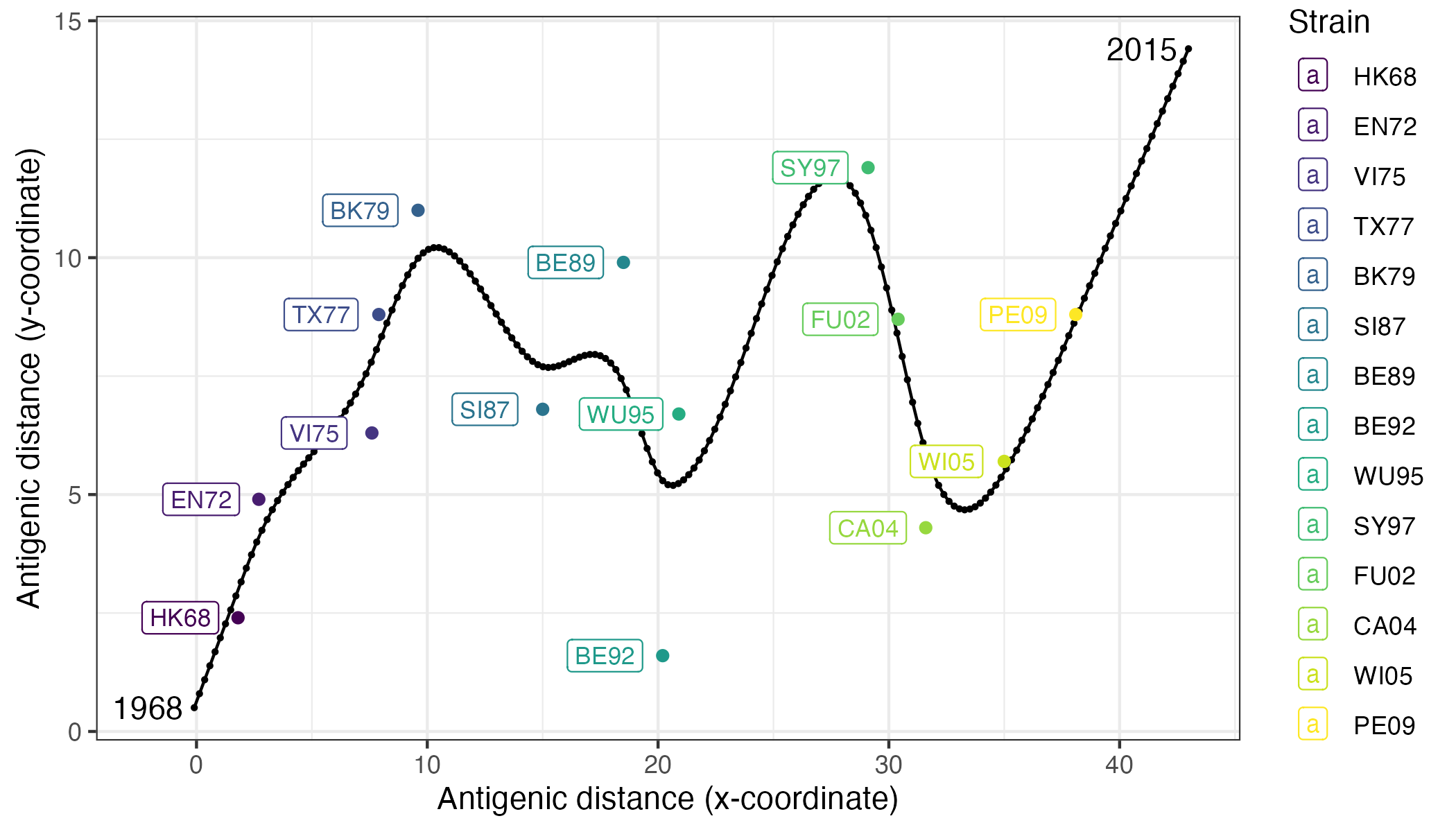

Supplement: S15 Fig — Each coloured point shows the location of the labelled strain on the antigenic map given in [37]. Strains which are further apart are less antigenically similar, and therefore exhibit less cross-reactivity following a seroresponse. The smoothing spline shows the inferred coordinates of each strain j assumed to have circulated in each 3-month time period, where each black point shows the assumed location in successive time periods. First, a cubic smoothing spline was fitted to the locations of the measured strain with smoothing parameter 0.3. Second, a linear model was fitted to predict the x-coordinate as a function of the strain isolation time. Finally, we generated predicted x-coordinates for each possible j given the circulation time from the linear model, and then used the predicted x-coordinate to predict the y-coordinate from the fitted smoothing spline. The antigenic distance between each pair of strains k and j was then calculated based on their Euclidean distance. The data underlying this figure can be found at https://doi.org/10.5281/zenodo.12795911. (TIF) [file pbio.3002864.s015.tif]

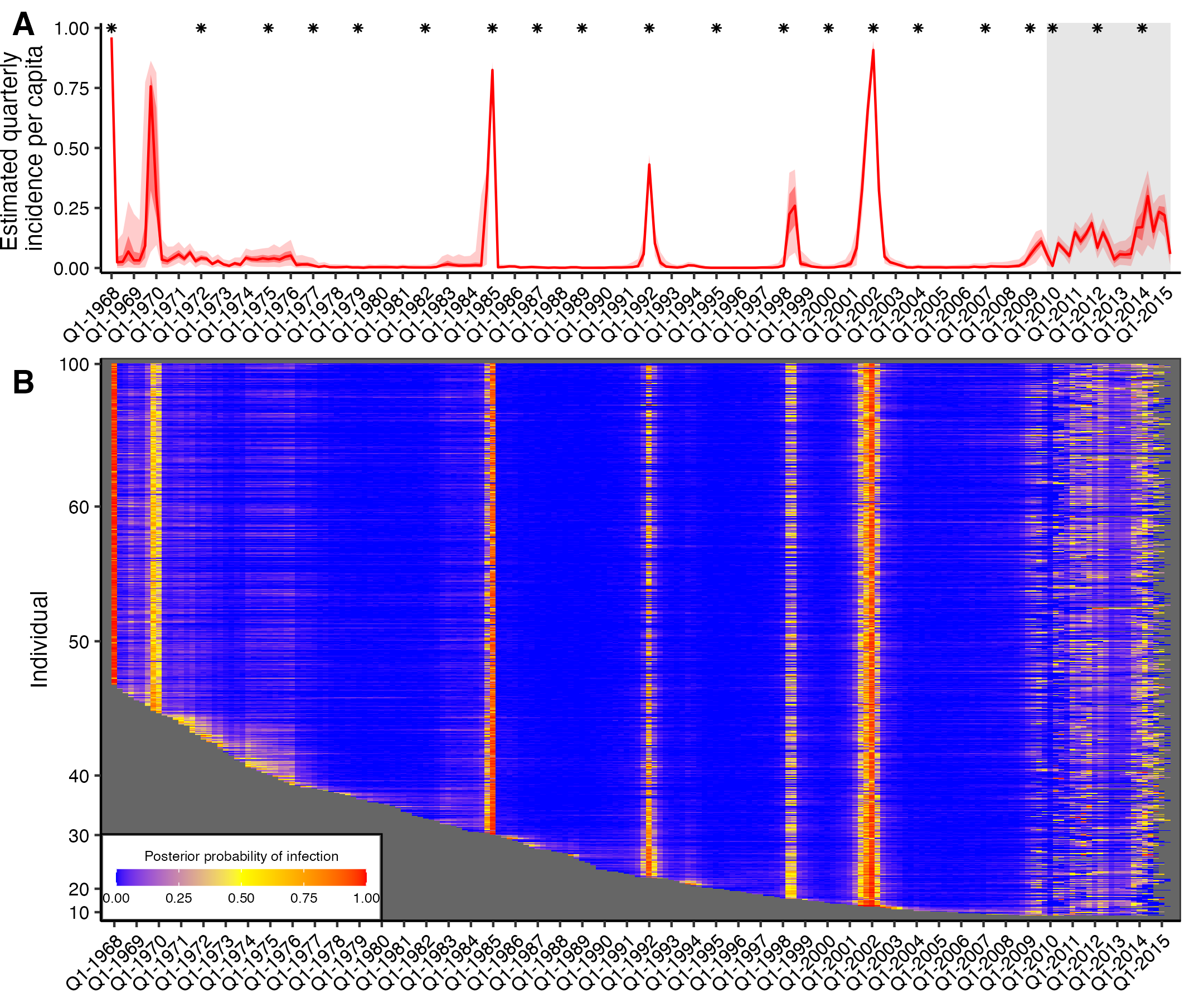

Supplement: S16 Fig — Identical to Fig 2, but without the inclusion of strain-specific measurement offsets in the observation model. (A) Model predicted per-capita incidence per quarter. Attack rates were estimated by dividing the number of inferred infections by the number alive in each 3-month period. Red line shows the posterior median estimate from 1,000 posterior samples. Dark and light red shaded regions show 50% and 95% credible intervals respectively from 1,000 posterior samples. Grey shaded box shows duration of the Fluscape study. Asterisks mark times from which a sample circulating strain was tested. (B) Inferred infection histories for each individual. Each row represents an individual ordered by increasing age in years. Each column represents the time of a potential infection. Cells are shaded based on the number of the posterior samples with an infection at that time divided by the total number of posterior samples for that infection state. The data underlying this figure can be found at https://doi.org/10.5281/zenodo.12795911. (TIF) [file pbio.3002864.s016.tif]

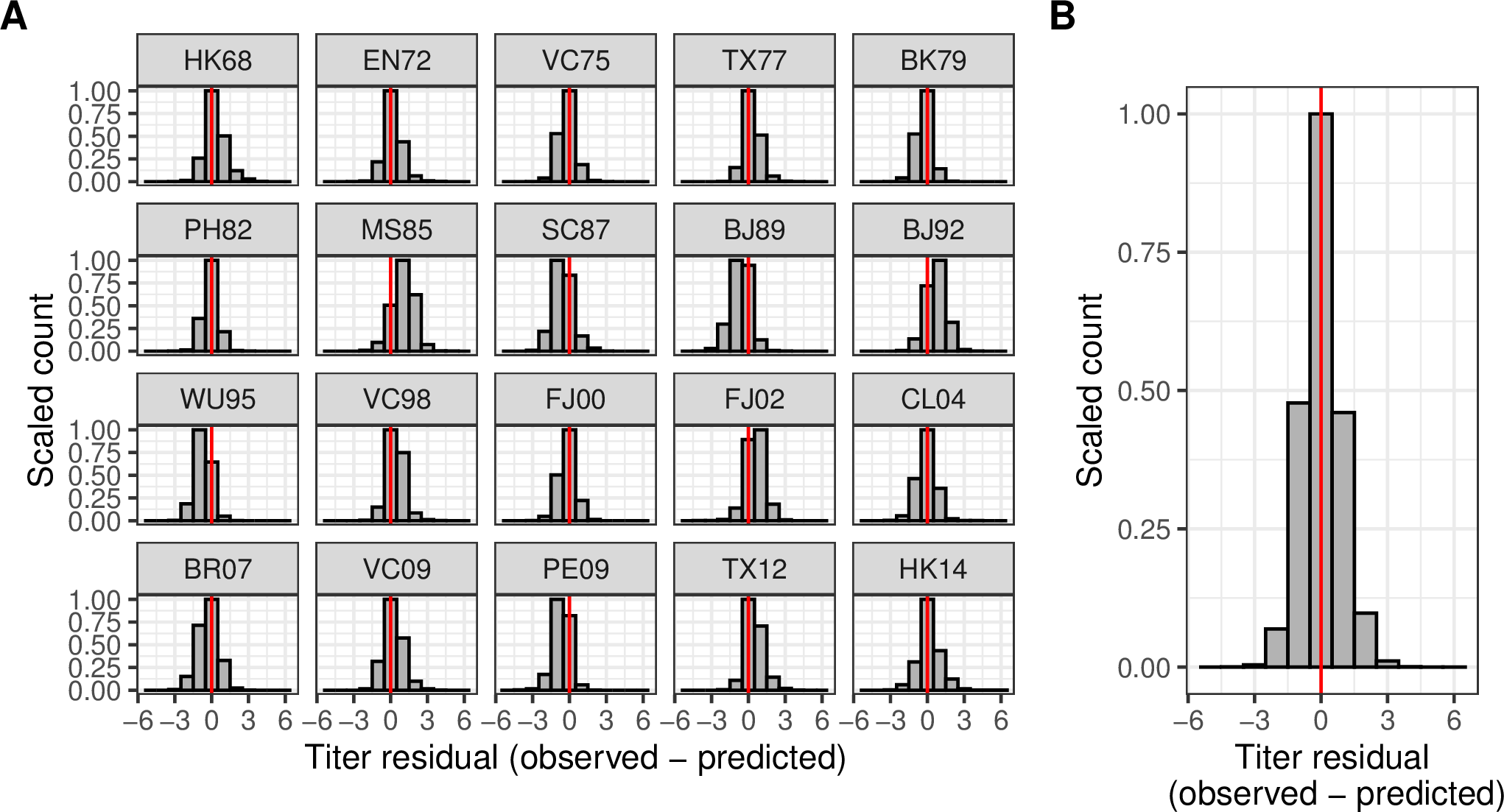

Supplement: S17 Fig — (A) Distribution of titre prediction errors stratified by tested A/H3N2 strain. (B) Overall distribution of titre prediction errors across all measured viruses. Vertical red line shows x = 0; buckets to the right of the red line suggest underestimation of titres; histograms to the left of the red line buckets suggest overestimation of titres. The data underlying this figure can be found at https://doi.org/10.5281/zenodo.12795911. (TIF) [file pbio.3002864.s017.tif]

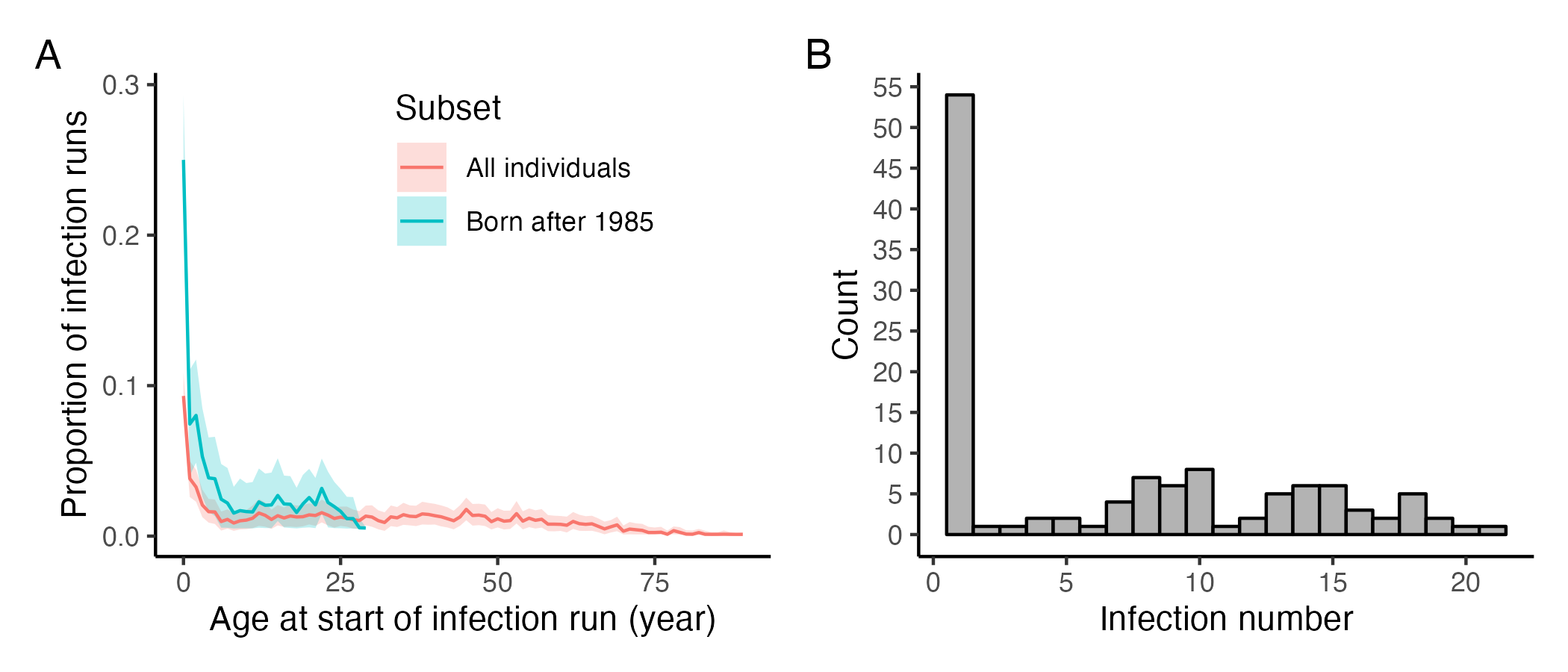

Supplement: S18 Fig — (A) Proportion of infection episodes which are estimated as runs of consecutive infections by age at start of infection run, using either all infection episodes or only those from individuals born after 1985. (B) Number of inferred infection episodes which are estimated as runs of consecutive infections stratified by the infection order in each individual’s infection history. For example, if the run is the first infection an individual has experienced, this is given an infection number of one. Of the 10,558 distinct infection episodes, 757 (posterior median; 95% CrI: 676–861) were runs of 2 consecutive infections, 79 (posterior median; 95% CrI: 62–97) were runs of 3 consecutive infections and 19 (posterior median; 95% CrI: 13–27) were runs of 4 or more. The data underlying this figure can be found at https://doi.org/10.5281/zenodo.12795911. (TIF) [file pbio.3002864.s018.tif]

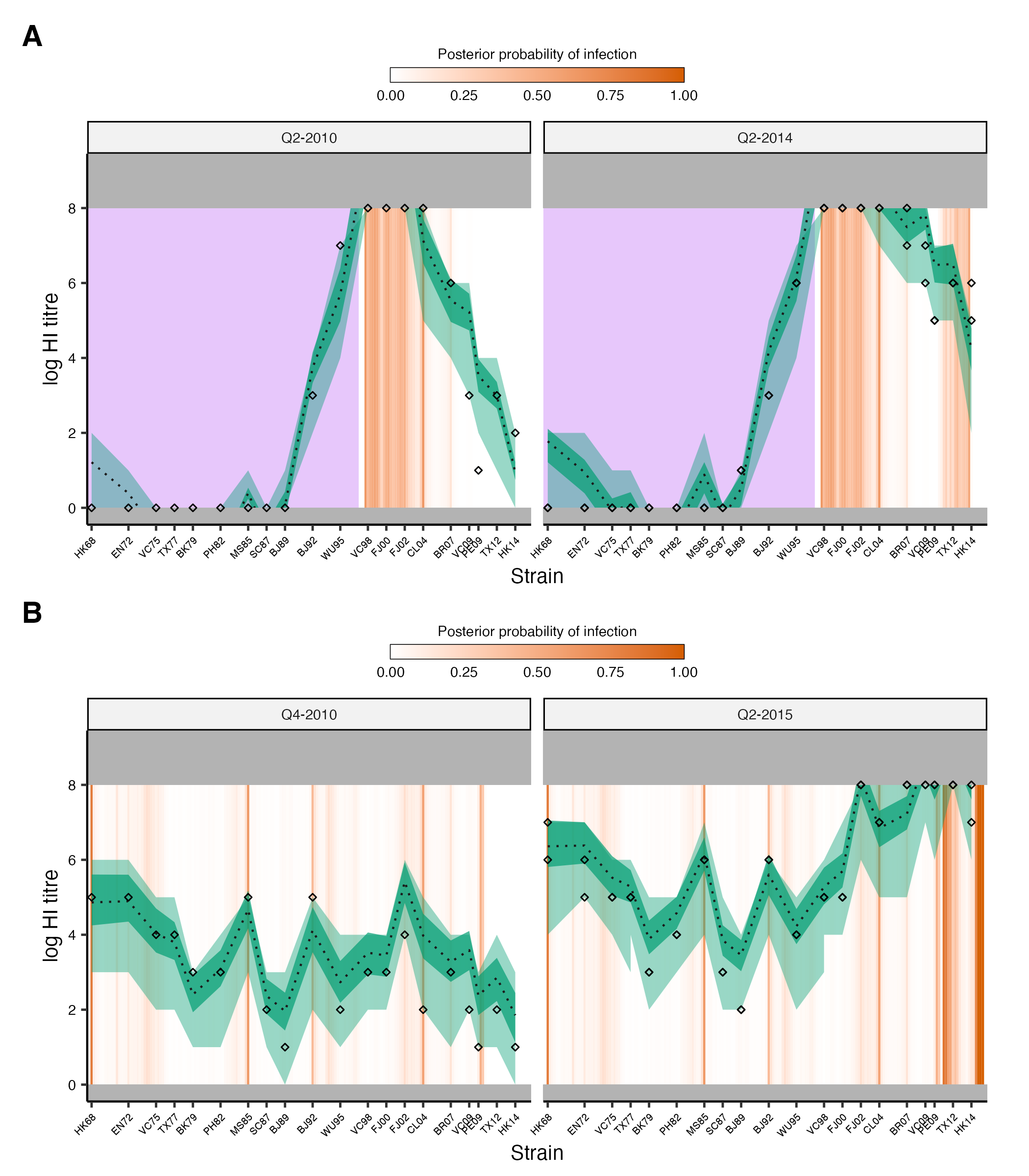

Supplement: S19 Fig — Model-predicted titres compared to observed HI titres at each sampling time for 2 individuals, as in S4 Fig. Diamonds show titre measurements; green shaded region shows 95% CrI and 95% prediction intervals; dashed line shows posterior median; orange bars show posterior probability of infection in a given time window. Purple rectangles show time periods prior to birth. (A) Example of an individual estimated to have experienced multiple consecutive infections immediately following birth to explain high titres. (B) Example of an individual estimated to have experienced multiple consecutive infections between the 2 serum sampling times to explain the drastic increase in titres to recently circulating strains. The data underlying this figure can be found at https://doi.org/10.5281/zenodo.12795911. (TIF) [file pbio.3002864.s019.tif]

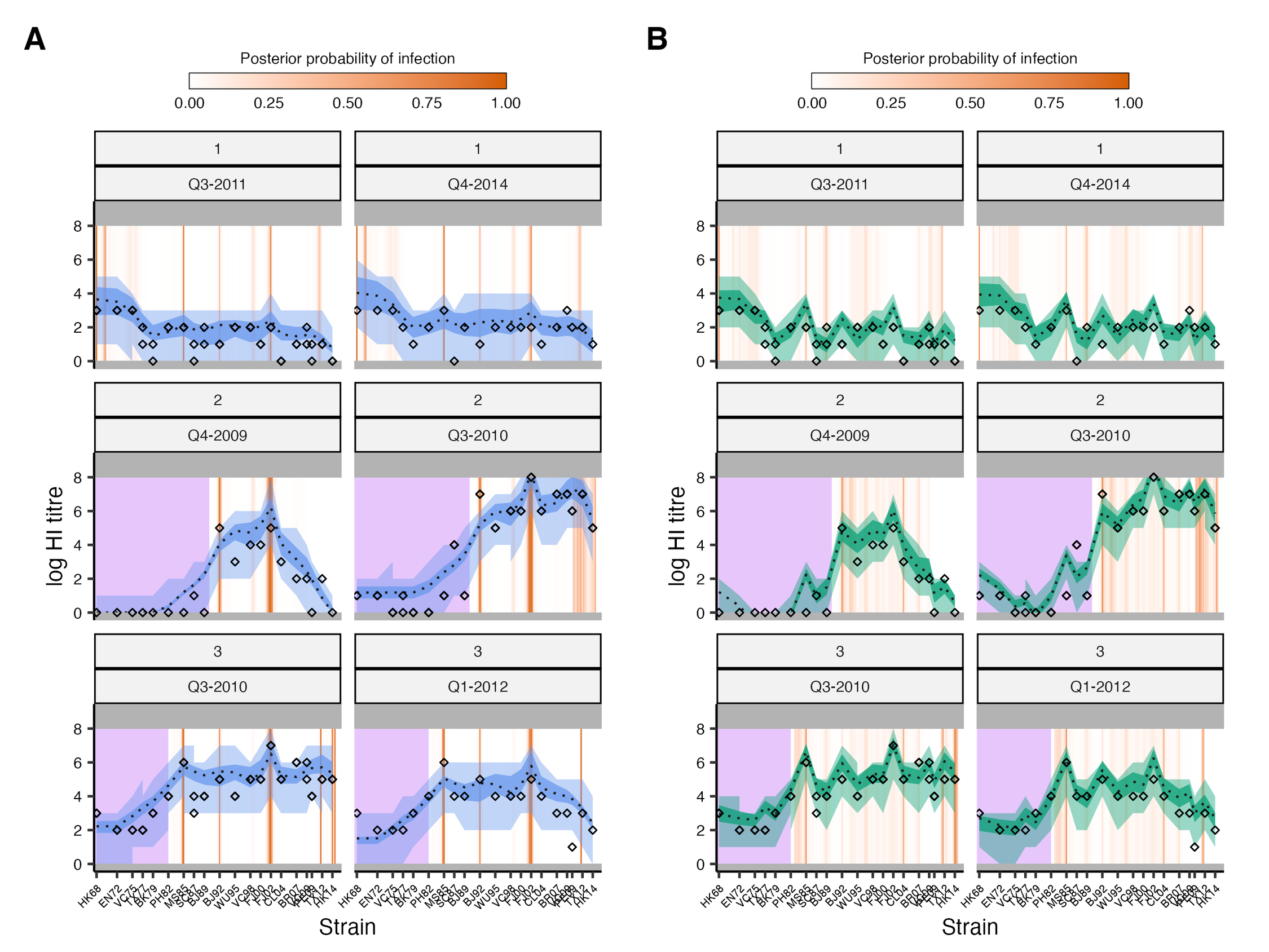

Supplement: S24 Fig — Rows represent individuals. Subplots show antibody titres based on serum samples taken at that time. X-axis represents a position along the antigenic summary path. Black diamonds show observed titres. Black line and blue or green shaded regions show posterior median and 95% credible intervals (CrI) on model-predicted latent titres (dark blue/green) and 95% prediction intervals (light blue/green). Orange bars show posterior probability of infection in that 3-month window. Grey rectangles denote the limit of detection of the HI assay. Purple rectangles show time periods prior to birth. (A) Model-predicted titres compared to observed HI titres at each sampling time for 3 randomly selected individuals, as in S5 Fig, but from fitting the model without strain-specific measurement offsets. (B) as in (A), but with the estimated strain-specific measurement offsets included. The data underlying this figure can be found at https://doi.org/10.5281/zenodo.12795911. (TIF) [file pbio.3002864.s024.tif]

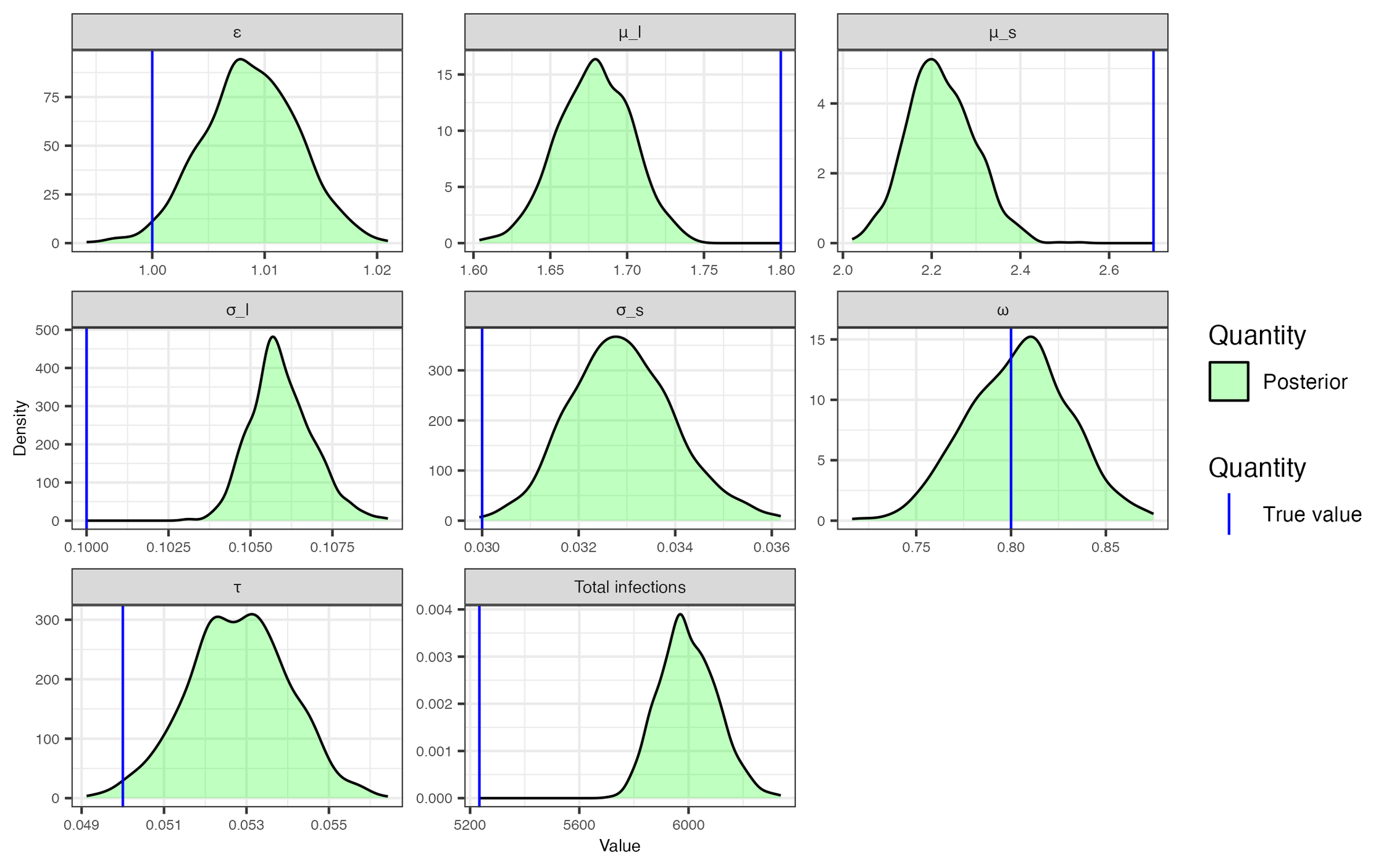

Supplement: S25 Fig — The data underlying this figure can be found at https://doi.org/10.5281/zenodo.12795911. (TIF) [file pbio.3002864.s025.tif]

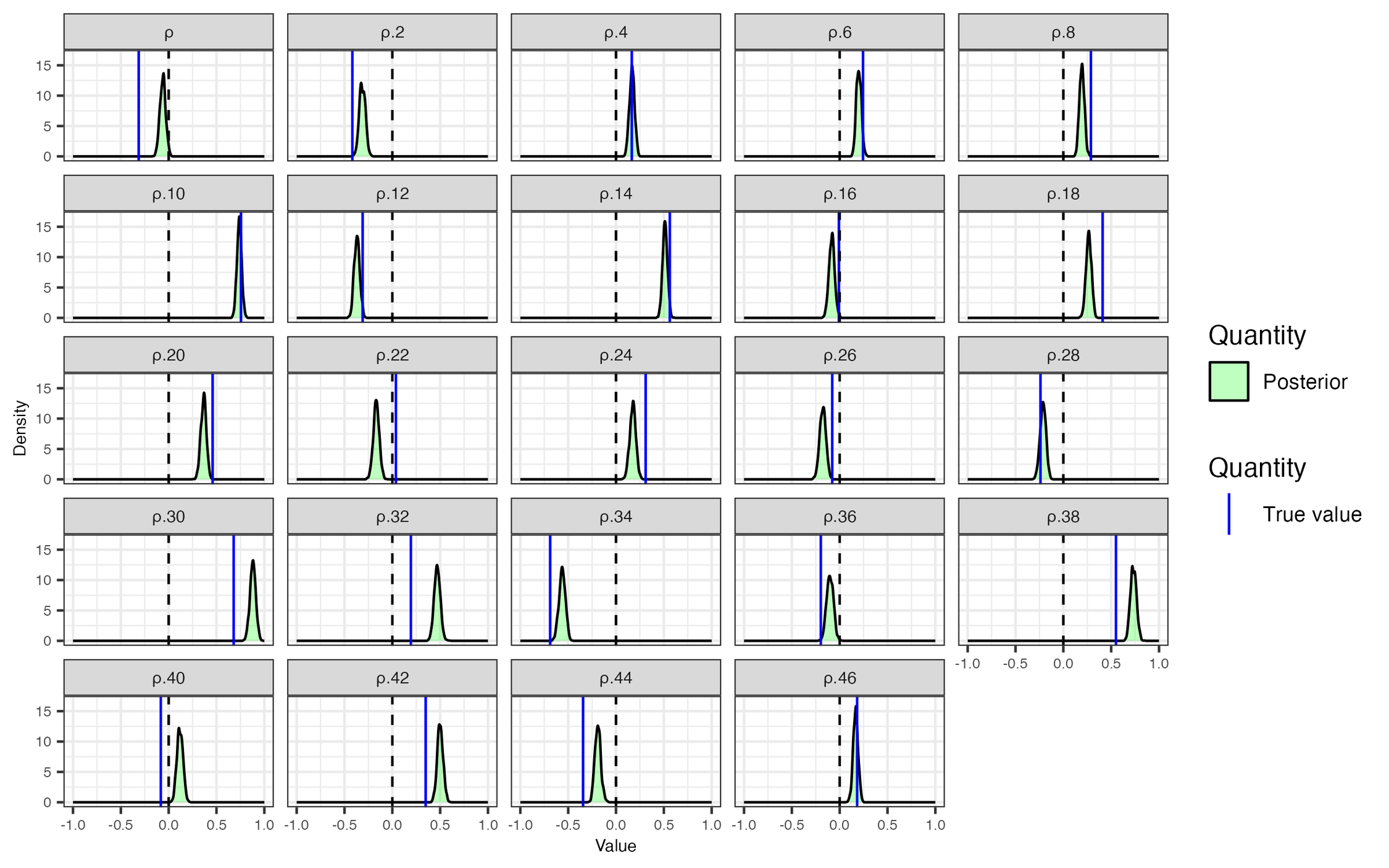

Supplement: S26 Fig — The data underlying this figure can be found at https://doi.org/10.5281/zenodo.12795911. (TIF) [file pbio.3002864.s026.tif]

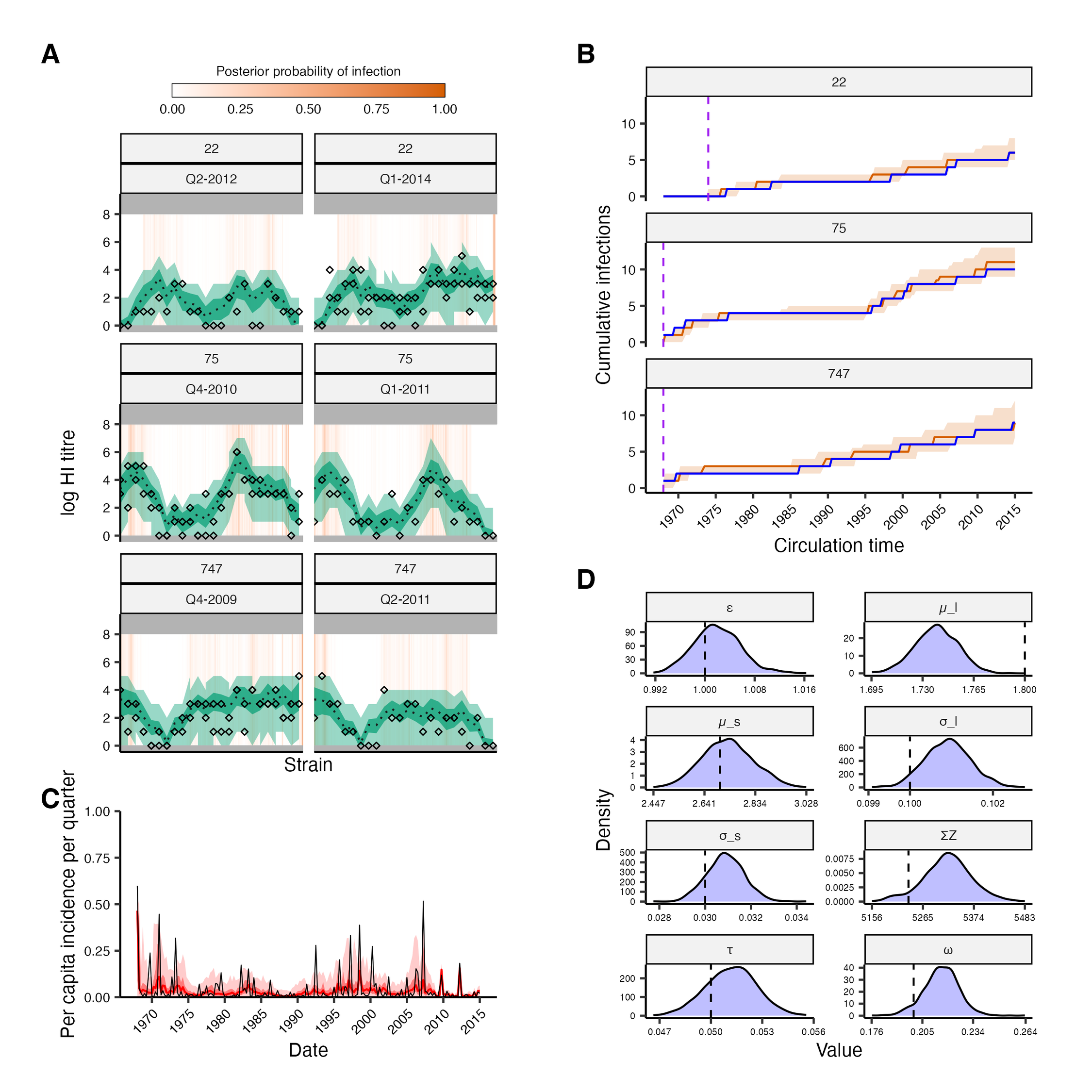

Supplement: S27 Fig — Results shown are from fitting the full model to simulated infection histories and antibody titres with known parameters. (A) Model-predicted titres compared to observed HI titres at each sampling time for three individuals. (B) Posterior median and 95% credible intervals (CrI) for the cumulative number of infections over time from birth (orange). Blue solid line shows the true, known cumulative number of infections. Purple dashed line shows the time of birth. (C) Posterior estimated per-capita per-3-month attack rates. Red line and shaded region shows posterior median and 95% CrI. Grey line shows the true values used for the simulation. (D) Shaded regions show posterior distributions of estimated antibody kinetics parameters. Dashed lines show the true value used for simulation. Note the x-axis range is small relative to the prior ranges in S5 Table. The data underlying this figure can be found at https://doi.org/10.5281/zenodo.12795911. (TIF) [file pbio.3002864.s027.tif]

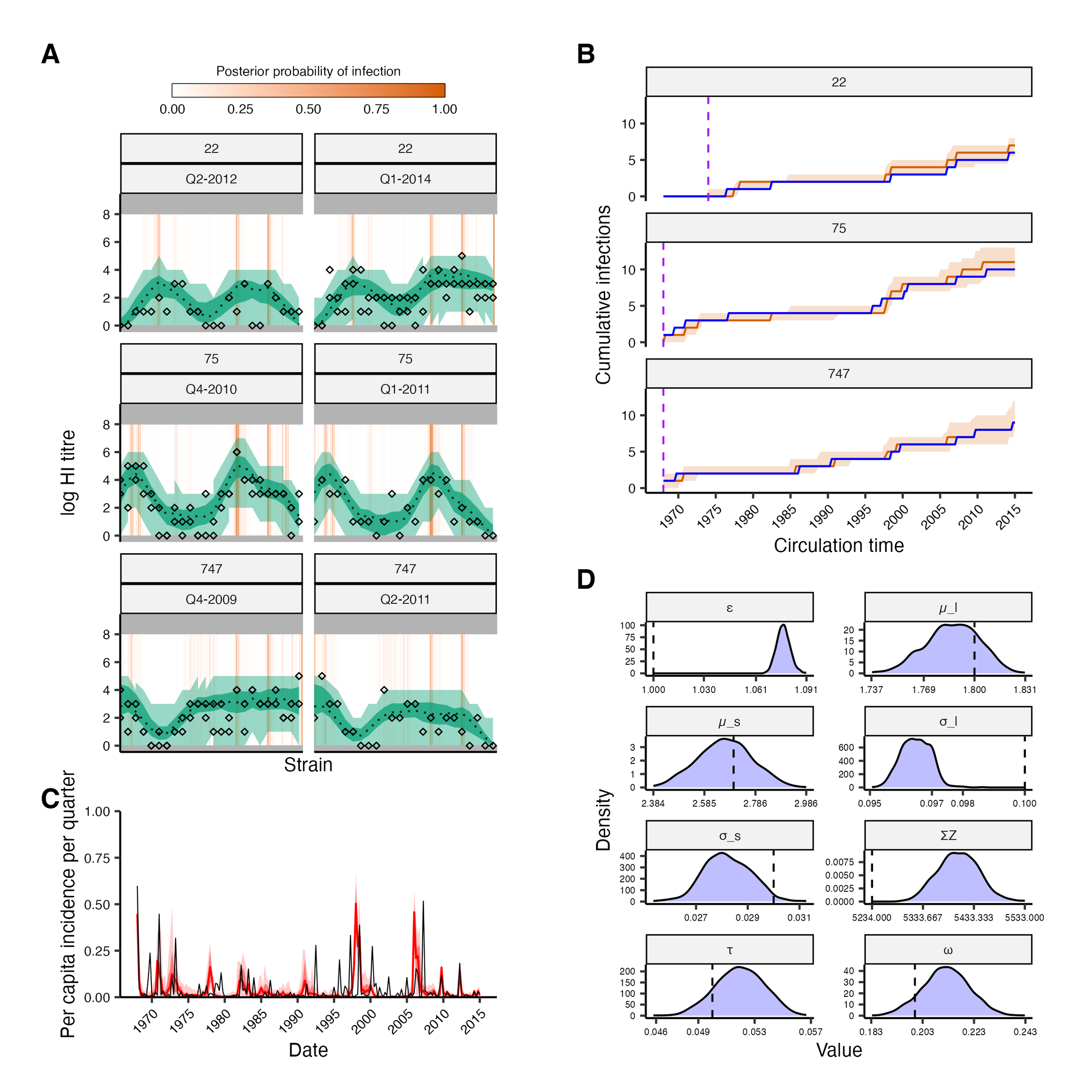

Supplement: S28 Fig — Results shown are from fitting the full model to simulated infection histories and antibody titres with known parameters where strain-specific measurement offsets are used in the simulation, but ignored in the fitted model. (A) Model-predicted titres compared to observed HI titres at each sampling time for 3 individuals. (B) Posterior median and 95% credible intervals (CrI) for the cumulative number of infections over time from birth (orange). Blue solid line shows the true, known cumulative number of infections. Purple dashed line shows the time of birth. (C) Posterior estimated per-capita per-3-month attack rates. Red line and shaded region shows posterior median and 95% CrI. Grey line shows the true values used for the simulation. (D) Shaded regions show posterior distributions of estimated antibody kinetics parameters. Dashed lines show the true value used for simulation. Note the x-axis range is small relative to the prior ranges in S5 Table. The data underlying this figure can be found at https://doi.org/10.5281/zenodo.12795911. (TIF) [file pbio.3002864.s028.tif]

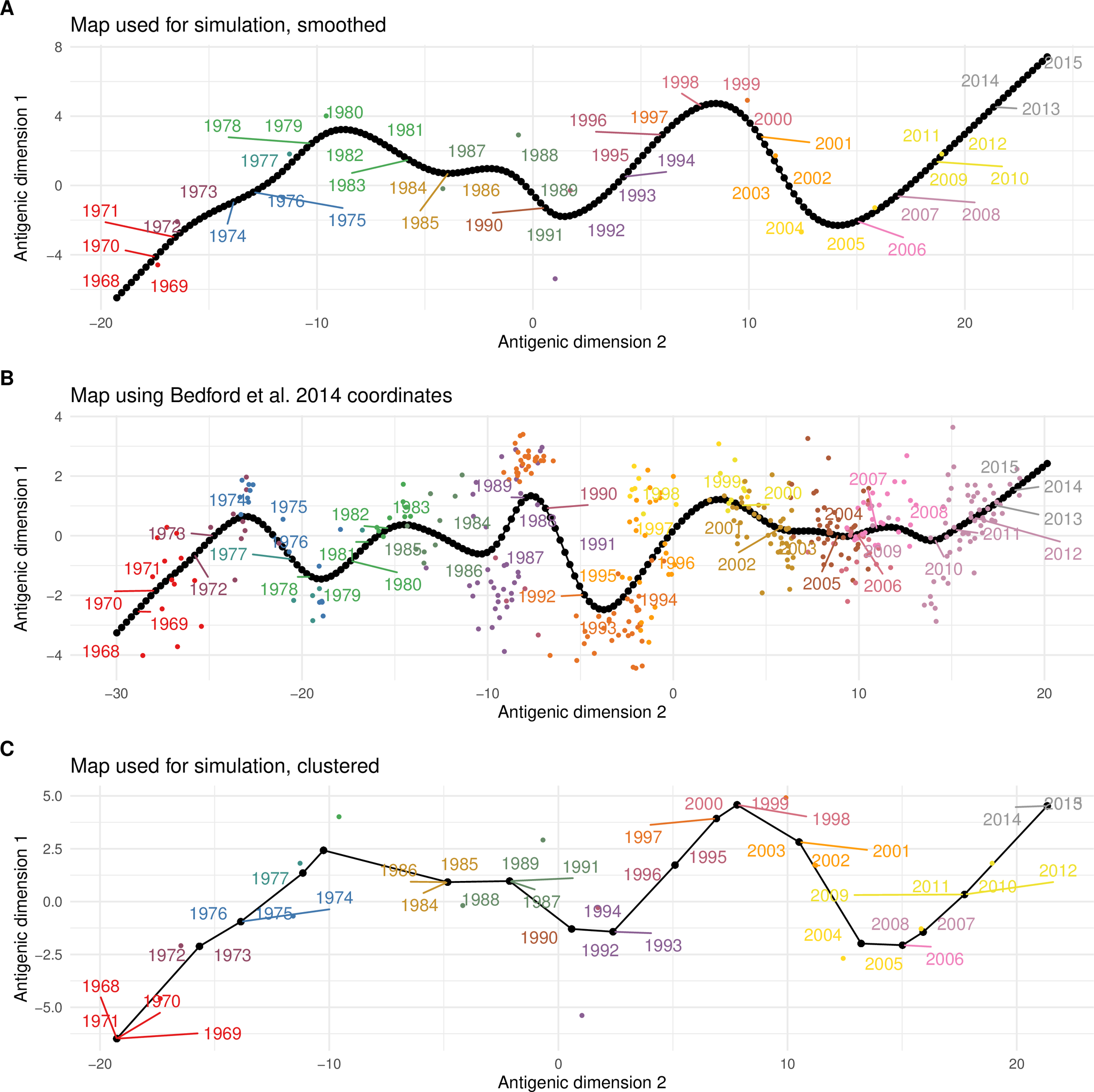

Supplement: S29 Fig — Antigenic maps used for the simulation (A) compared to the map produced using data from Bedford and colleagues (B), and the map which assumes a punctuated path through antigenic space (C). Axes show arbitrary antigenic dimensions. Coloured points show the position of individual strains—all labels of the same colour correspond to the same antigenic cluster. Black line shows the fitted antigenic summary path for each map. Black dots show the antigenic position of the strain corresponding to each time period; (A) and (B) assume continuous evolution through antigenic space, whereas (C) assumes that the same position is used for all strains within a cluster. The data underlying this figure can be found at https://doi.org/10.5281/zenodo.12795911. (TIF) [file pbio.3002864.s029.tif]

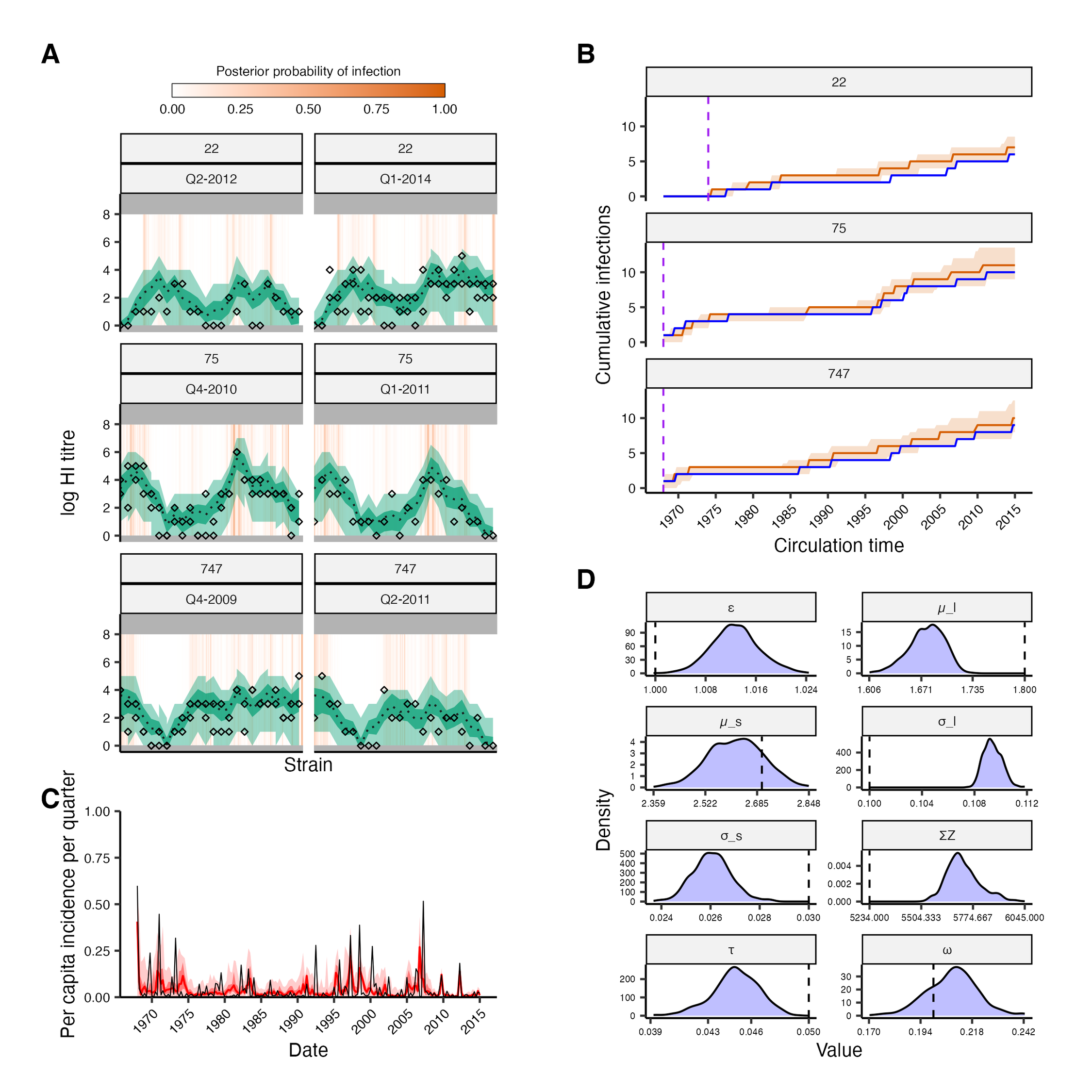

Supplement: S30 Fig — Results shown are from fitting the full model to simulated infection histories and antibody titres with known parameters. (A) Model-predicted titres compared to observed HI titres at each sampling time for 3 individuals. (B) Posterior median and 95% credible intervals (CrI) for the cumulative number of infections over time from birth (orange). Blue solid line shows the true, known cumulative number of infections. Purple dashed line shows the time of birth. (C) Posterior estimated per-capita per-3-month attack rates. Red line and shaded region shows posterior median and 95% CrI. Grey line shows the true values used for the simulation. (D) Shaded regions show posterior distributions of estimated antibody kinetics parameters. Dashed lines show the true value used for simulation. Note the x-axis range is small relative to the prior ranges in S5 Table. The data underlying this figure can be found at https://doi.org/10.5281/zenodo.12795911. (TIF) [file pbio.3002864.s030.tif]

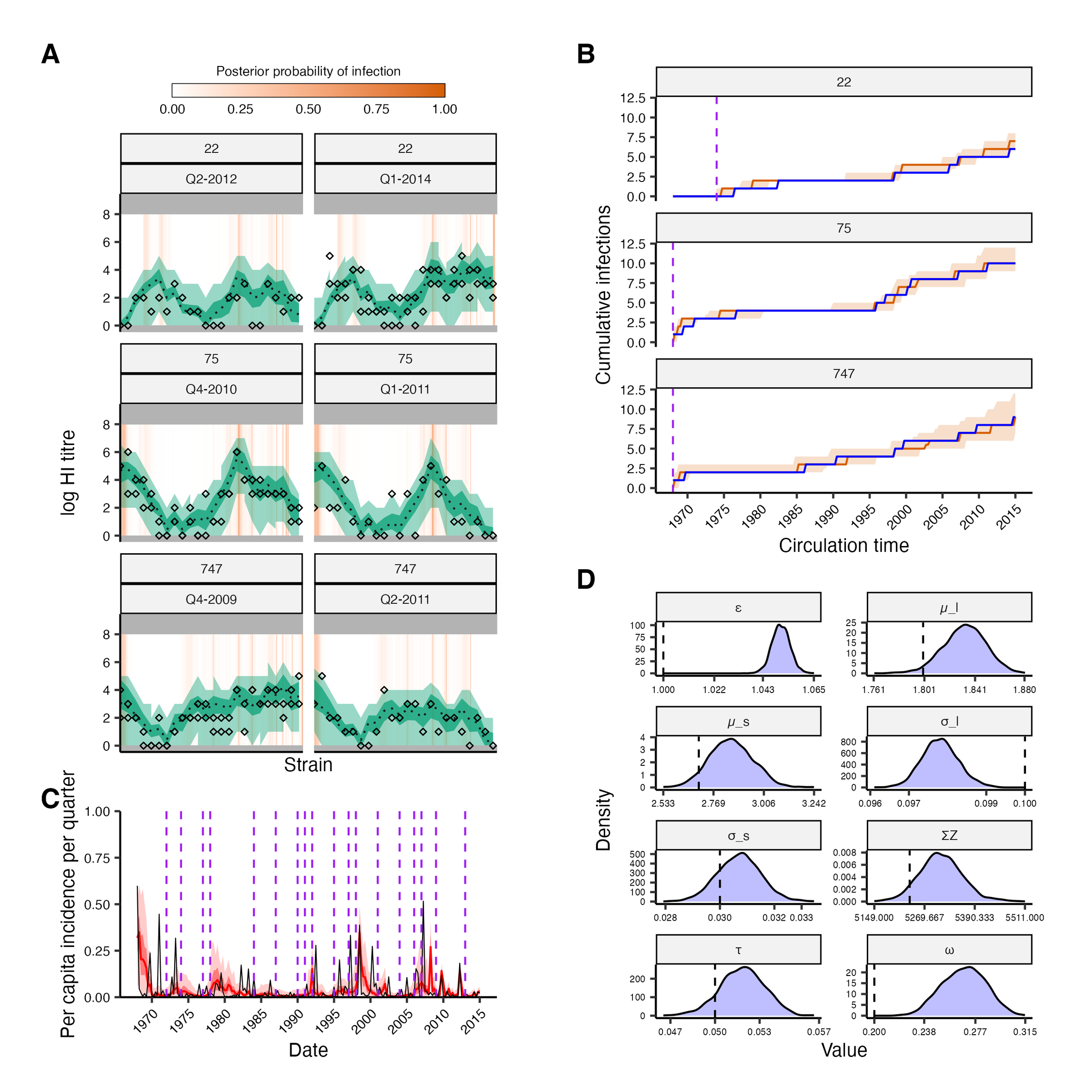

Supplement: S31 Fig — (A) Model-predicted titres compared to observed HI titres at each sampling time for 3 individuals. (B) Posterior median and 95% credible intervals (CrI) for the cumulative number of infections over time from birth (orange). Blue solid line shows the true, known cumulative number of infections. Purple dashed line shows the time of birth. (C) Posterior estimated per-capita per-3-month attack rates. Red line and shaded region shows posterior median and 95% CrI. Grey line shows the true values used for the simulation. Purple dashed lines show cluster transitions. (D) Shaded regions show posterior distributions of estimated antibody kinetics parameters. Dashed lines show the true value used for simulation. Note the x-axis range is small relative to the prior ranges in S5 Table. The data underlying this figure can be found at https://doi.org/10.5281/zenodo.12795911. (TIF) [file pbio.3002864.s031.tif]
