# Supplementary material for: Reconstructed influenza A/H3N2 infection histories reveal variation in incidence and antibody dynamics over the life course
Source: PLoS Biol. 2024 Nov 7;22(11):e3002864. doi: 10.1371/journal.pbio.3002864 (PMC11542844; doi:10.1371/journal.pbio.3002864)
Supplement: S20 Fig — Each subplot shows antibody levels measured against each of the 20 H3N2 strains. The x-axis shows the isolation year of the measured strain. The areas are shaded by sample number, showing titre measurements from the first (blue) and second (red) samples. Grey rectangles mark strains which circulated before that individual was born. The vertical coloured lines show the timing of the serum samples relative to the strain isolation times. Plots where the red region extends above the blue region reflect antibody boosting between the first and second serum sample. Where multiple titres were measured against the same strain from the same serum sample, we plotted the mean of the log titres. The data underlying this figure can be found at https://doi.org/10.5281/zenodo.12795911. (PDF) [file pbio.3002864.s020.pdf]

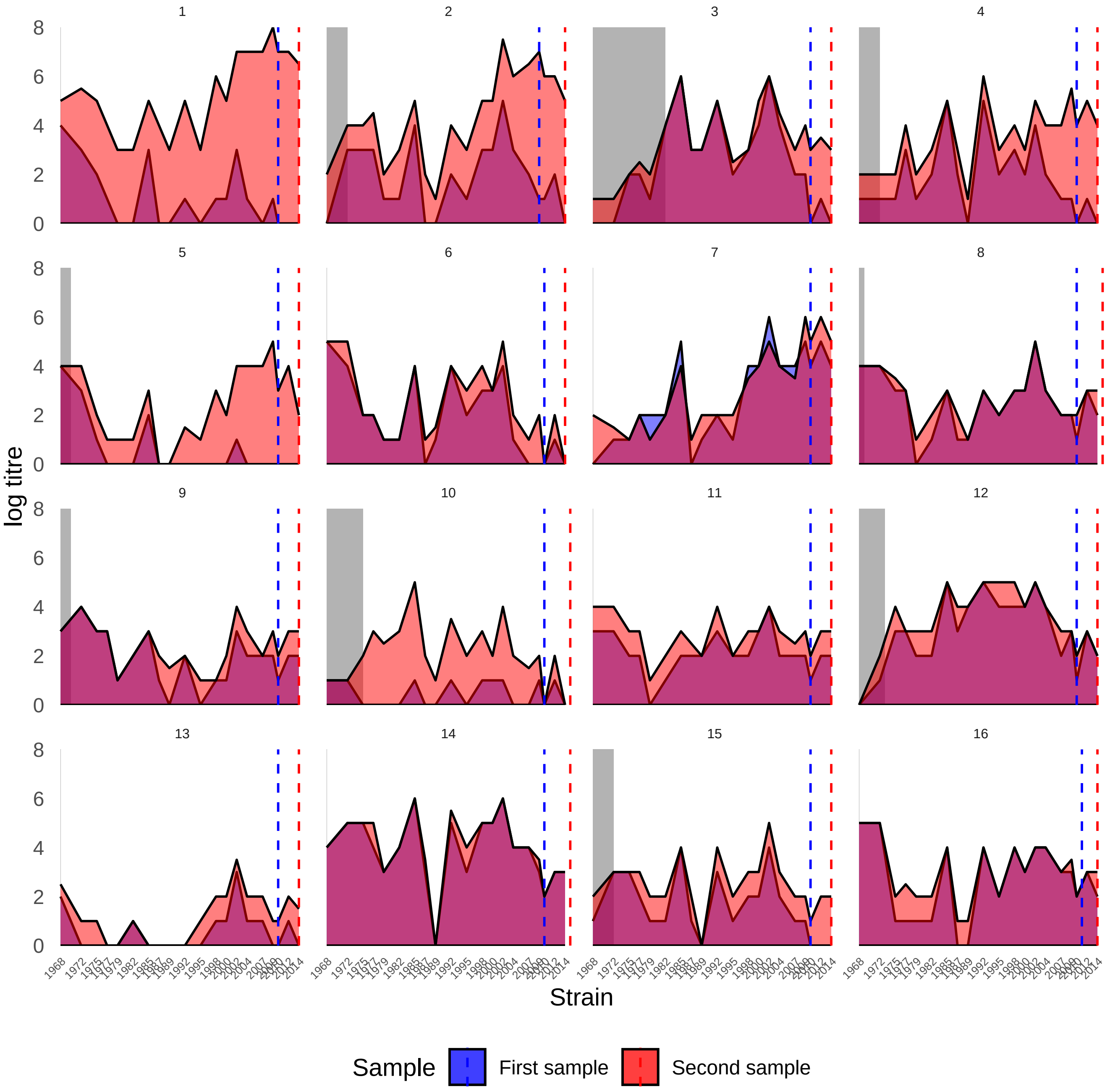

1
2
3
4
5
6
7
8
9
10
11
12
13
14
15
16
log titre
0
2
4
6
8
1968
1972
1975
1977
1979
1982
1985
1987
1989
1992
1995
1998
2000
2002
2004
2007
2009
2010
2012
2014
Strain
Sample
First sample
Second sample

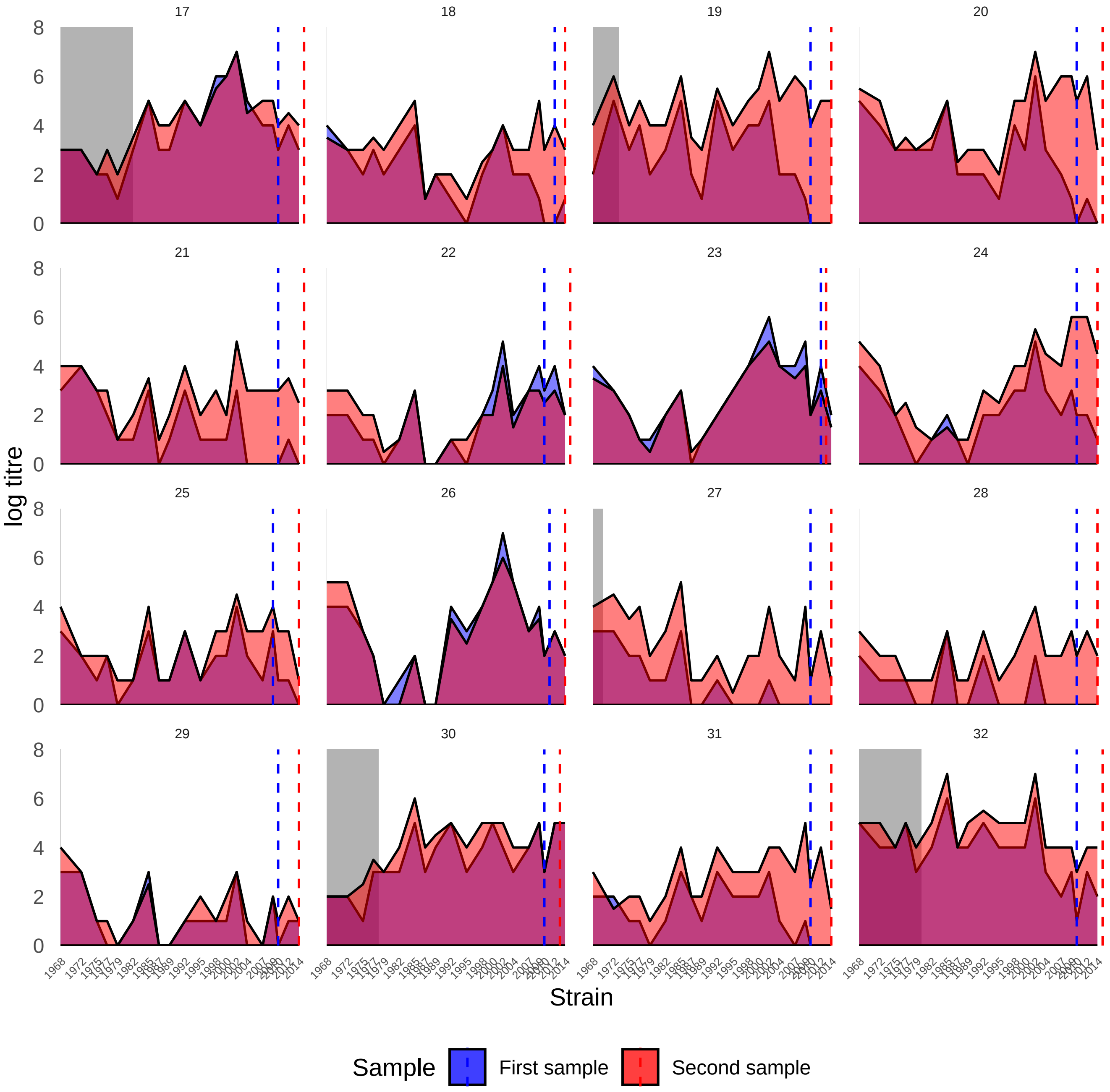

17
18
19
20
21
22
23
24
25
26
27
28
29
30
31
32
log titre
Strain
Sample
First sample
Second sample

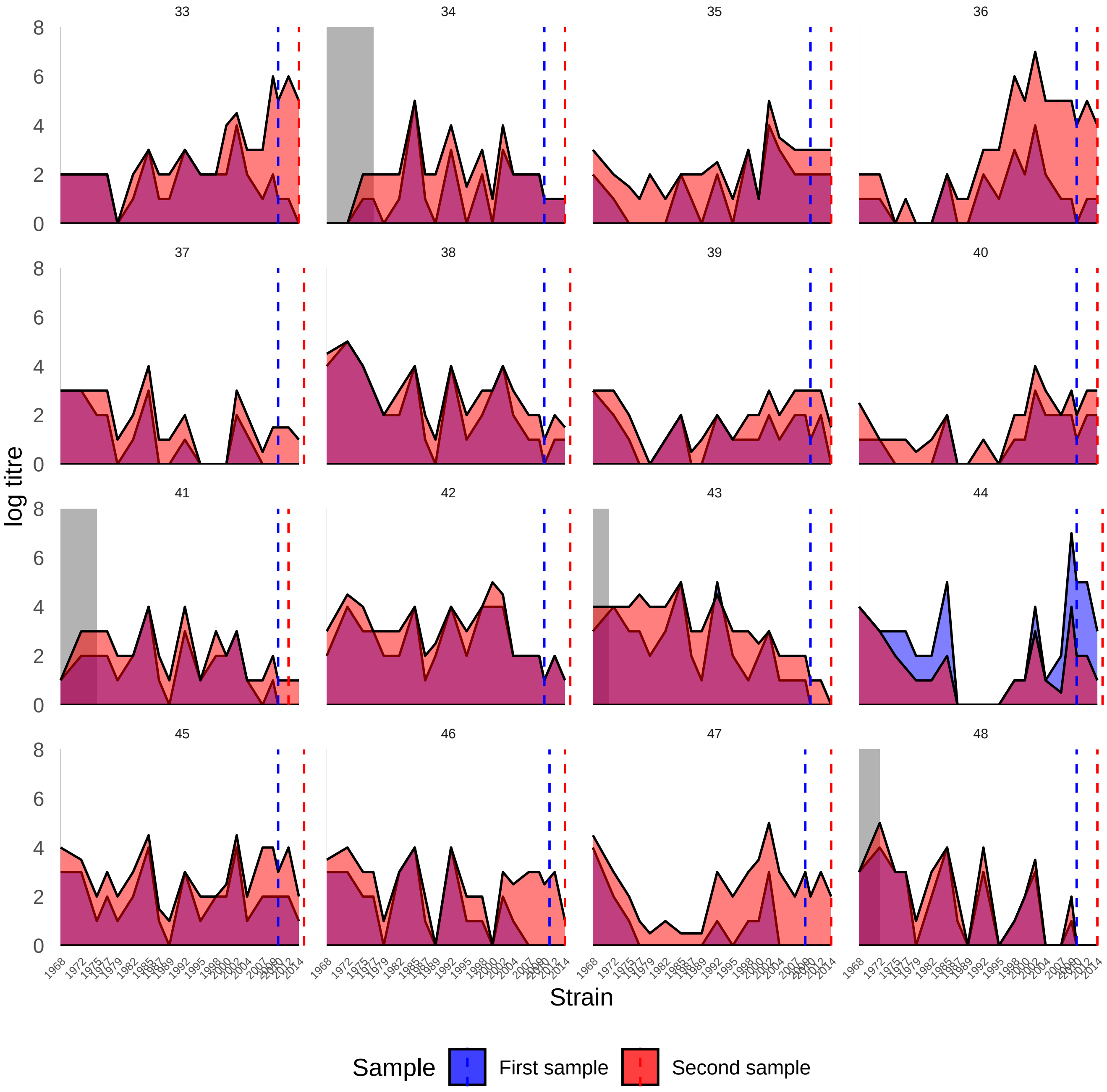

33
34
35
36
37
38
39
40
41
42
43
44
45
46
47
48
log titre
Strain
1968
1972
1975
1977
1979
1982
1985
1987
1989
1992
1995
1998
2000
2002
2004
2007
2009
2010
2012
2014
Sample
First sample
Second sample

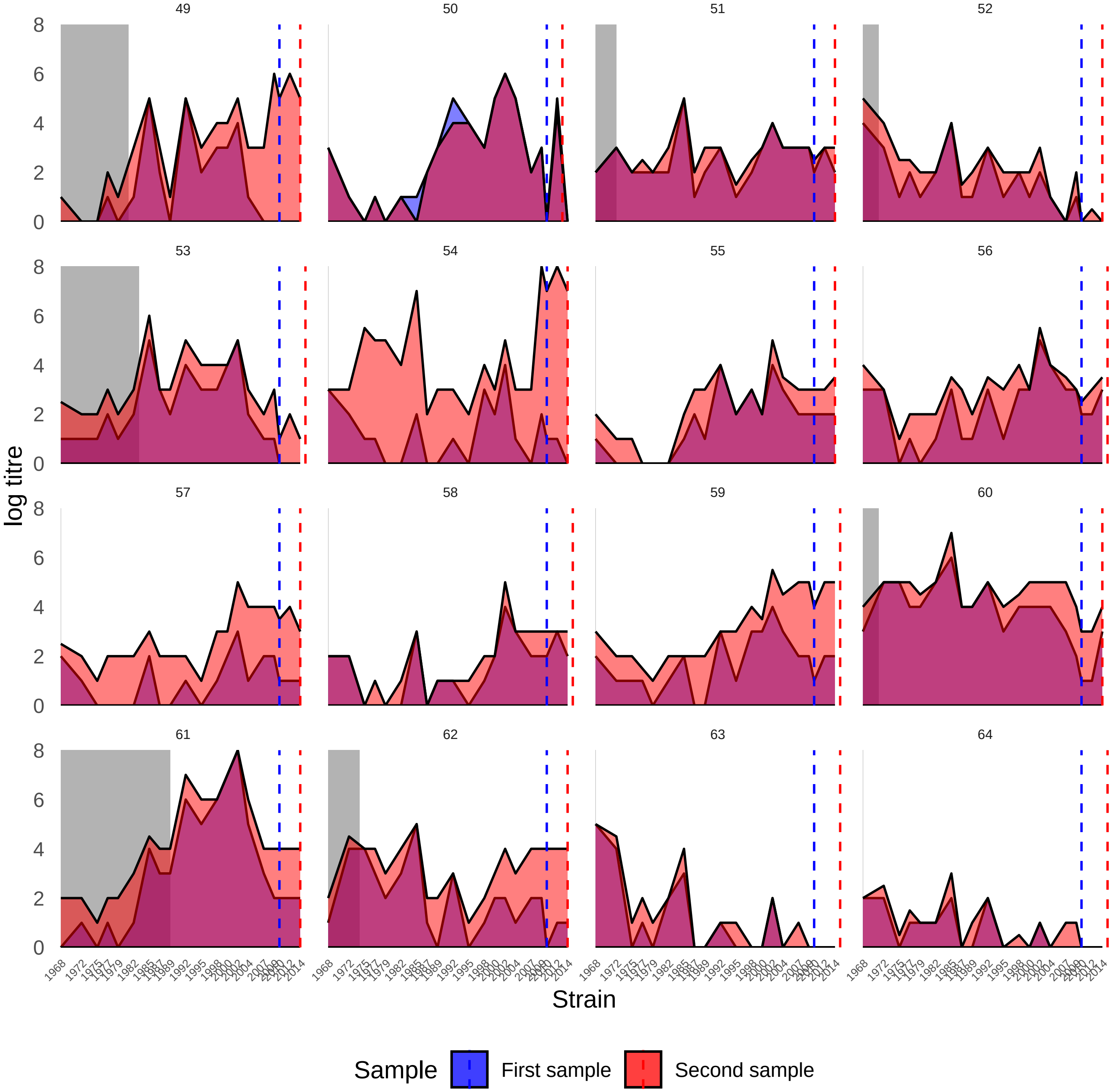

49
50
51
52
53
54
55
56
57
58
59
60
61
62
63
64
log titre
0
2
4
6
8
1968
1972
1975
1977
1979
1982
1985
1987
1989
1992
1995
1998
2000
2002
2004
2007
2009
2010
2012
2014
Strain
Sample
First sample
Second sample

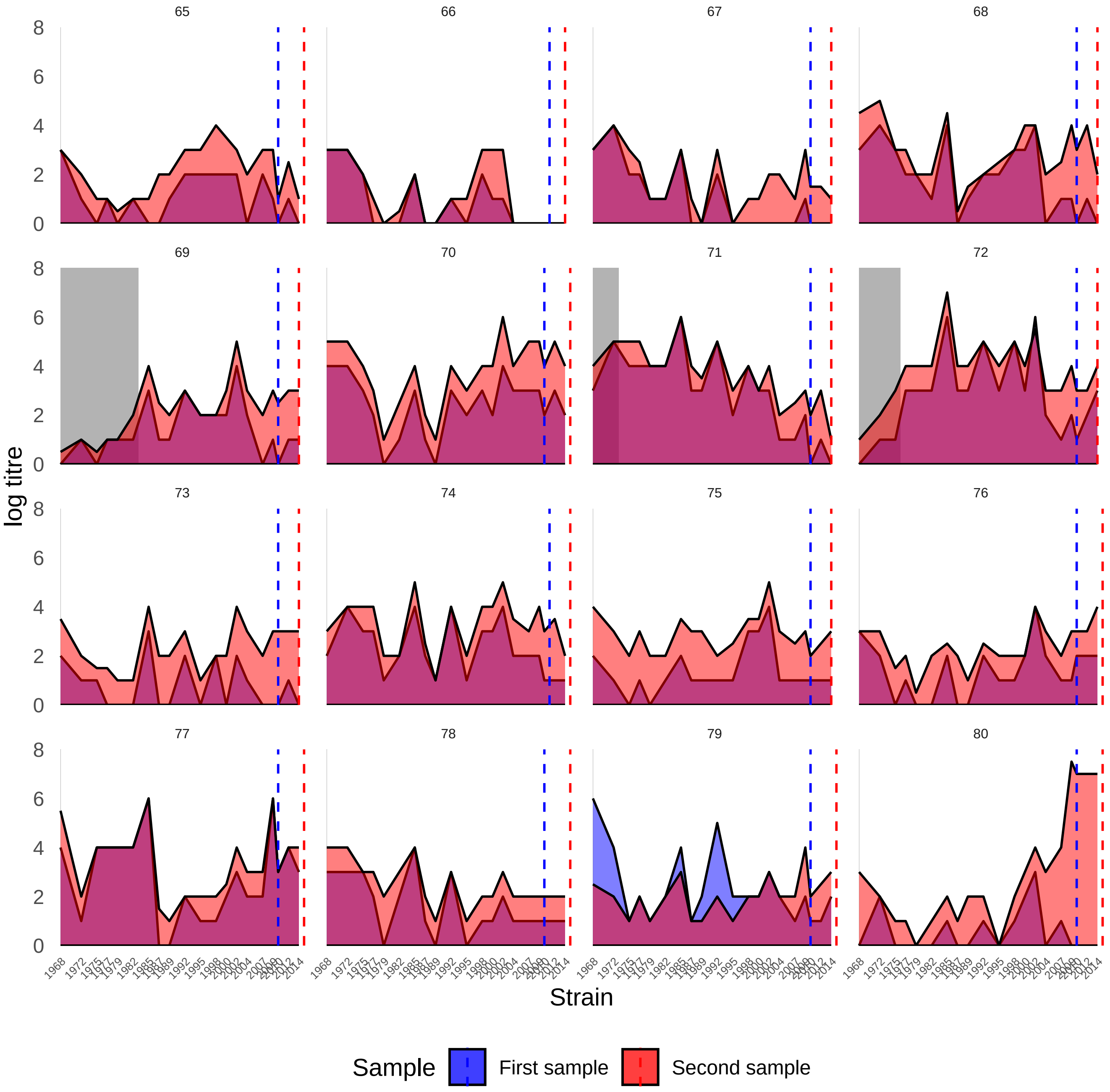
65
66
67
68
69
70
71
72
73
74
75
76
77
78
79
80
log titre
Strain
0
2
4
6
8
1968
1972
1975
1977
1979
1982
1985
1987
1989
1992
1995
1998
2000
2002
2004
2007
2009
2010
2012
2014
Sample
First sample
Second sample

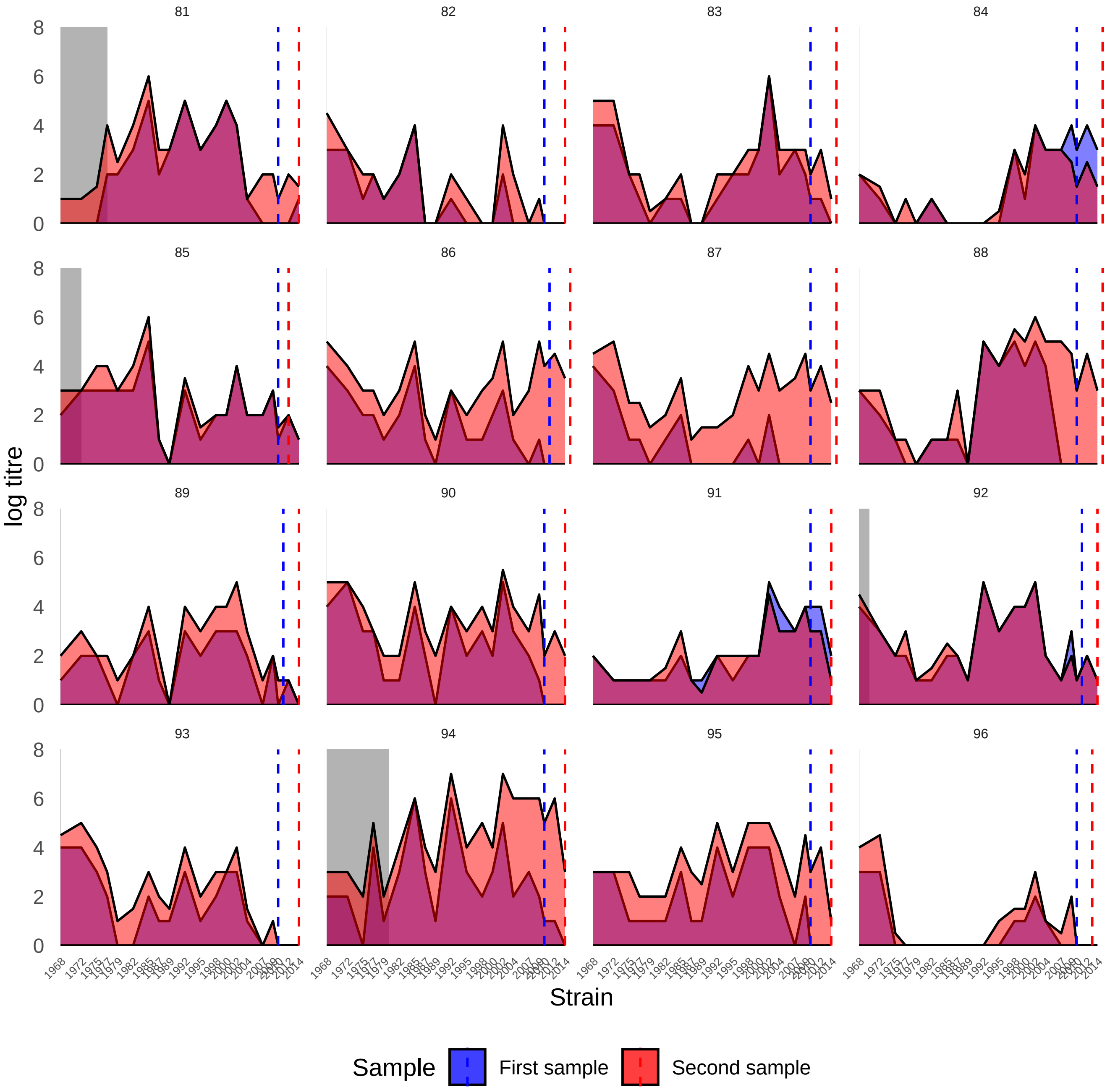

81
82
83
84
85
86
87
88
89
90
91
92
93
94
95
96
log titre
0
2
4
6
8
1968
1972
1975
1977
1979
1982
1985
1987
1989
1992
1995
1998
2000
2002
2004
2007
2009
2010
2012
2014
Strain
Sample
First sample
Second sample

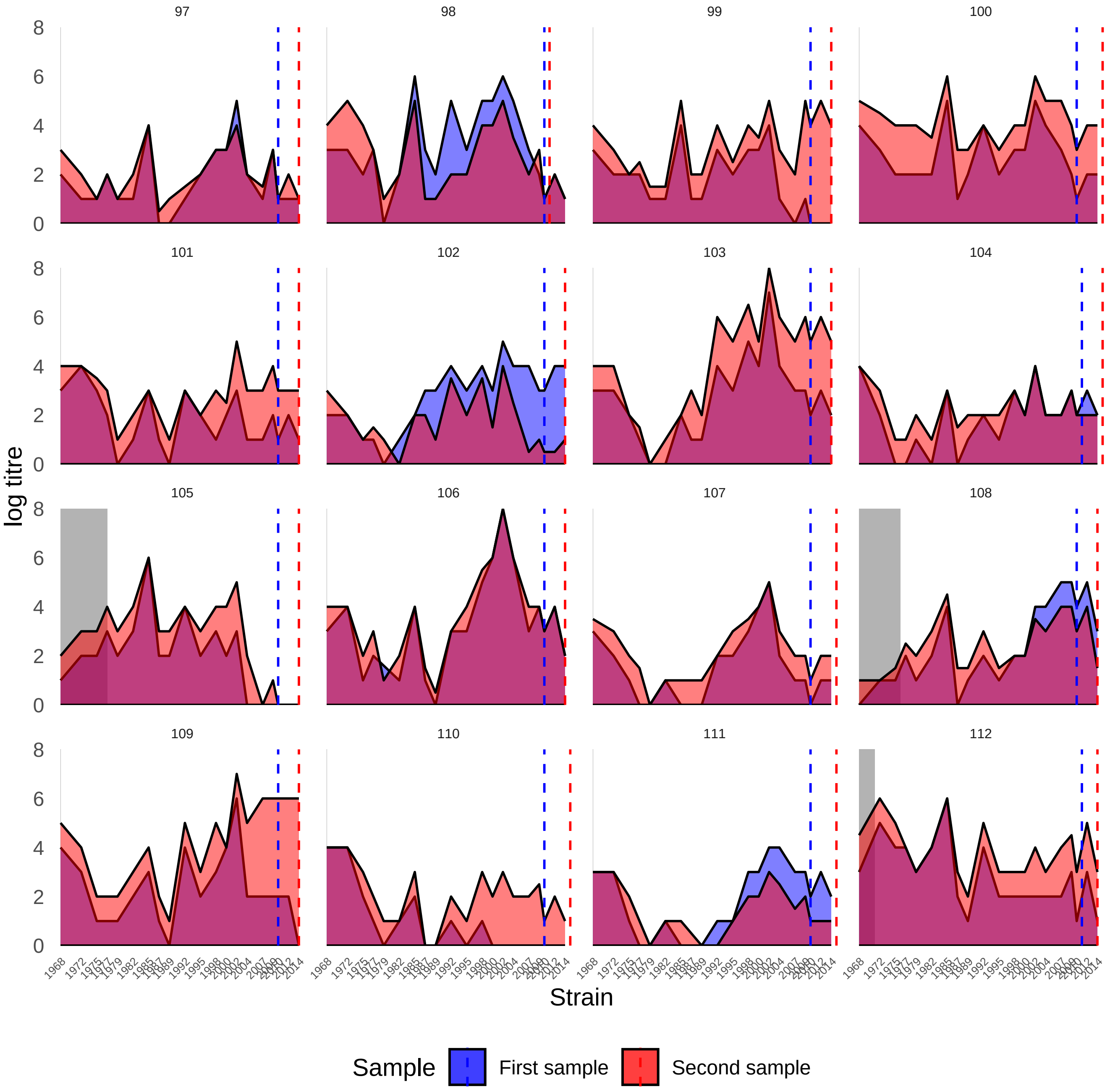
97
98
99
100
101
102
103
104
105
106
107
108
109
110
111
112
log titre
0
2
4
6
8
1968
1972
1975
1977
1979
1982
1985
1987
1989
1992
1995
1998
2000
2002
2004
2007
2009
2010
2012
2014
Strain
Sample
First sample
Second sample

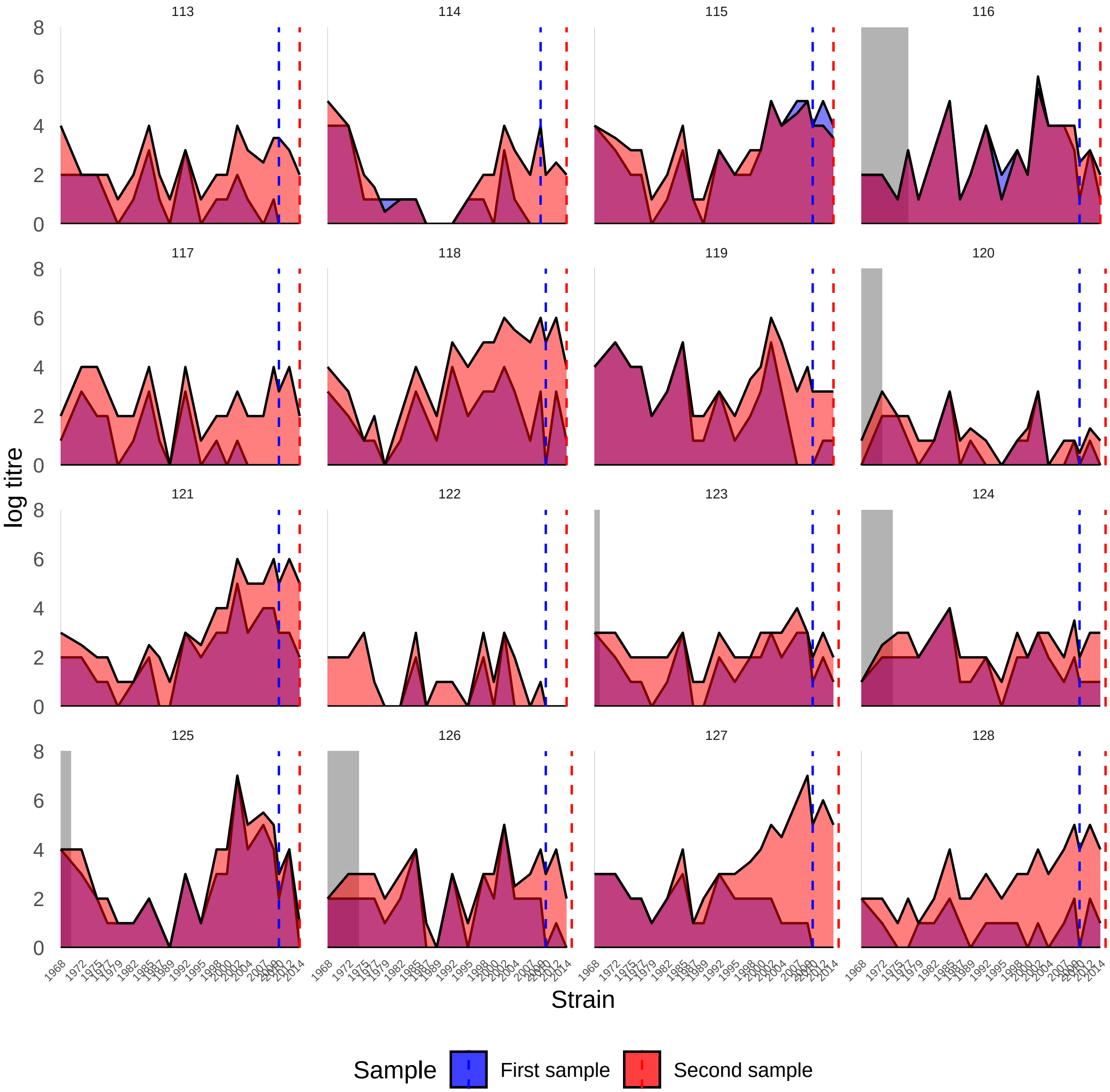
113
114
115
116
117
118
119
120
121
122
123
124
125
126
127
128
log titre
Strain
Sample
First sample
Second sample

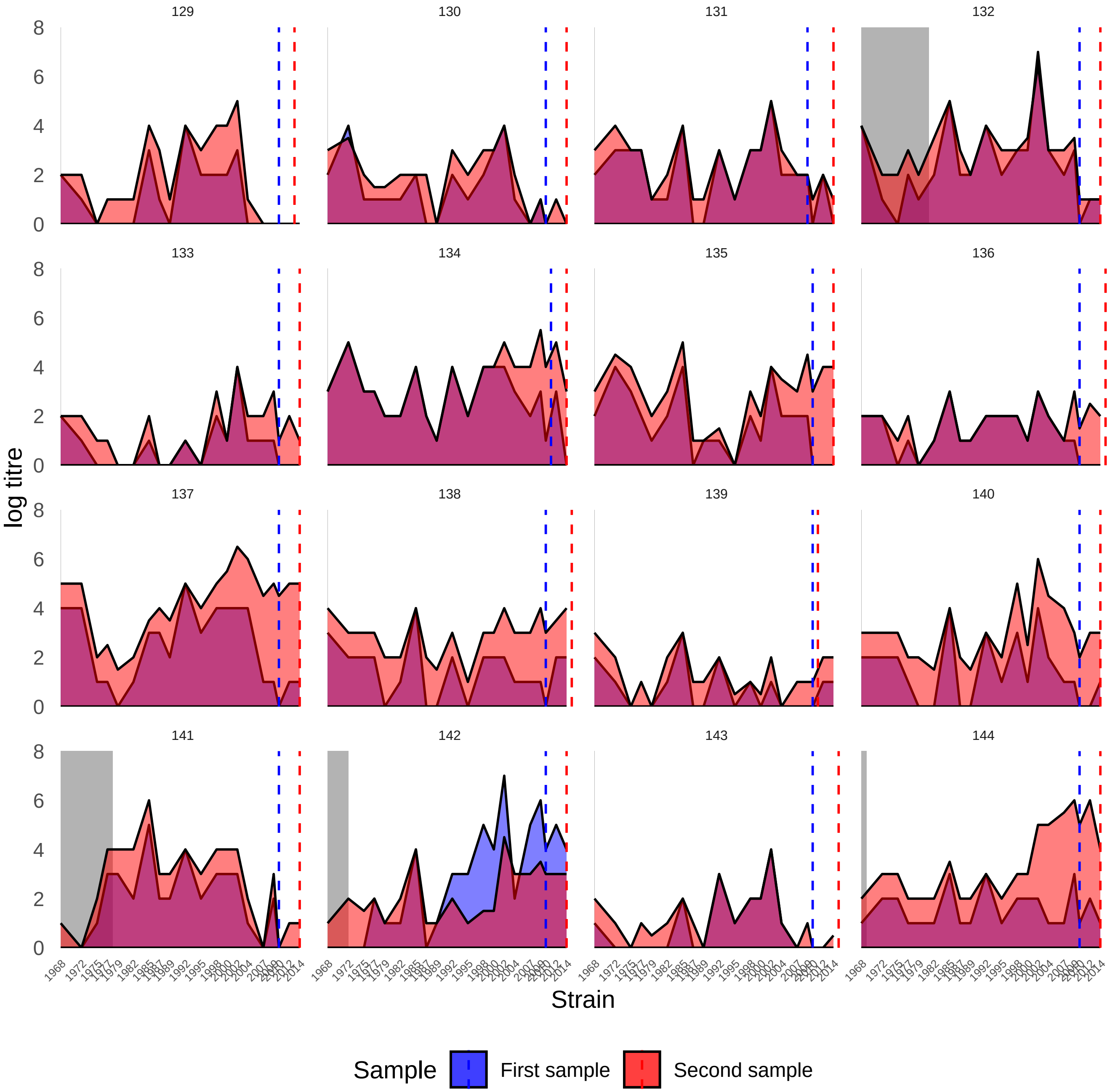

129
130
131
132
133
134
135
136
137
138
139
140
141
142
143
144
log titre
Strain
Sample
First sample
Second sample

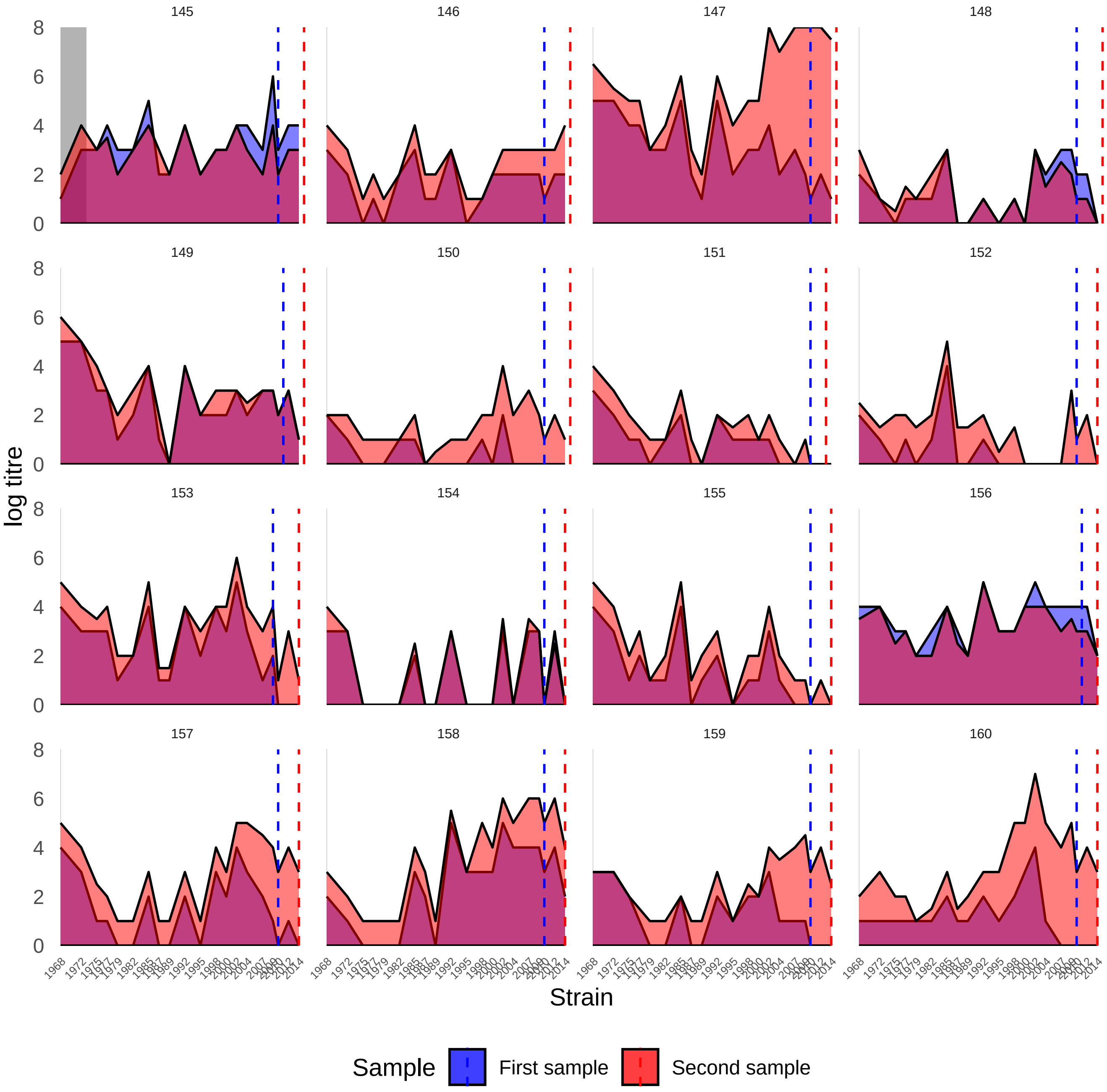
145
146
147
148
149
150
151
152
153
154
155
156
157
158
159
160
log titre
0
2
4
6
8
1968
1972
1975
1977
1979
1982
1985
1987
1989
1992
1995
1998
2000
2002
2004
2007
2009
2012
2014
Strain
Sample
First sample
Second sample

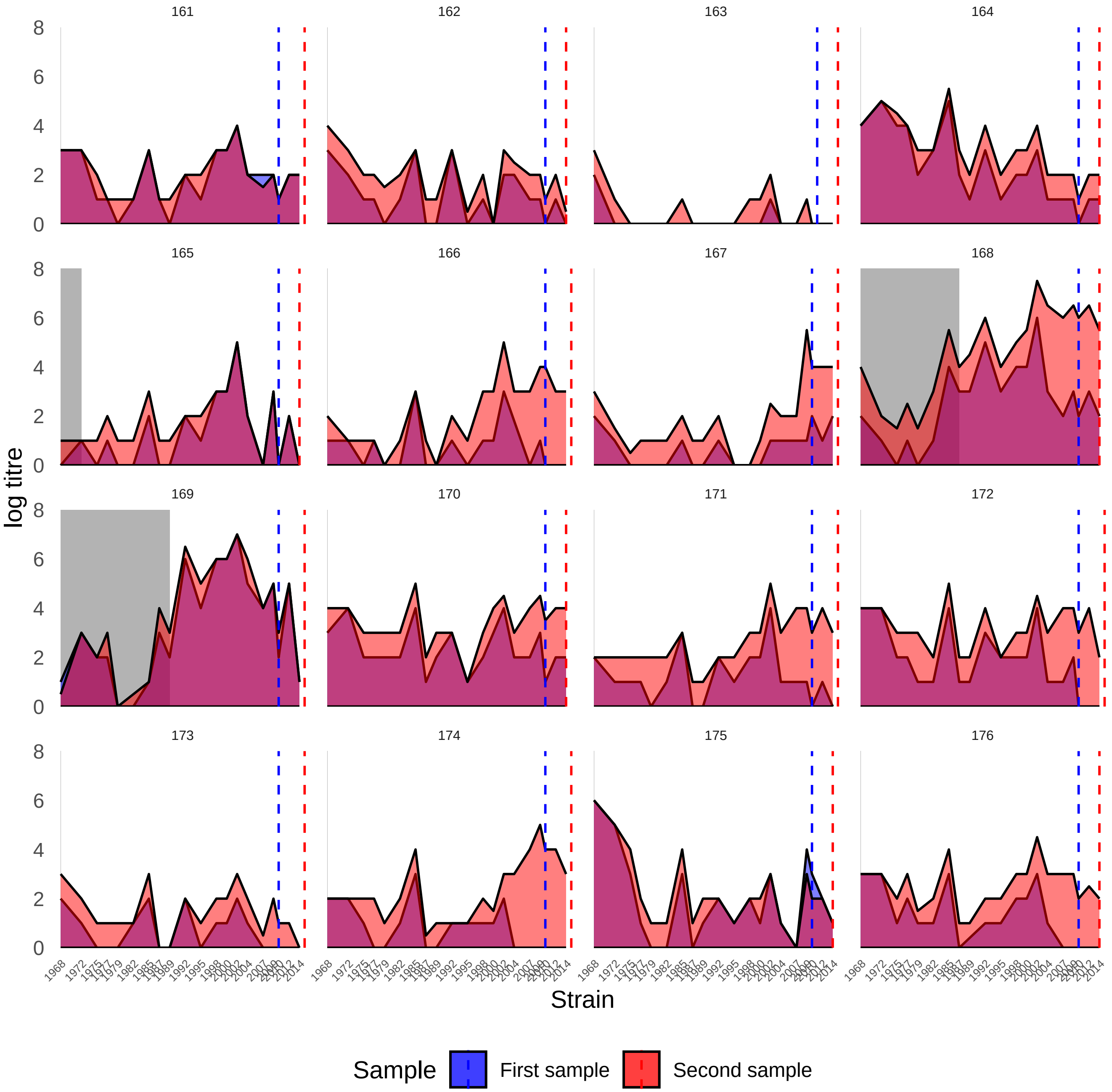
161
162
163
164
165
166
167
168
169
170
171
172
173
174
175
176
log titre
0
2
4
6
8
1968
1972
1975
1977
1979
1982
1985
1987
1989
1992
1995
1998
2000
2002
2004
2007
2009
2010
2012
2014
Strain
Sample
First sample
Second sample

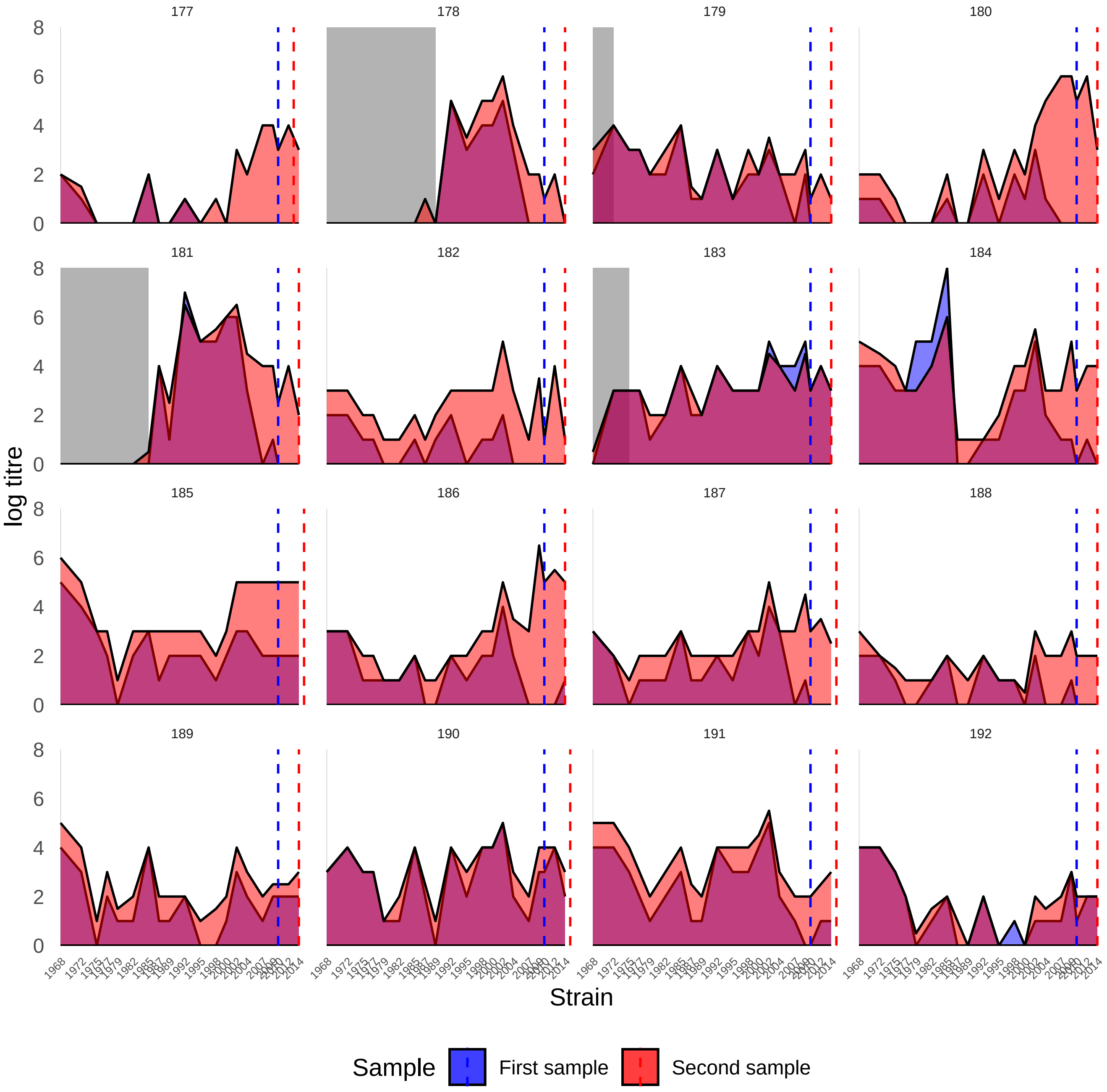

177
178
179
180
181
182
183
184
185
186
187
188
189
190
191
192
log titre
Strain
Sample
First sample
Second sample

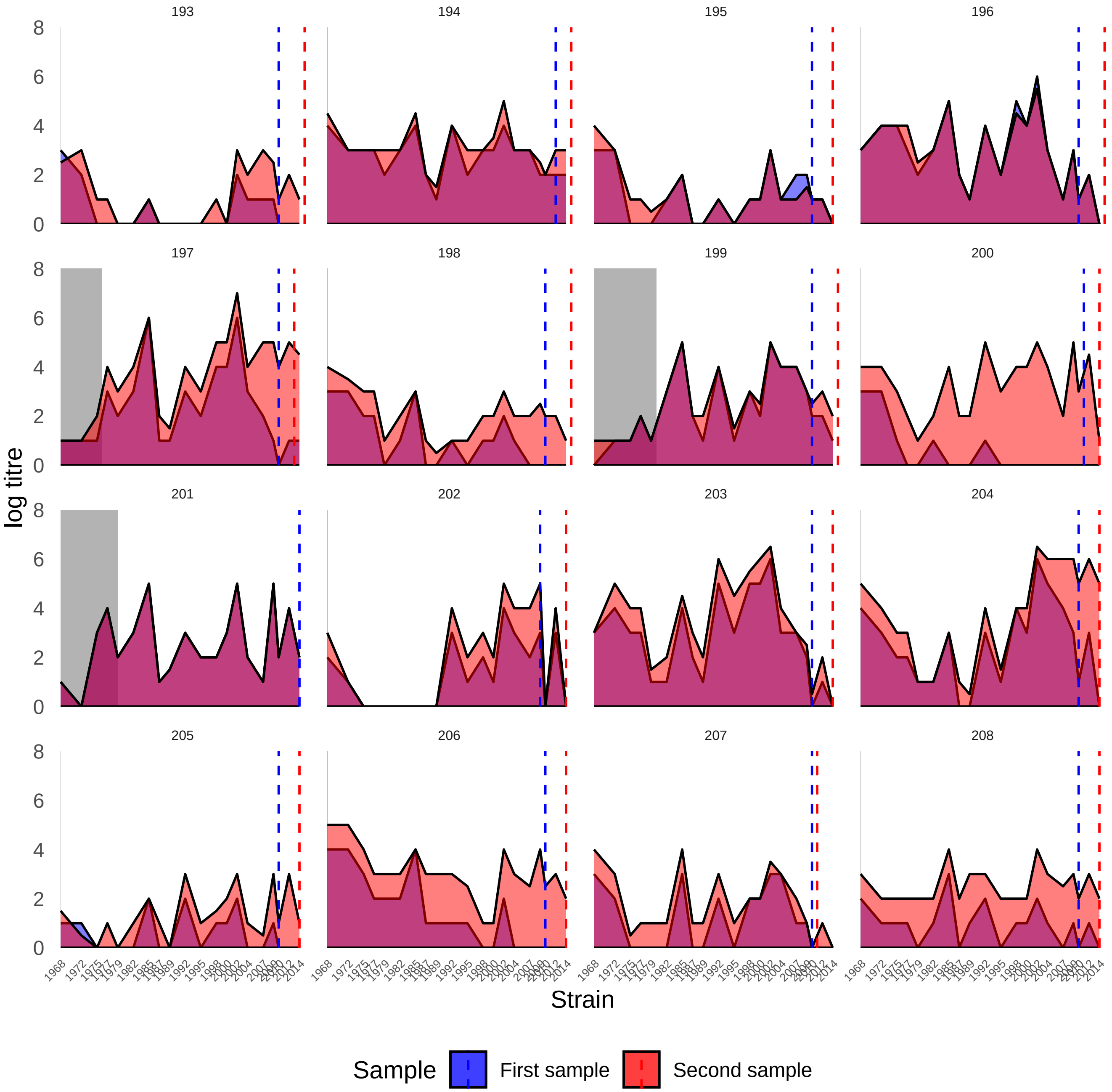

193
194
195
196
197
198
199
200
201
202
203
204
205
206
207
208
log titre
0
2
4
6
8
1968
1972
1975
1977
1979
1982
1985
1987
1989
1992
1995
1998
2000
2002
2004
2007
2009
2010
2012
2014
Strain
Sample
First sample
Second sample

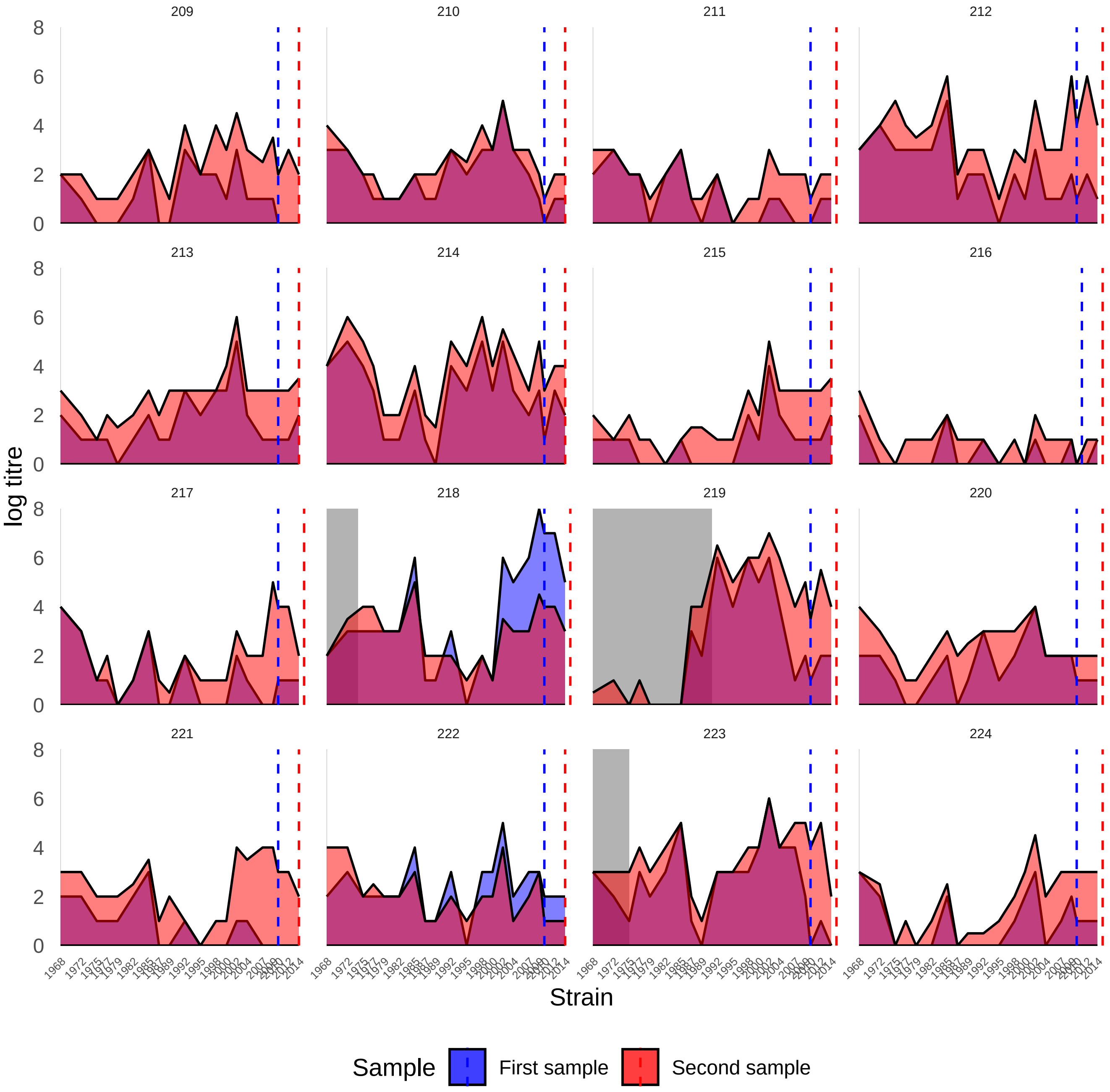
209
210
211
212
213
214
215
216
217
218
219
220
221
222
223
224
log titre
0
2
4
6
8
1968
1972
1975
1977
1979
1982
1985
1987
1989
1992
1995
1998
2000
2002
2004
2007
2009
2010
2012
2014
Strain
Sample
First sample
Second sample

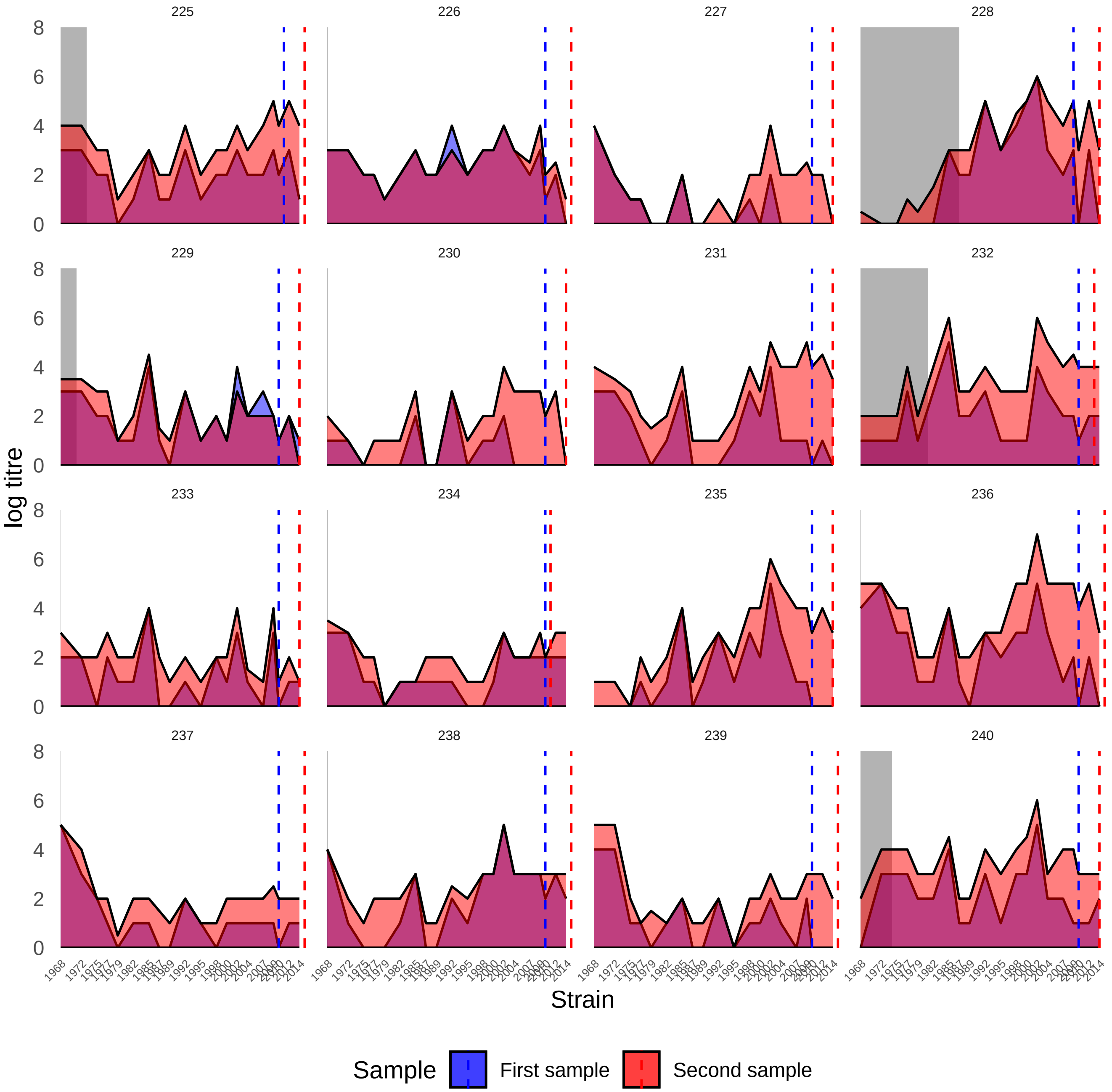

225
226
227
228
229
230
231
232
233
234
235
236
237
238
239
240
log titre
Strain
Sample
First sample
Second sample

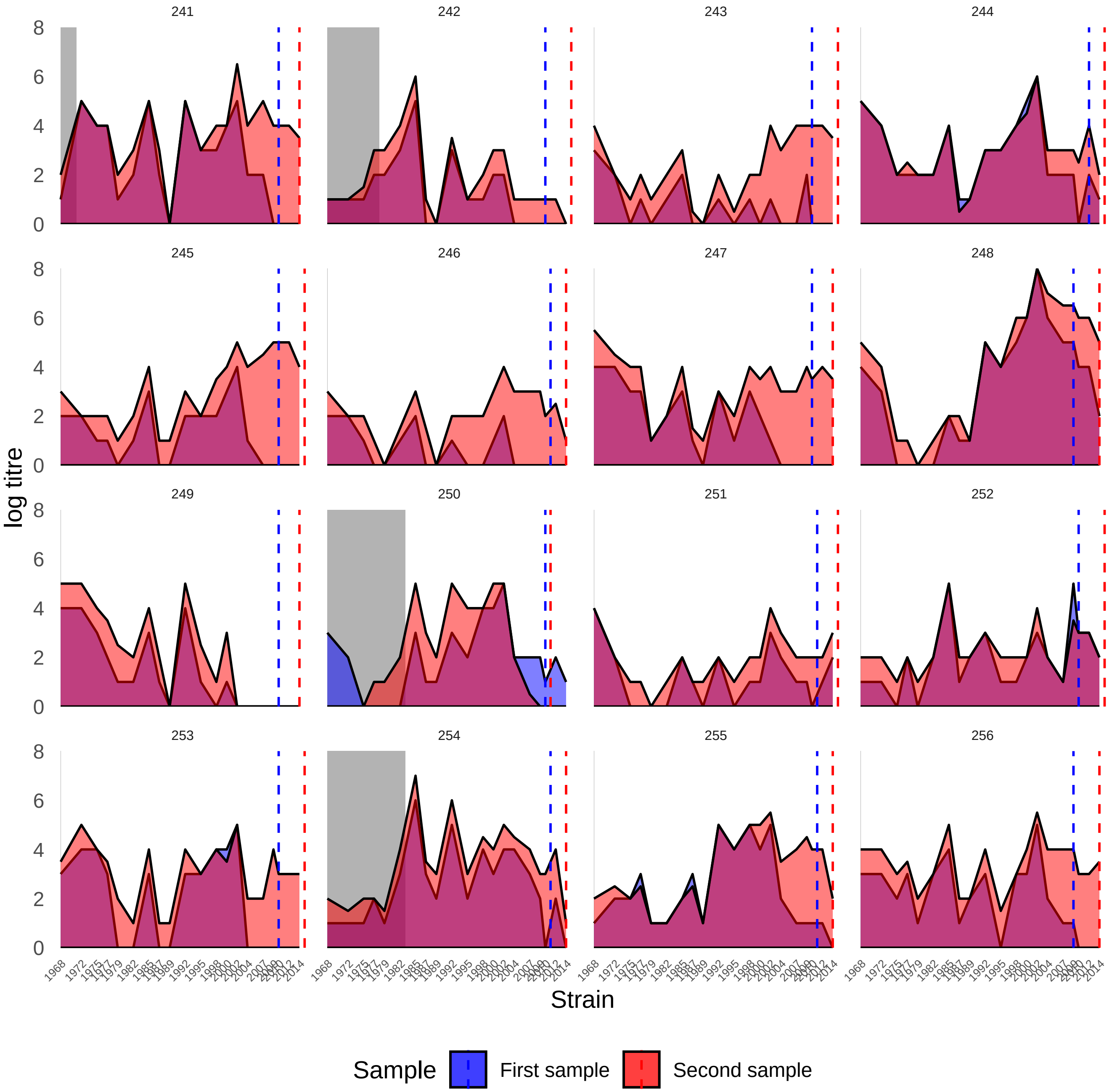

241
242
243
244
245
246
247
248
249
250
251
252
253
254
255
256
log titre
0
2
4
6
8
1968
1972
1975
1977
1979
1982
1985
1987
1989
1992
1995
1998
2000
2002
2004
2007
2009
2010
2012
2014
Strain
Sample
First sample
Second sample

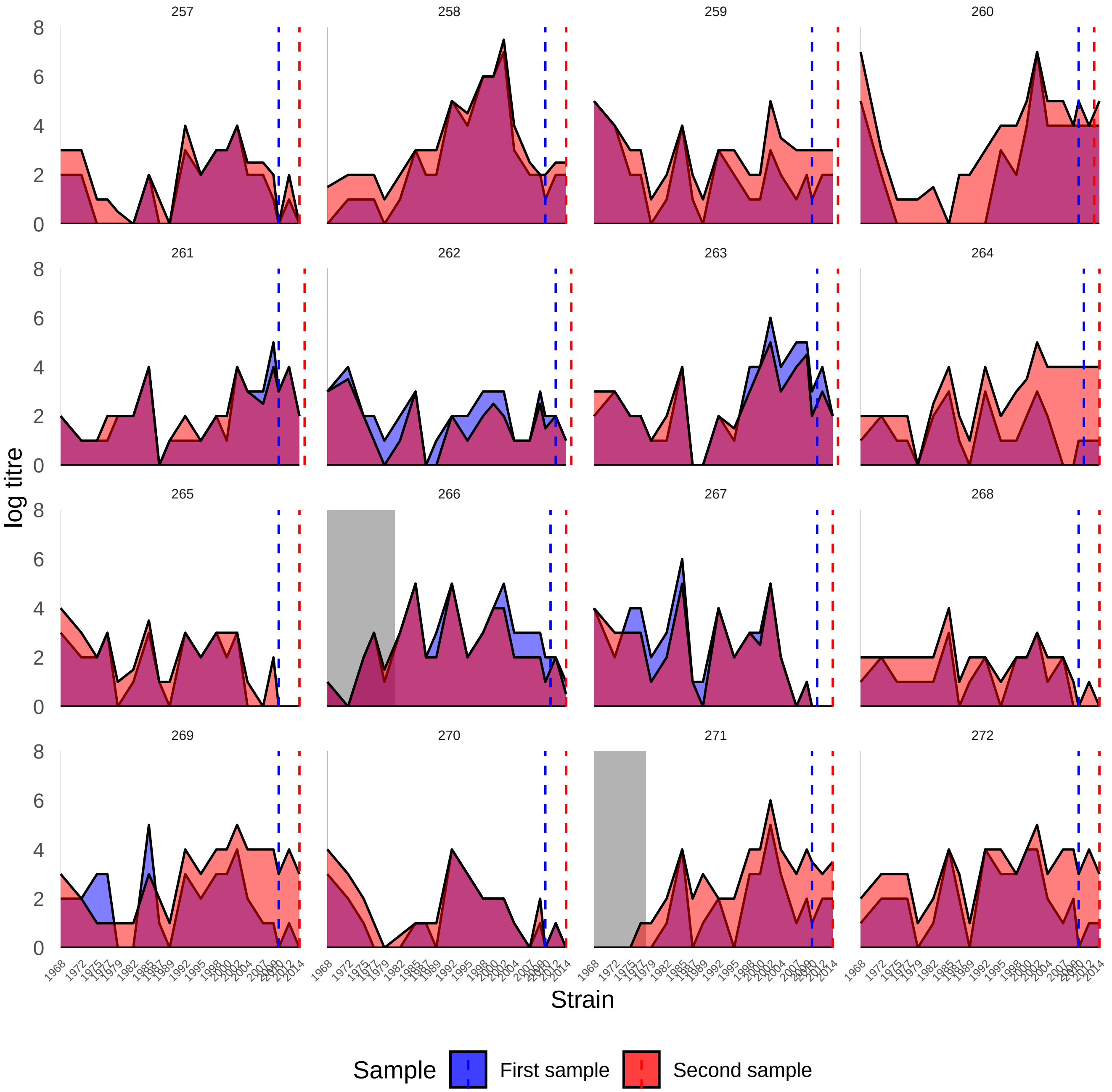
257
258
259
260
261
262
263
264
265
266
267
268
269
270
271
272
log titre
Strain
Sample
First sample
Second sample

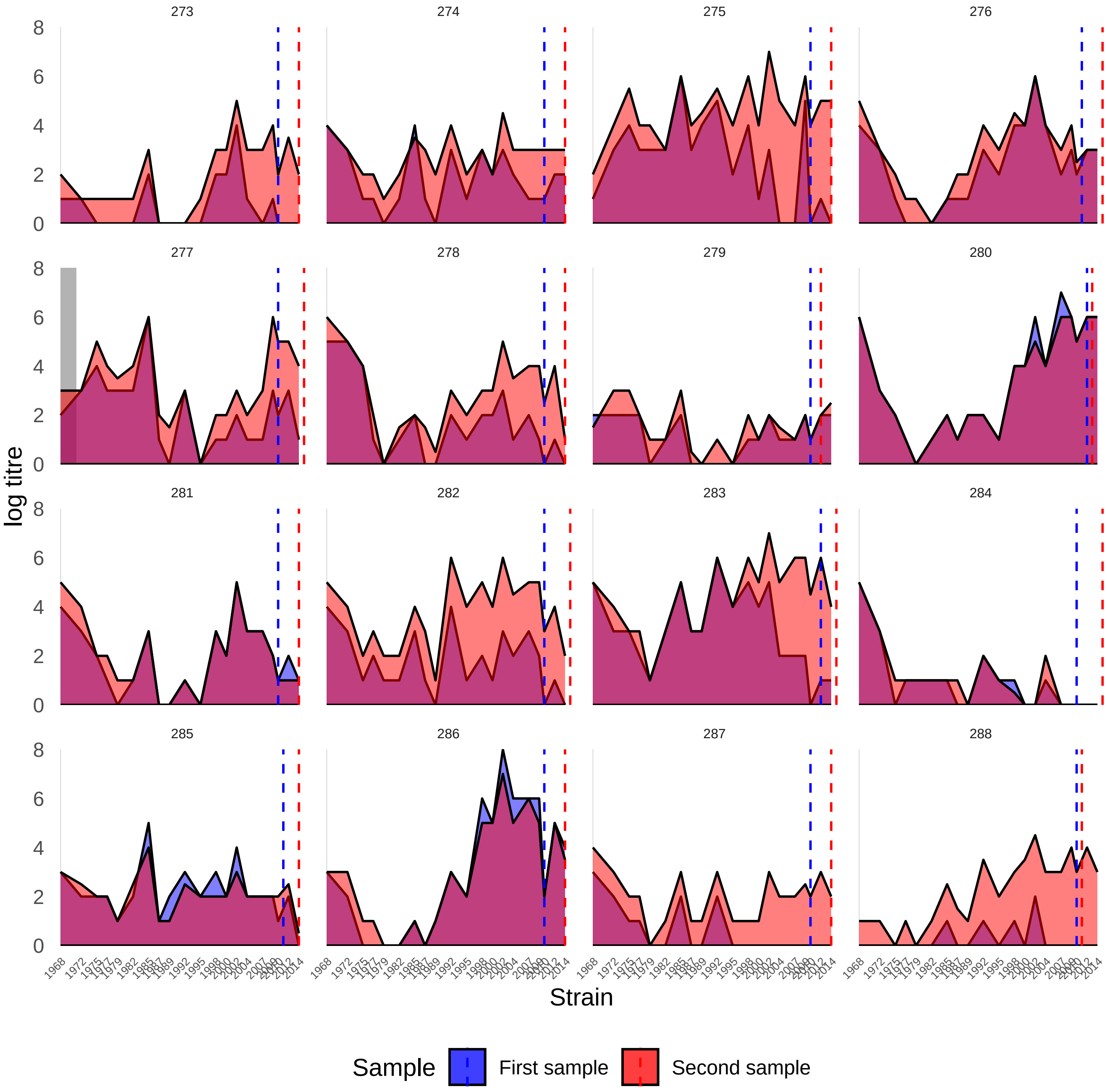
273
274
275
276
277
278
279
280
281
282
283
284
285
286
287
288
log titre
Strain
Sample
First sample
Second sample

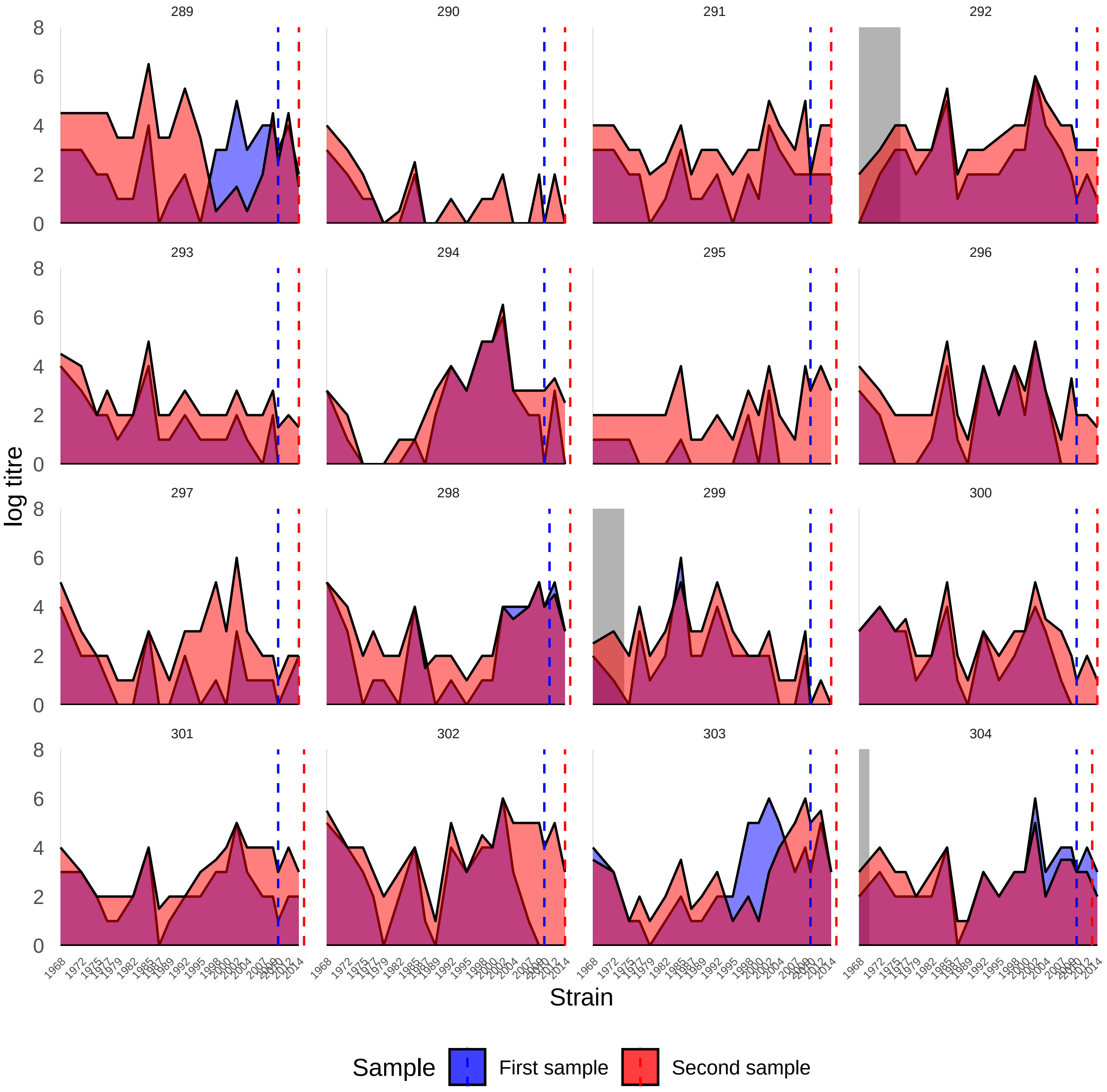

289
290
291
292
293
294
295
296
297
298
299
300
301
302
303
304
log titre
Strain
Sample
First sample
Second sample

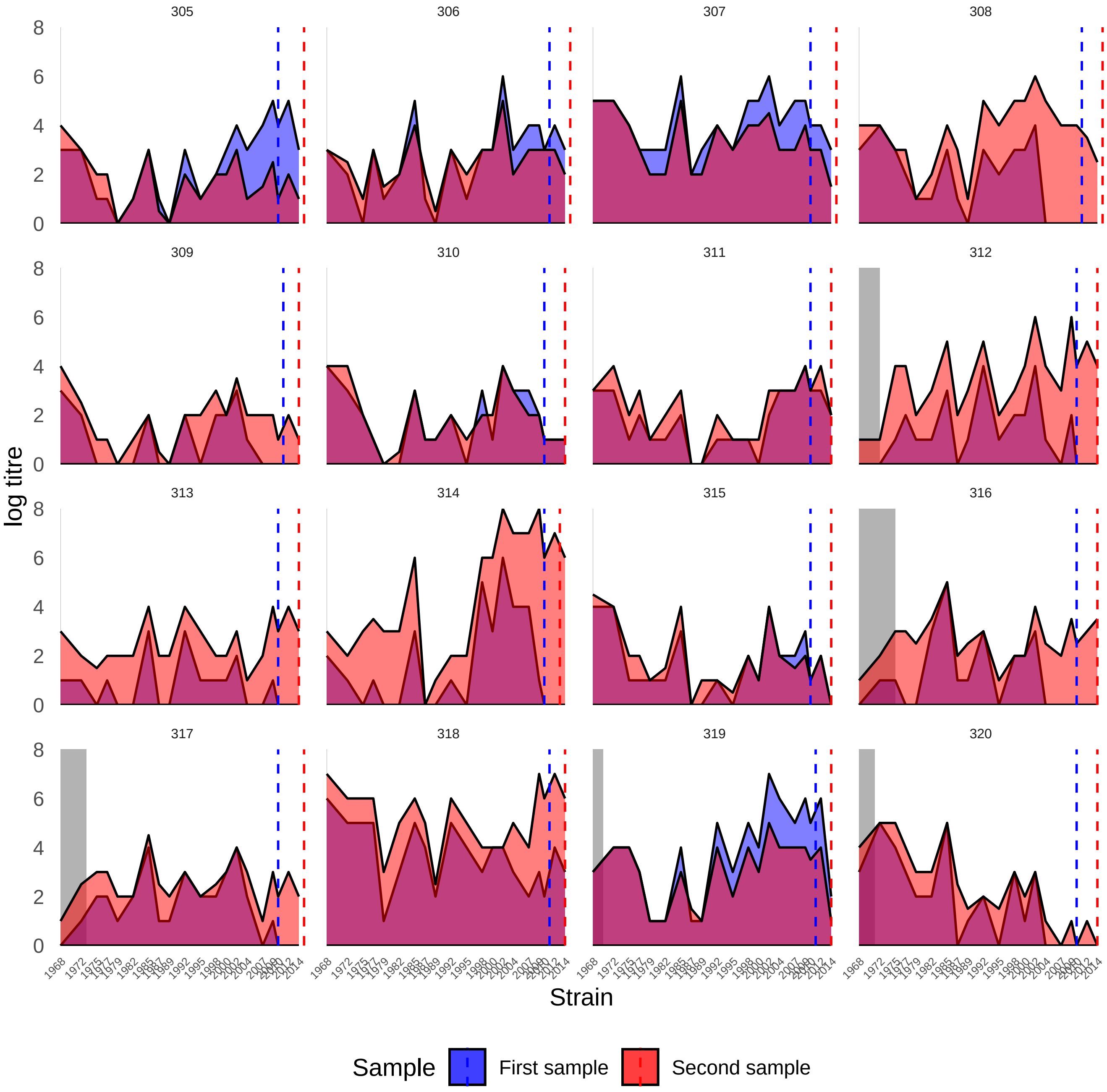

305
306
307
308
309
310
311
312
313
314
315
316
317
318
319
320
log titre
Strain
Sample
First sample
Second sample

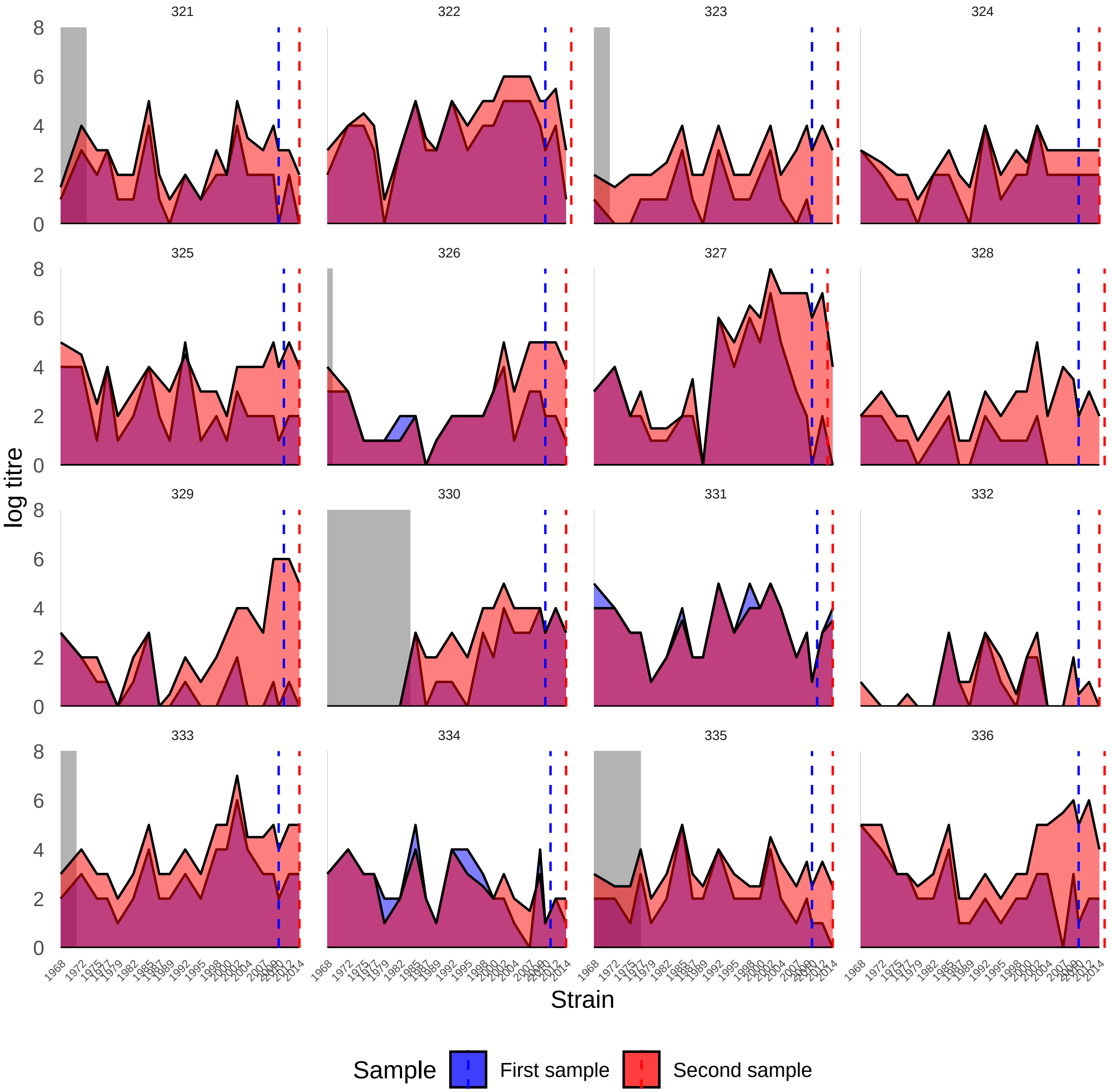

321
322
323
324
325
326
327
328
329
330
331
332
333
334
335
336
log titre
Strain
Sample
First sample
Second sample

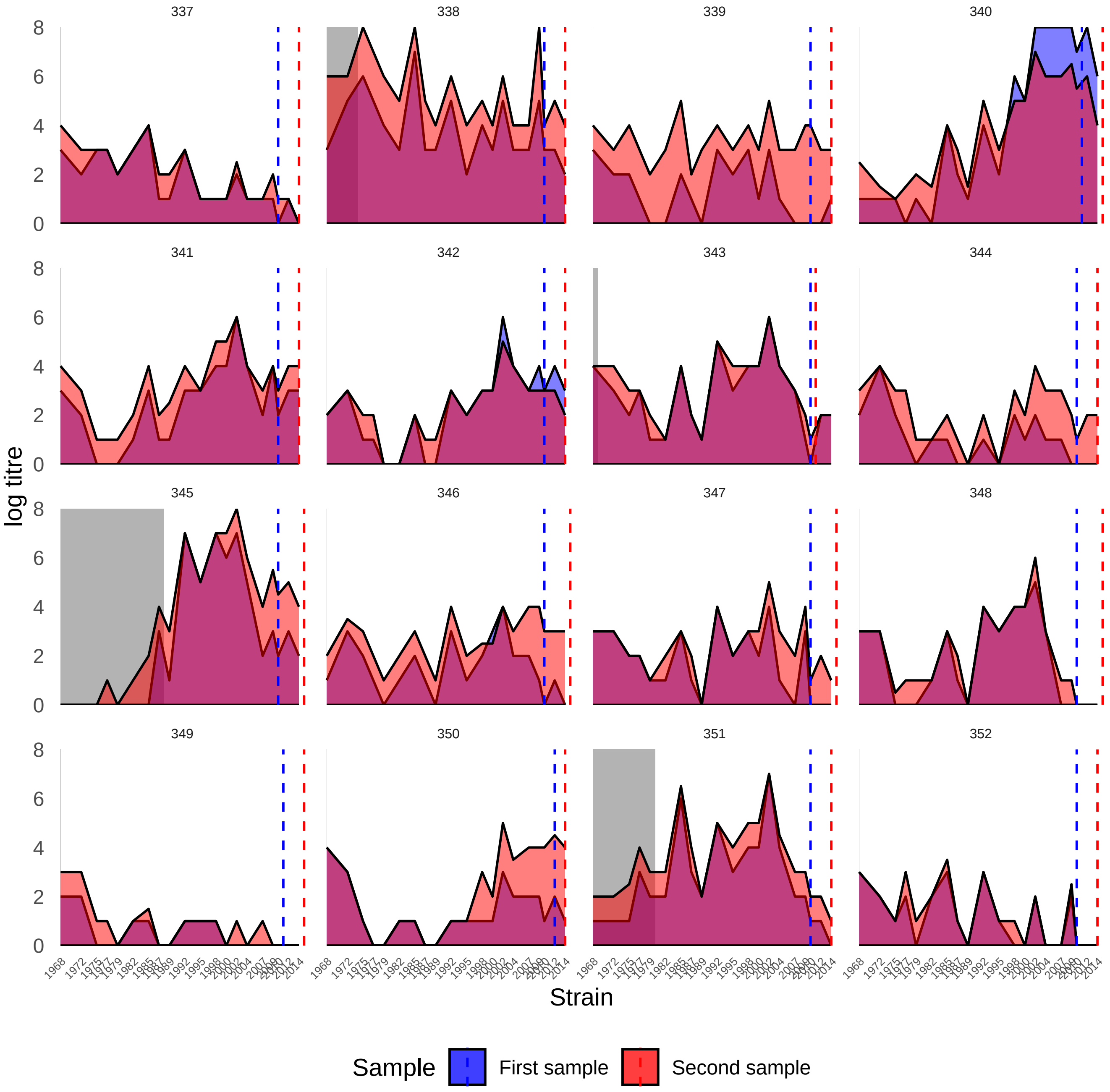

337
338
339
340
341
342
343
344
345
346
347
348
349
350
351
352
log titre
0
2
4
6
8
1968
1972
1975
1977
1979
1982
1985
1987
1989
1992
1995
1998
2000
2002
2004
2007
2009
2010
2012
2014
Strain
Sample
First sample
Second sample

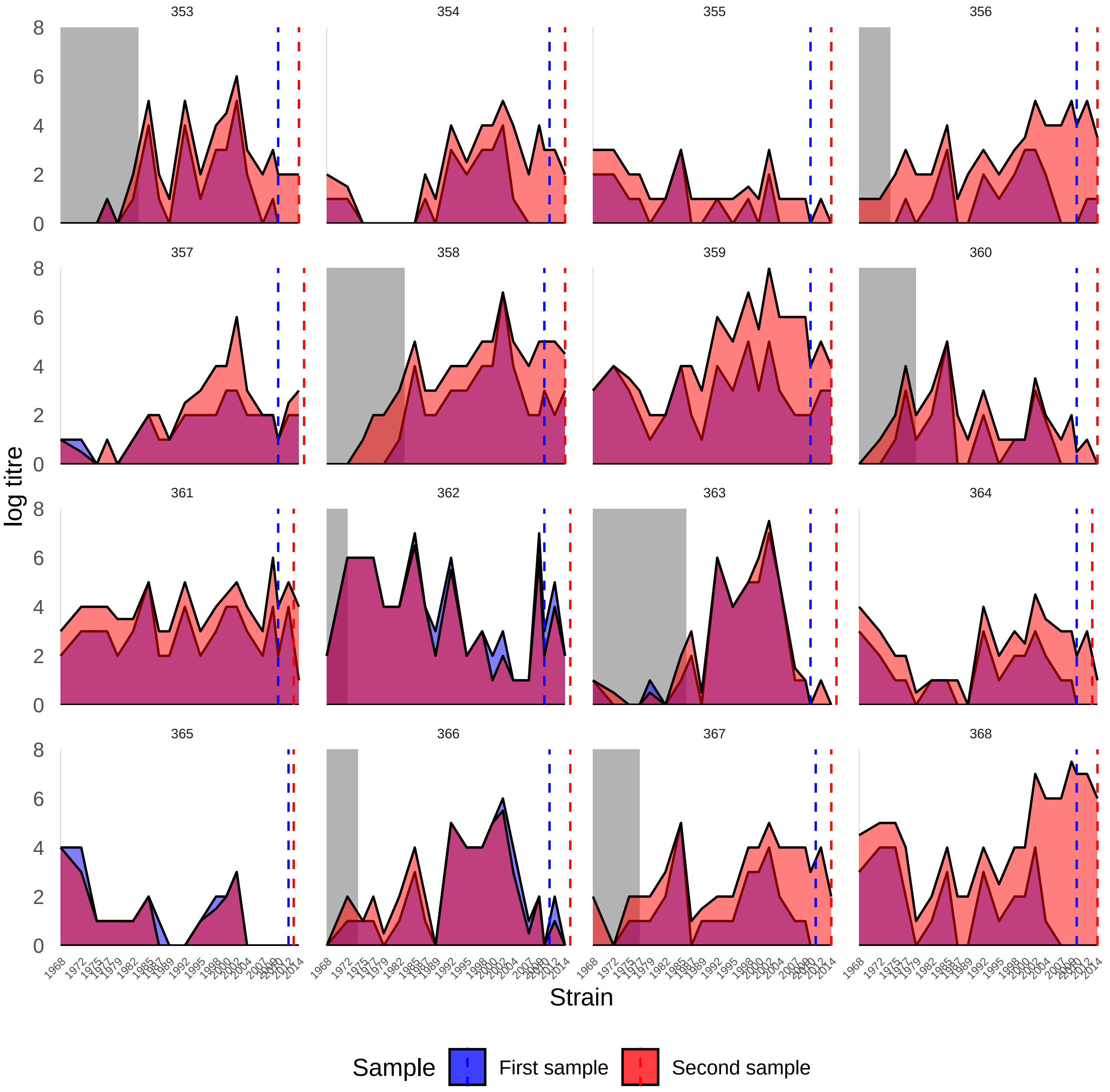
353
354
355
356
357
358
359
360
361
362
363
364
365
366
367
368
log titre
Strain
1968
1972
1975
1977
1979
1982
1985
1987
1989
1992
1995
1998
2000
2002
2004
2007
2009
2010
2012
2014
Sample
First sample
Second sample

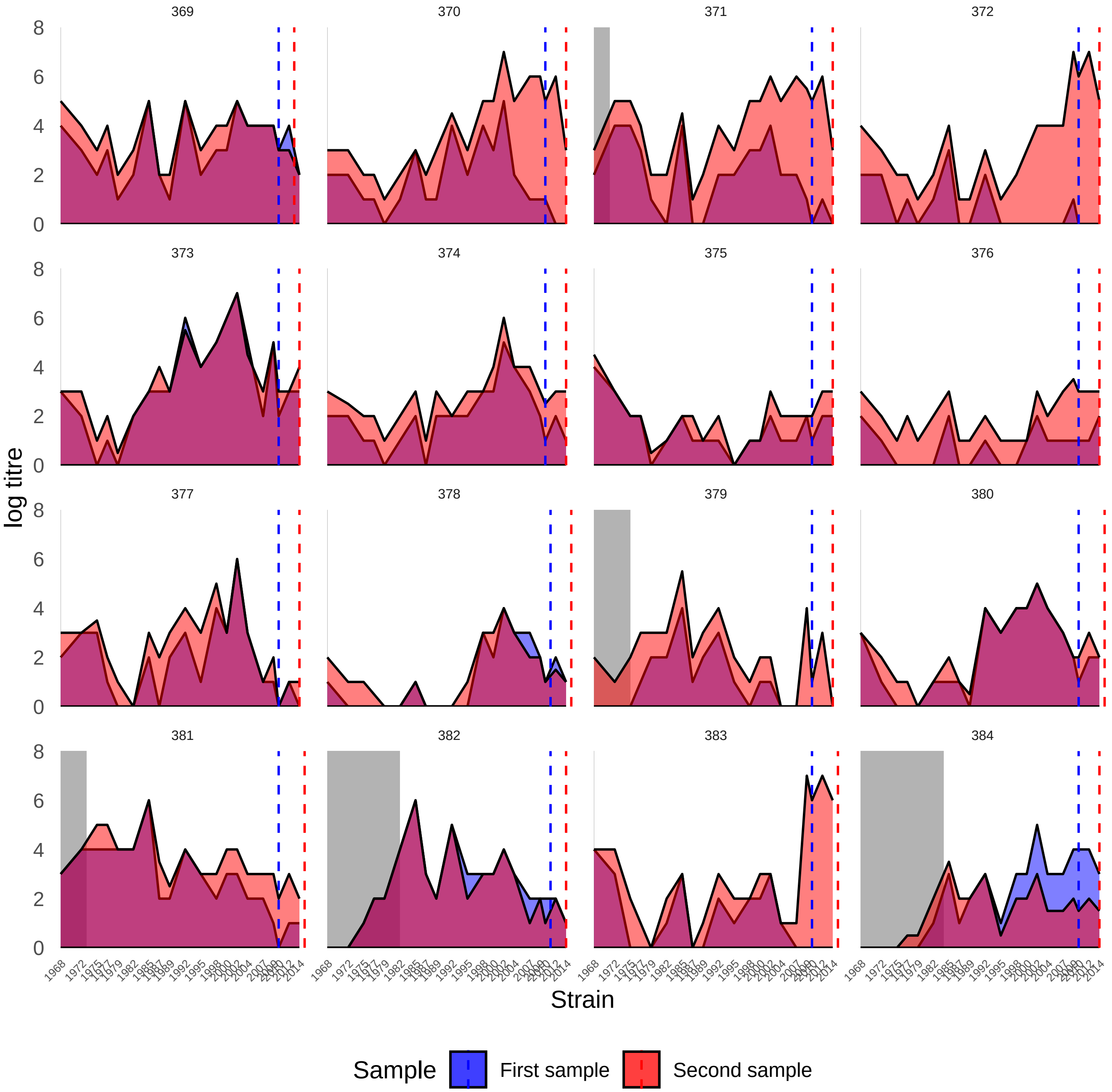

369
370
371
372
373
374
375
376
377
378
379
380
381
382
383
384
log titre
Strain
Sample
First sample
Second sample

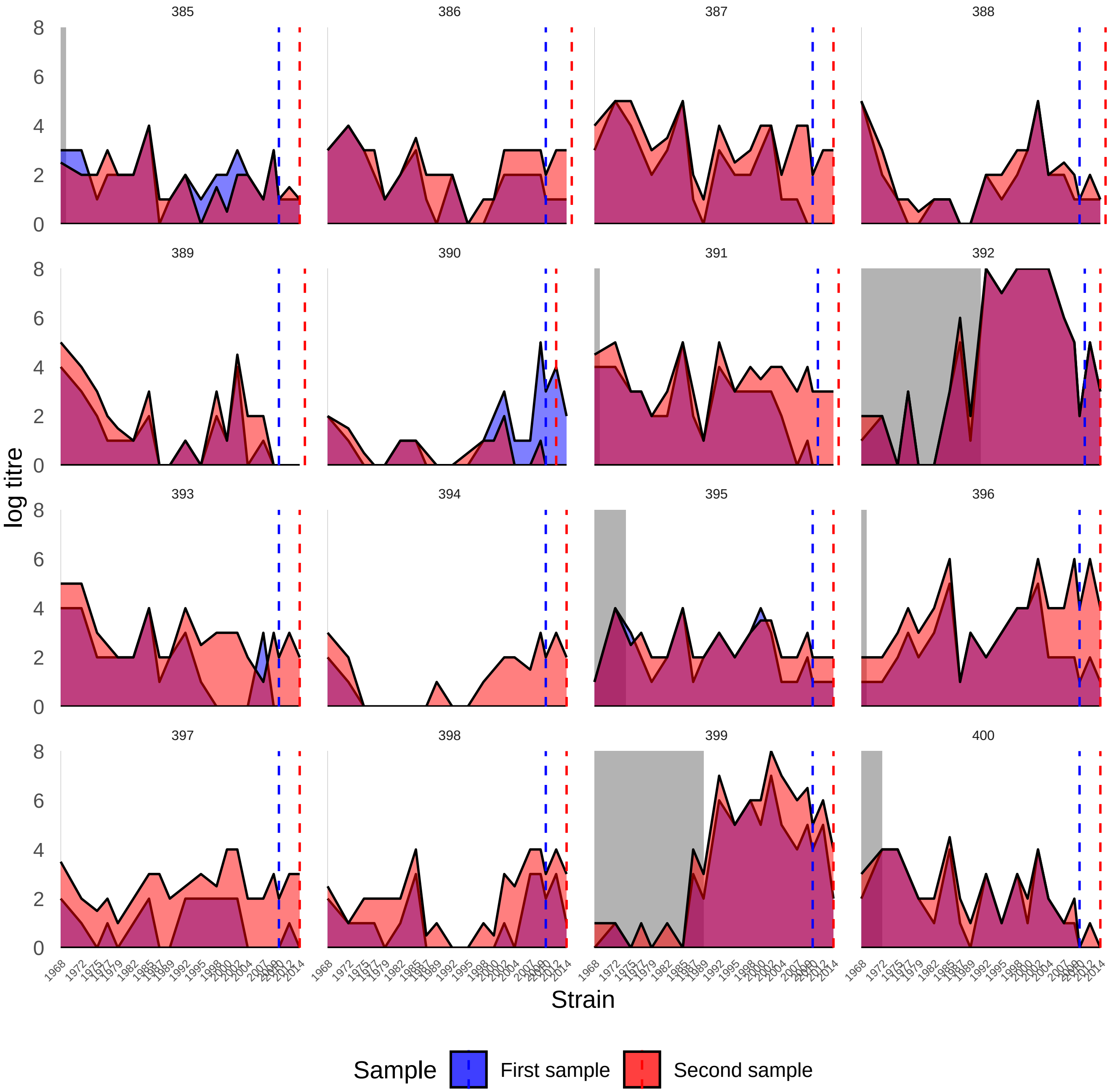

385
386
387
388
389
390
391
392
393
394
395
396
397
398
399
400
log titre
0
2
4
6
8
1968
1972
1975
1977
1979
1982
1985
1987
1989
1992
1995
1998
2000
2002
2004
2007
2009
2010
2012
2014
Strain
Sample
First sample
Second sample

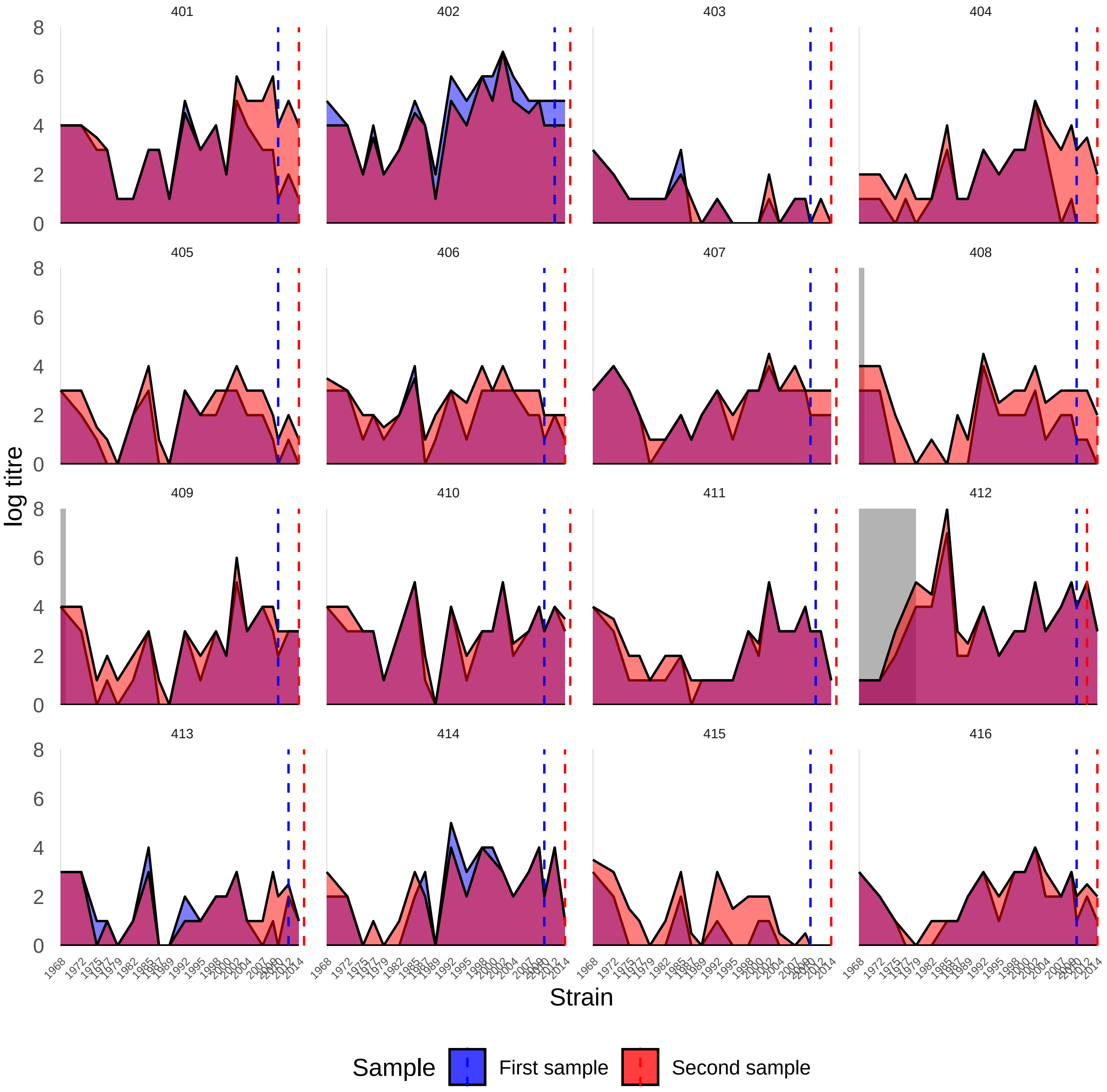

401
402
403
404
405
406
407
408
409
410
411
412
413
414
415
416
log titre
Strain
Sample
First sample
Second sample

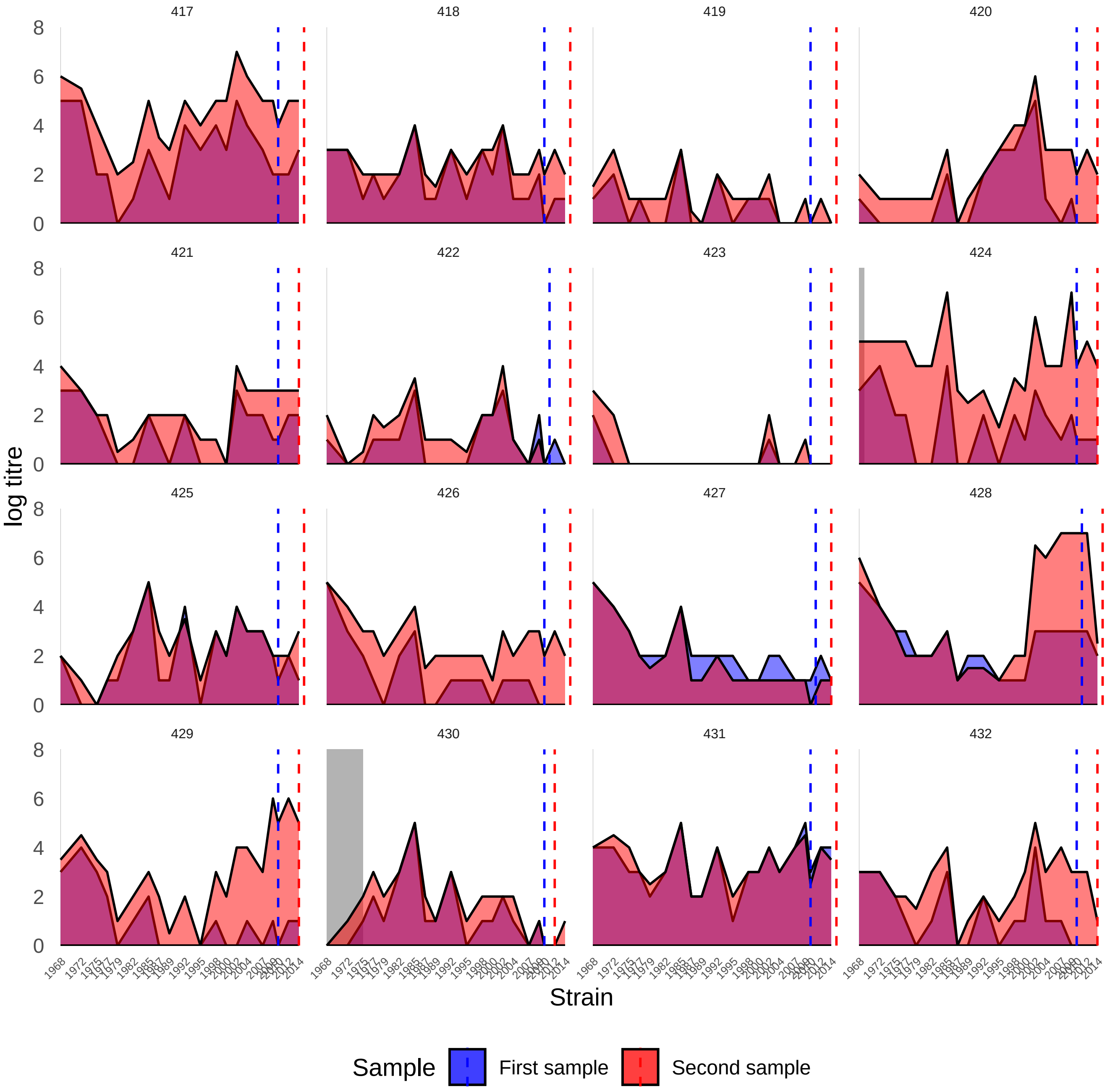
417
418
419
420
421
422
423
424
425
426
427
428
429
430
431
432
log titre
0
2
4
6
8
1968
1972
1975
1977
1979
1982
1985
1987
1989
1992
1995
1998
2000
2002
2004
2007
2009
2010
2012
2014
Strain
Sample
First sample
Second sample

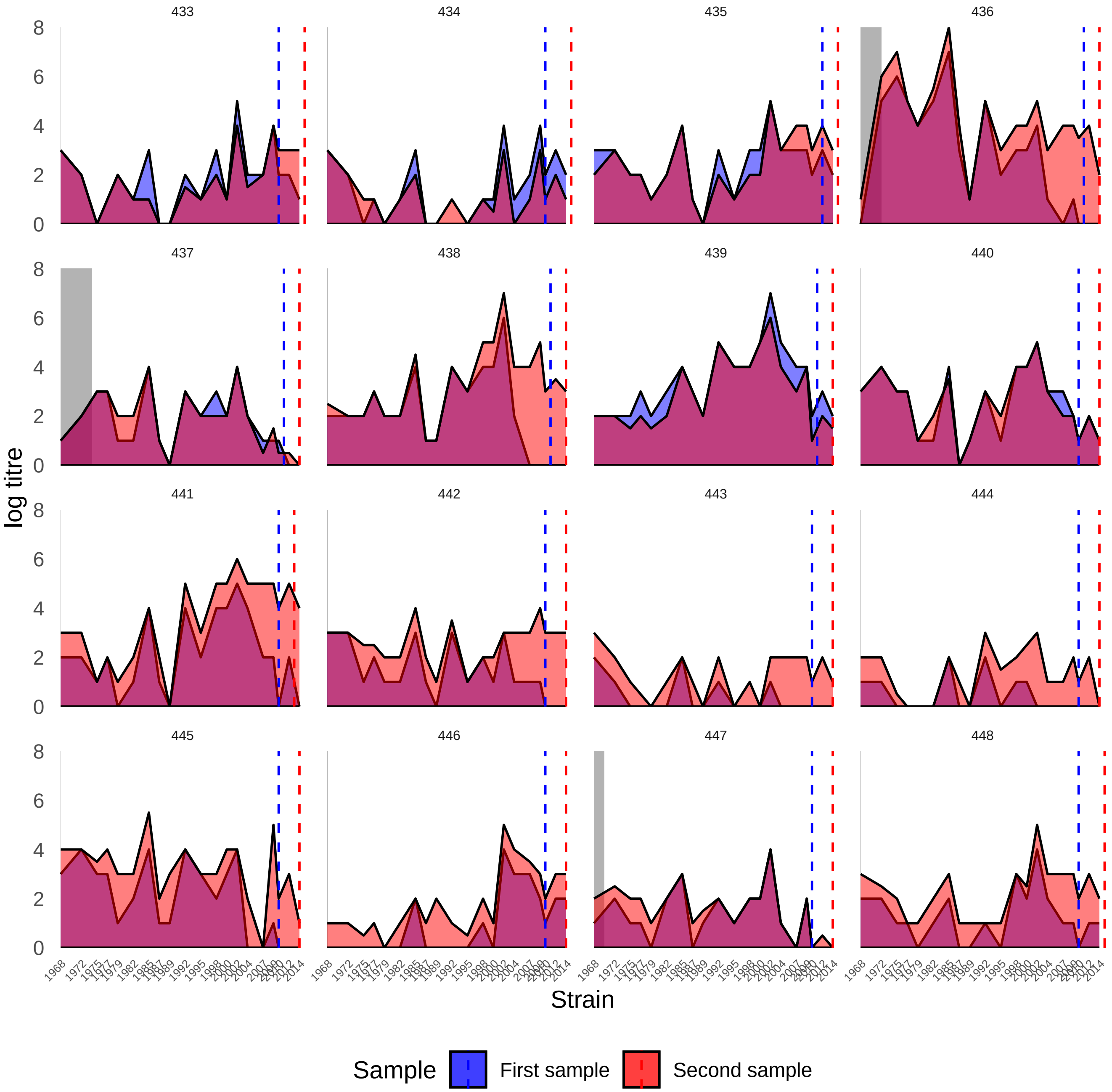
433
434
435
436
437
438
439
440
441
442
443
444
445
446
447
448
log titre
Strain
Sample
First sample
Second sample

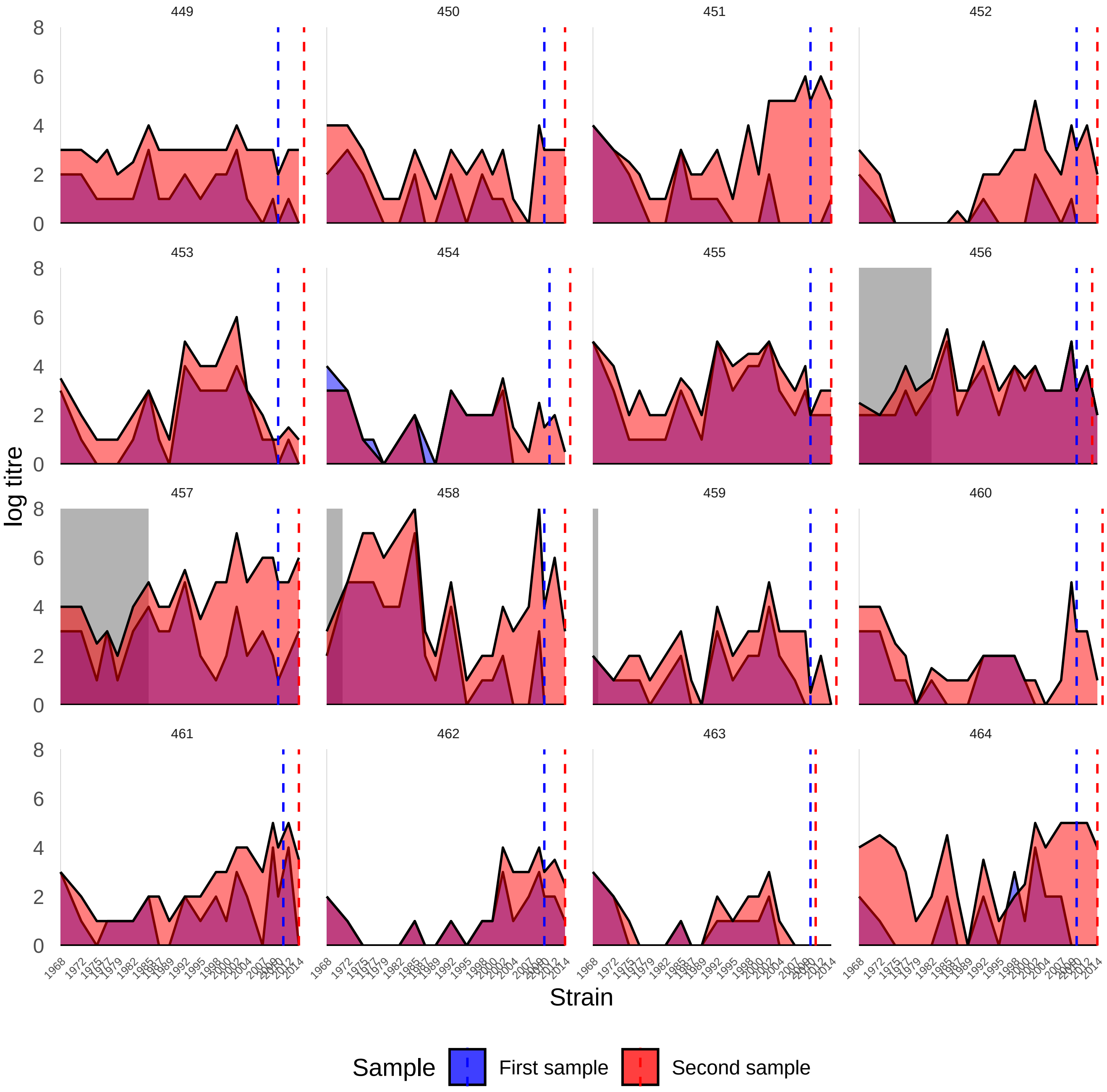
449
450
451
452
453
454
455
456
457
458
459
460
461
462
463
464
log titre
Strain
Sample
First sample
Second sample

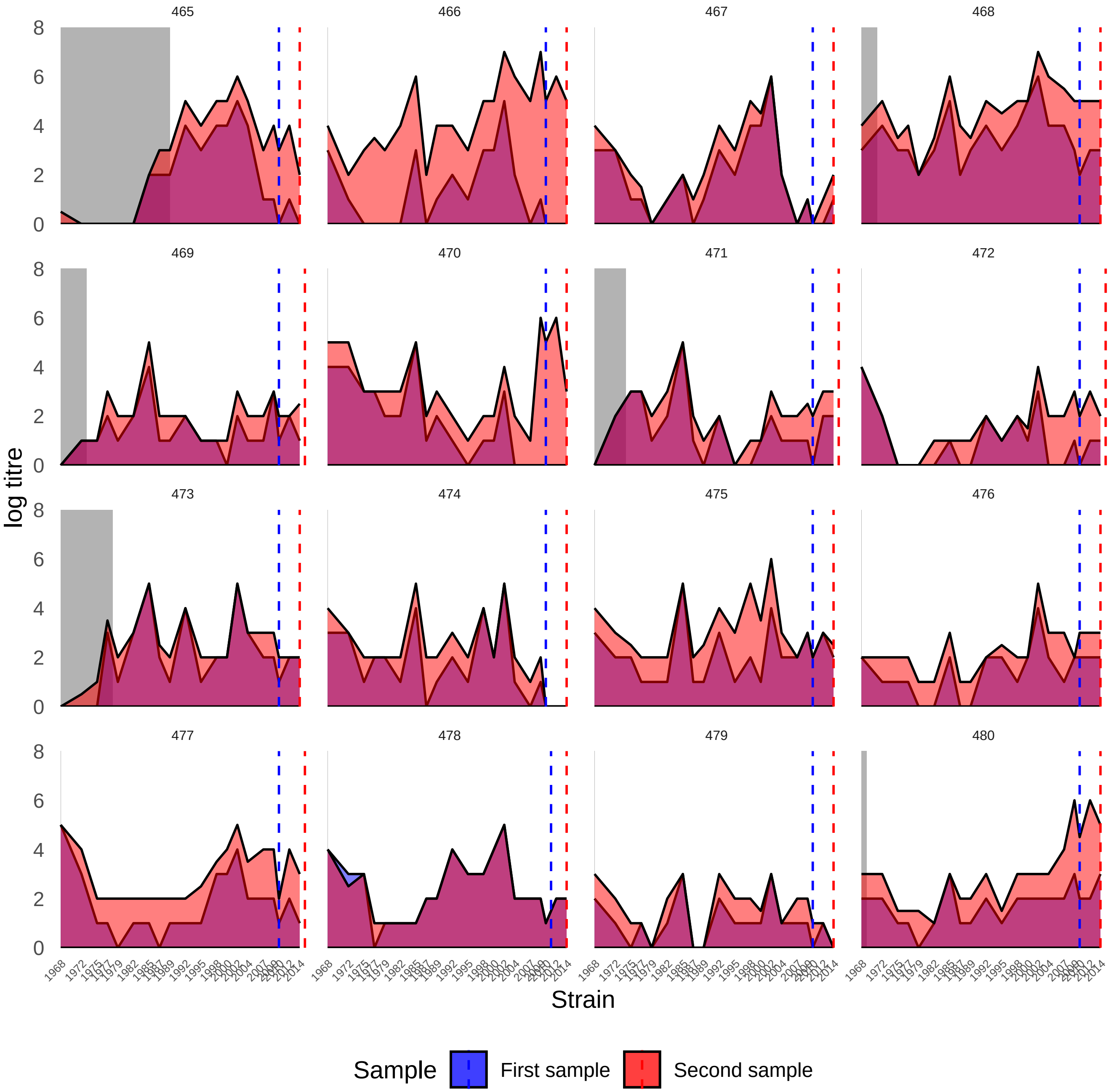

465
466
467
468
469
470
471
472
473
474
475
476
477
478
479
480
log titre
Strain
Sample
First sample
Second sample

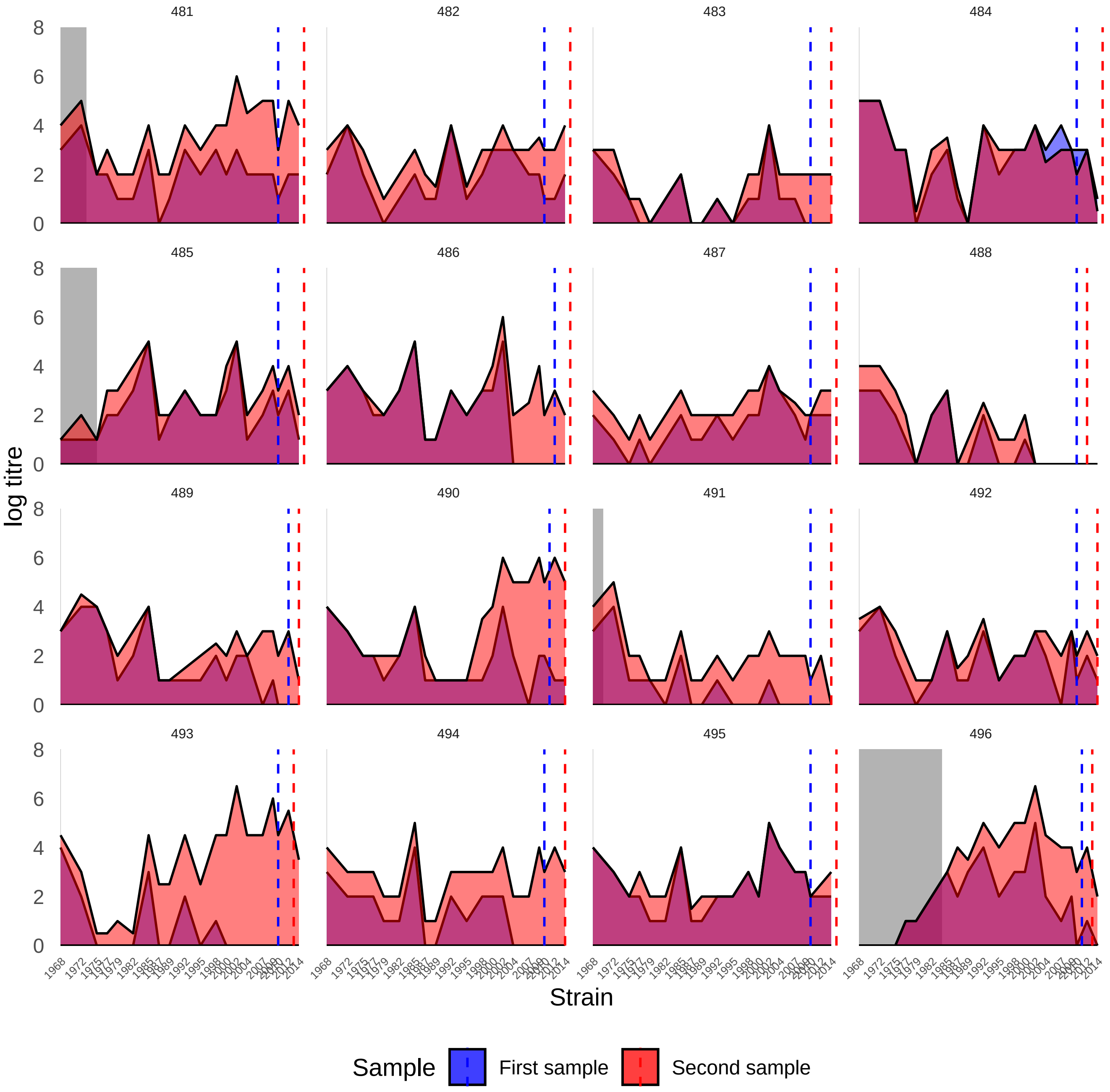

481
482
483
484
485
486
487
488
489
490
491
492
493
494
495
496
log titre
Strain
Sample
First sample
Second sample

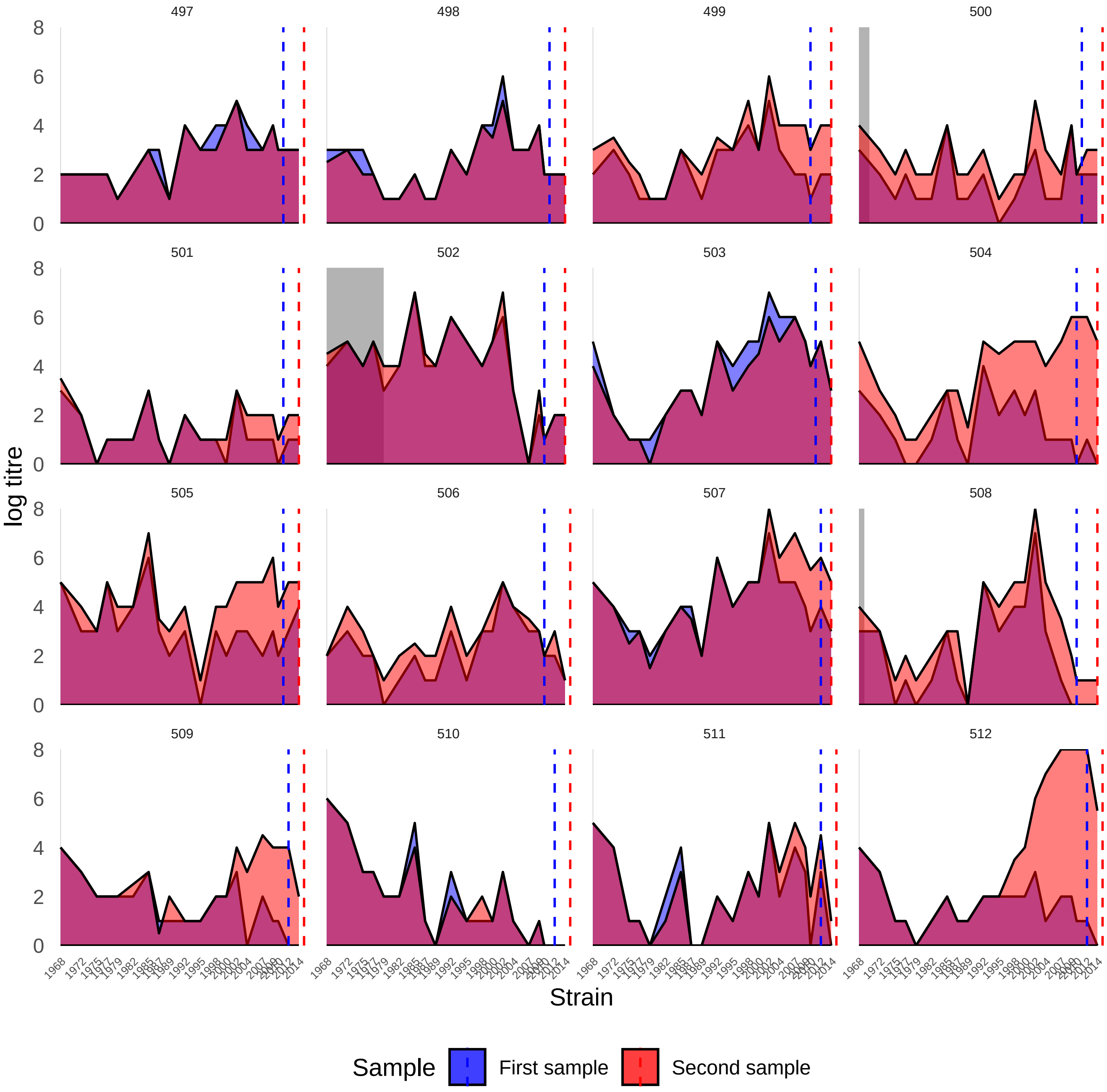

497
498
499
500
501
502
503
504
505
506
507
508
509
510
511
512
log titre
Strain
Sample
First sample
Second sample

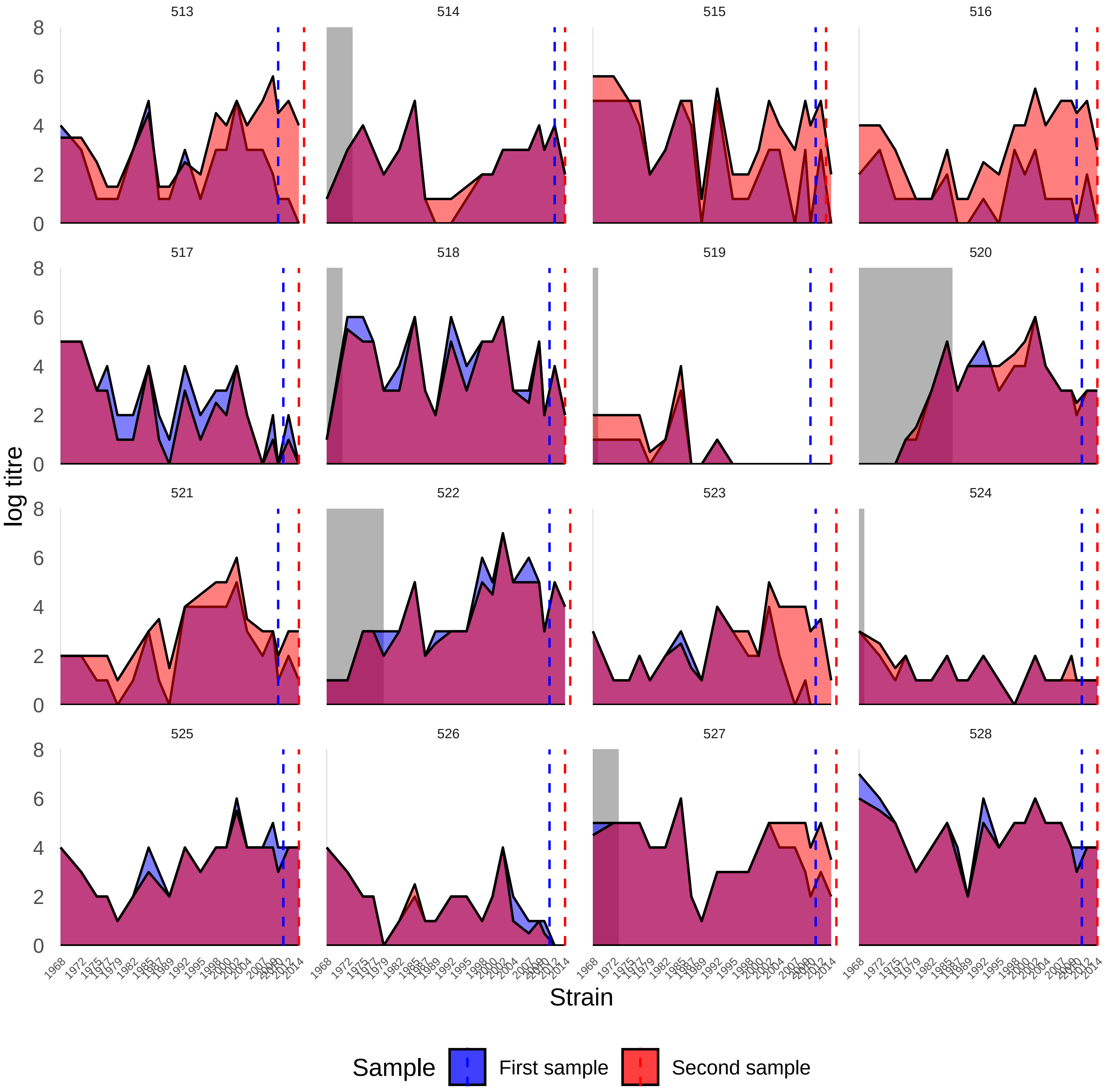
513
514
515
516
517
518
519
520
521
522
523
524
525
526
527
528
log titre
Strain
Sample
First sample
Second sample

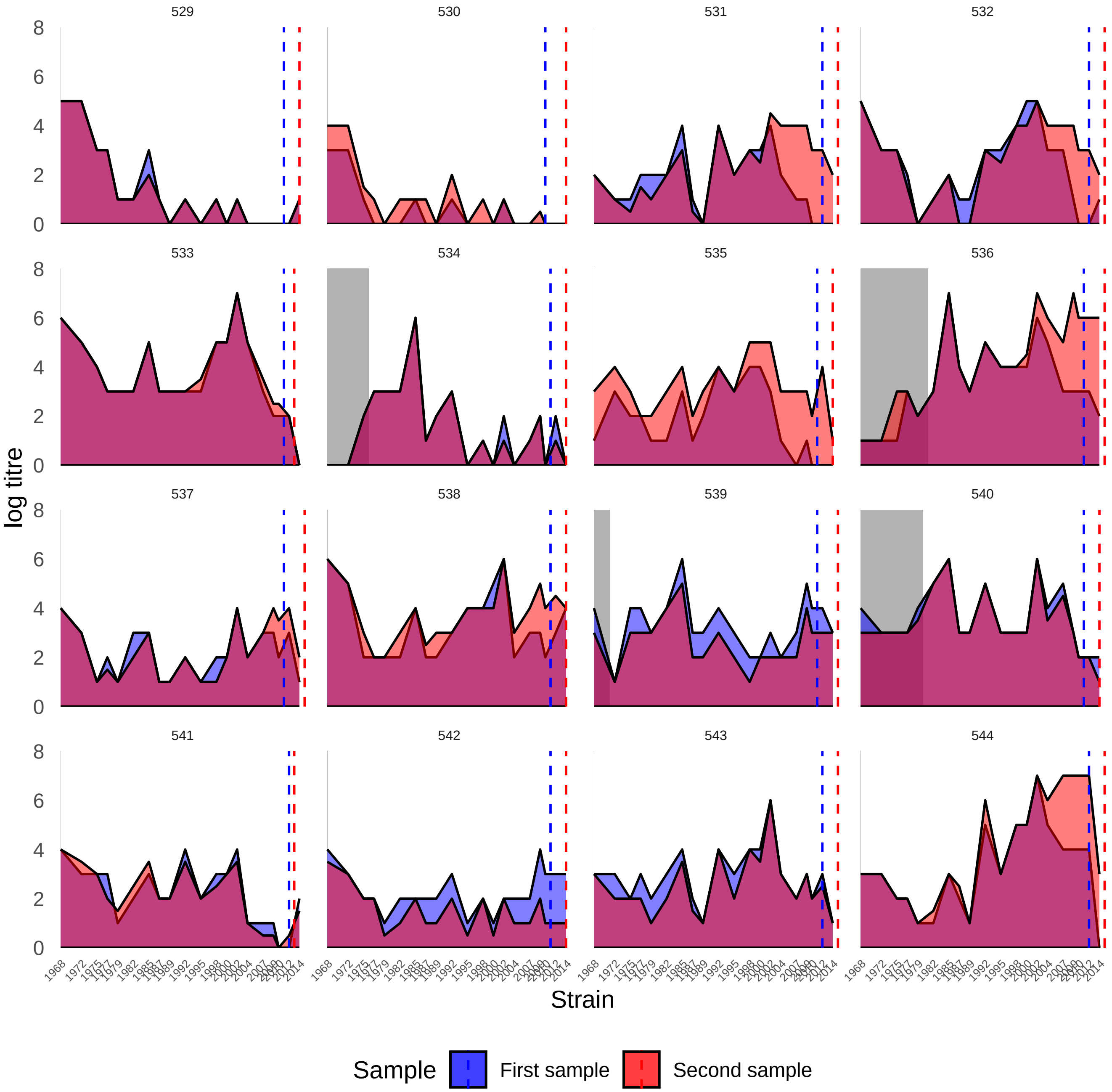
529
530
531
532
533
534
535
536
537
538
539
540
541
542
543
544
log titre
Strain
1968
1972
1975
1977
1979
1982
1985
1987
1989
1992
1995
1998
2000
2002
2004
2007
2009
2010
2012
2014
Sample
First sample
Second sample

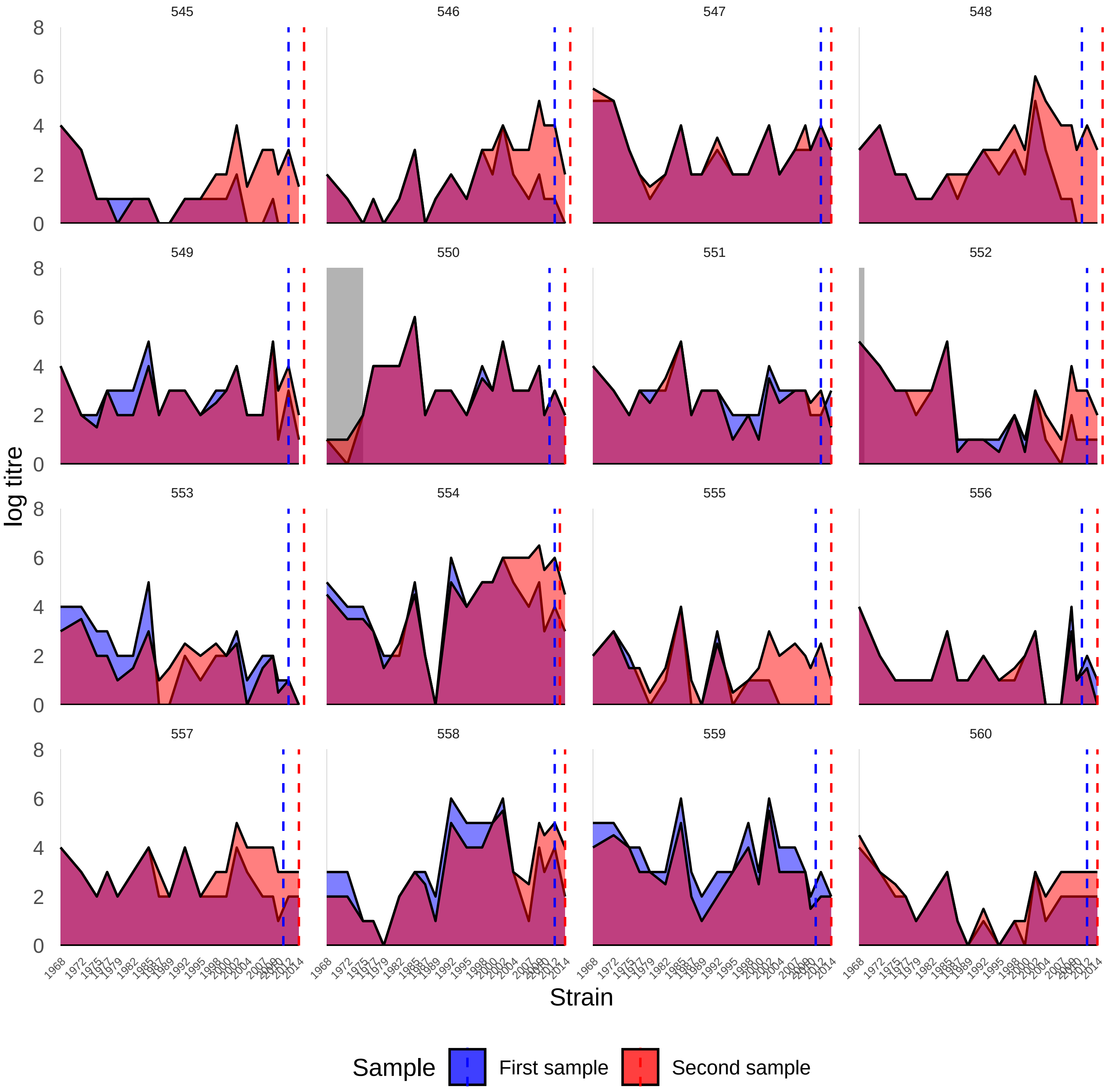

545
546
547
548
549
550
551
552
553
554
555
556
557
558
559
560
log titre
0
2
4
6
8
1968
1972
1975
1977
1979
1982
1985
1987
1989
1992
1995
1998
2000
2002
2004
2007
2009
2010
2012
2014
Strain
Sample
First sample
Second sample

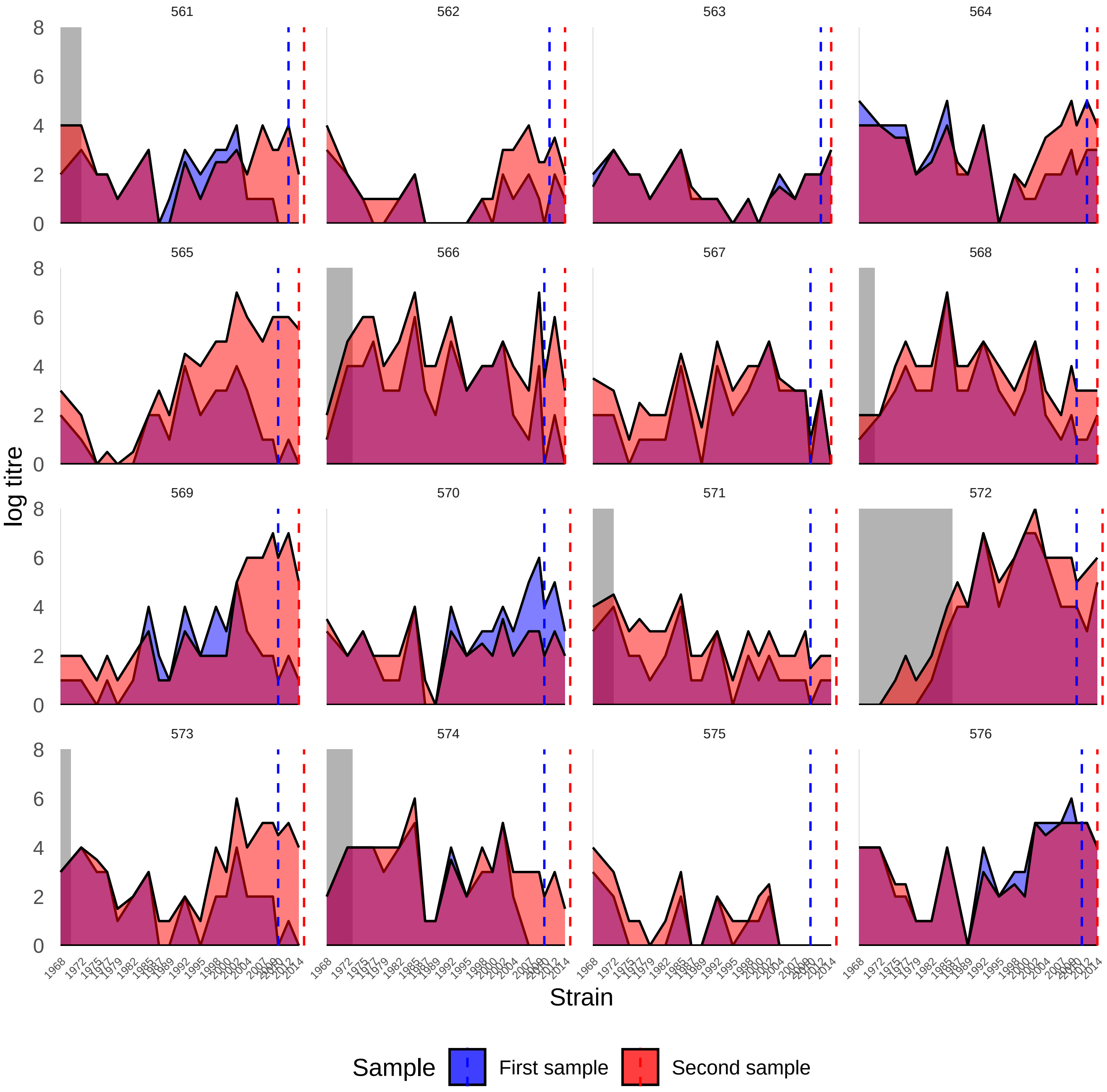
561
562
563
564
565
566
567
568
569
570
571
572
573
574
575
576
log titre
Strain
Sample
First sample
Second sample

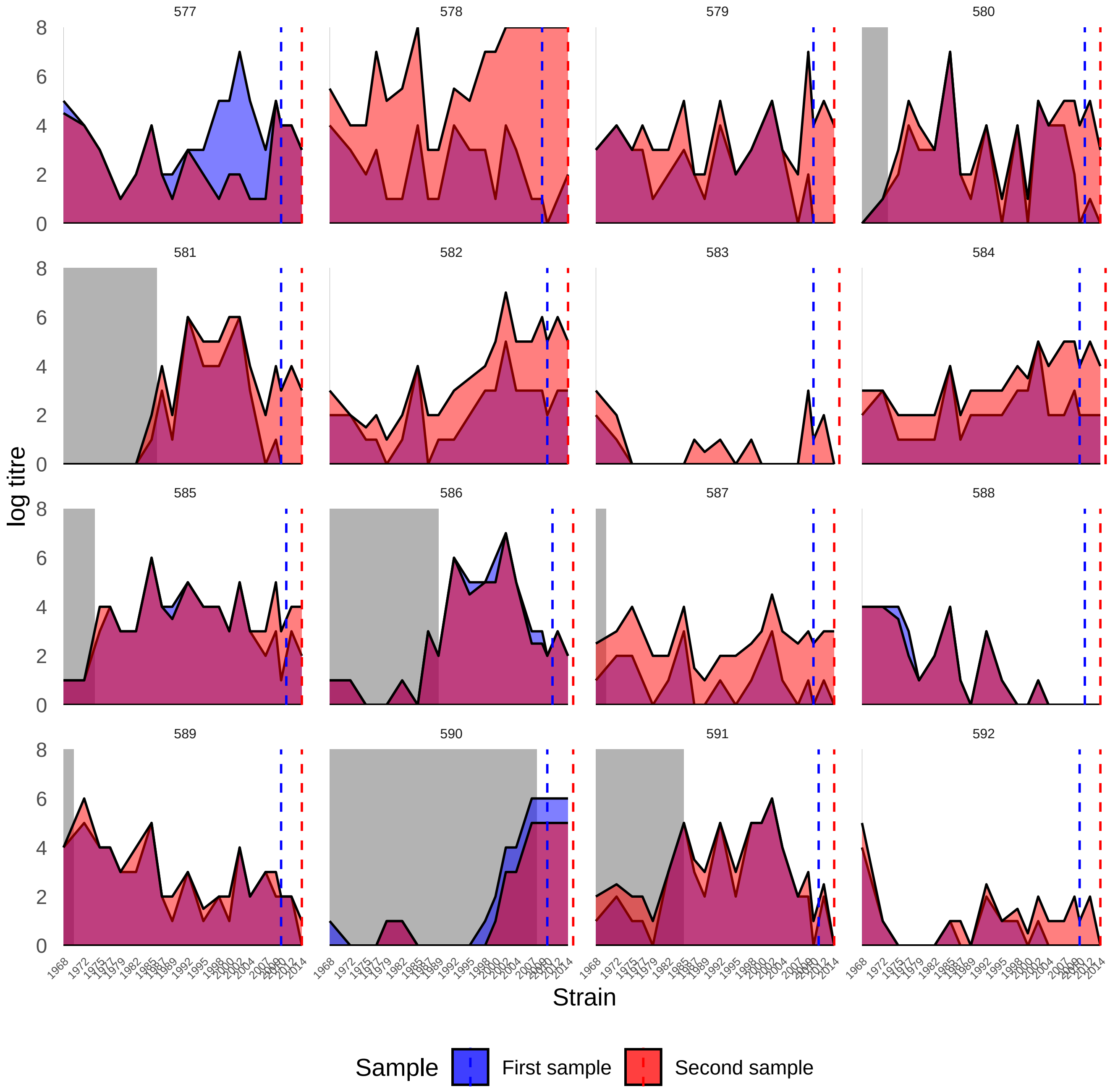

577
578
579
580
581
582
583
584
585
586
587
588
589
590
591
592
log titre
Strain
Sample
First sample
Second sample

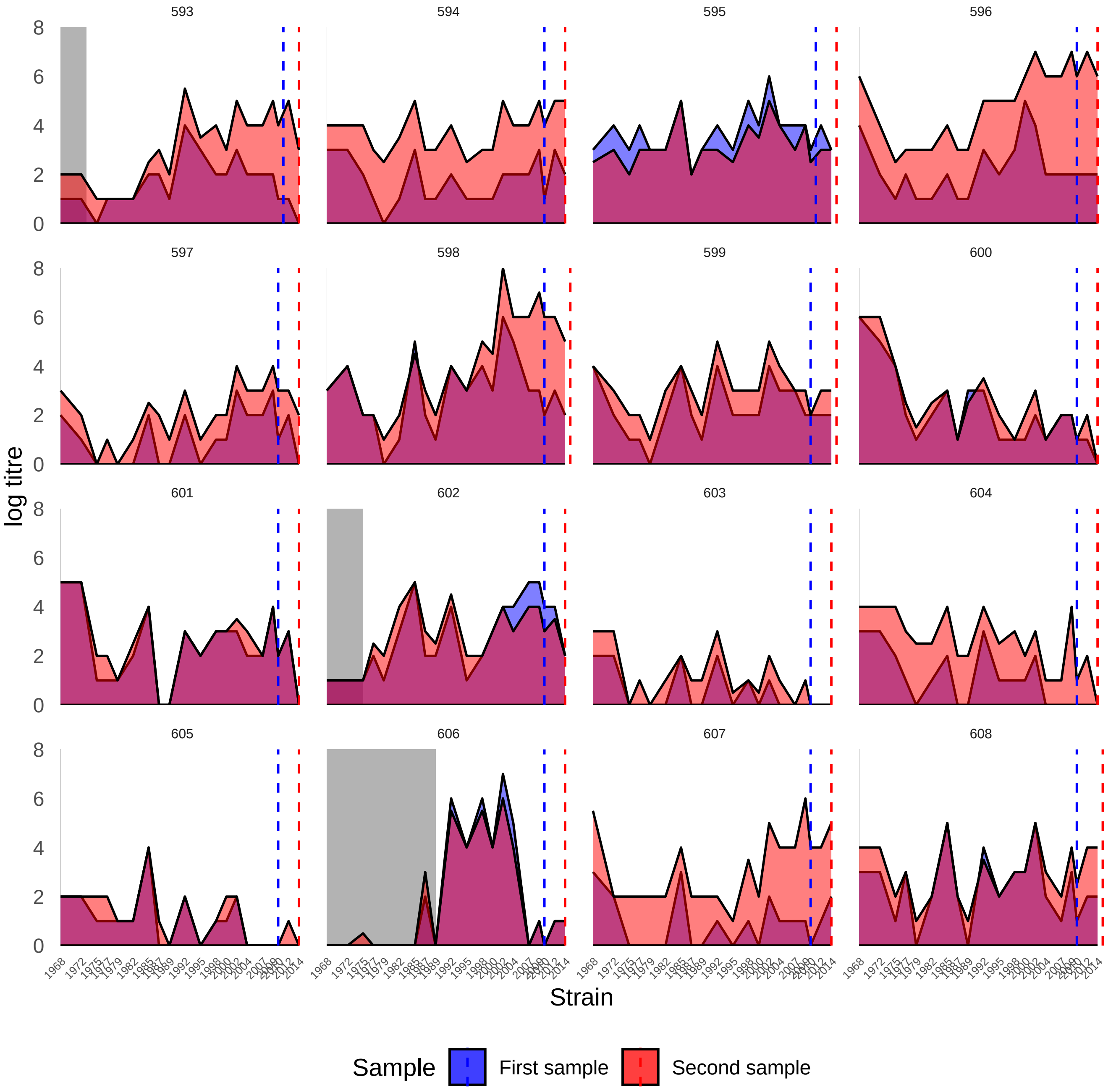

593
594
595
596
597
598
599
600
601
602
603
604
605
606
607
608
log titre
Strain
Sample
First sample
Second sample

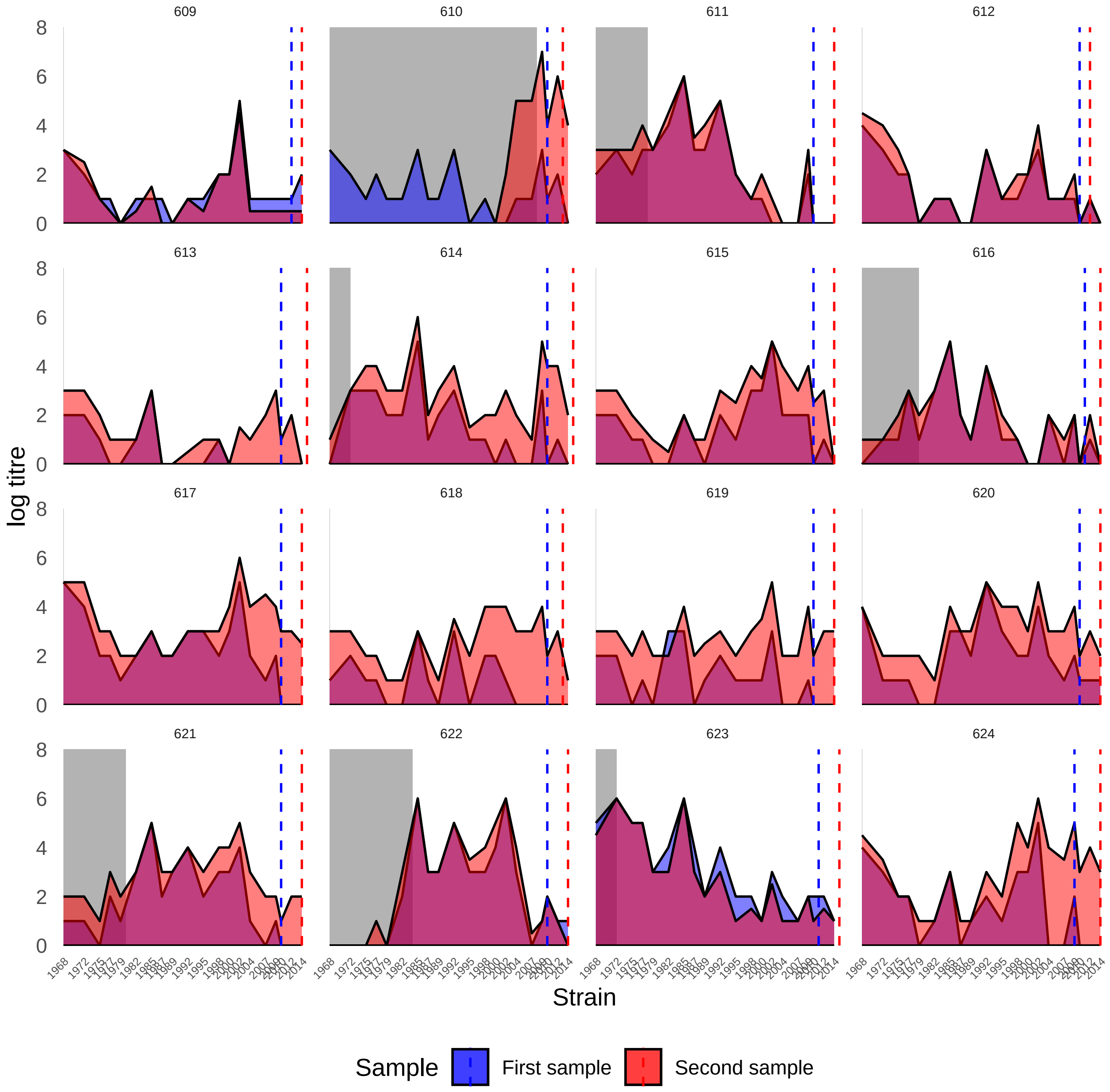
609
610
611
612
613
614
615
616
617
618
619
620
621
622
623
624
log titre
Strain
Sample
First sample
Second sample

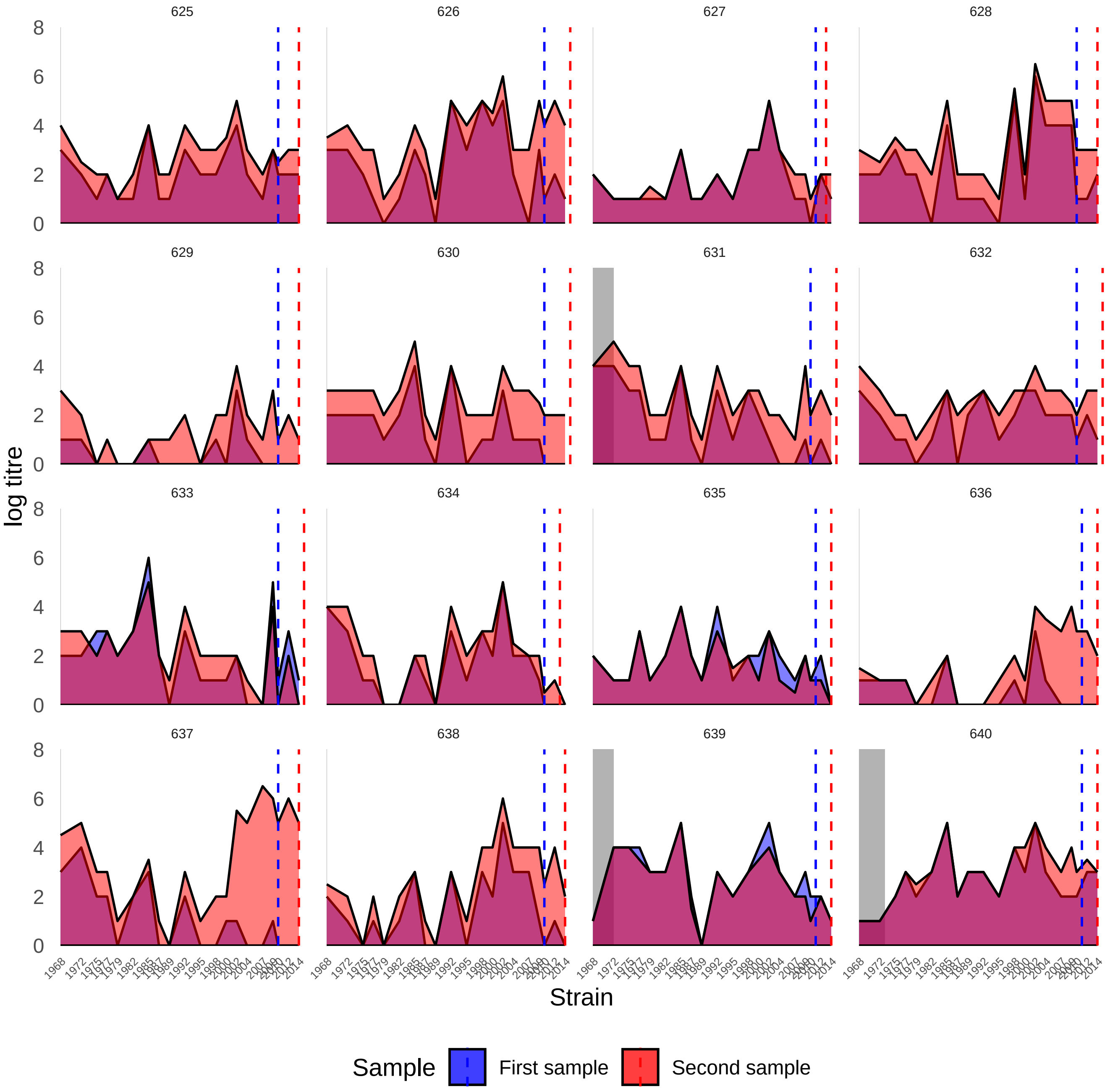
625
626
627
628
629
630
631
632
633
634
635
636
637
638
639
640
log titre
Strain
0
2
4
6
8
1968
1972
1975
1977
1979
1982
1985
1987
1989
1992
1995
1998
2000
2002
2004
2007
2009
2010
2012
2014
Sample
First sample
Second sample

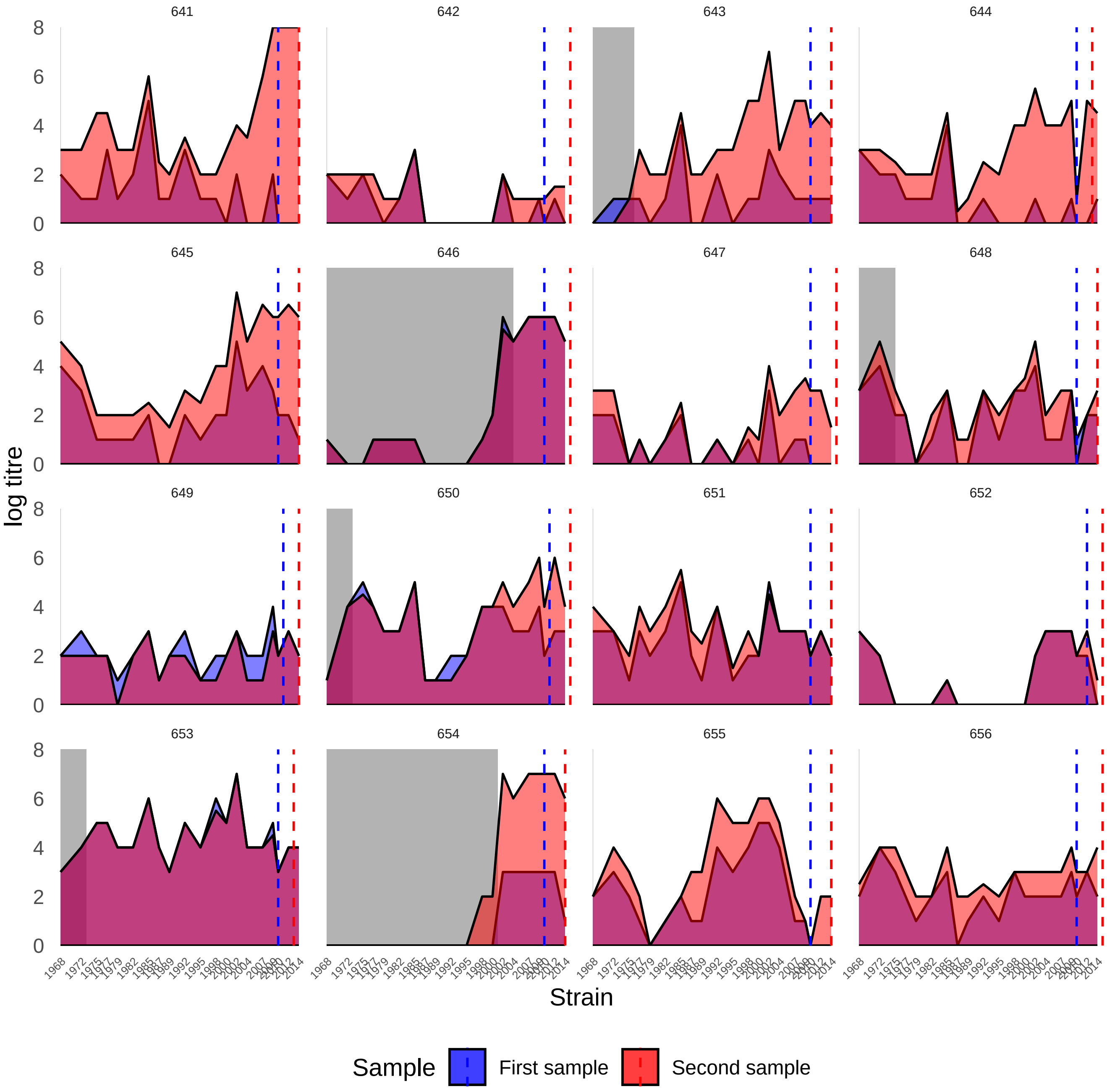

641
642
643
644
645
646
647
648
649
650
651
652
653
654
655
656
log titre
Strain
Sample
First sample
Second sample

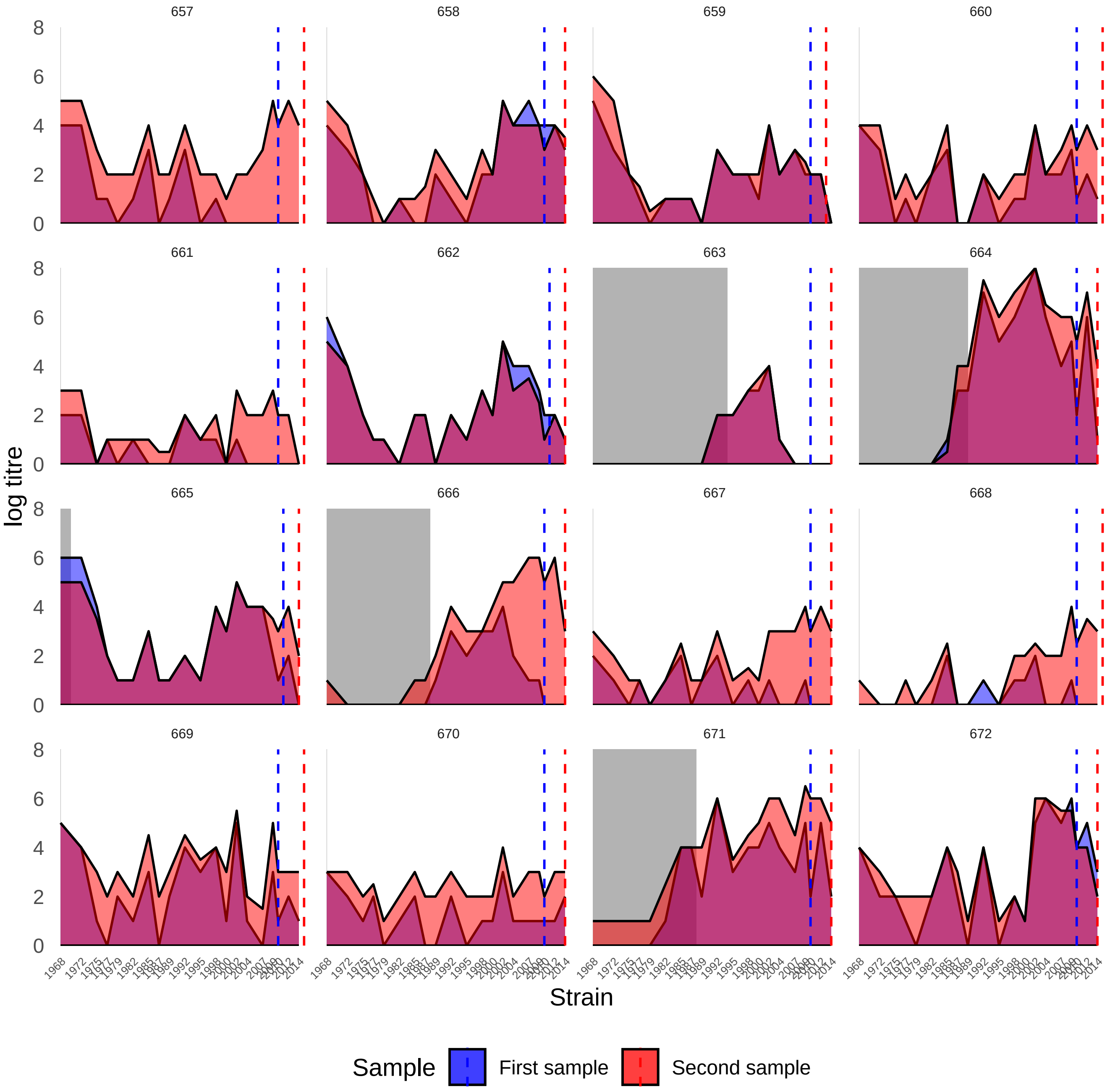

657
658
659
660
661
662
663
664
665
666
667
668
669
670
671
672
log titre
Strain
Sample
First sample
Second sample

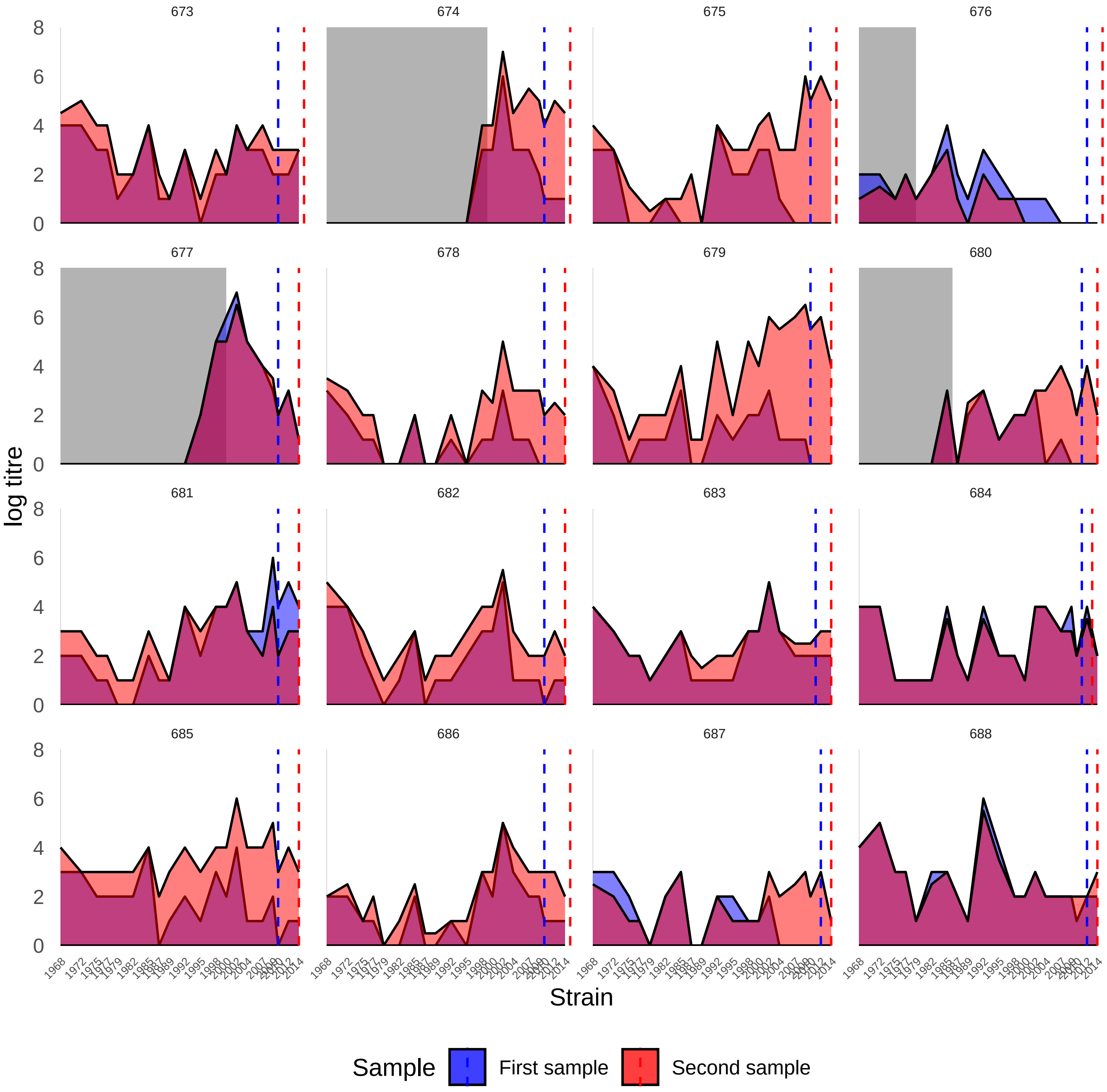
673
674
675
676
677
678
679
680
681
682
683
684
685
686
687
688
log titre
Strain
Sample
First sample
Second sample

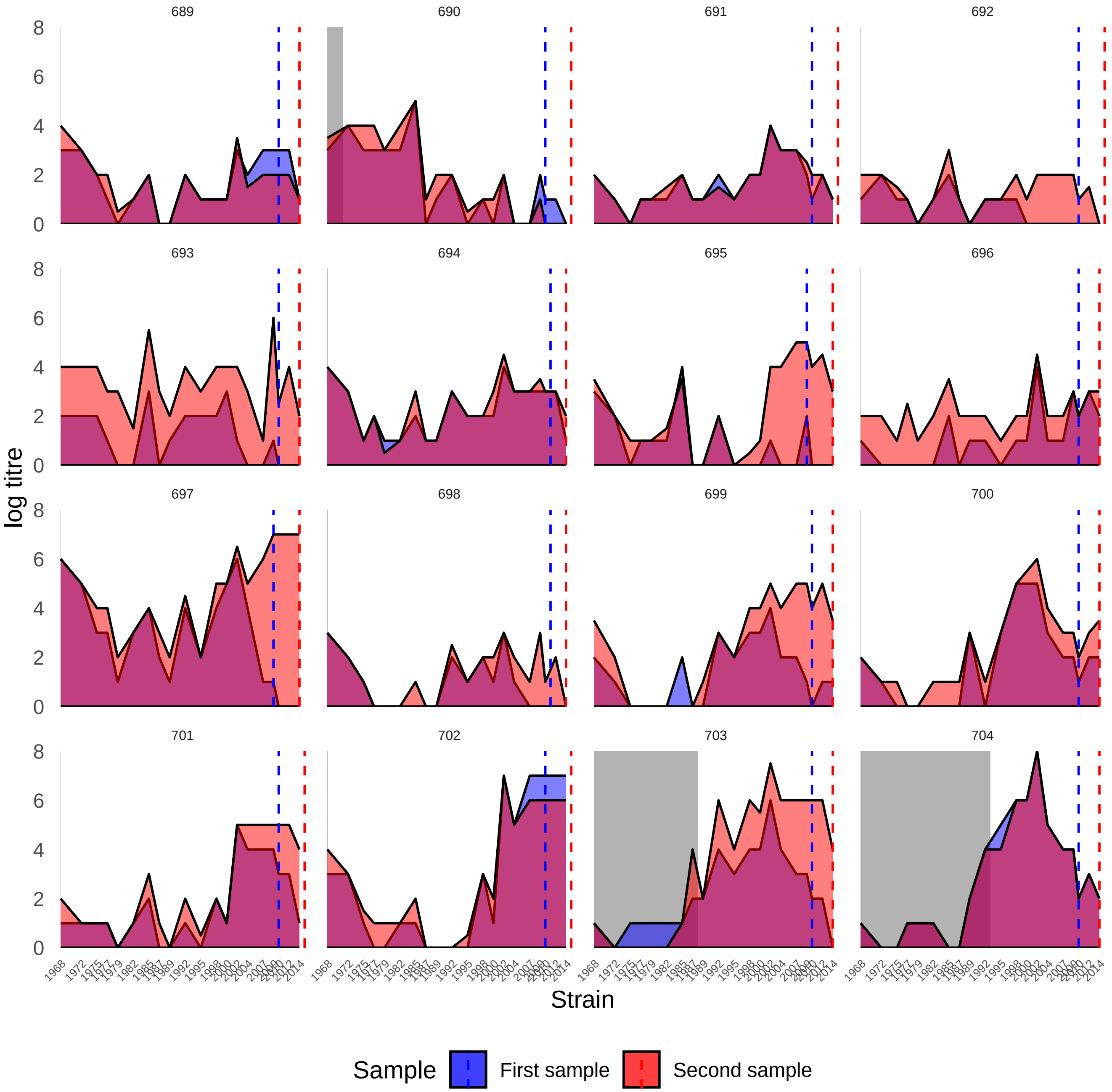

689
690
691
692
693
694
695
696
697
698
699
700
701
702
703
704
log titre
0
2
4
6
8
1968
1972
1975
1977
1979
1982
1985
1987
1989
1992
1995
1998
2000
2002
2004
2007
2009
2010
2012
2014
Strain
Sample
First sample
Second sample

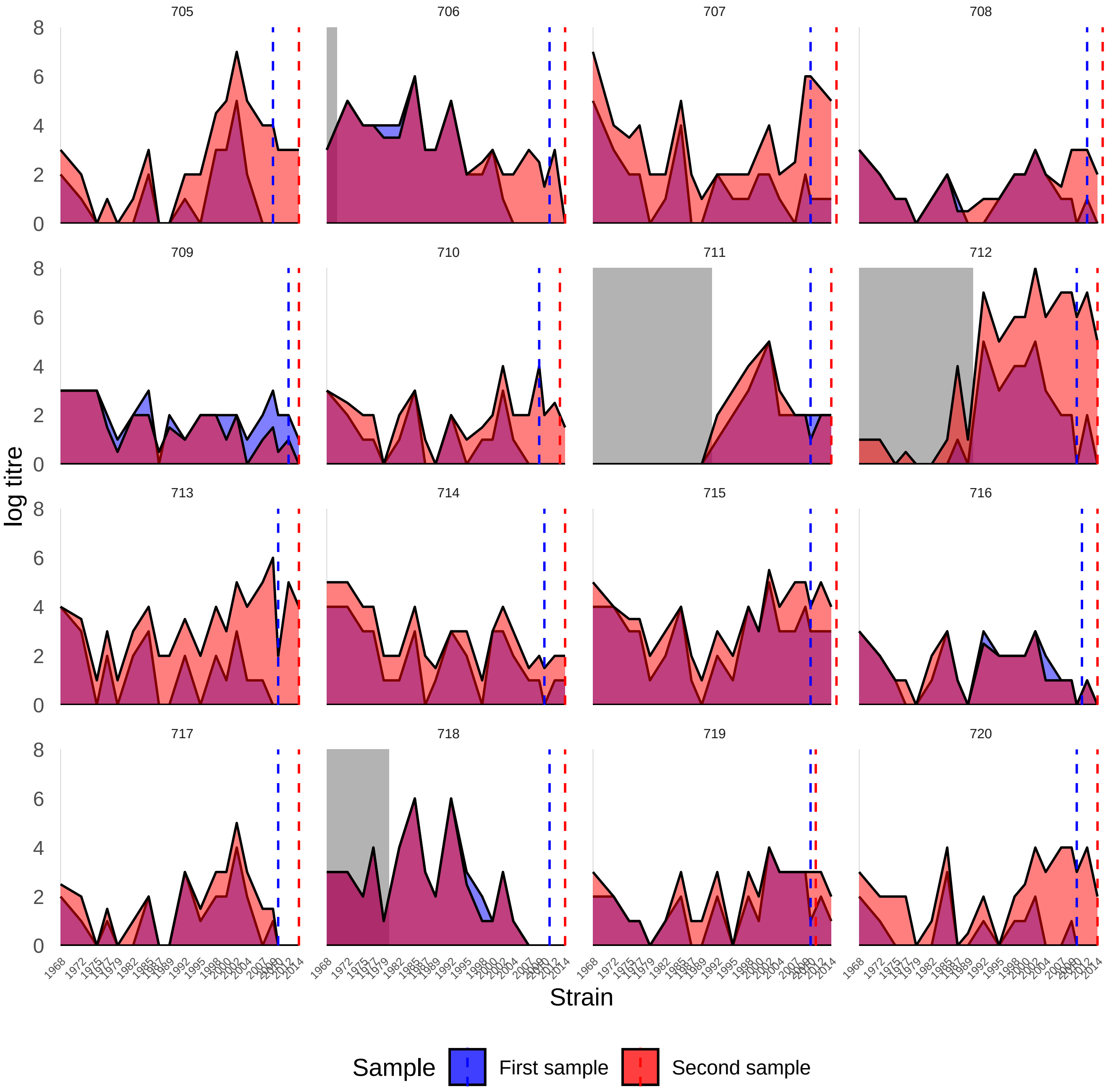
705
706
707
708
709
710
711
712
713
714
715
716
717
718
719
720
log titre
Strain
1968
1972
1975
1977
1979
1982
1985
1987
1989
1992
1995
1998
2000
2002
2004
2007
2009
2010
2012
2014
Sample
First sample
Second sample

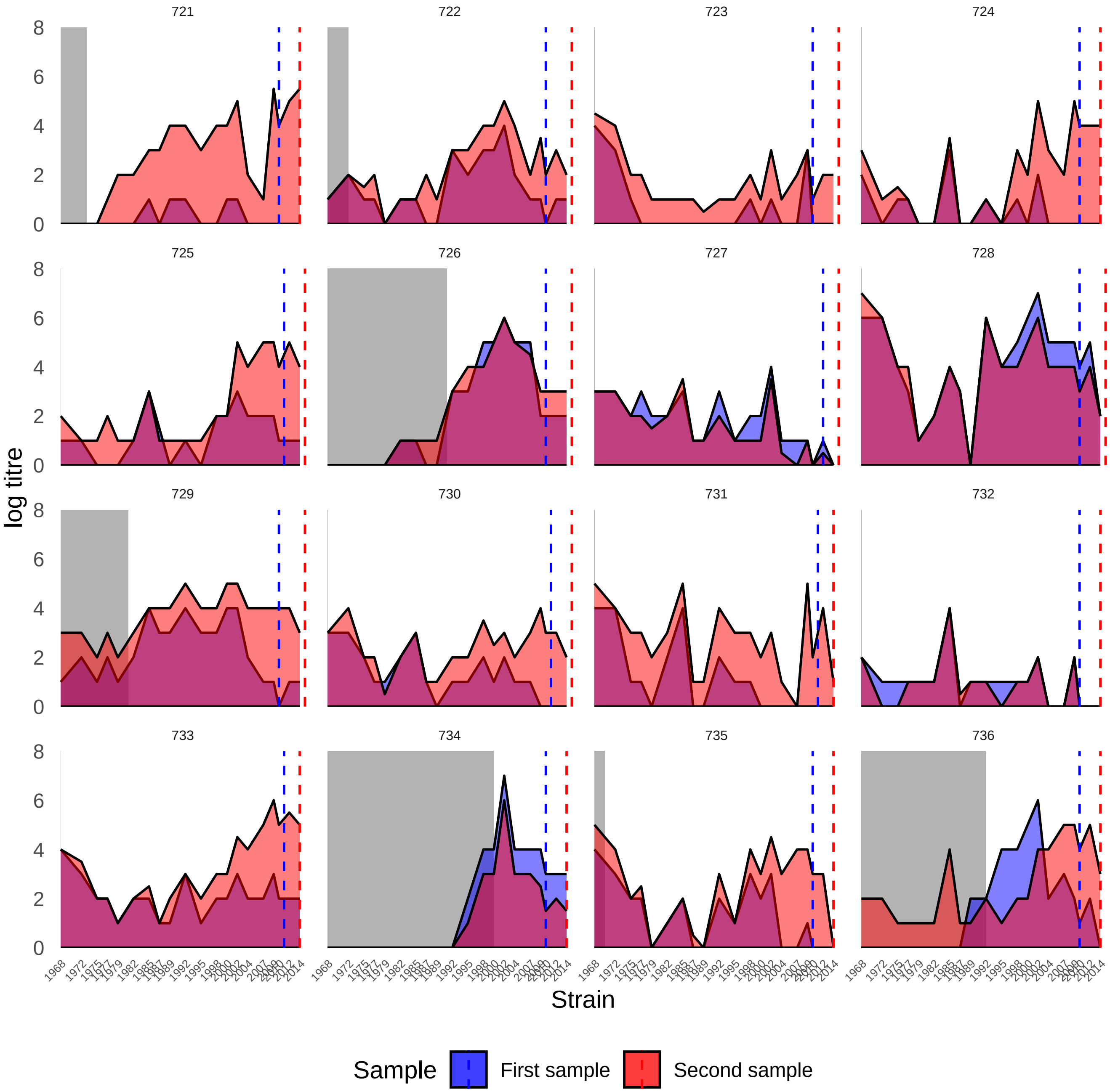

721
722
723
724
725
726
727
728
729
730
731
732
733
734
735
736
log titre
Strain
1968
1972
1975
1977
1979
1982
1985
1987
1989
1992
1995
1998
2000
2002
2004
2007
2009
2010
2012
2014
Sample
First sample
Second sample

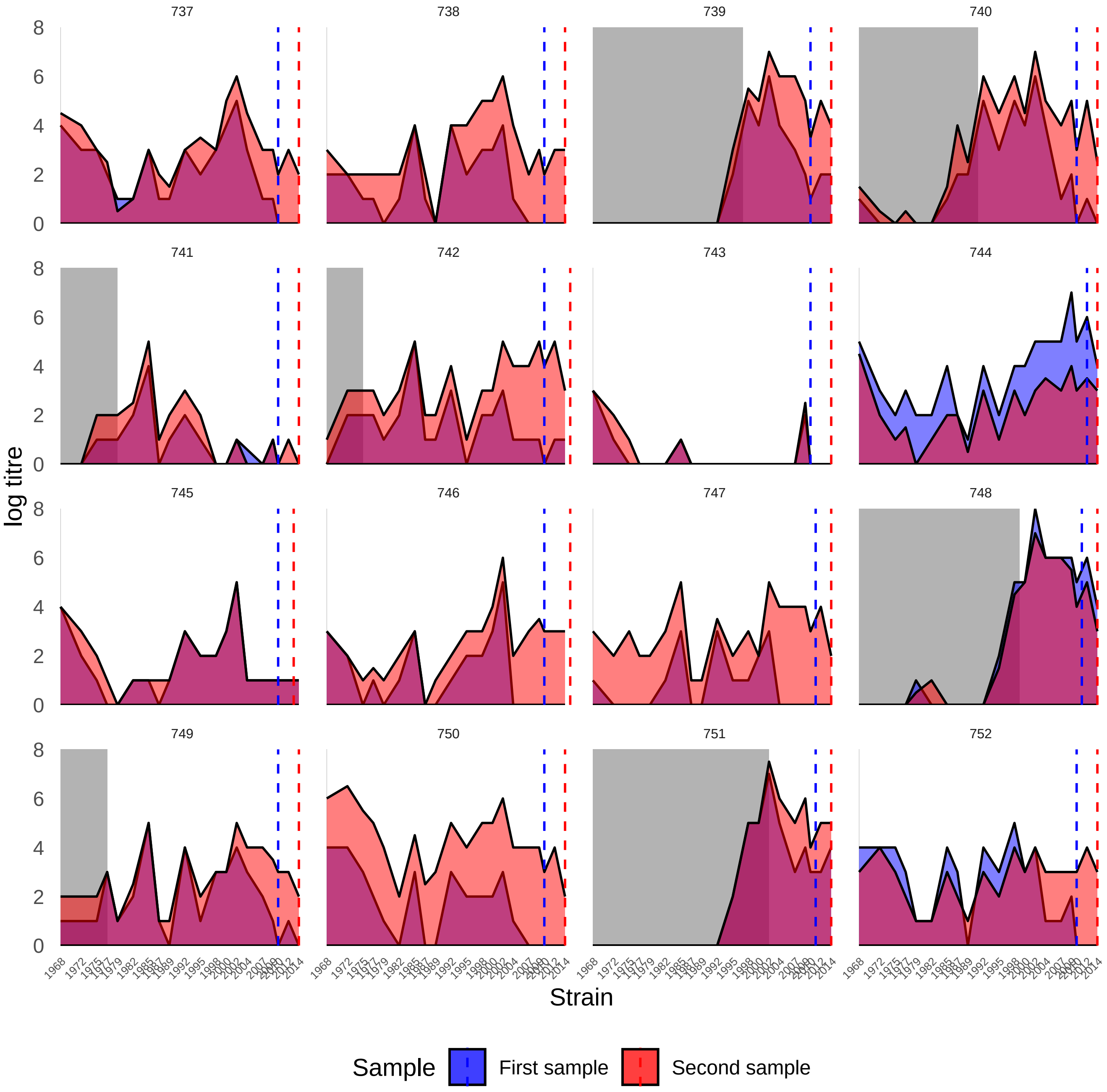
737
738
739
740
741
742
743
744
745
746
747
748
749
750
751
752
log titre
Strain
0
2
4
6
8
1968
1972
1975
1977
1979
1982
1985
1987
1989
1992
1995
1998
2000
2002
2004
2007
2009
2010
2012
2014
Sample
First sample
Second sample

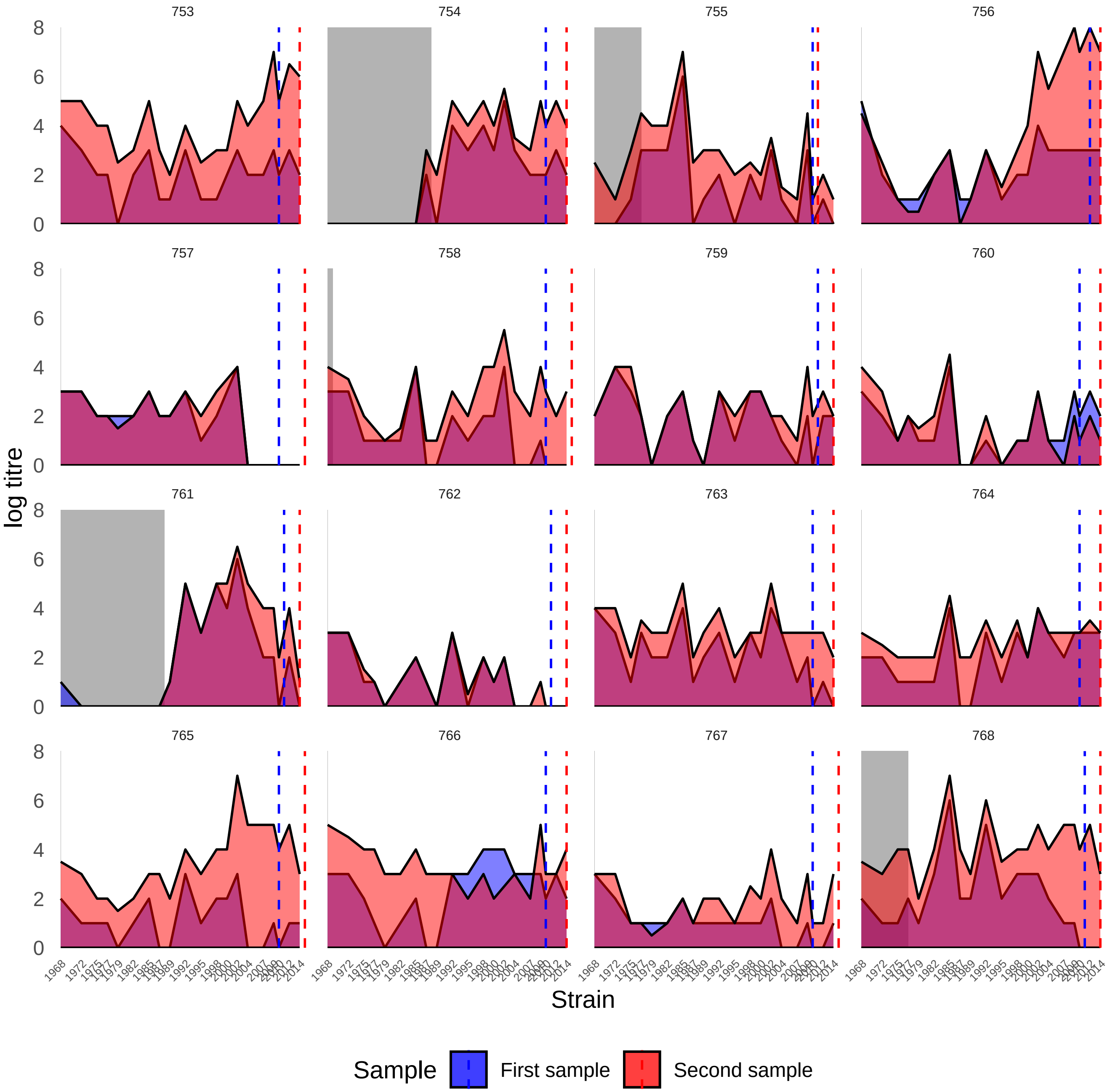
753
754
755
756
757
758
759
760
761
762
763
764
765
766
767
768
log titre
Strain
Sample
First sample
Second sample

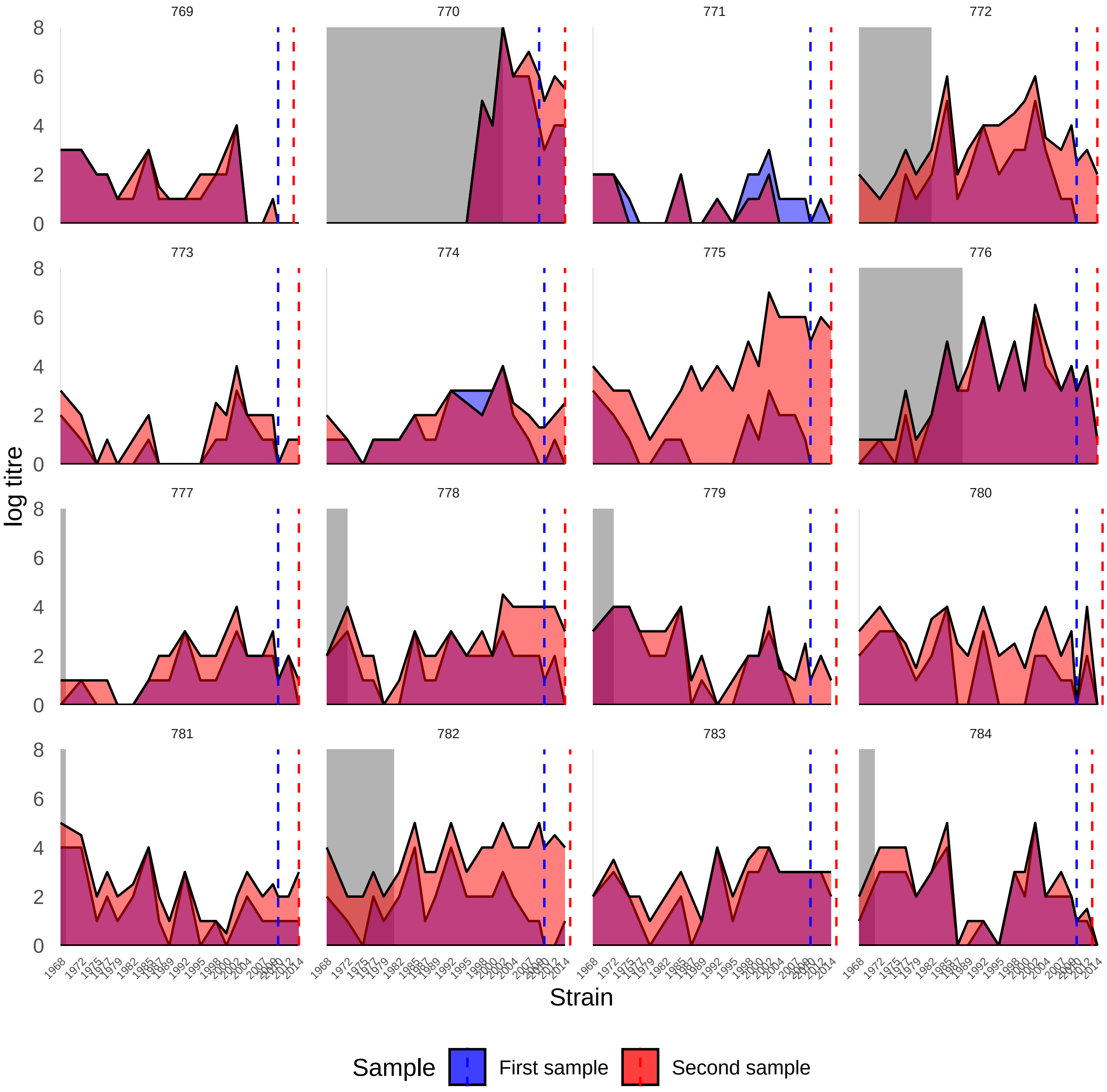

769
770
771
772
773
774
775
776
777
778
779
780
781
782
783
784
log titre
Strain
Sample
First sample
Second sample

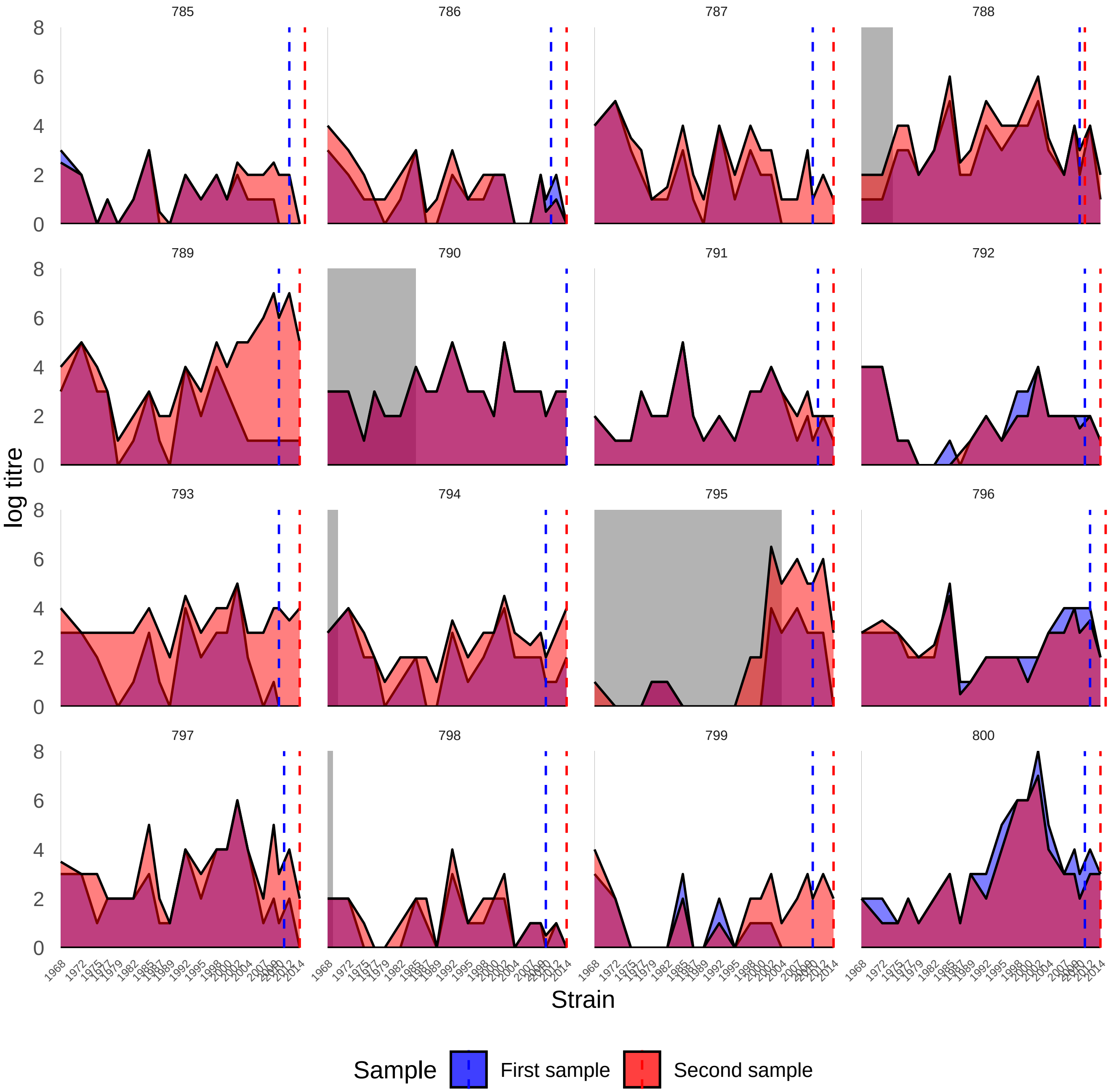
785
786
787
788
789
790
791
792
793
794
795
796
797
798
799
800
log titre
Strain
Sample
First sample
Second sample

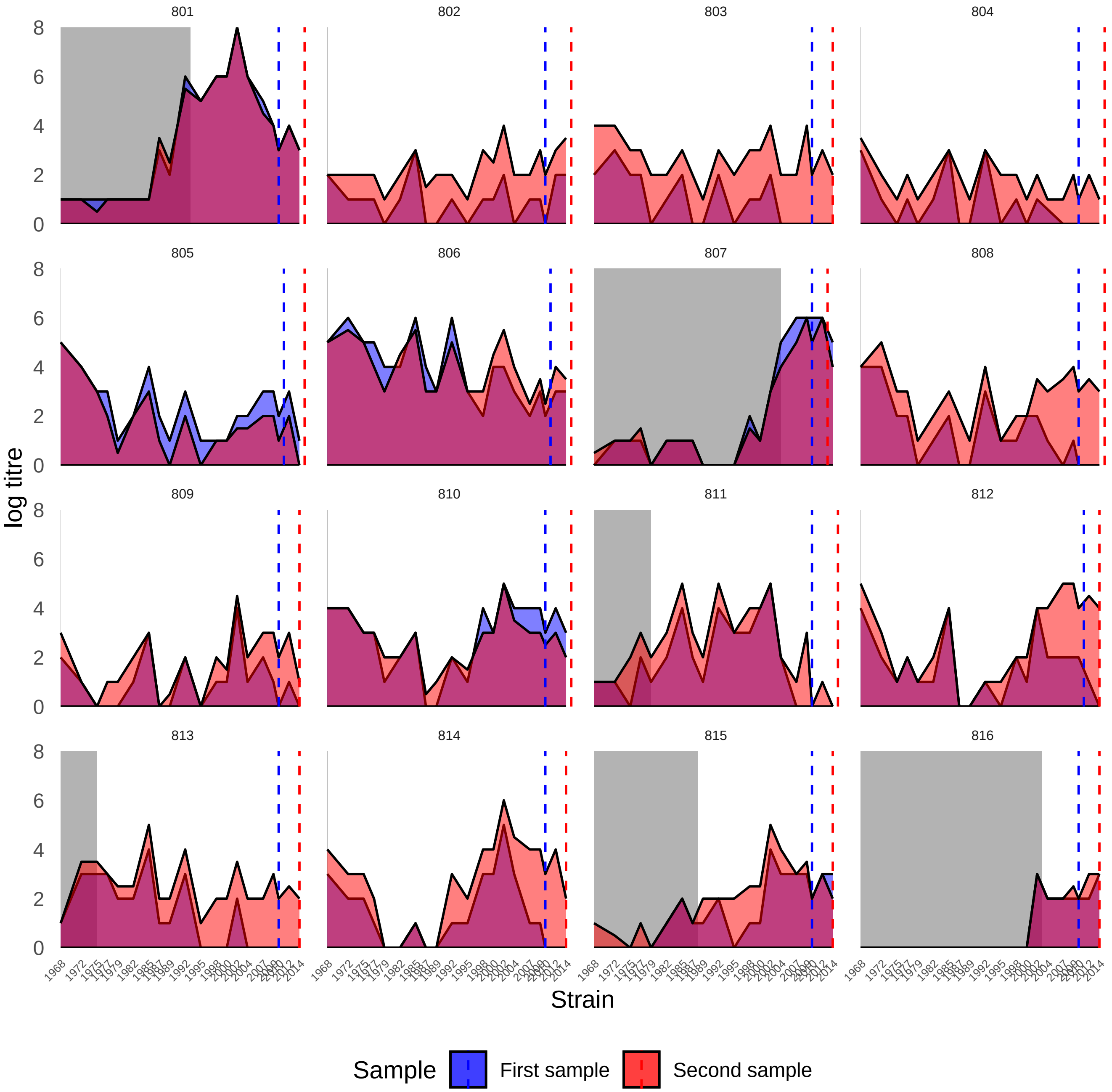

801
802
803
804
805
806
807
808
809
810
811
812
813
814
815
816
log titre
Strain
Sample
First sample
Second sample

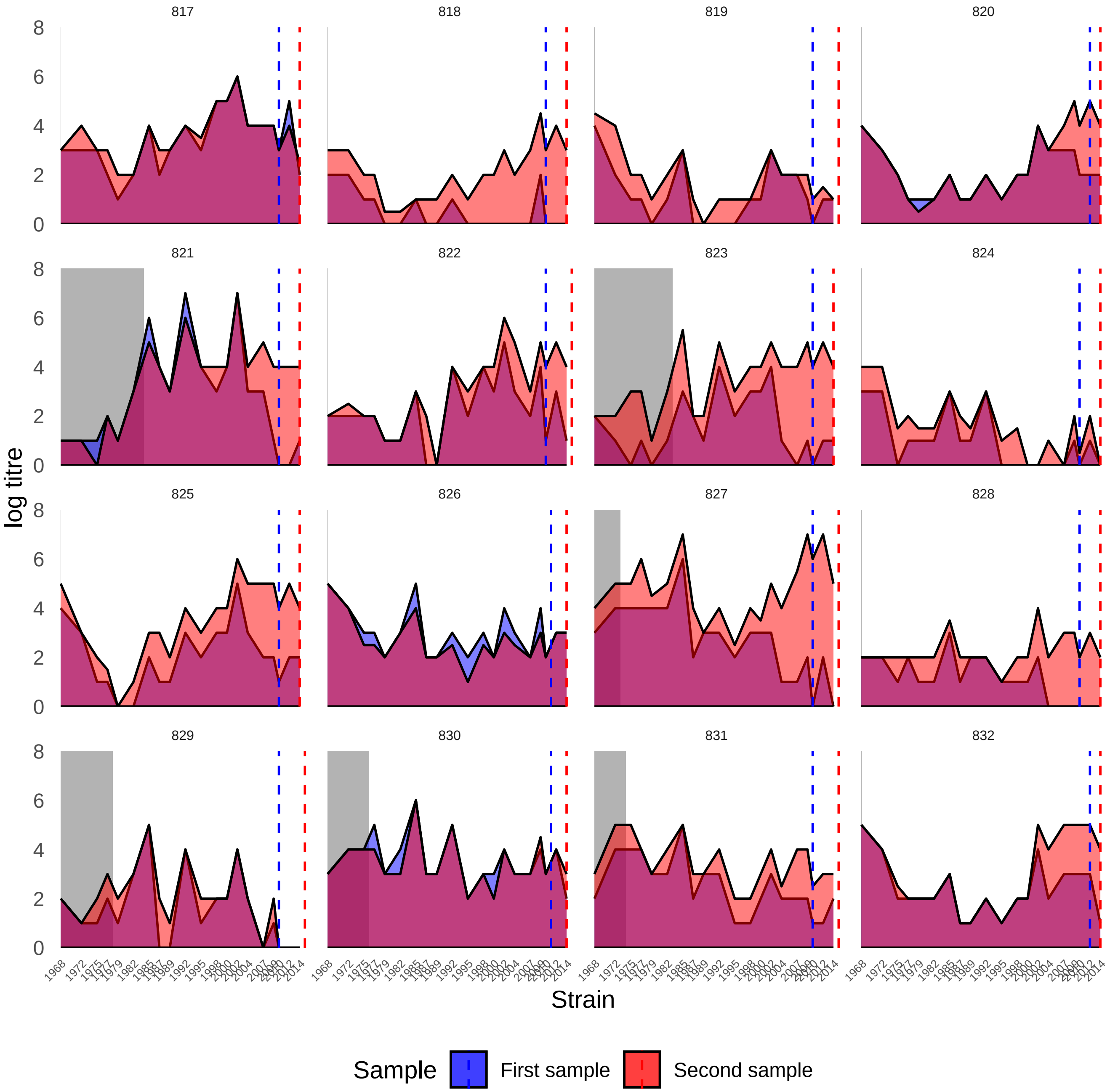
817
818
819
820
821
822
823
824
825
826
827
828
829
830
831
832
log titre
Strain
Sample
First sample
Second sample

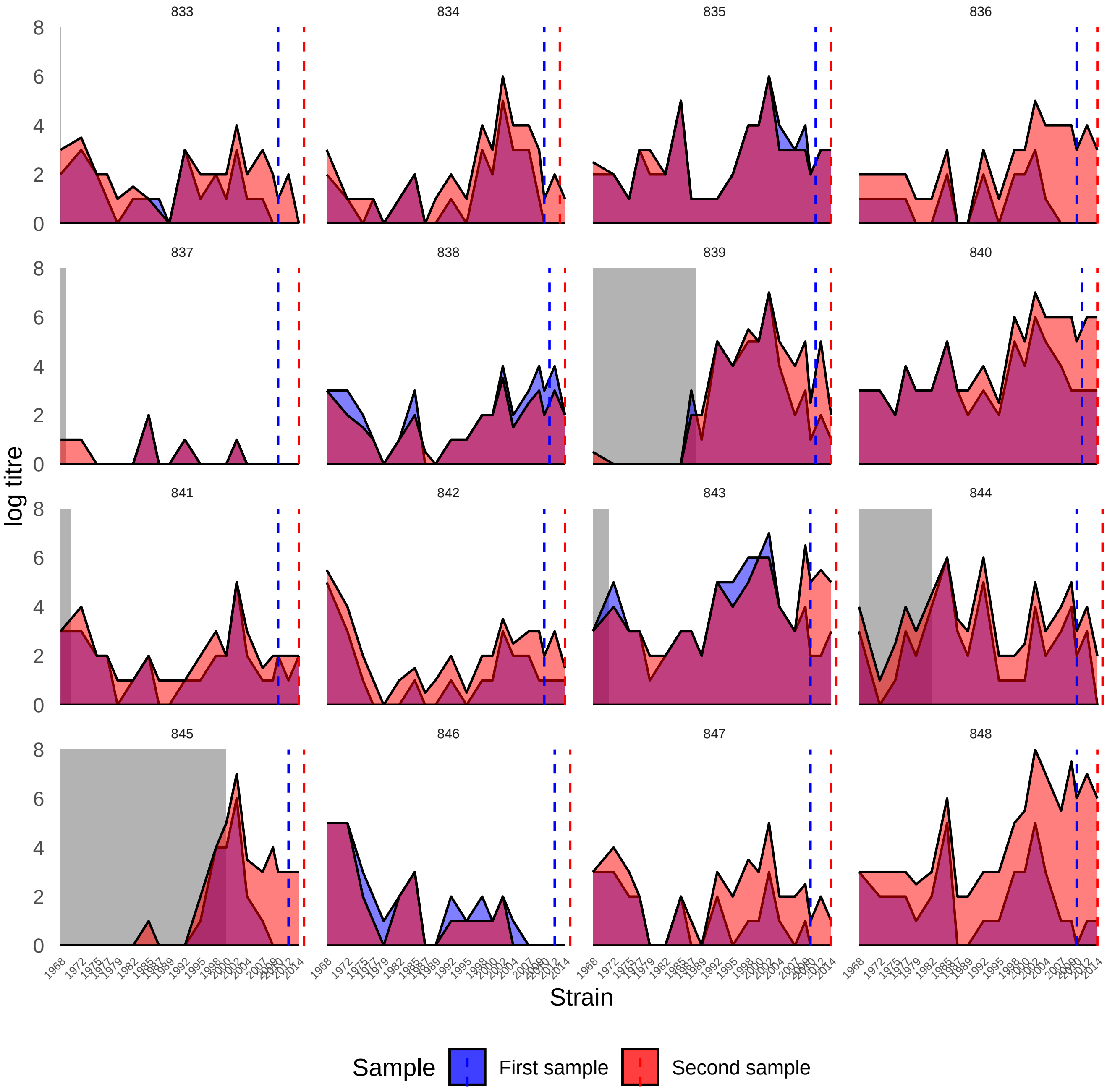

833
834
835
836
837
838
839
840
841
842
843
844
845
846
847
848
log titre
Strain
Sample
First sample
Second sample

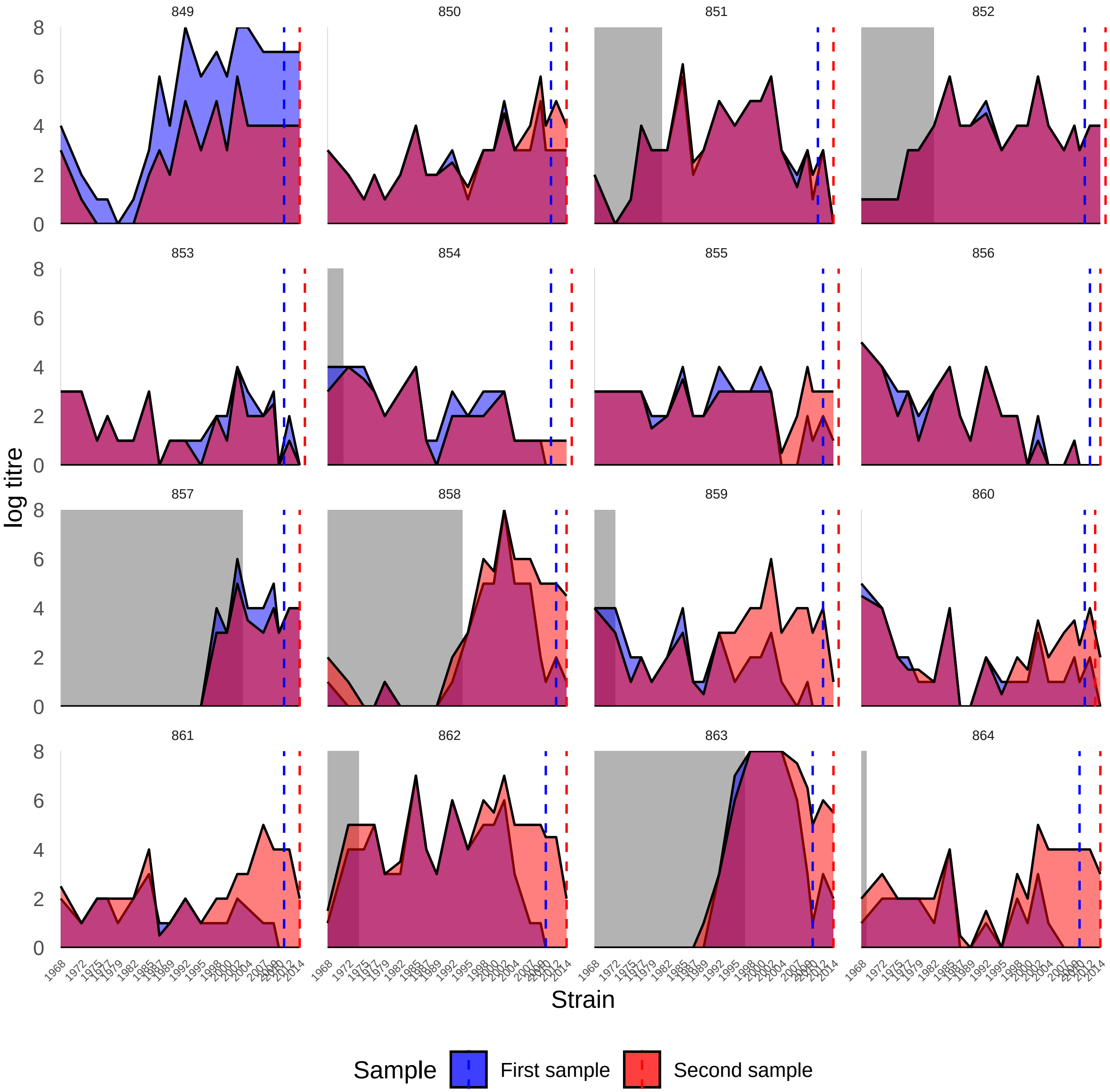
849
850
851
852
853
854
855
856
857
858
859
860
861
862
863
864
log titre
0
2
4
6
8
1968
1972
1975
1977
1979
1982
1985
1987
1989
1992
1995
1998
2000
2002
2004
2007
2009
2010
2012
2014
Strain
Sample
First sample
Second sample

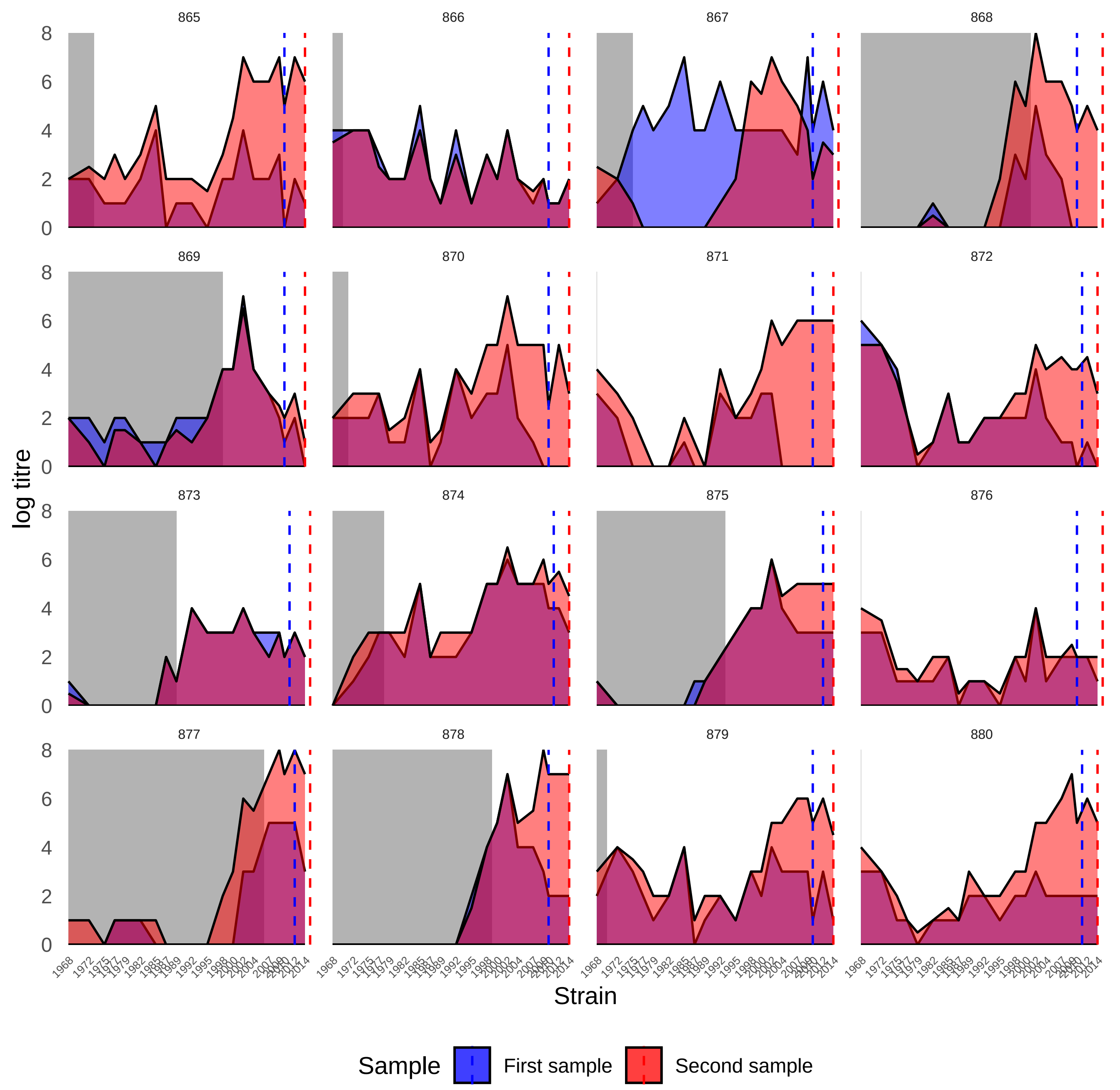
865
866
867
868
869
870
871
872
873
874
875
876
877
878
879
880
log titre
Strain
Sample
First sample
Second sample

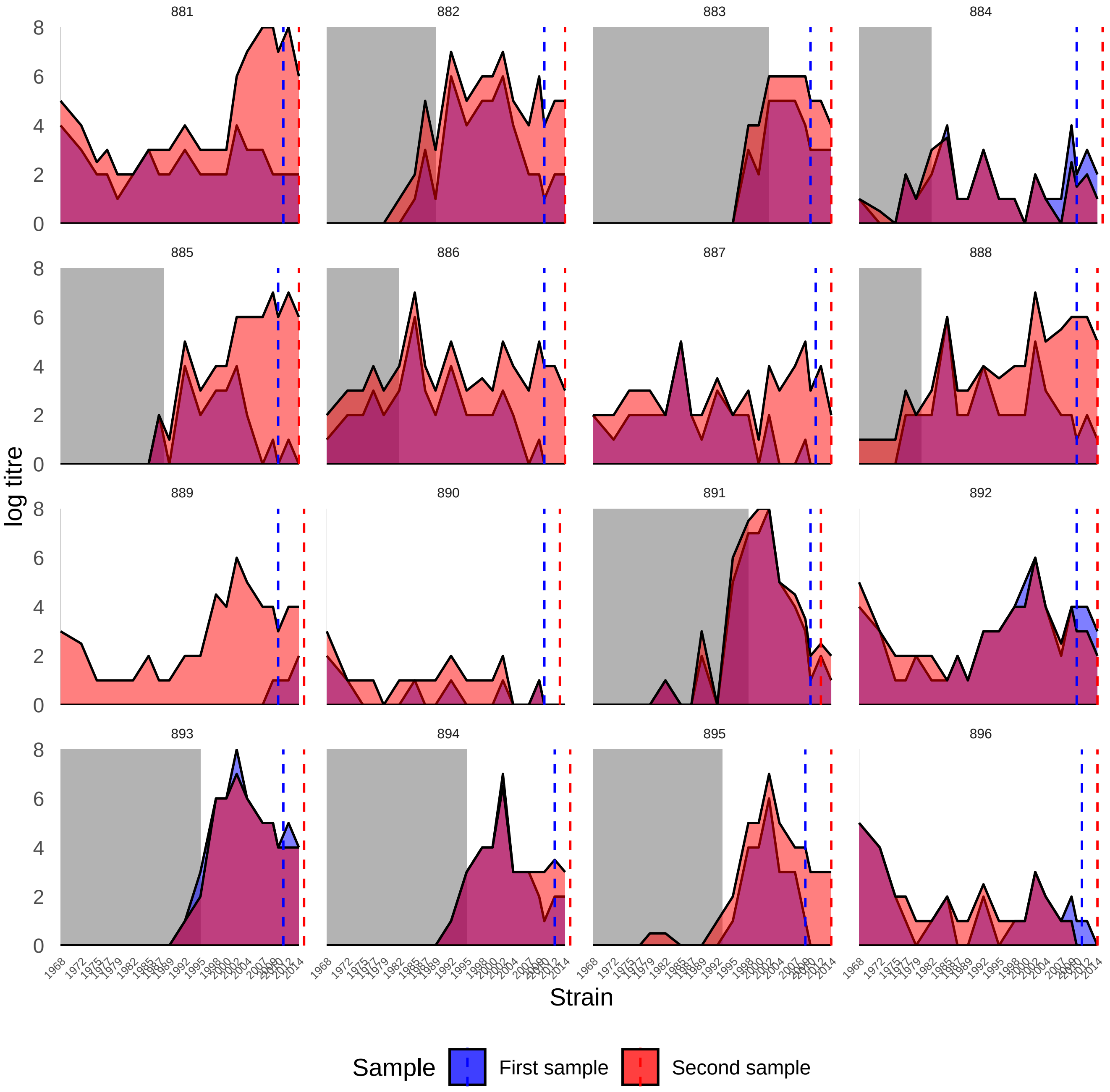
881
882
883
884
885
886
887
888
889
890
891
892
893
894
895
896
log titre
Strain
Sample
First sample
Second sample

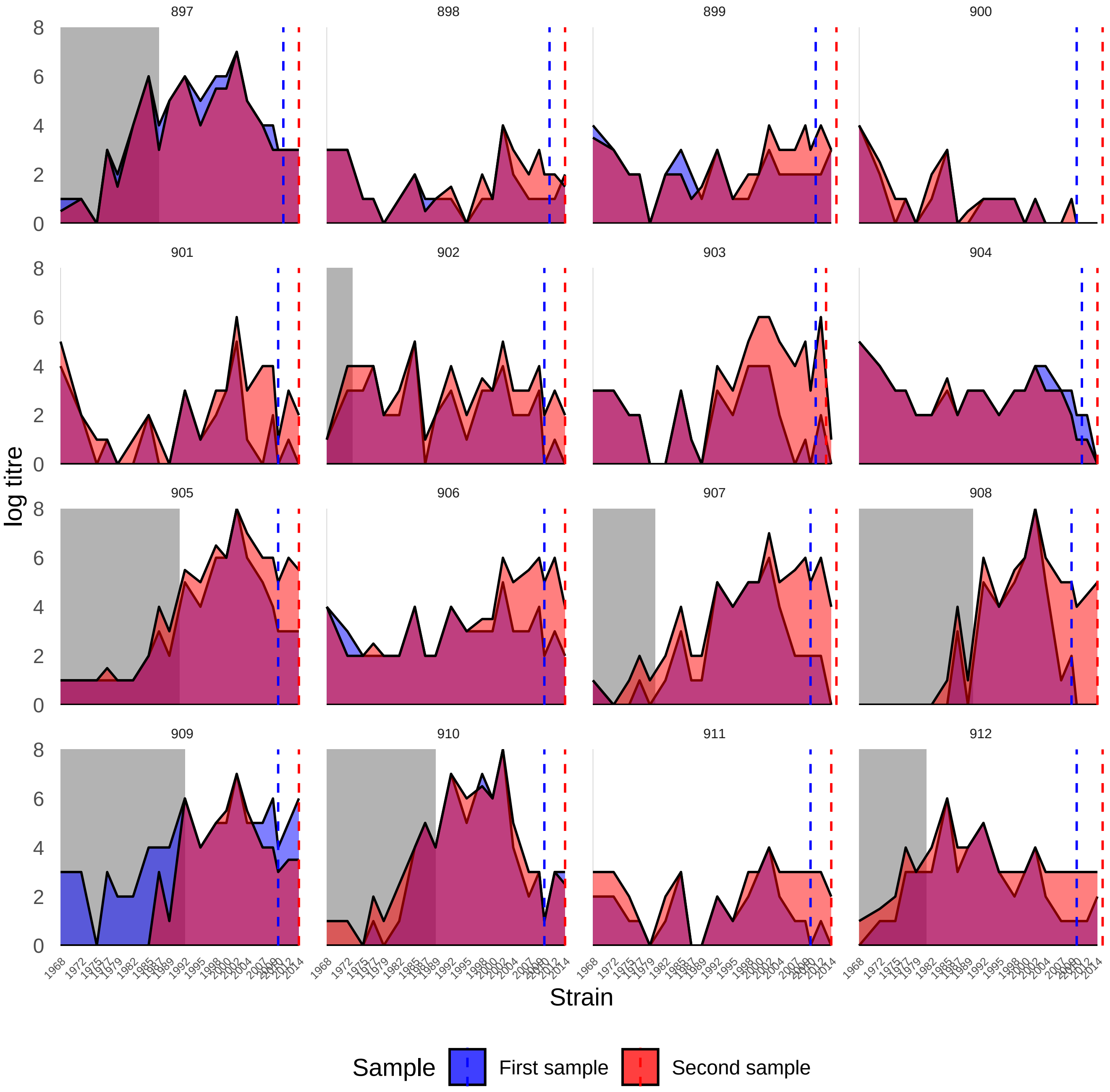
897
898
899
900
901
902
903
904
905
906
907
908
909
910
911
912
log titre
Strain
Sample
First sample
Second sample

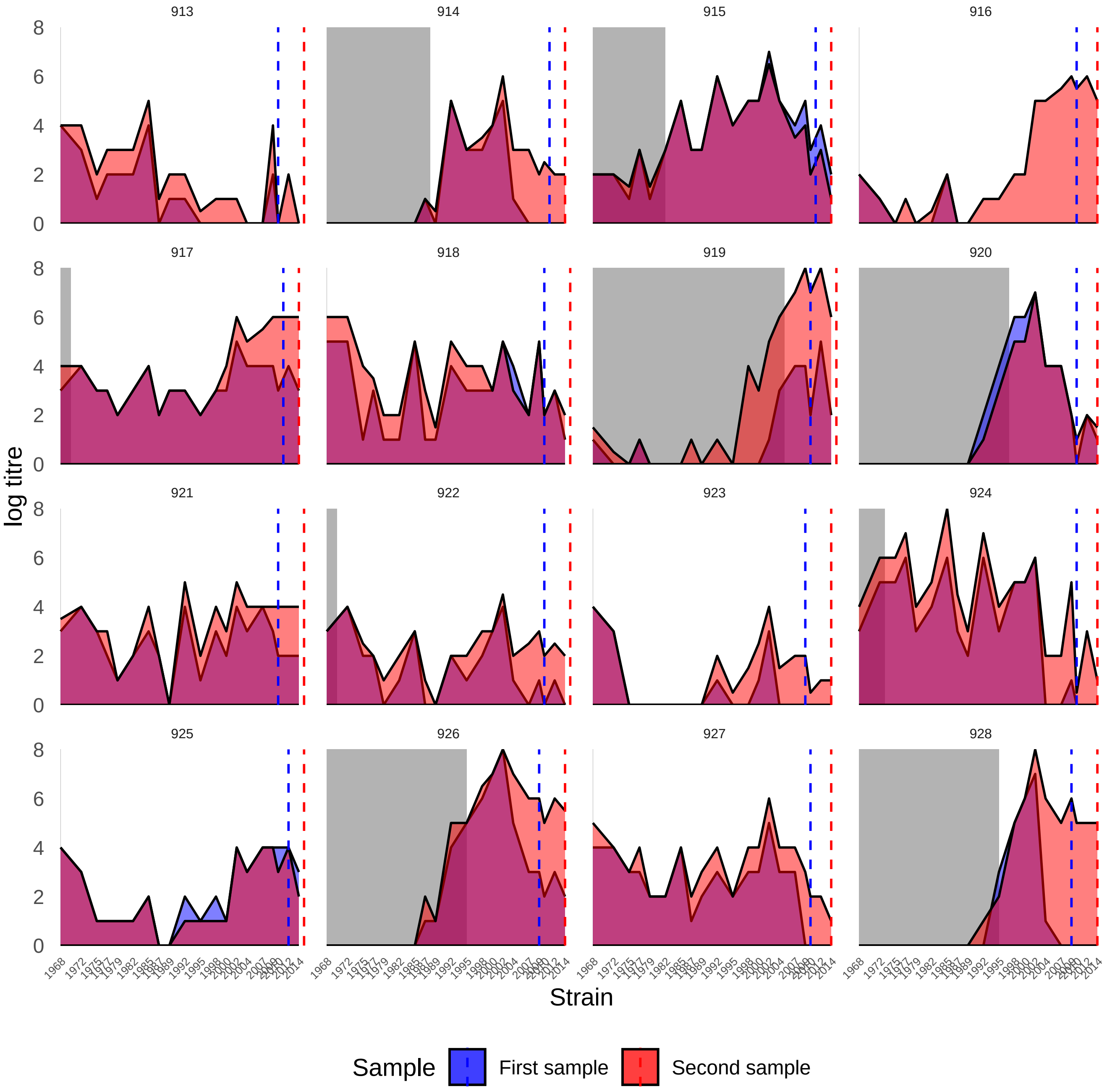
913
914
915
916
917
918
919
920
921
922
923
924
925
926
927
928
log titre
0
2
4
6
8
1968
1972
1975
1977
1979
1982
1985
1987
1989
1992
1995
1998
2000
2002
2004
2007
2009
2010
2012
2014
Strain
Sample
First sample
Second sample

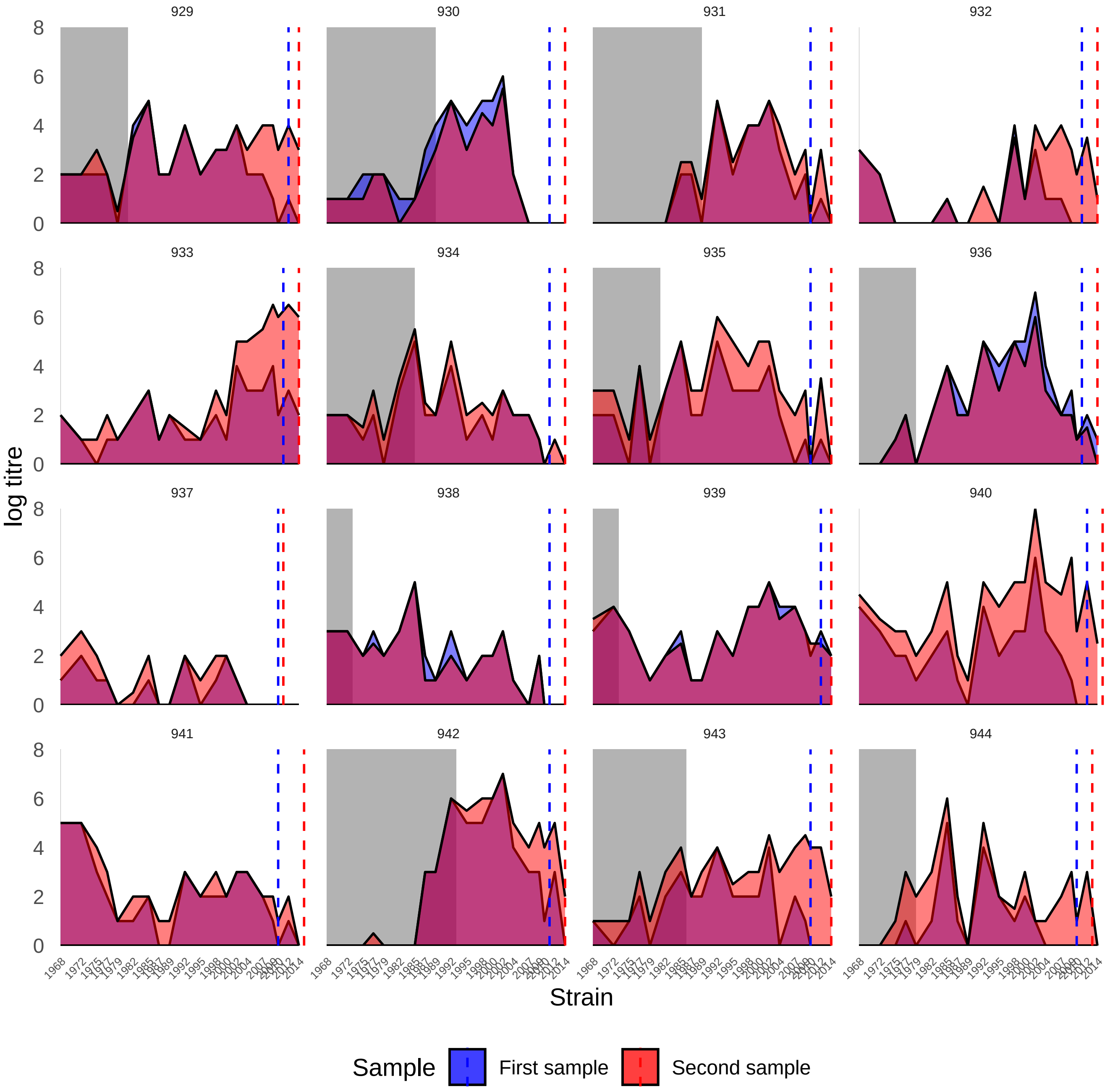
929
930
931
932
933
934
935
936
937
938
939
940
941
942
943
944
log titre
Strain
Sample
First sample
Second sample

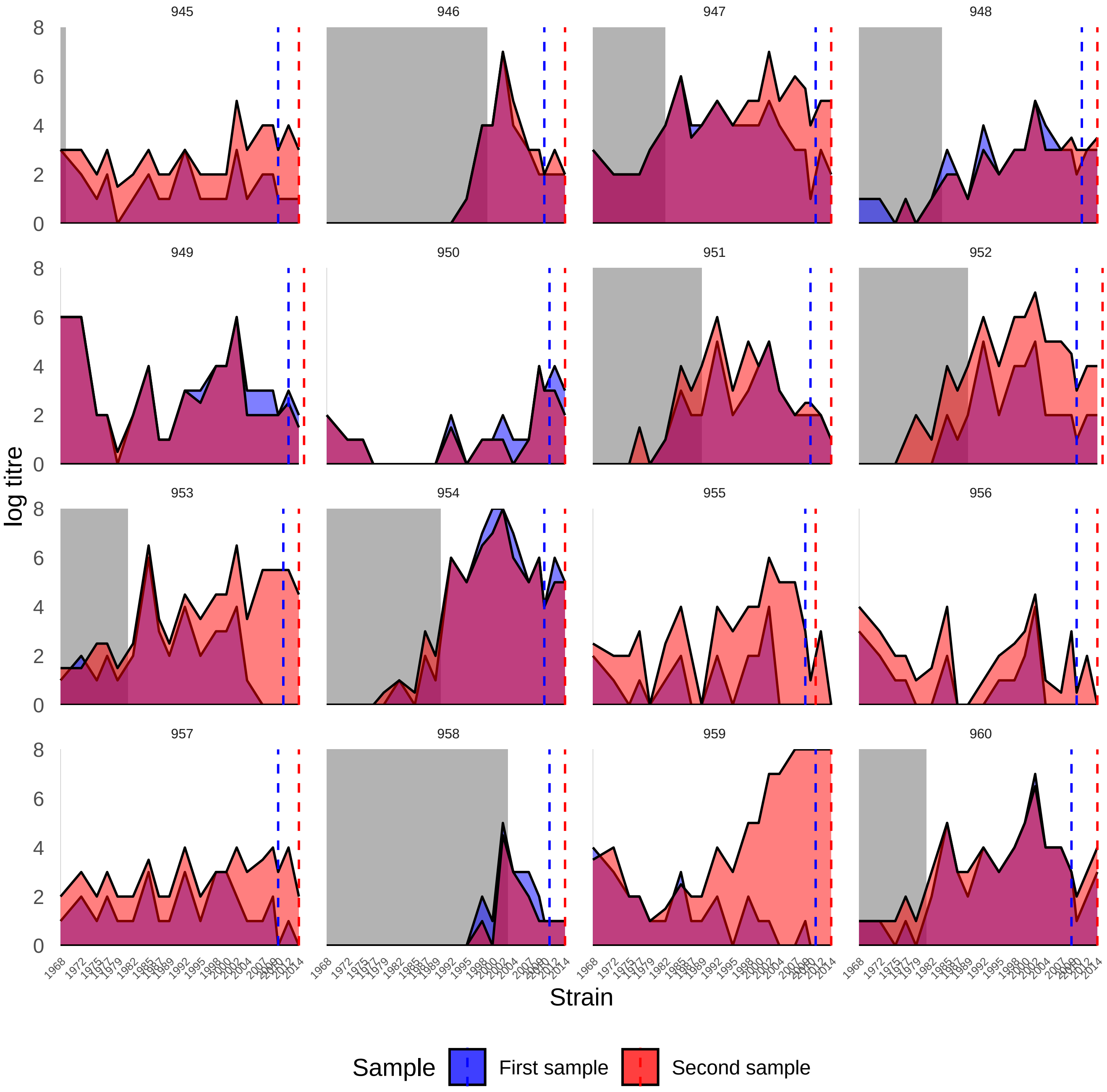
945
946
947
948
949
950
951
952
953
954
955
956
957
958
959
960
log titre
Strain
Sample
First sample
Second sample

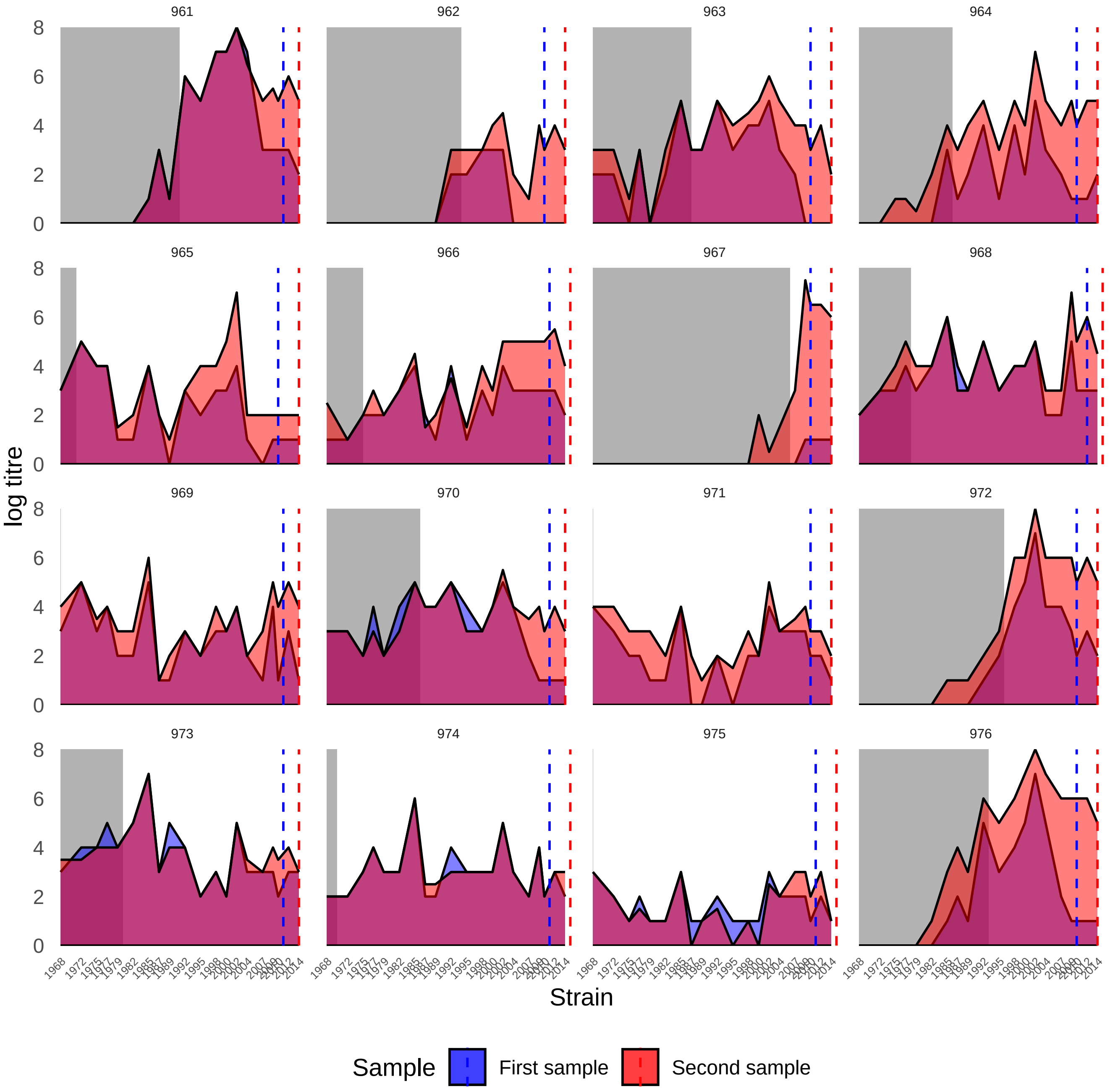

961
962
963
964
965
966
967
968
969
970
971
972
973
974
975
976
log titre
Strain
Sample
First sample
Second sample

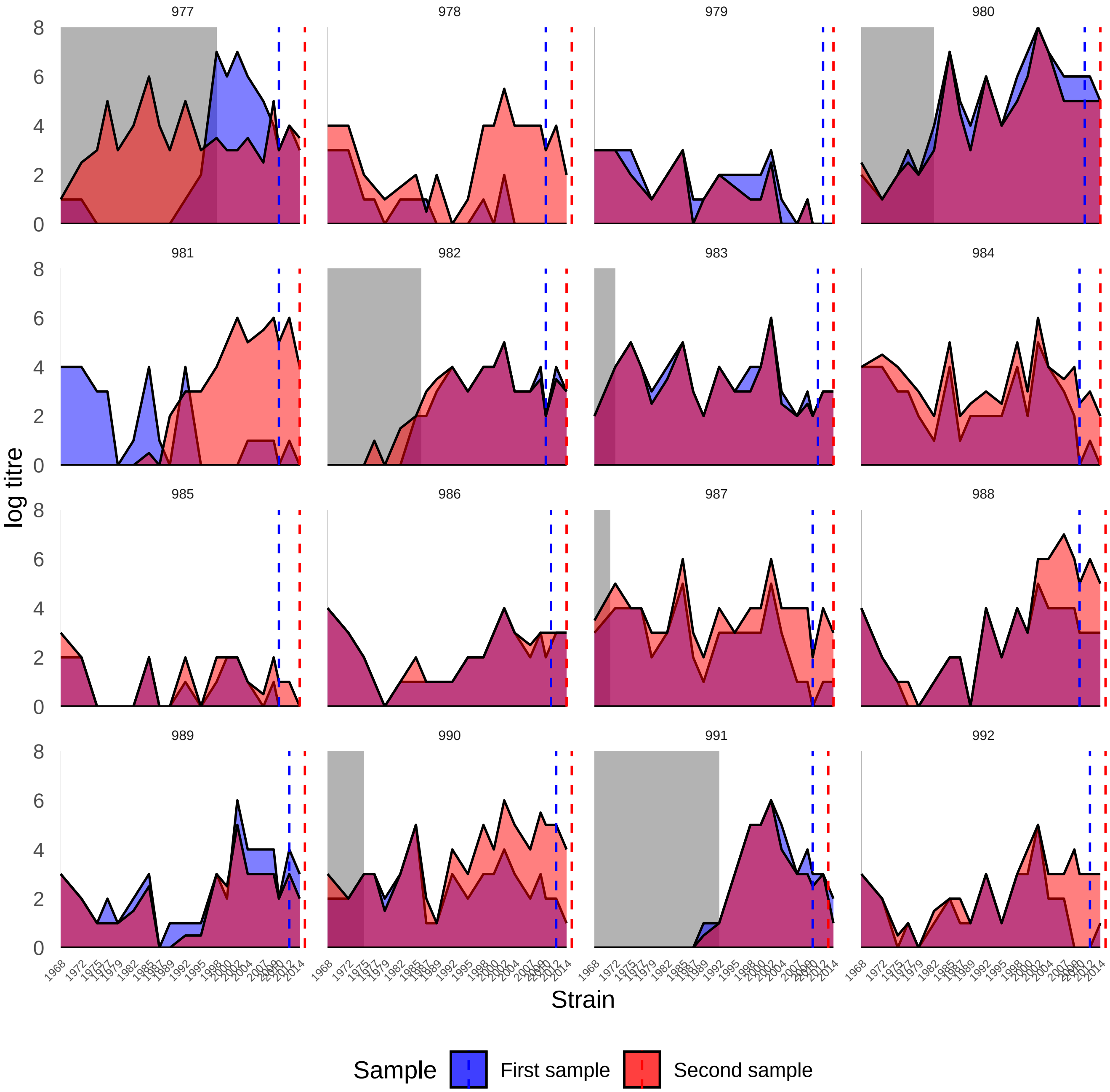

977
978
979
980
981
982
983
984
985
986
987
988
989
990
991
992
log titre
Strain
Sample
First sample
Second sample

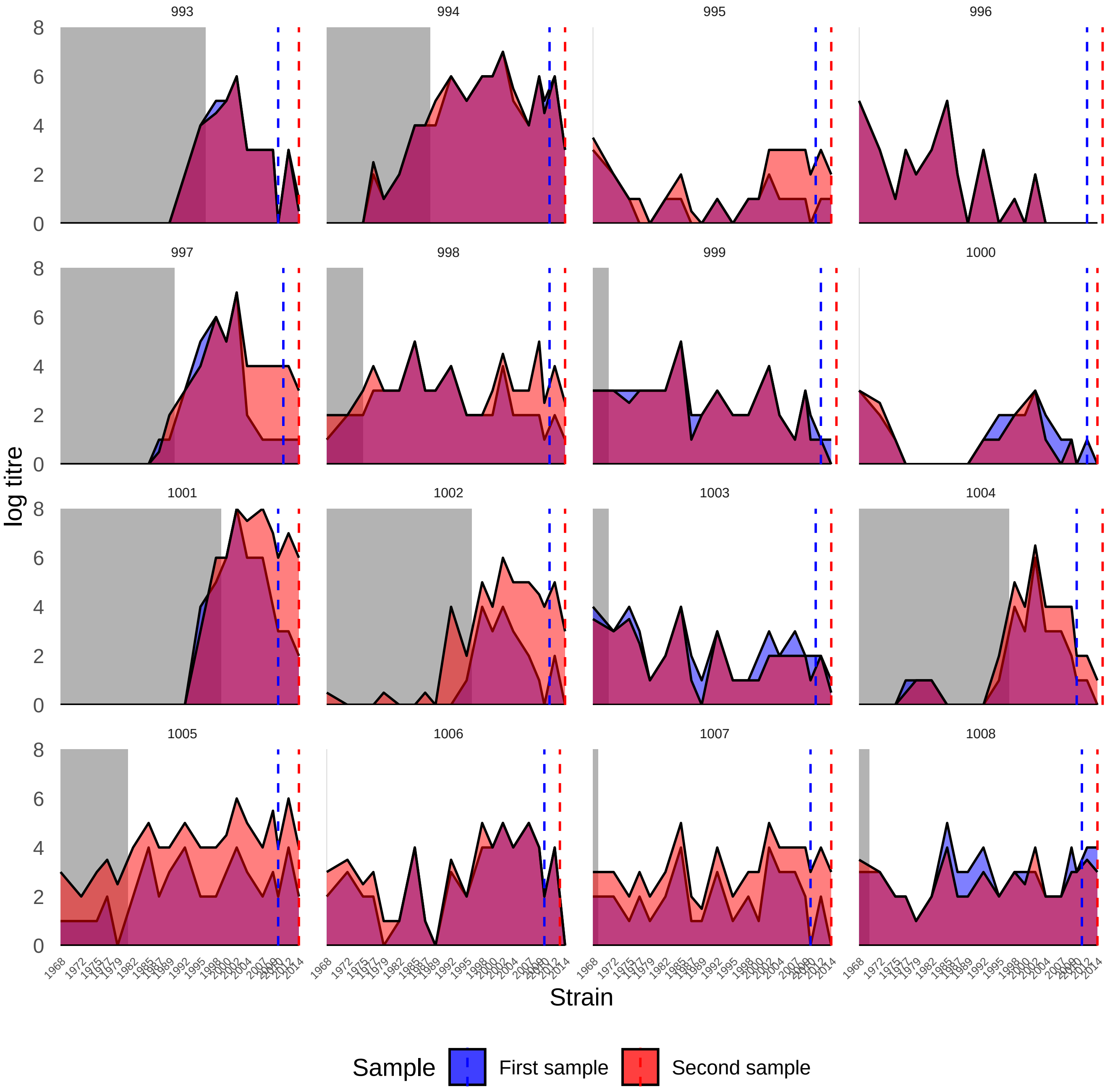
993
994
995
996
997
998
999
1000
1001
1002
1003
1004
1005
1006
1007
1008
log titre
Strain
0
2
4
6
8
1968
1972
1975
1977
1979
1982
1985
1987
1989
1992
1995
1998
2000
2002
2004
2007
2009
2010
2012
2014
Sample
First sample
Second sample

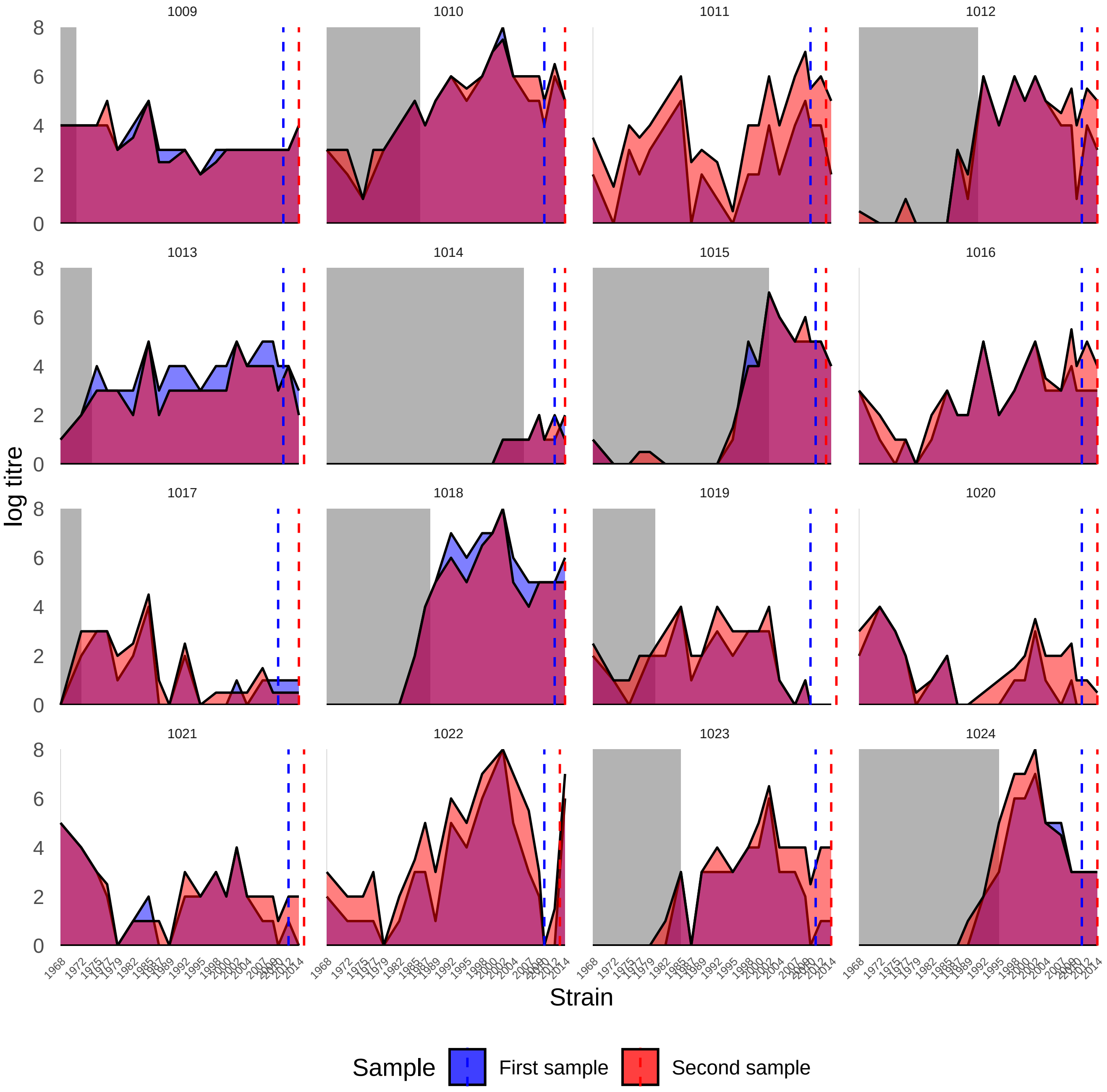

1009
1010
1011
1012
1013
1014
1015
1016
1017
1018
1019
1020
1021
1022
1023
1024
log titre
Strain
Sample
First sample
Second sample

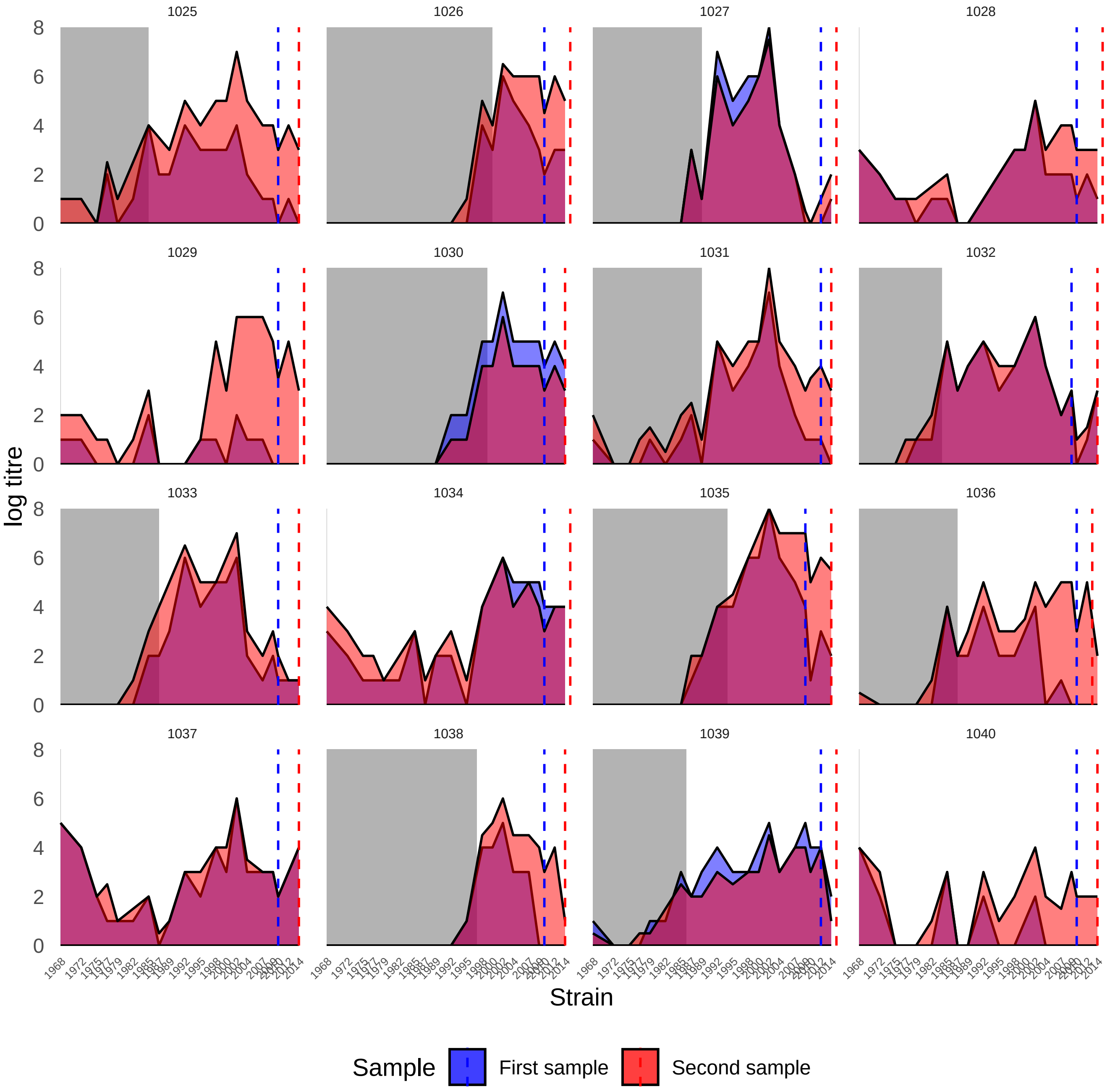

1025
1026
1027
1028
1029
1030
1031
1032
1033
1034
1035
1036
1037
1038
1039
1040
log titre
Strain
Sample
First sample
Second sample

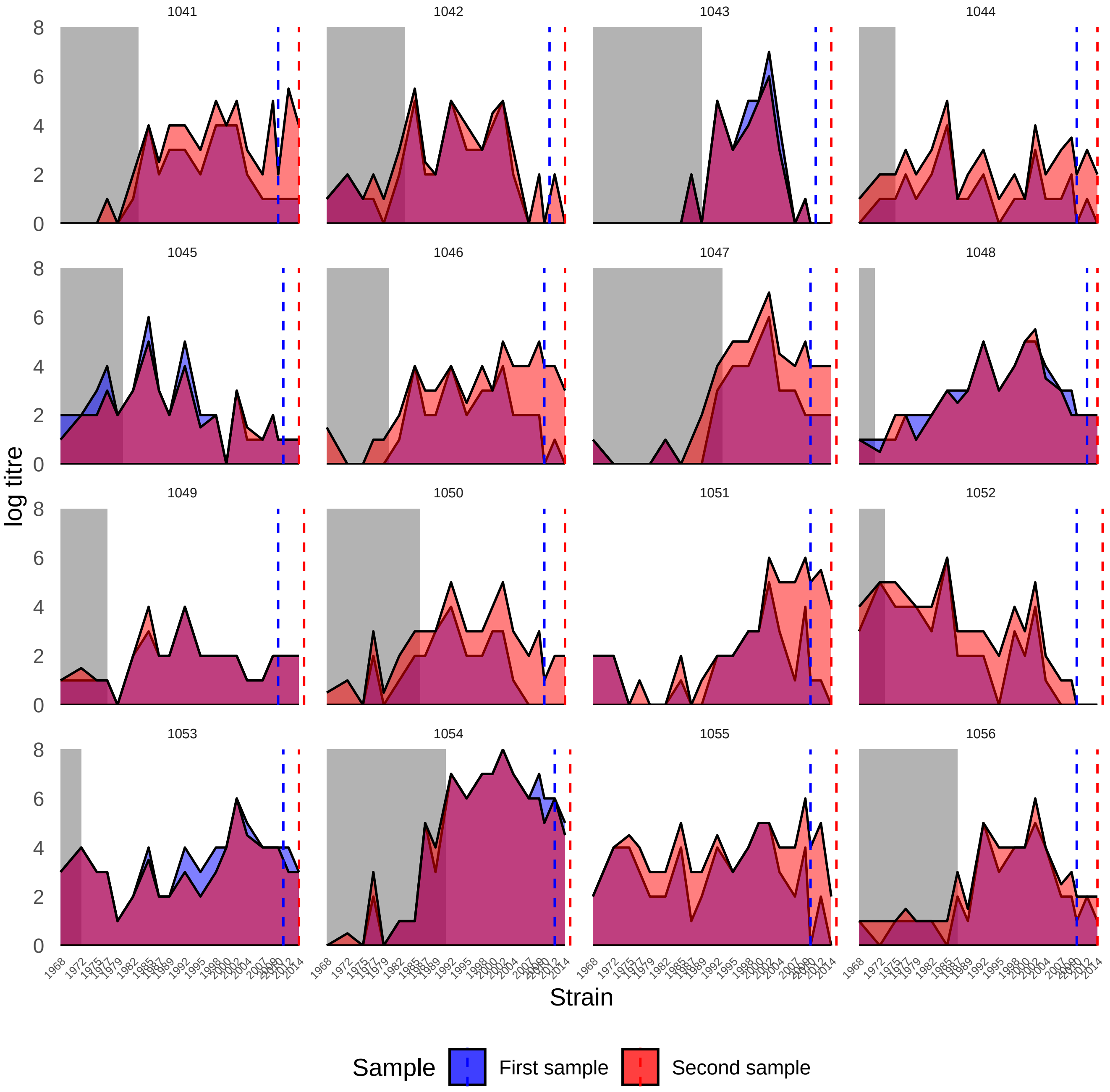

1041
1042
1043
1044
1045
1046
1047
1048
1049
1050
1051
1052
1053
1054
1055
1056
log titre
Strain
Sample
First sample
Second sample

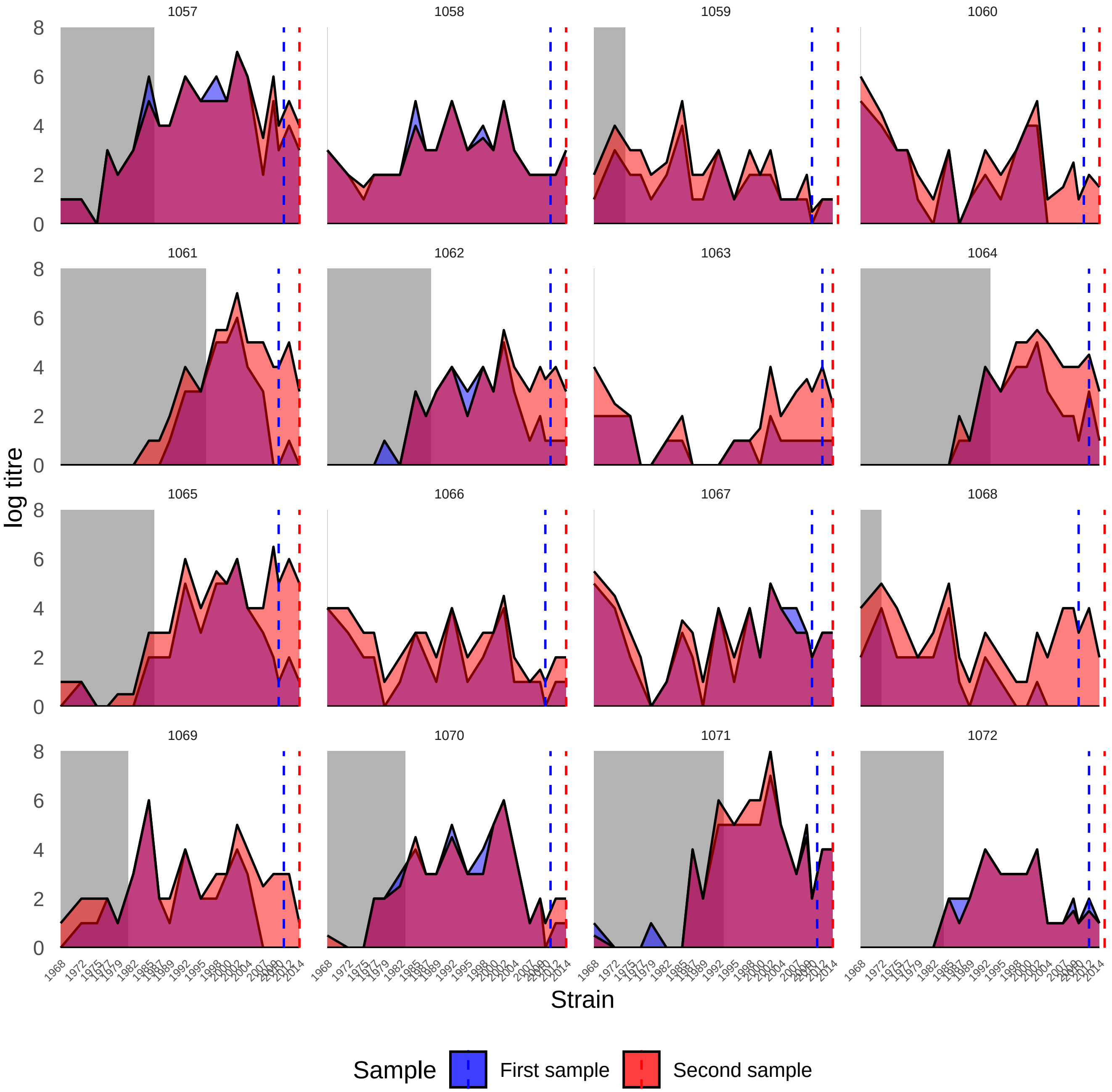

1057
1058
1059
1060
1061
1062
1063
1064
1065
1066
1067
1068
1069
1070
1071
1072
log titre
Strain
Sample
First sample
Second sample

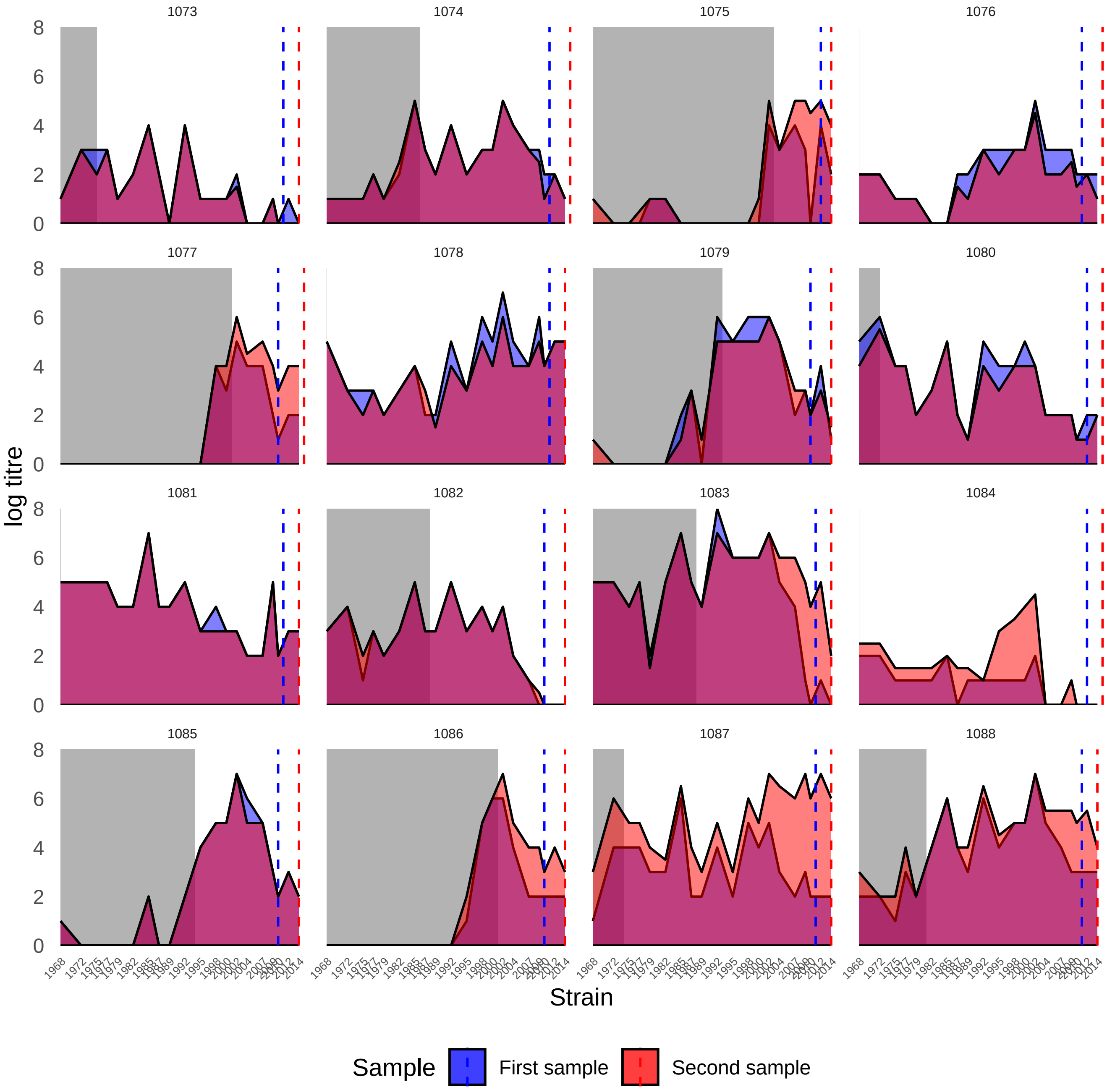

1073
1074
1075
1076
1077
1078
1079
1080
1081
1082
1083
1084
1085
1086
1087
1088
log titre
Strain
Sample
First sample
Second sample

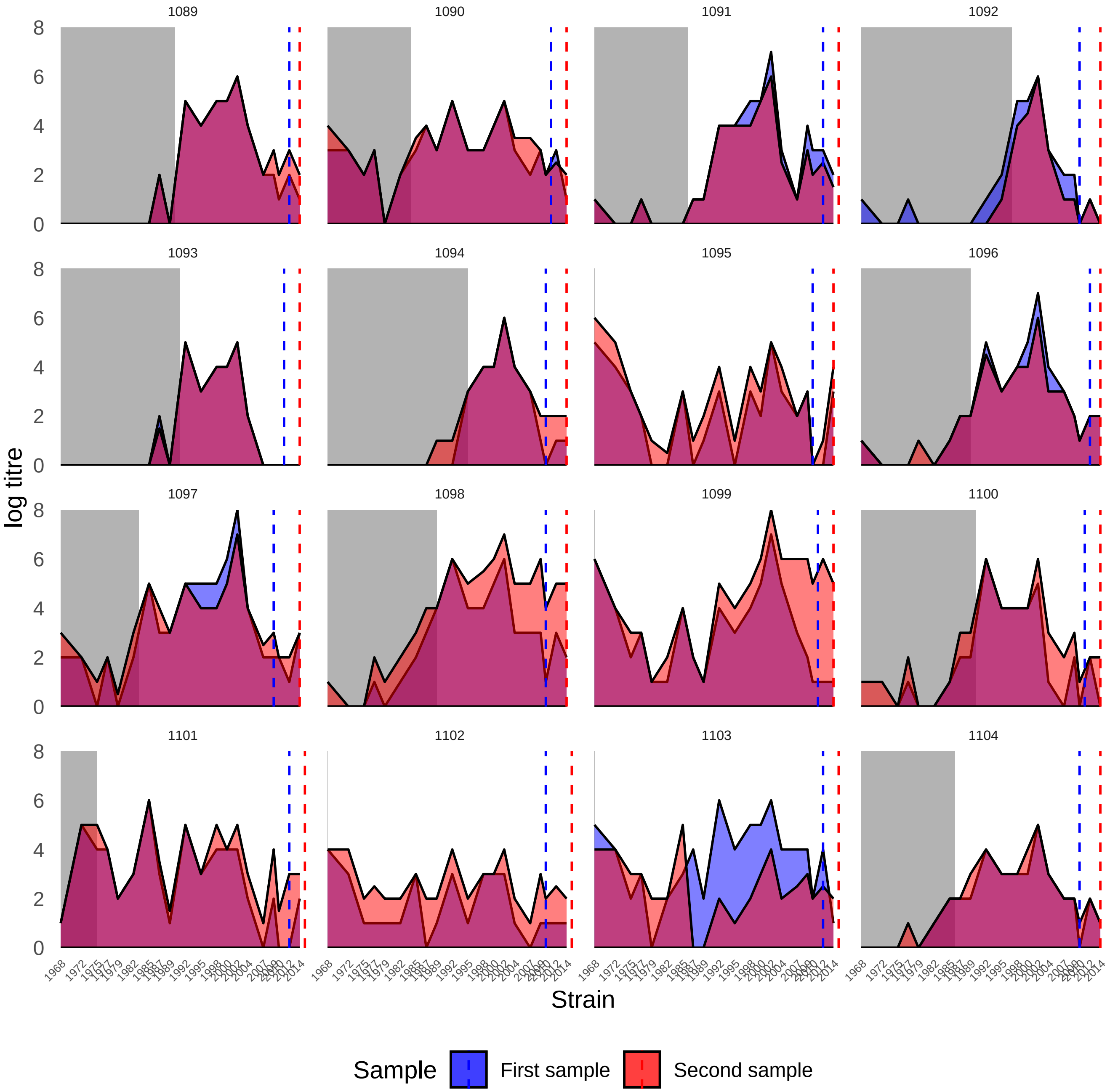
1089
1090
1091
1092
1093
1094
1095
1096
1097
1098
1099
1100
1101
1102
1103
1104
log titre
0
2
4
6
8
1968
1972
1975
1977
1979
1982
1985
1987
1989
1992
1995
1998
2000
2002
2004
2007
2009
2010
2012
2014
Strain
Sample
First sample
Second sample

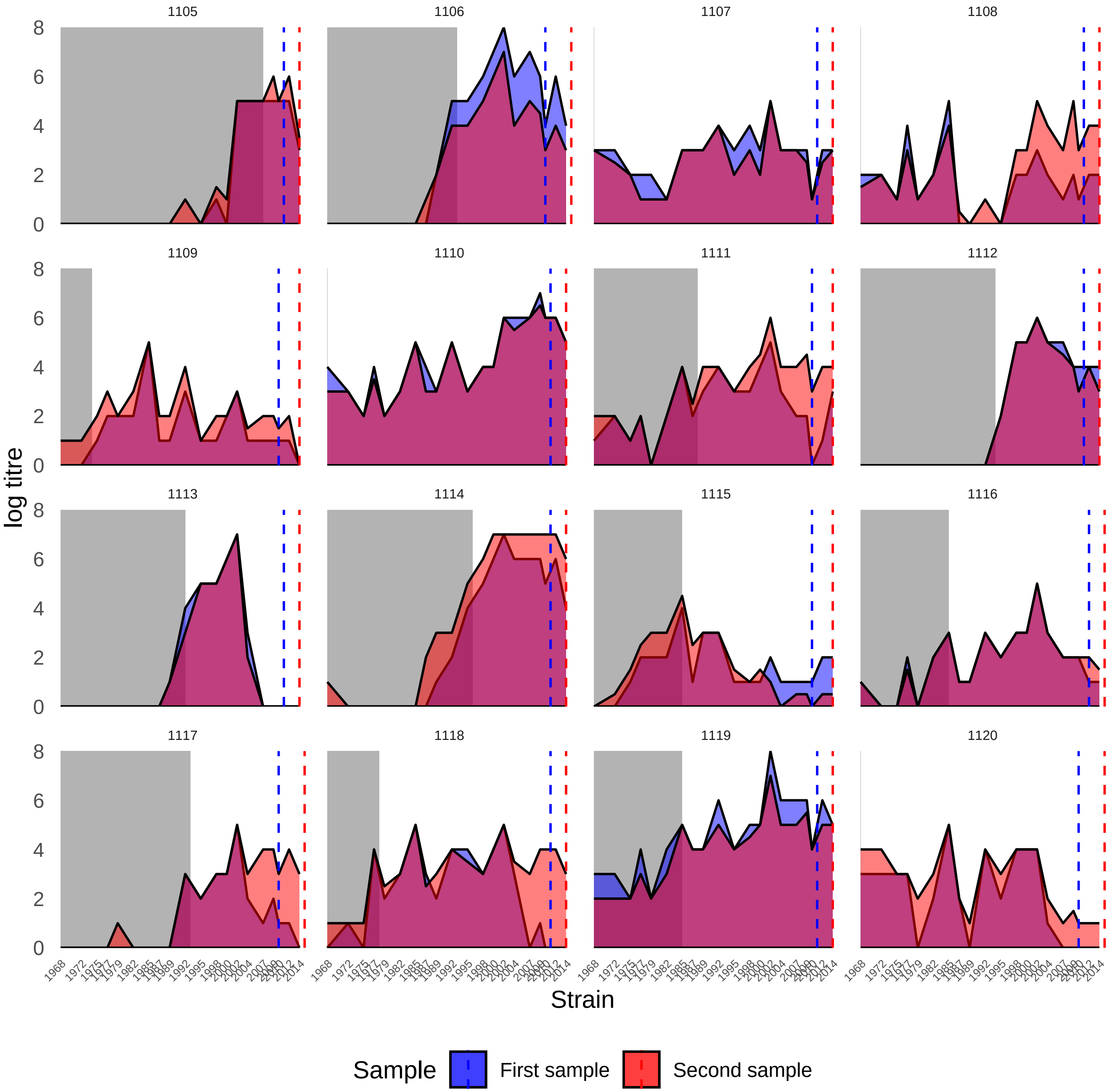
1105
1106
1107
1108
1109
1110
1111
1112
1113
1114
1115
1116
1117
1118
1119
1120
log titre
Strain
Sample
First sample
Second sample

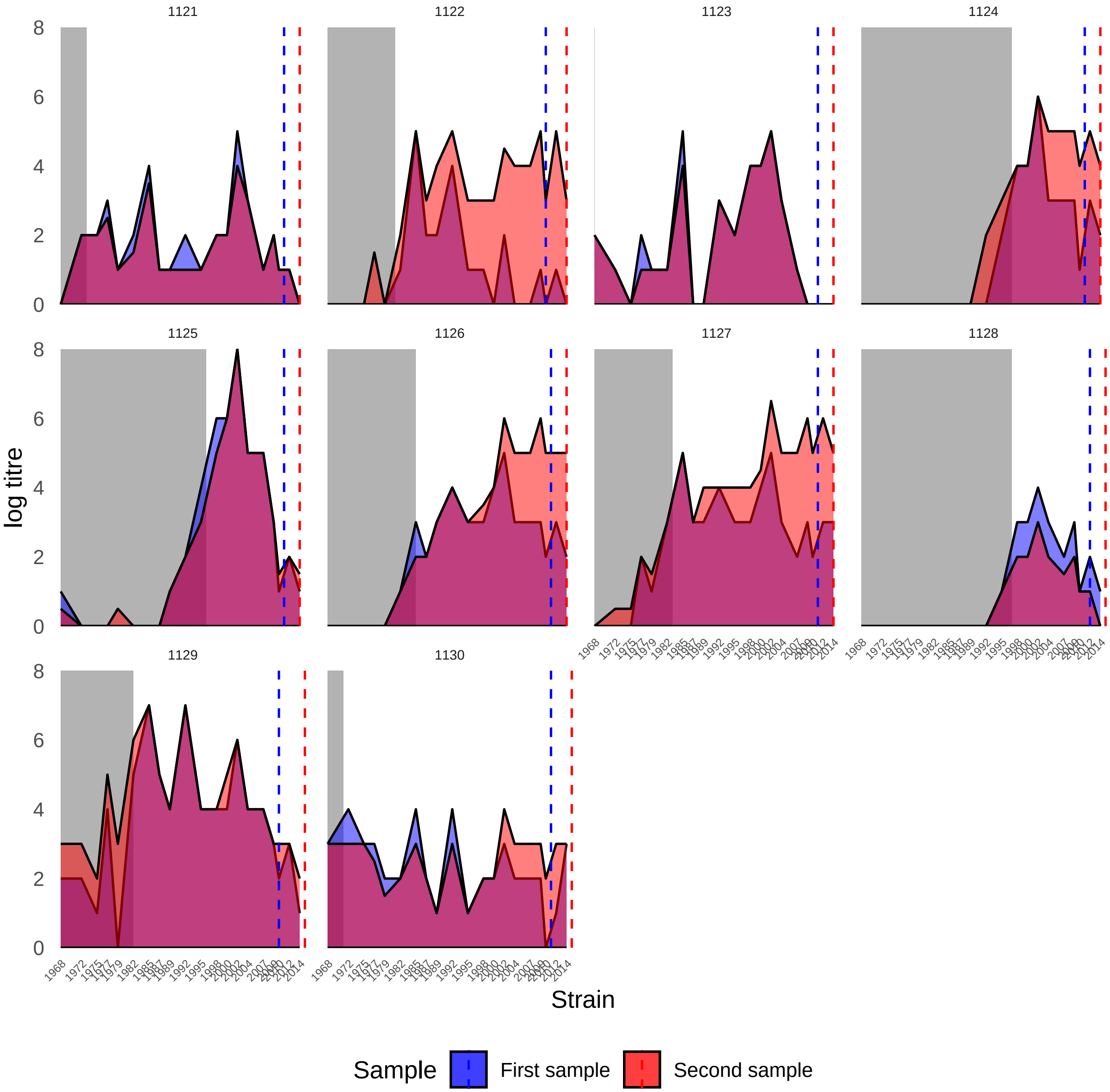
1121
1122
1123
1124
1125
1126
1127
1128
1129
1130
log titre
0
2
4
6
8
1968
1972
1975
1977
1979
1982
1985
1987
1989
1992
1995
1998
2000
2002
2004
2007
2009
2010
2012
2014
Strain
Sample
First sample
Second sample
