# Supplementary material for: Reconstructed influenza A/H3N2 infection histories reveal variation in incidence and antibody dynamics over the life course
Source: PLoS Biol. 2024 Nov 7;22(11):e3002864. doi: 10.1371/journal.pbio.3002864 (PMC11542844; doi:10.1371/journal.pbio.3002864)
Supplement: S22 Fig — Left-hand column shows raw data as in S20 Fig. Right-hand column shows the model-estimated posterior probability of infection (higher grey area suggested higher probability of infection) compared to self-reported vaccination states. Orange regions show time periods in which individuals reported having been vaccinated for influenza. Purple regions show time periods in which individuals reported no vaccination for influenza. Vertical dashed lines show the time of serum sample collection. Individuals were included in this plot (rather than S23 Fig) if the posterior probability of infection during one of the orange time windows was >25%. The data underlying this figure can be found at https://doi.org/10.5281/zenodo.12795911. (PDF) [file pbio.3002864.s022.pdf]

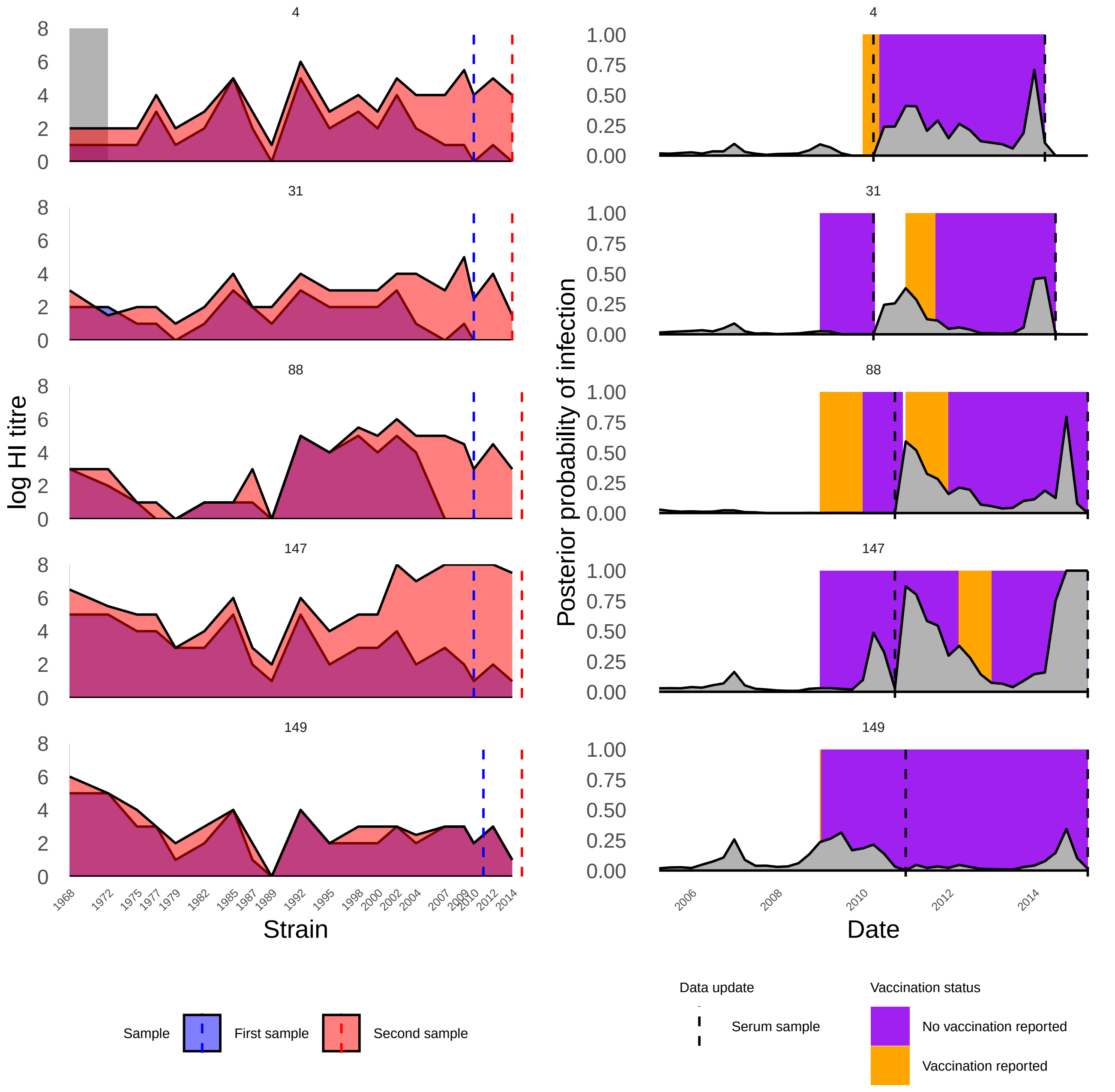

4
31
88
147
149
log HI titre
Strain
Posterior probability of infection
Date
Sample
First sample
Second sample
Data update
Serum sample
Vaccination status
No vaccination reported
Vaccination reported

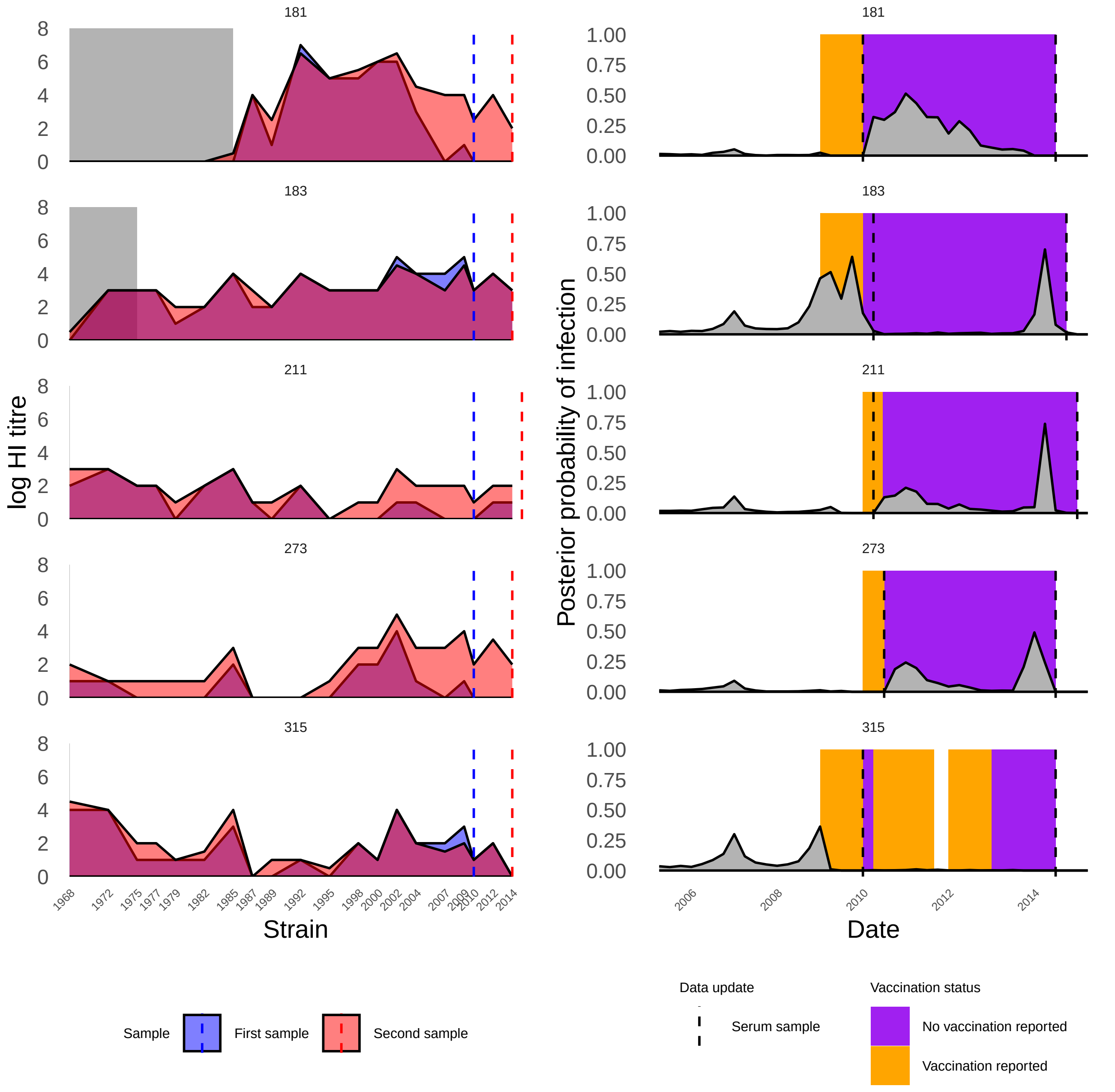

181
183
211
273
315
log HI titre
Posterior probability of infection
Strain
Date
Sample
First sample
Second sample
Data update
Serum sample
Vaccination status
No vaccination reported
Vaccination reported

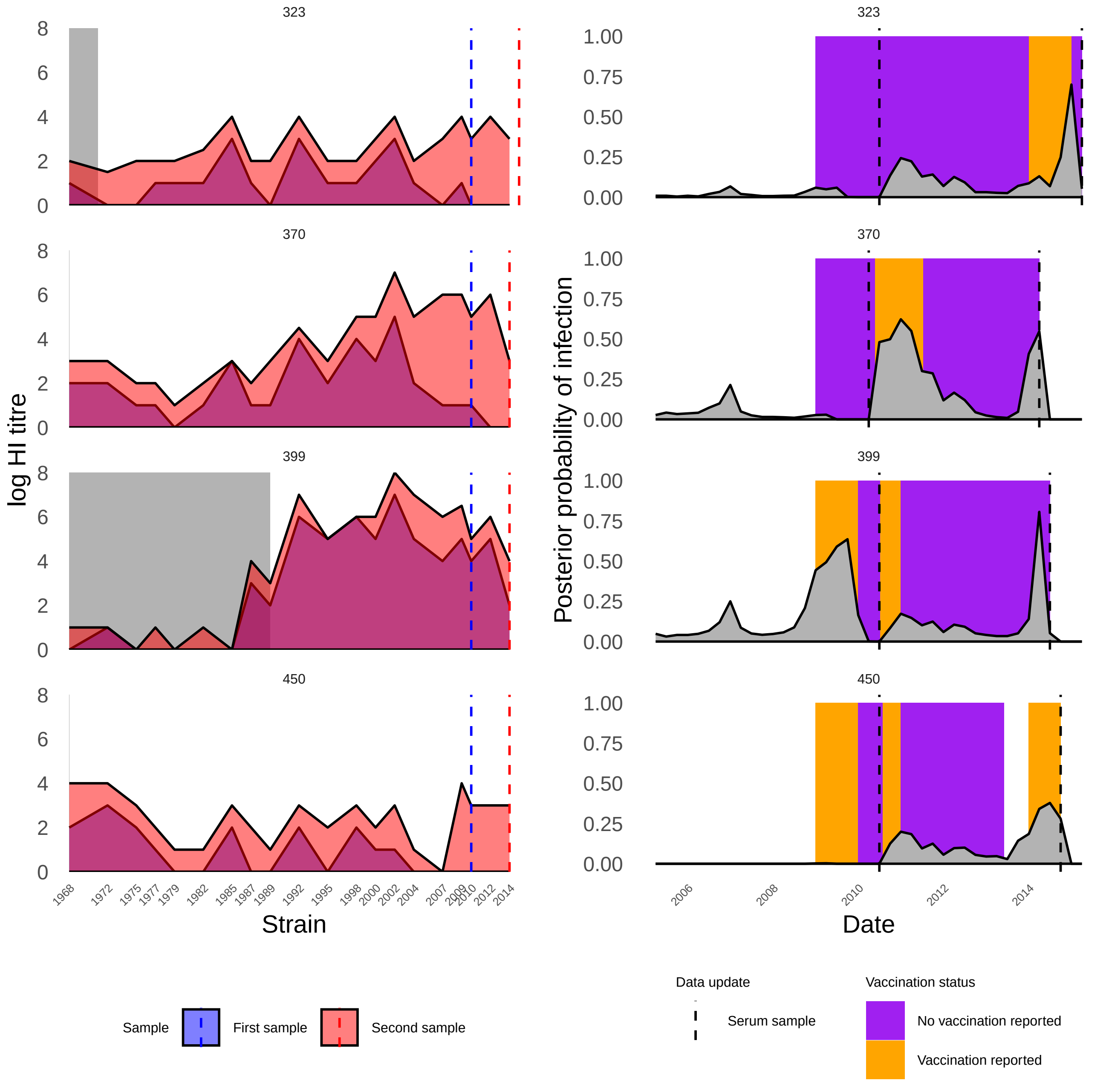

323
370
399
450
log HI titre
Strain
1968
1972
1975
1977
1979
1982
1985
1987
1989
1992
1995
1998
2000
2002
2004
2007
2009
2010
2012
2014
Posterior probability of infection
Date
2006
2008
2010
2012
2014
Sample
First sample
Second sample
Data update
Serum sample
Vaccination status
No vaccination reported
Vaccination reported

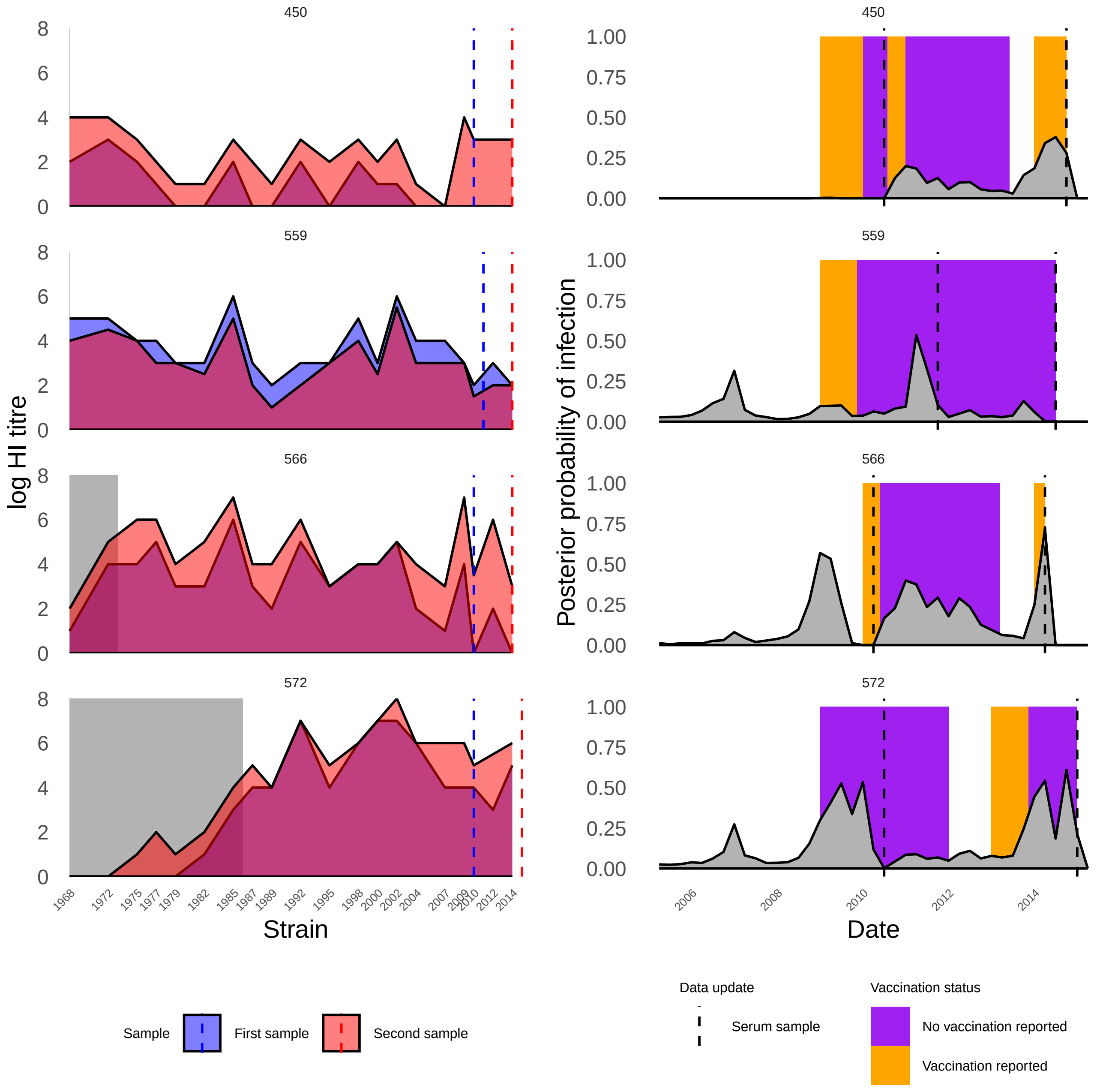
450
559
566
572
log HI titre
Strain
1968
1972
1975
1977
1979
1982
1985
1987
1989
1992
1995
1998
2000
2002
2004
2007
2009
2010
2012
2014
Posterior probability of infection
Date
2006
2008
2010
2012
2014
Sample
First sample
Second sample
Data update
Serum sample
Vaccination status
No vaccination reported
Vaccination reported

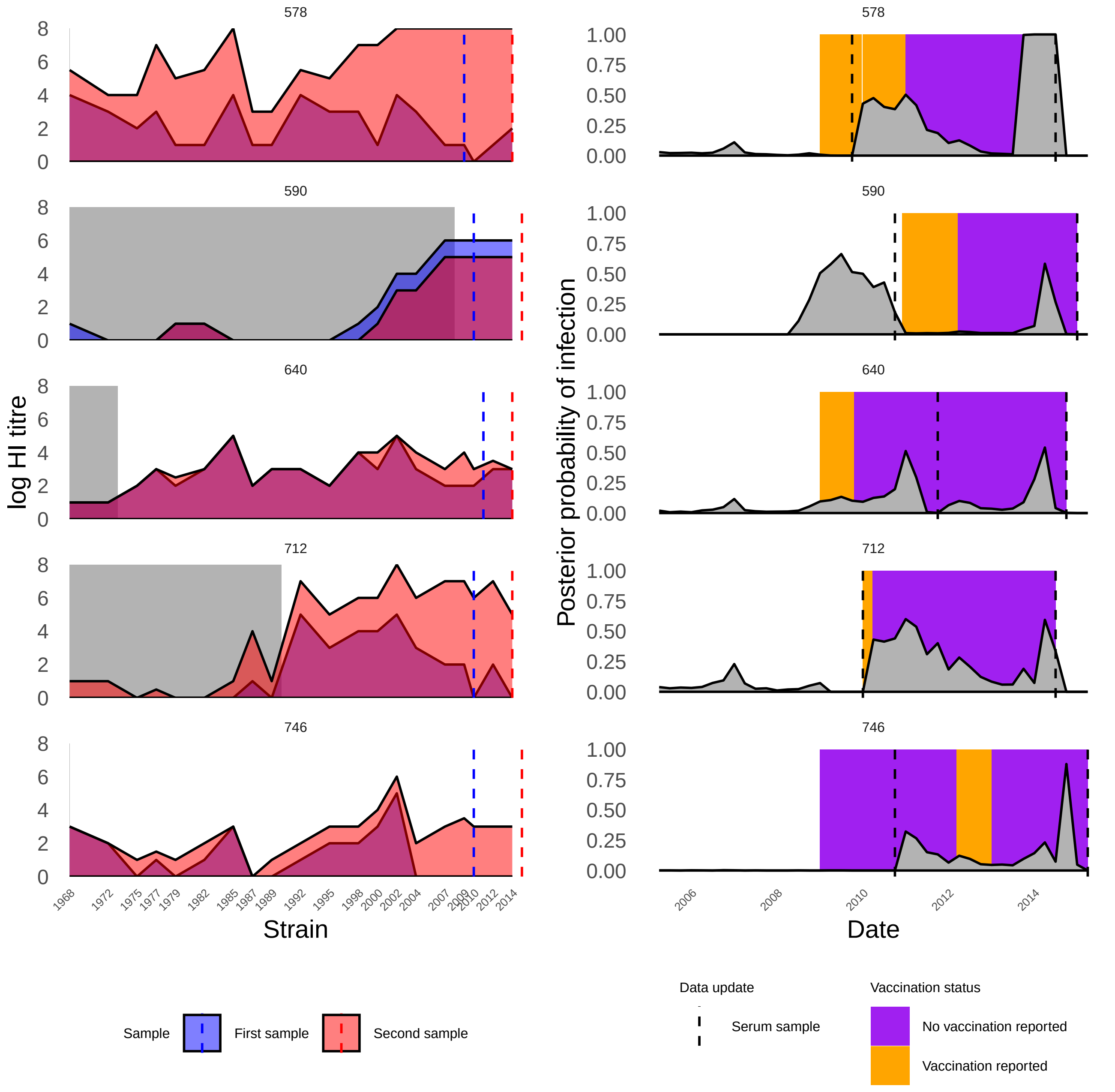
578
590
640
712
746
log HI titre
Strain
Posterior probability of infection
Date
Sample
First sample
Second sample
Data update
Serum sample
Vaccination status
No vaccination reported
Vaccination reported

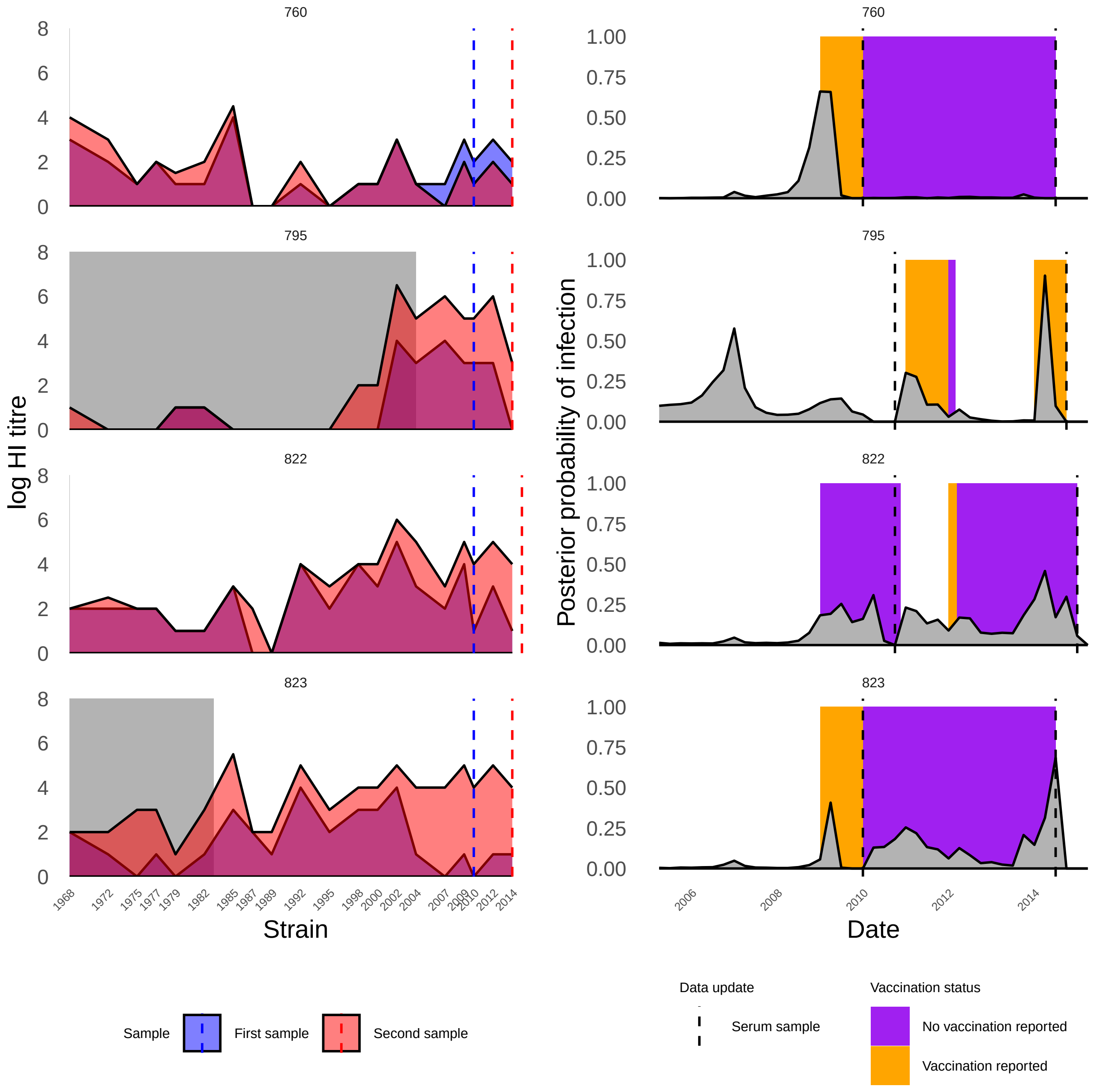

760
795
822
823
log HI titre
Strain
Posterior probability of infection
Date
Sample
First sample
Second sample
Data update
Serum sample
Vaccination status
No vaccination reported
Vaccination reported

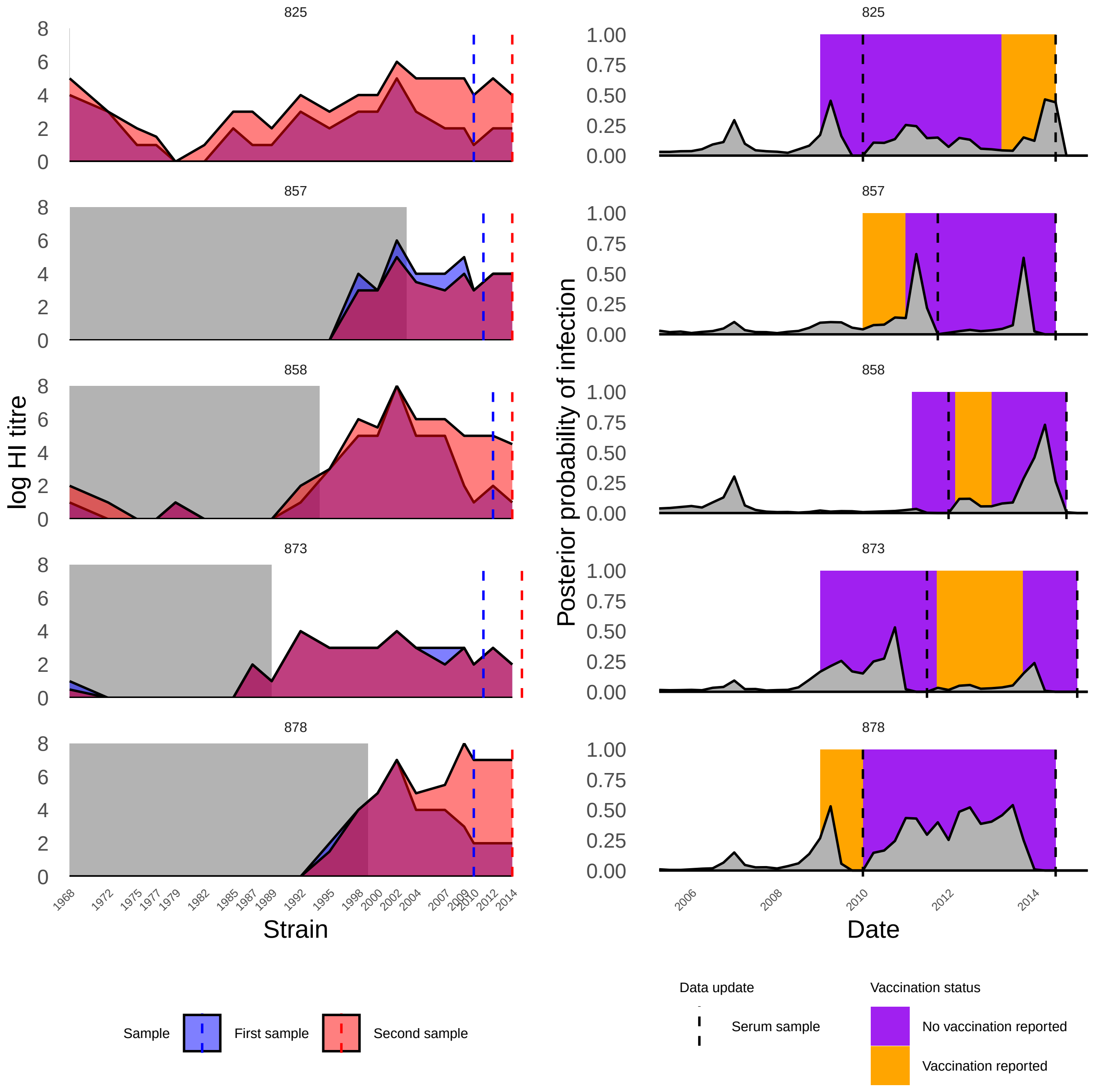

825
857
858
873
878
log HI titre
Strain
1968
1972
1975
1977
1979
1982
1985
1987
1989
1992
1995
1998
2000
2002
2004
2007
2009
2010
2012
2014
Posterior probability of infection
Date
2006
2008
2010
2012
2014
Sample
First sample
Second sample
Data update
Serum sample
Vaccination status
No vaccination reported
Vaccination reported

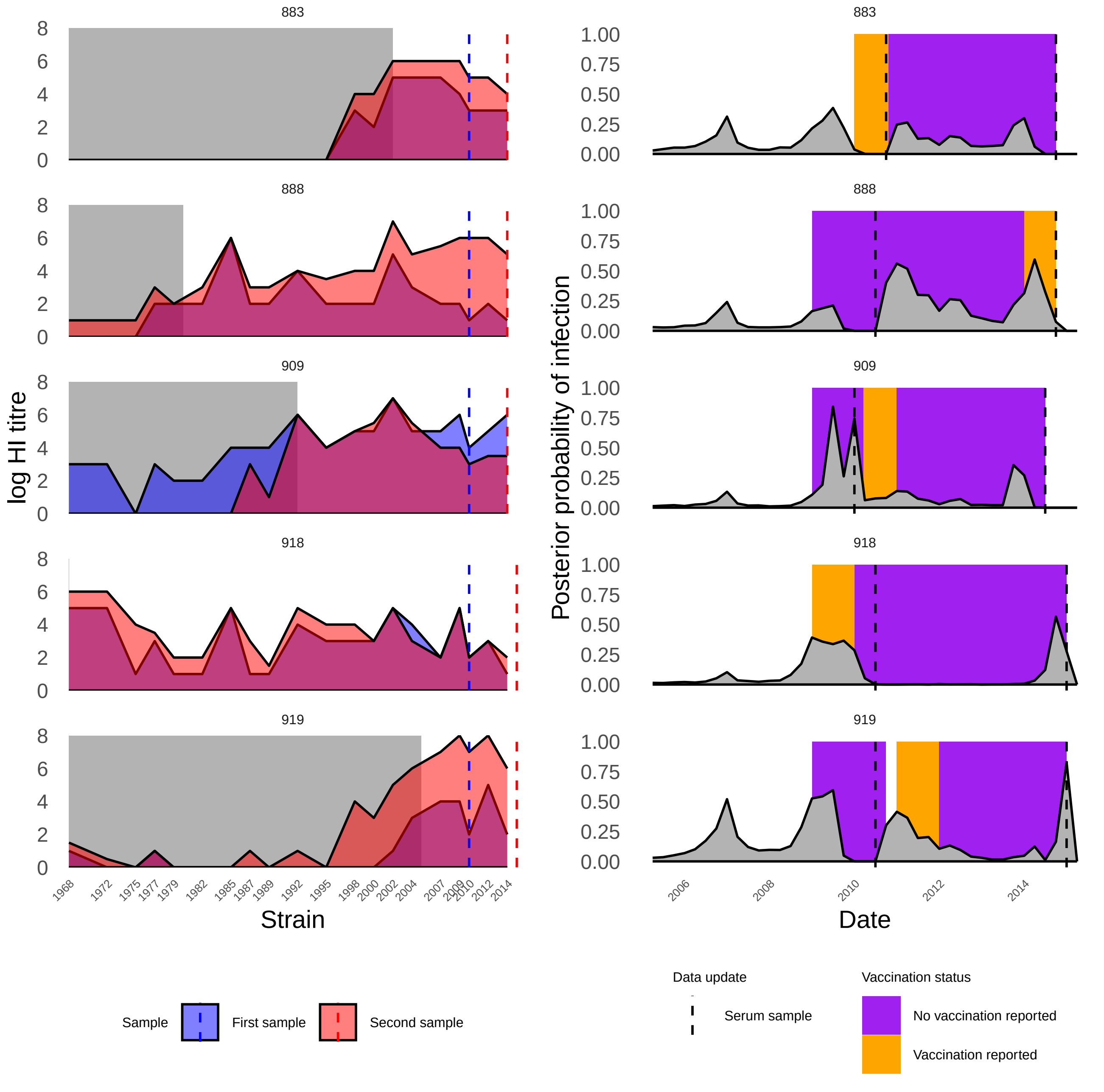
883
888
909
918
919
log HI titre
Strain
1968
1972
1975
1977
1979
1982
1985
1987
1989
1992
1995
1998
2000
2002
2004
2007
2009
2010
2012
2014
Posterior probability of infection
Date
2006
2008
2010
2012
2014
Sample
First sample
Second sample
Data update
Serum sample
Vaccination status
No vaccination reported
Vaccination reported

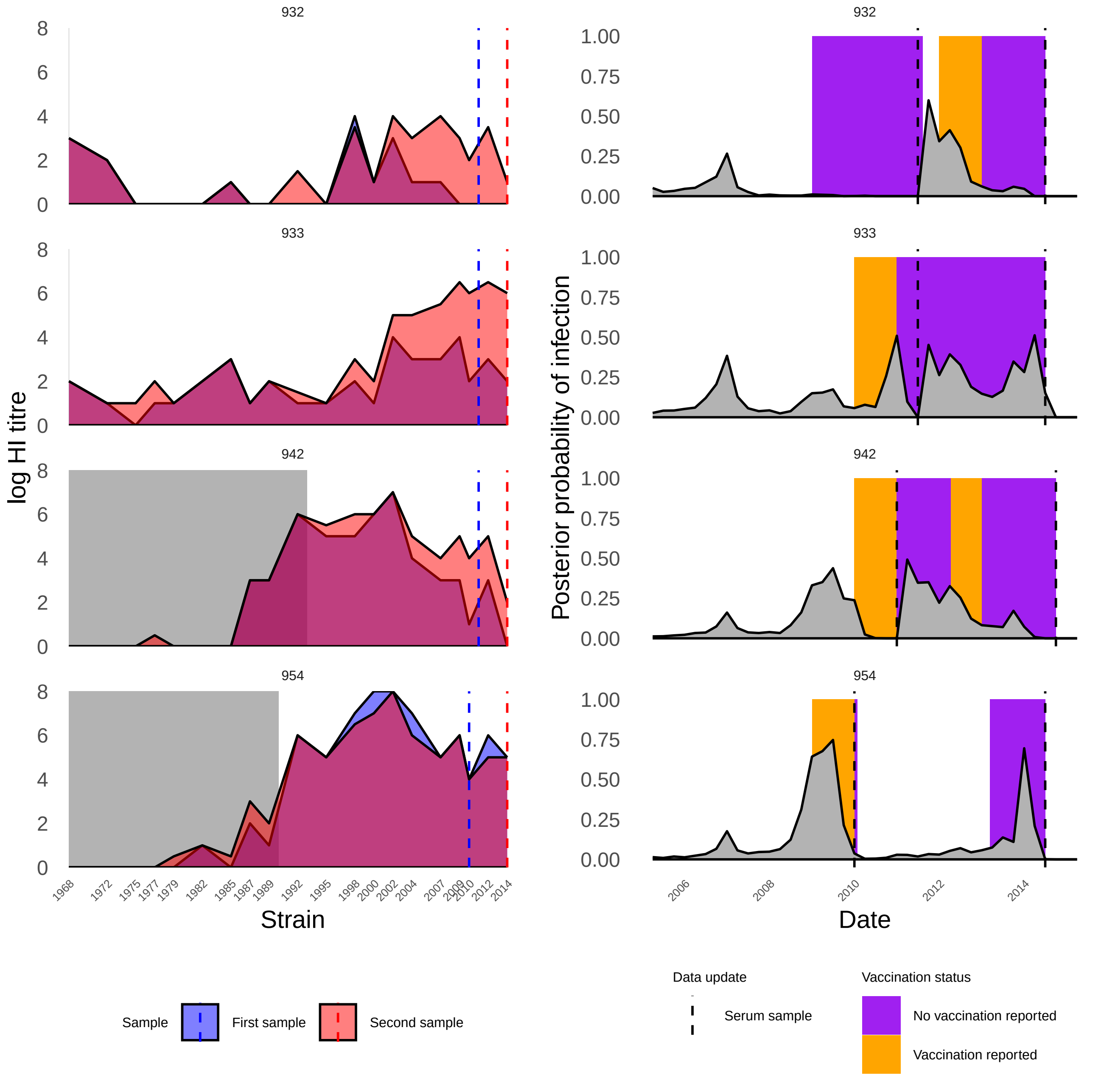

932
933
942
954
log HI titre
Strain
Posterior probability of infection
Date
Sample
First sample
Second sample
Data update
Serum sample
Vaccination status
No vaccination reported
Vaccination reported

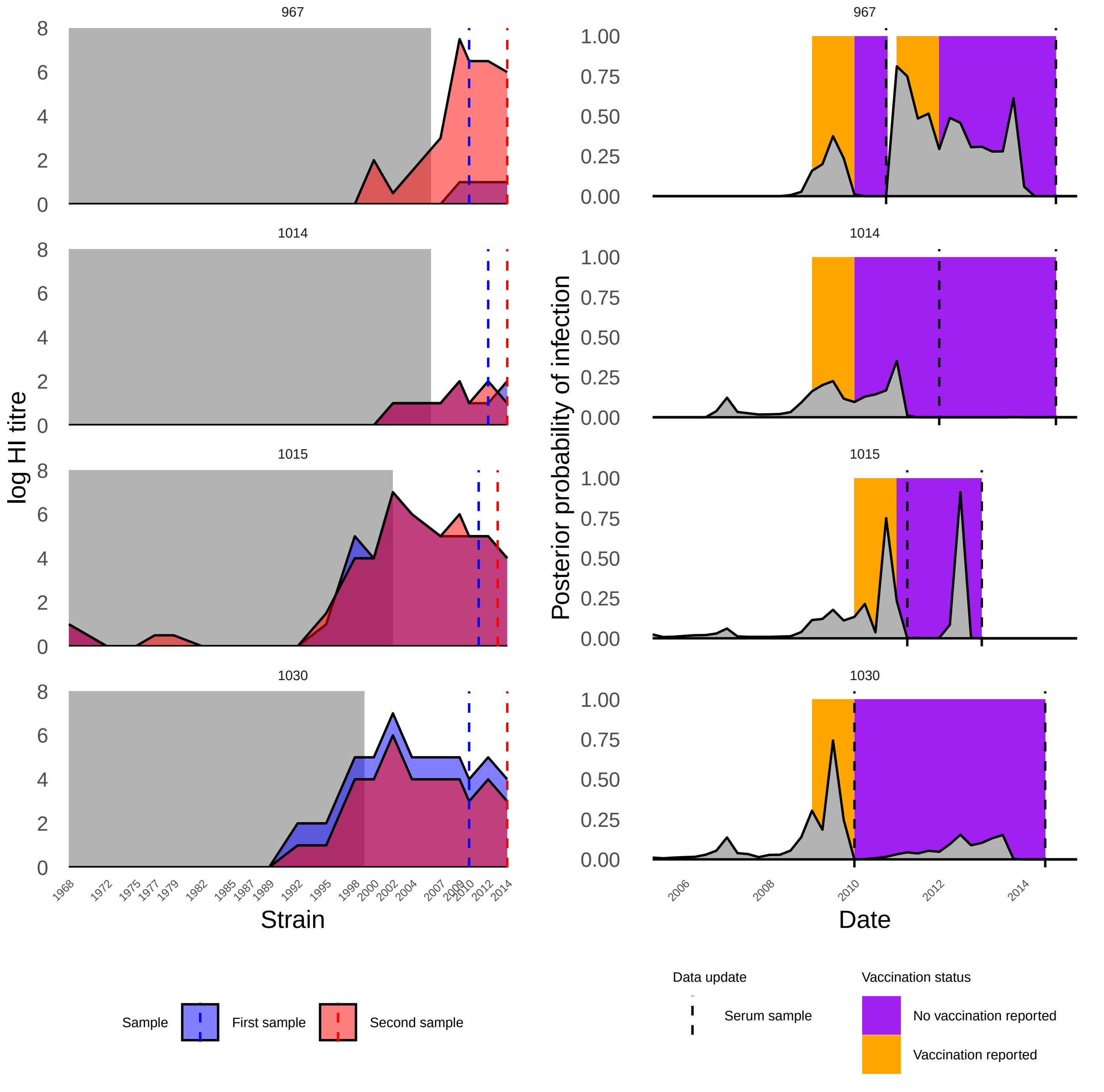

967
1014
1015
1030
log HI titre
Strain
Posterior probability of infection
Date
Sample
First sample
Second sample
Data update
Serum sample
Vaccination status
No vaccination reported
Vaccination reported

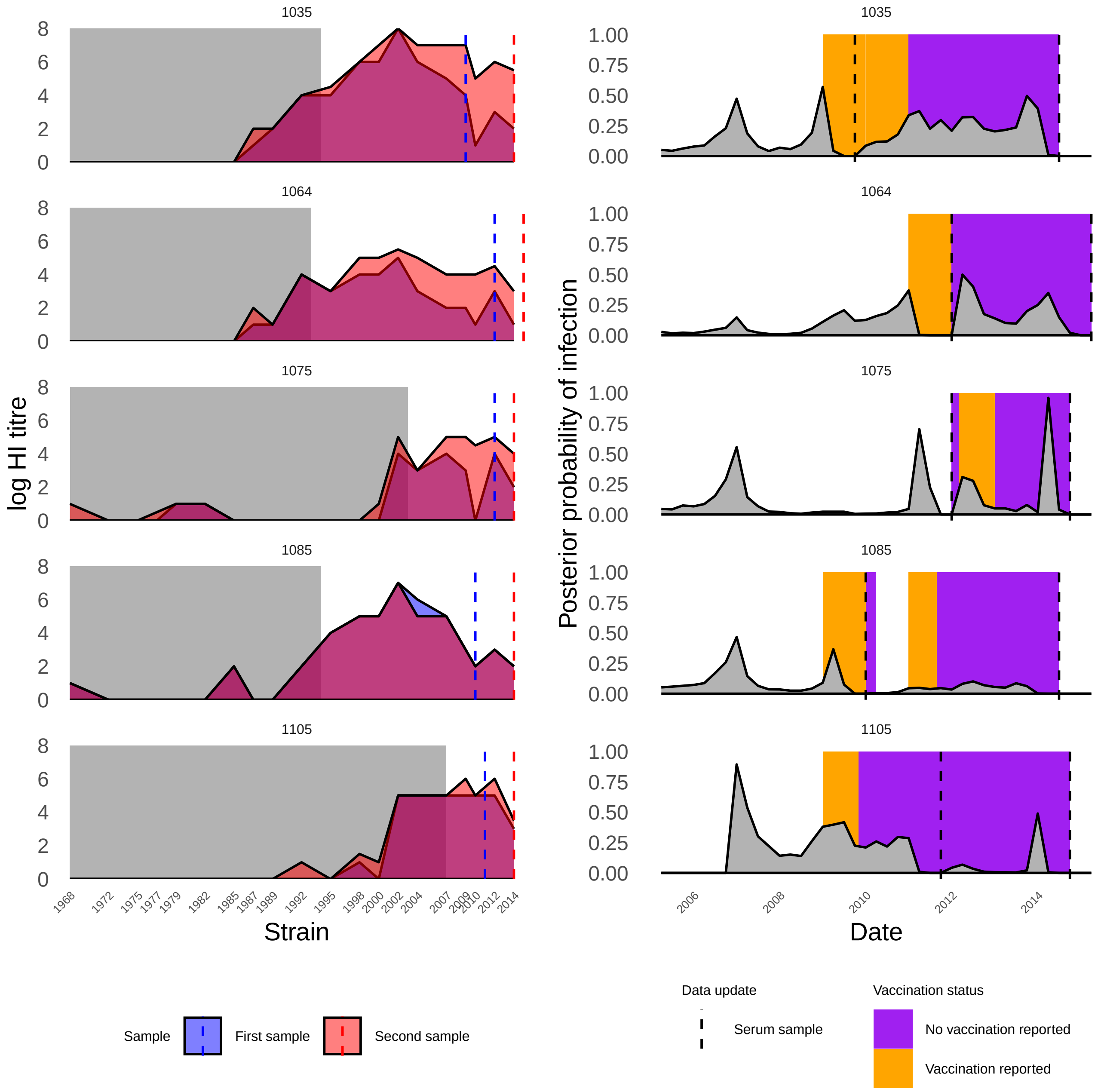

1035
1064
1075
1085
1105
log HI titre
Strain
1968
1972
1975
1977
1979
1982
1985
1987
1989
1992
1995
1998
2000
2002
2004
2007
2009
2010
2012
2014
Posterior probability of infection
Date
2006
2008
2010
2012
2014
Sample
First sample
Second sample
Data update
Serum sample
Vaccination status
No vaccination reported
Vaccination reported

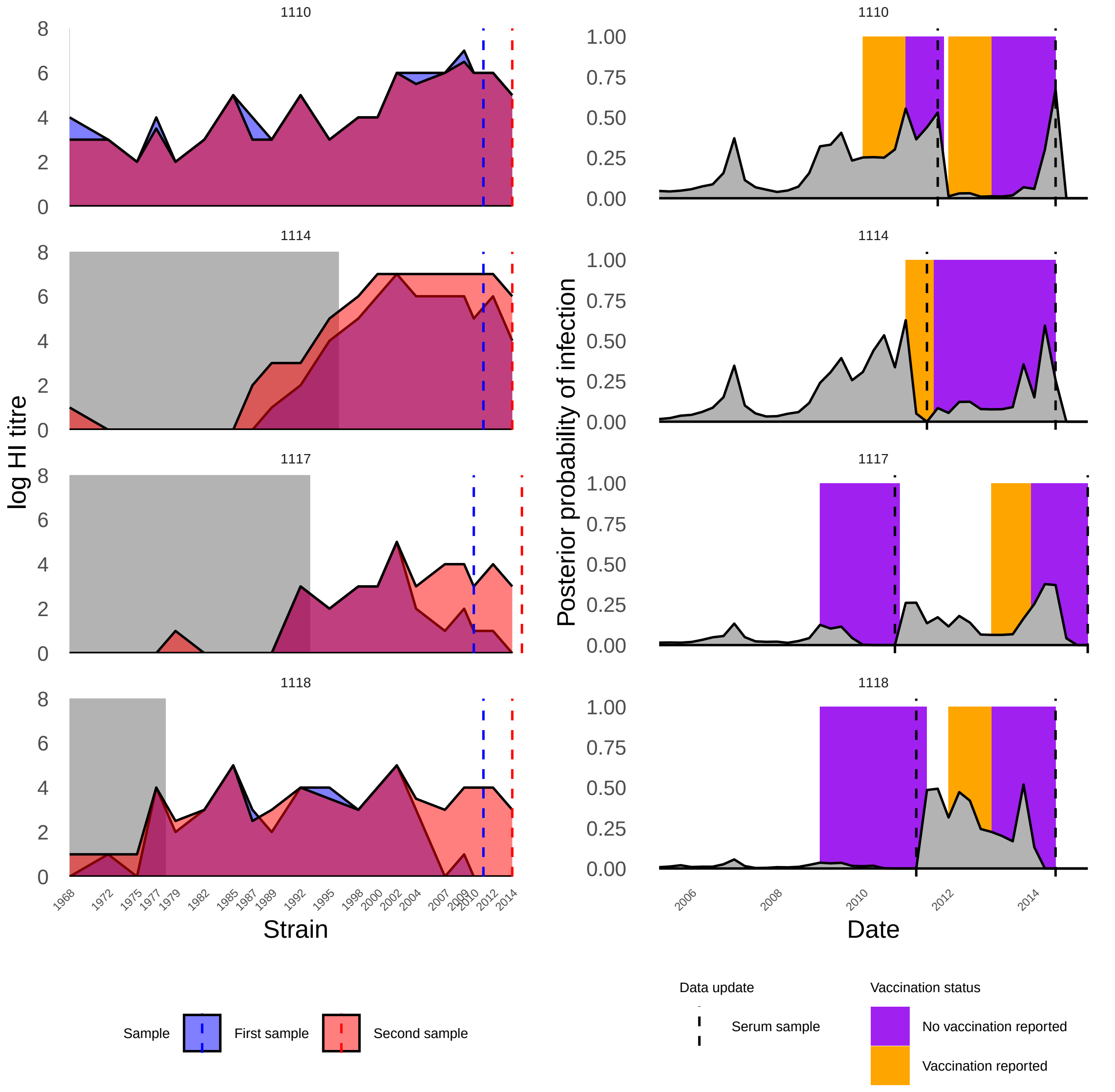

1110
1114
1117
1118
log HI titre
Strain
1968
1972
1975
1977
1979
1982
1985
1987
1989
1992
1995
1998
2000
2002
2004
2007
2009
2010
2012
2014
Posterior probability of infection
Date
2006
2008
2010
2012
2014
Sample
First sample
Second sample
Data update
Serum sample
Vaccination status
No vaccination reported
Vaccination reported

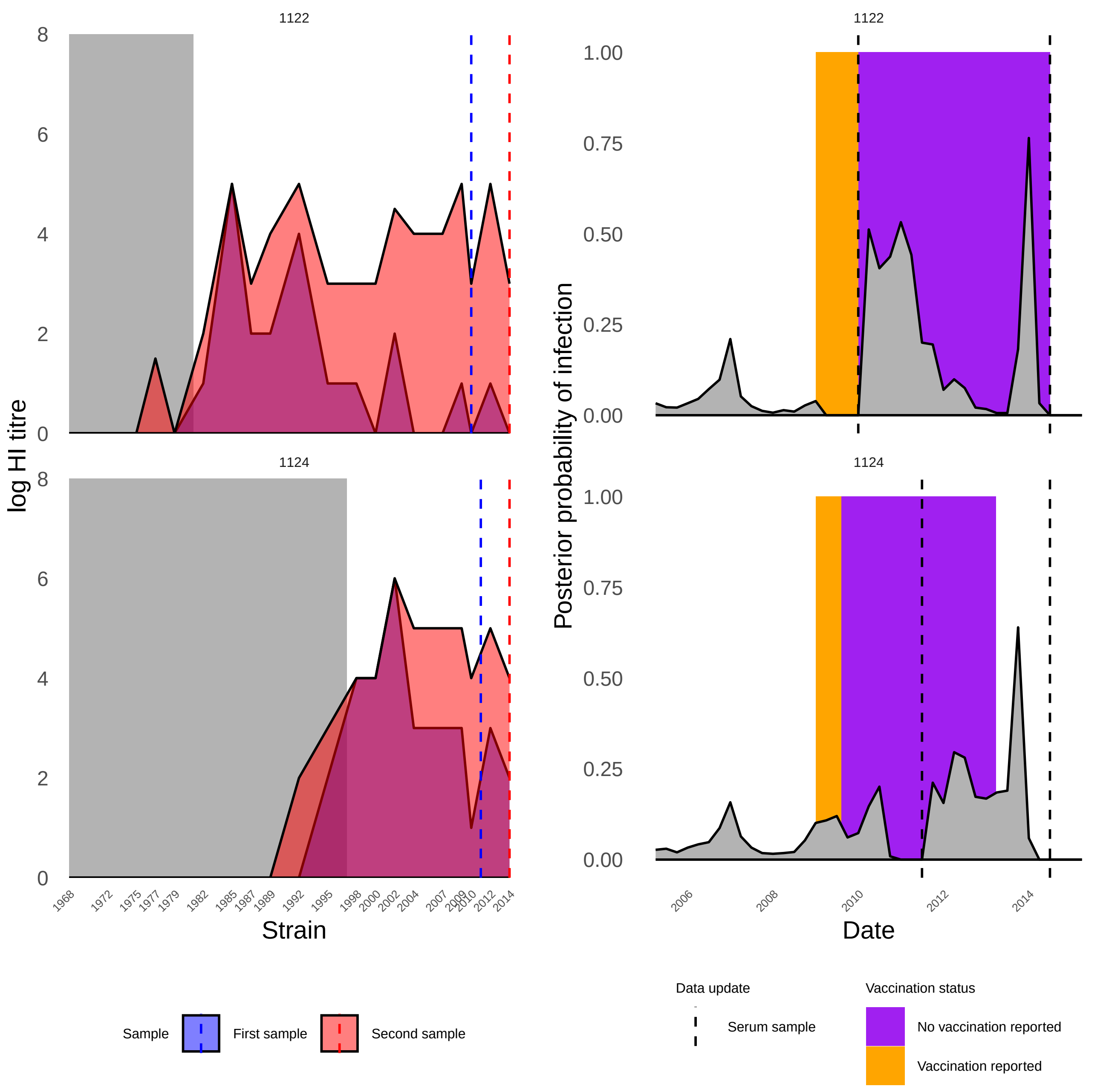

1122
1124
log HI titre
Strain
Posterior probability of infection
Date
Sample
First sample
Second sample
Data update
Serum sample
Vaccination status
No vaccination reported
Vaccination reported
