# Supplementary material for: Reconstructed influenza A/H3N2 infection histories reveal variation in incidence and antibody dynamics over the life course
Source: PLoS Biol. 2024 Nov 7;22(11):e3002864. doi: 10.1371/journal.pbio.3002864 (PMC11542844; doi:10.1371/journal.pbio.3002864)
Supplement: S23 Fig — Left-hand column shows raw data as in S20 Fig. Right-hand column shows the model-estimated posterior probability of infection (higher grey area suggested higher probability of infection) compared to self-reported vaccination states. Orange regions show time periods in which individuals reported having been vaccinated for influenza. Purple regions show time periods in which individuals reported no vaccination for influenza. Vertical dashed lines show the time of serum sample collection. Individuals were included in this plot (rather than S22 Fig) if the posterior probability of infection during one of the orange time windows was <25%. The data underlying this figure can be found at https://doi.org/10.5281/zenodo.12795911. (PDF) [file pbio.3002864.s023.pdf]

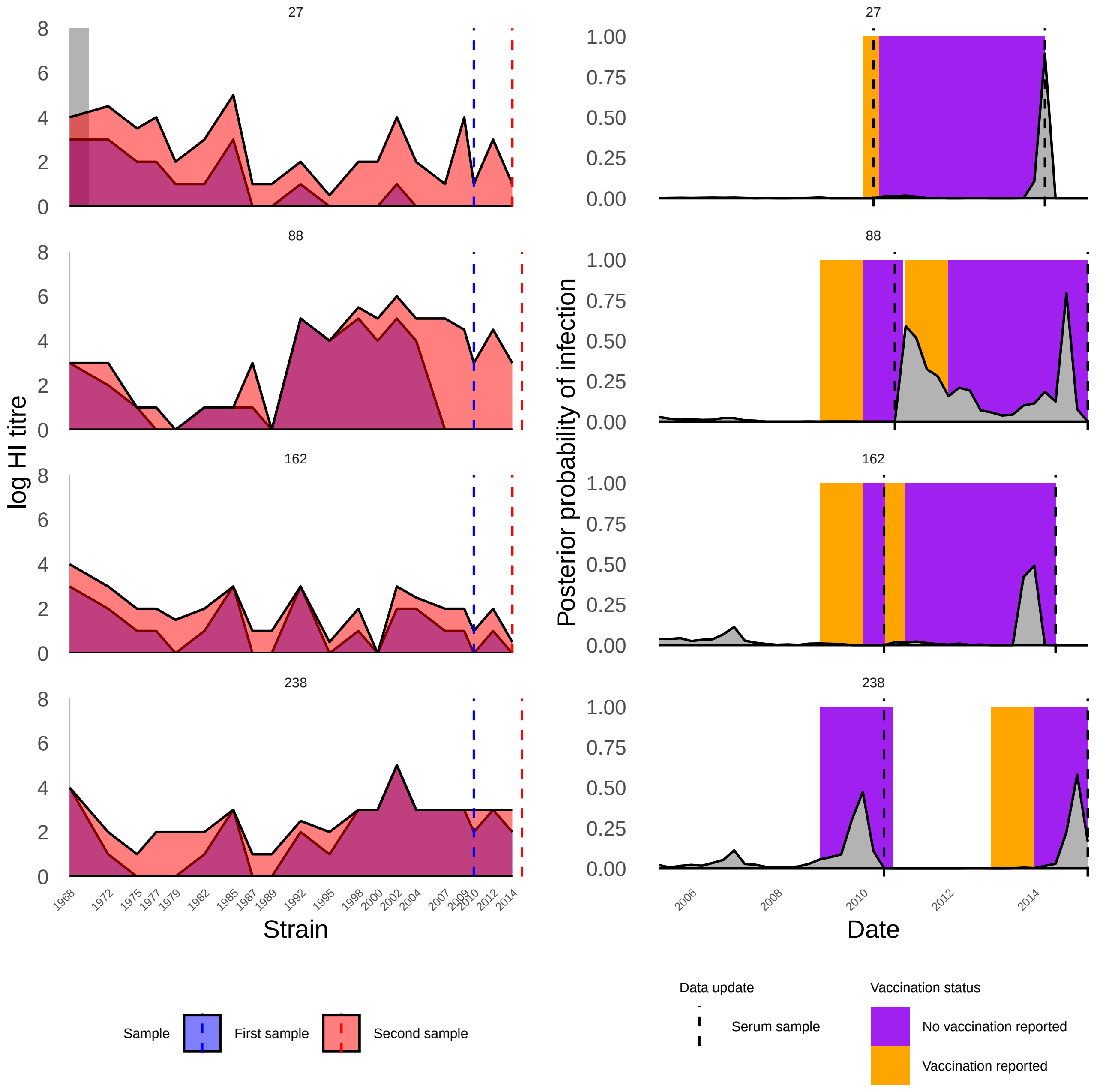

27
88
162
238
log HI titre
Strain
Posterior probability of infection
Date
Sample
First sample
Second sample
Data update
Serum sample
Vaccination status
No vaccination reported
Vaccination reported

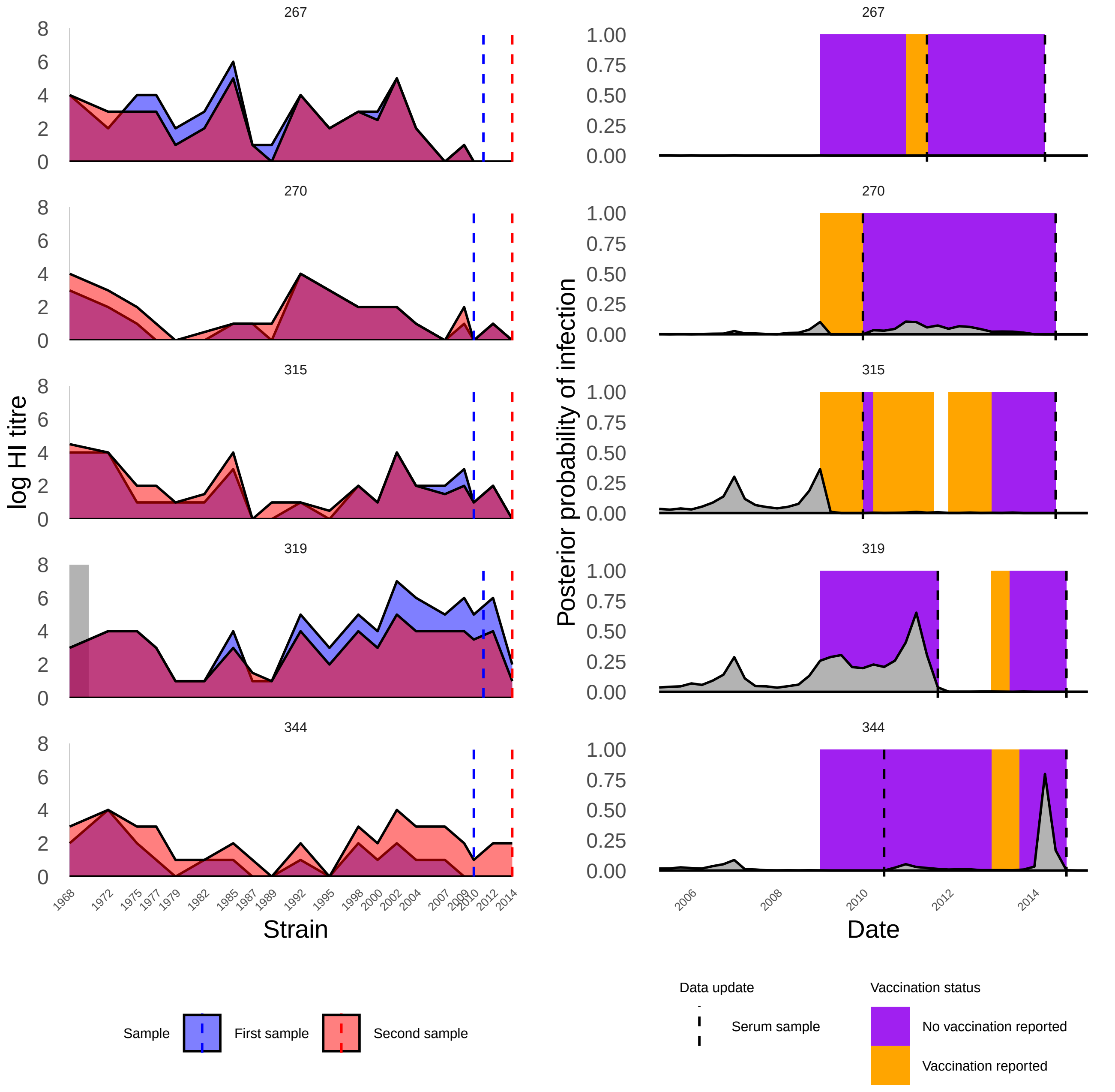

267
270
315
319
344
log HI titre
Posterior probability of infection
Strain
Date
1968
1972
1975
1977
1979
1982
1985
1987
1989
1992
1995
1998
2000
2002
2004
2007
2009
2010
2012
2014
2006
2008
2010
2012
2014
Sample
First sample
Second sample
Data update
Serum sample
Vaccination status
No vaccination reported
Vaccination reported

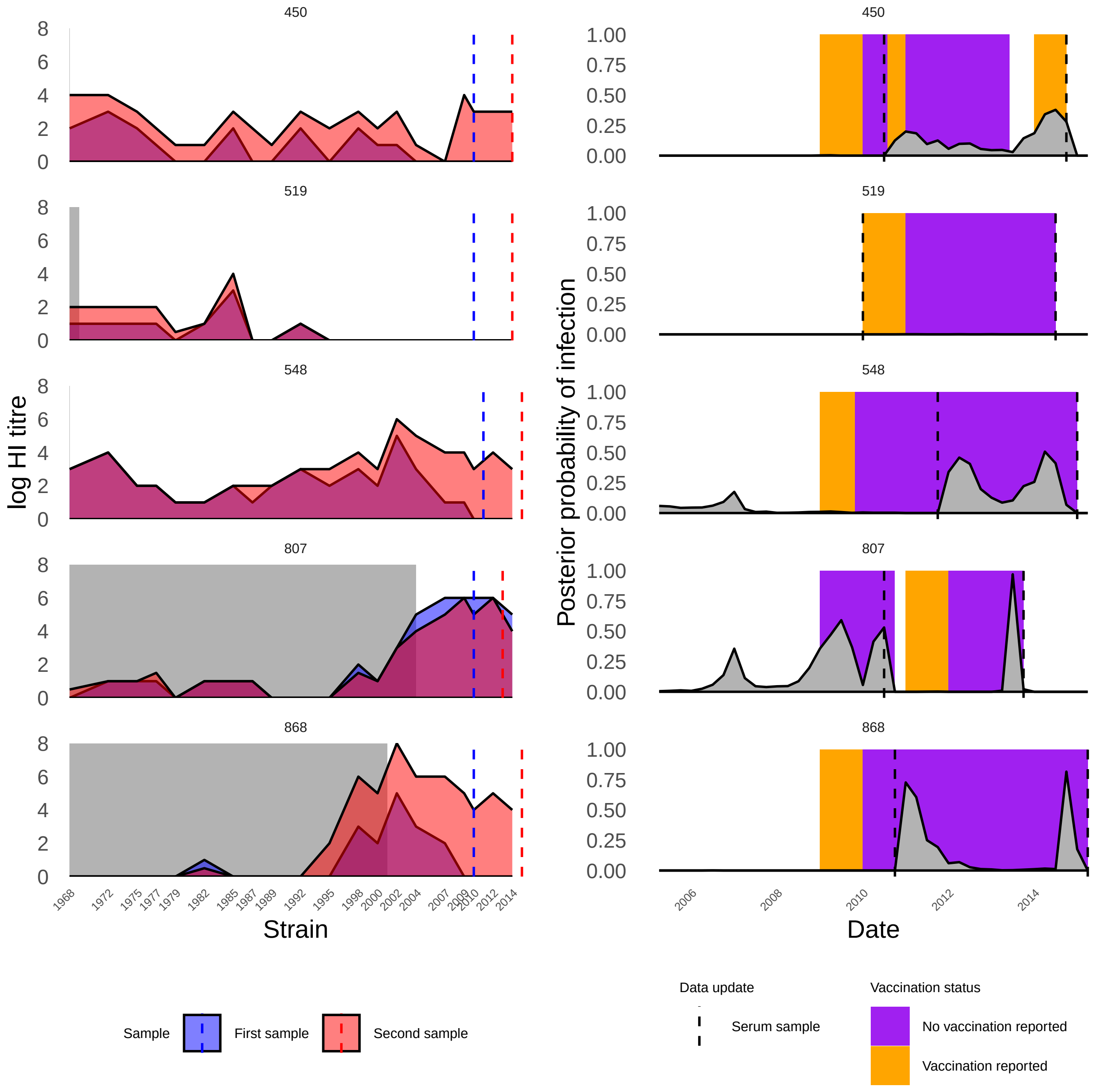

450
519
548
807
868
log HI titre
Strain
1968
1972
1975
1977
1979
1982
1985
1987
1989
1992
1995
1998
2000
2002
2004
2007
2009
2010
2012
2014
Posterior probability of infection
Date
2006
2008
2010
2012
2014
Sample
First sample
Second sample
Data update
Serum sample
Vaccination status
No vaccination reported
Vaccination reported

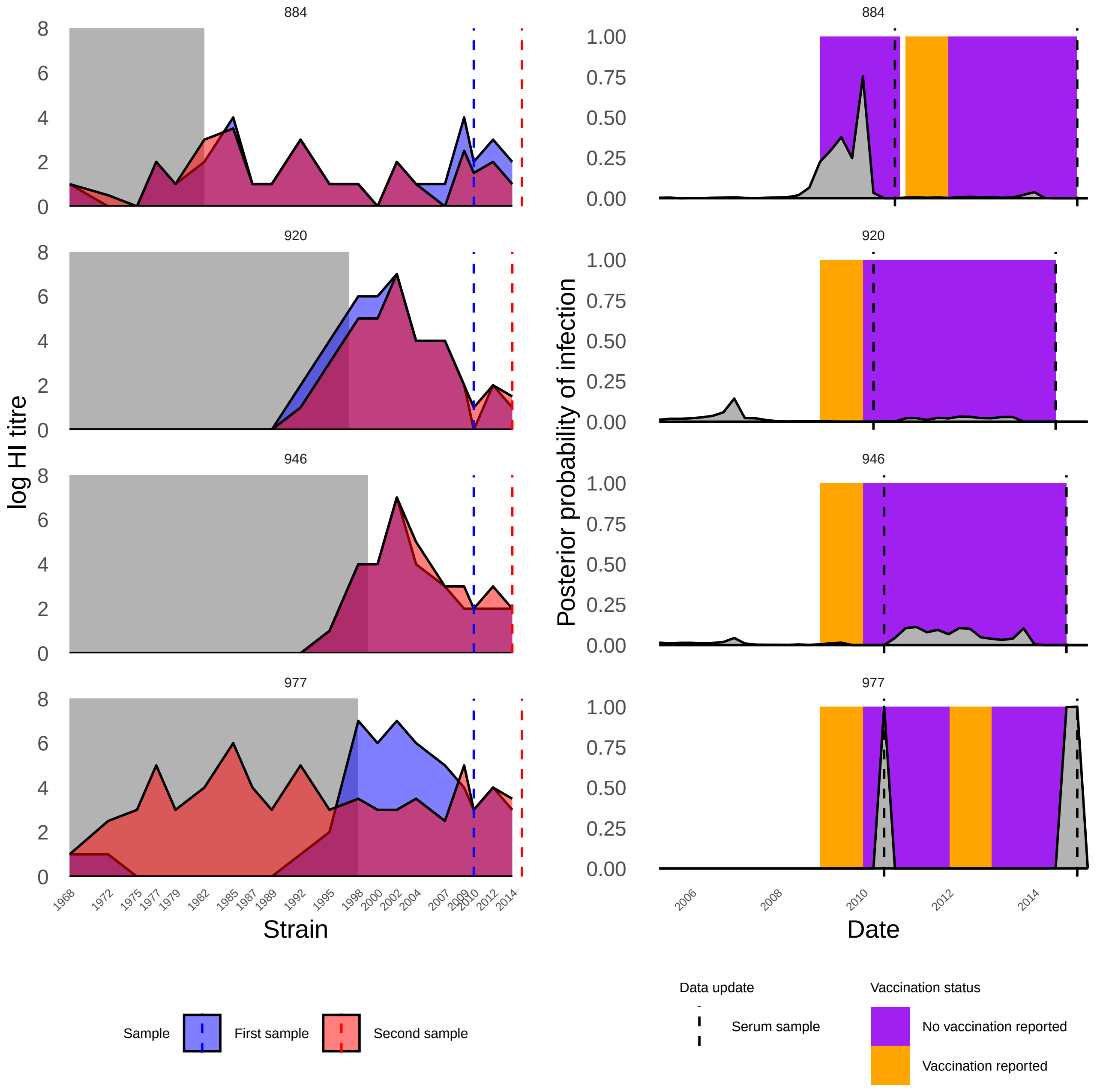

884
920
946
977
log HI titre
Strain
Posterior probability of infection
Date
Sample
First sample
Second sample
Data update
Serum sample
Vaccination status
No vaccination reported
Vaccination reported

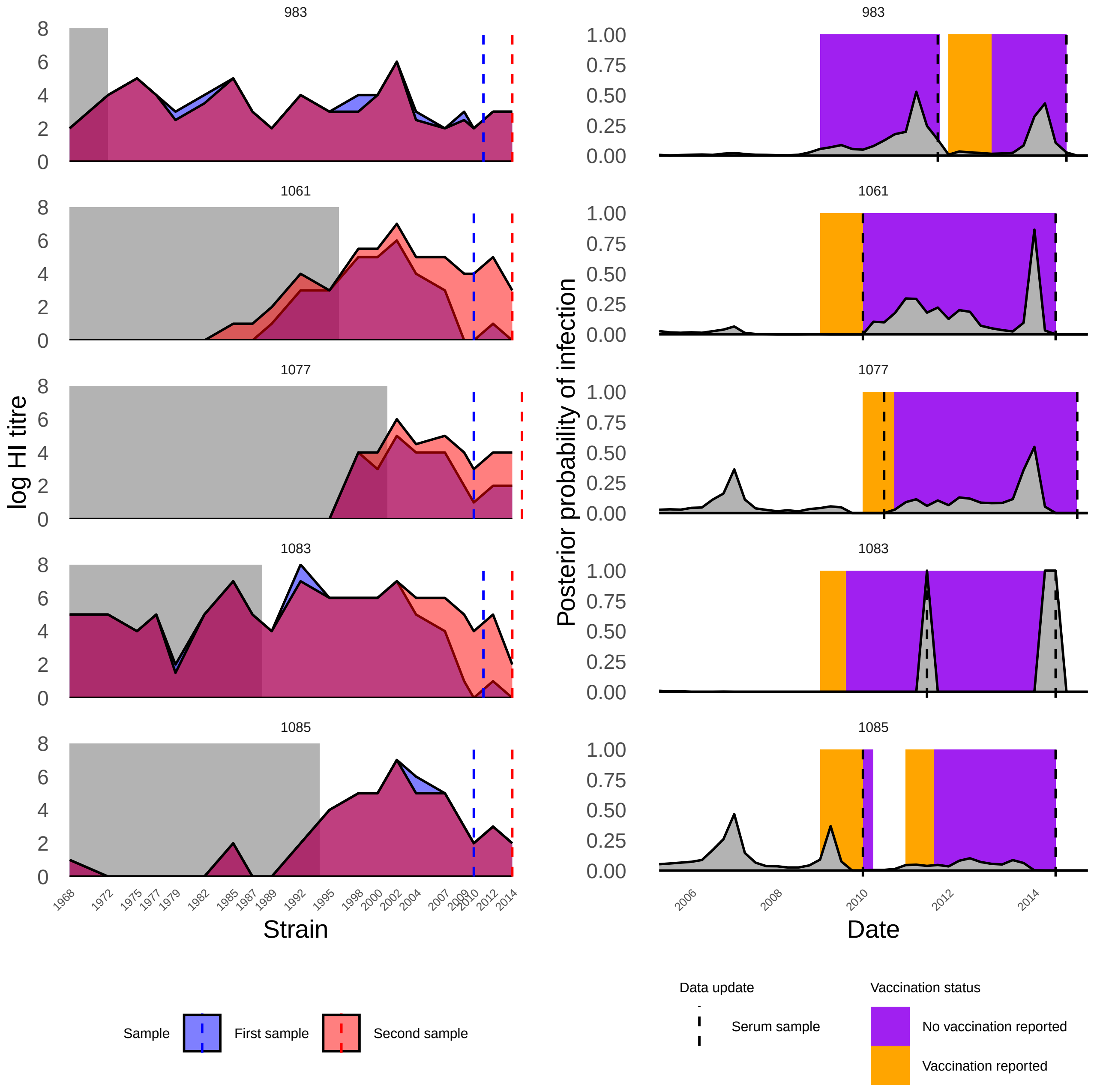

983
1061
1077
1083
1085
log HI titre
Strain
1968 1972 1975 1977 1979 1982 1985 1987 1989 1992 1995 1998 2000 2002 2004 2007 2009 2010 2012 2014
0 2 4 6 8
Posterior probability of infection
0.00 0.25 0.50 0.75 1.00
Date
2006 2008 2010 2012 2014
Sample
First sample
Second sample
Data update
Serum sample
Vaccination status
No vaccination reported
Vaccination reported

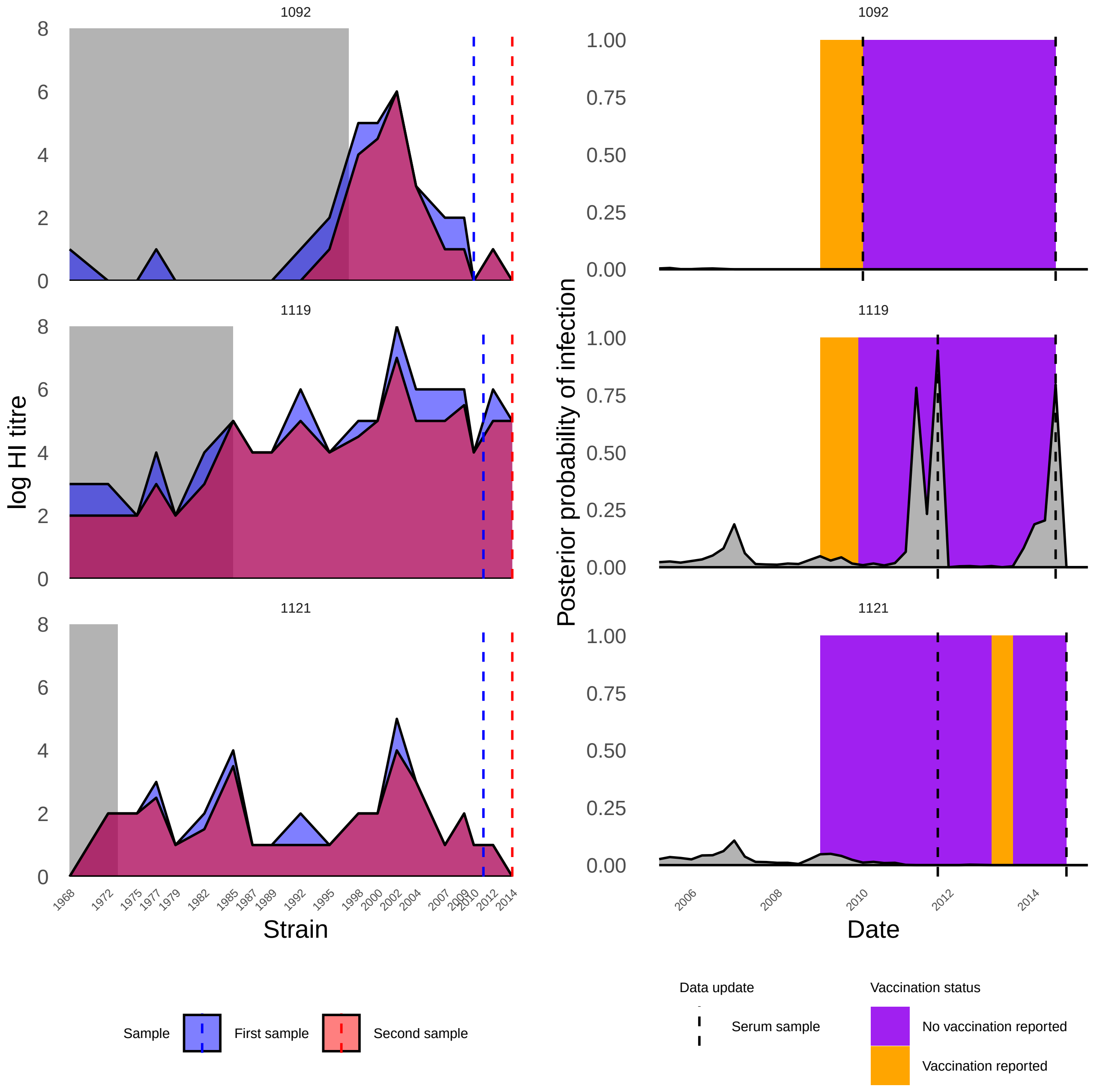

1092
1119
1121
log HI titre
Strain
1968
1972
1975
1977
1979
1982
1985
1987
1989
1992
1995
1998
2000
2002
2004
2007
2009
2010
2012
2014
Posterior probability of infection
Date
2006
2008
2010
2012
2014
Sample
First sample
Second sample
Data update
Serum sample
Vaccination status
No vaccination reported
Vaccination reported
