# Supplementary material for: Reconstructed influenza A/H3N2 infection histories reveal variation in incidence and antibody dynamics over the life course
Source: PLoS Biol. 2024 Nov 7;22(11):e3002864. doi: 10.1371/journal.pbio.3002864 (PMC11542844; doi:10.1371/journal.pbio.3002864)
Supplement: S1 Text — (DOCX) [file pbio.3002864.s032.docx]

**Reconstructed influenza A/H3N2 infection histories reveal variation in incidence and antibody dynamics over the life course**

**S1 Text: Estimation of strain-specific measurement offsets**

Our initial approach was to fit the full *serosolver* model exactly as described in the Materials and Methods, but without the inclusion of strain-specific measurement offsets (i.e., set all *χ_j_* to 0). Although convergence diagnostics were acceptable, we noted that estimated attack rates were extremely high (or low) in some time periods ([S16](#kix.xr41ui33egq0) Fig). These unusually high or low estimates were associated with systematic under- or over-estimation of expected log HI titres compared to the observed values ([S17](#kix.ahft76hioniy) Fig). For example, model predicted titres are systematically lower than observations for the viruses that circulated in years 1985 and 2002, but systematically higher for years 1995 and 2010. These strain-specific biases are potentially problematic for the attack rate estimates, as the model can only account for elevated titres against a particular strain through adding more infections. If some viruses have systematically higher antibody titres simply due to measurement bias, then the model may incorrectly infer more infections than occurred.

These systematic measurement biases have a number of possible biological explanations. Serum potency and virus avidity, resulting in different haemagglutinin (HA) reactivity [1,2], are known sources of variation when performing antigenic cartography using ferret sera. This effect was noted by Fonville et al. when using locally-weighted multiple linear regression to fit antibody landscape surfaces to antibody titre data. Fonville et al. found that some viruses had systematically under-predicted values (e.g., NL/620/89) whereas others were systematically overestimated (e.g., Victoria/361/11) [3]. This phenomenon was also described by Bedford et al. when simultaneously performing antigenic cartography alongside phylogenetic tree reconstruction using both genetic and HI titre data [4]. Bedford et al. found models that explicitly estimated parameters for “virus avidity” to represent the contribution of virus-specific effects to observed titres were better supported than models that did not.

Including a strain-specific offset term as part of the observation process enables the antibody kinetics and infection model described here to account for these systematic biases. In lieu of generating these strain-specific offset terms from experimental data, we would ideally jointly infer these offsets alongside the infection histories and antibody kinetics parameters. However, our attempts to do so using the infection history prior used in the main text were unsuccessful and we could not generate converged chains. This is likely because the offset terms and attack rate estimates were highly correlated; elevated titres against a strain that circulated in a given year may be explained either by high seroresponse rates or systematic overestimation of titres to that strain.

Instead, we generated estimates for the measurement offsets using a modified version of the main text model. We fit the same antibody kinetics and infection histories to the full Fluscape dataset, but with three changes: (1) the observation model included an additional, estimated strain-specific offset parameter, *χ_j_*, for each measured strain as described in the Materials and Methods; (2) we estimated infection histories at an annual rather than 3-monthly resolution to reduce the parameter space to be explored; (3) we used a different infection history prior (prior version 3 in *serosolver*) which led to much better convergence and identifiability of the offset terms at the expense of less interpretable attack rate estimates. This alternative prior version places a Beta-Binomial prior on each individual’s expected number of lifetime infections and, unlike the prior used in the main text, assumes that an individual’s probability of infection in a given time period is unrelated to any other individual:

$$P\left( \mathbf{Z} \right)=\prod_{i=1}^{n} P\left( \mathbf{Z}_{\mathbf{i}} \right)=\prod_{i=1}^{n} \int_{0}^{1} P\left( \mathbf{Z}_{\mathbf{i}} | \boldsymbol{\Lambda}_{\mathbf{i}}=\lambda\right)P\left( \boldsymbol{\Lambda}_{\mathbf{i}}=\lambda\right)d\lambda=\prod_{i=1}^{n} \frac{B\left( \alpha+k_{i},\beta+m_{i}+k_{i} \right)}{B\left( \alpha,\beta\right)}$$

Where *k_i_* is the total number of infections experienced by individual *i*, and *m_i_* is the number of time periods individual *i* could be infected. This prior is suitable for fitting antibody landscapes to each individual’s antibody profile, but generates less interpretable attack rate estimates [5]. Model fitting was exactly the same as described in the main text, but 6 MCMC chains were run for only 12,000,000 iterations with the first 2,000,000 discarded as burn-in. Each strain-specific offset term was estimated under a multi-level model, assuming that each offset term in **χ** was drawn from the same normal distribution with mean 0 and unknown standard deviation.

[S24](#kix.63lbvakyxkzw) Fig shows that the model-predicted titres provided a better fit to the data with the added offset terms. We used the maximum posterior probability estimates for each χ as a fixed parameter for the model used in the main text ([S3](#kix.j8nfsdbxlgfy) Table). Note that the model without the offset terms produced very similar antibody kinetics parameter estimates ([S4](#kix.7o74a89c0bh3) Table), as well as age- and location-specific attack rate patterns, to those shown in the main text.

**References**

1. Hensley SE, Das SR, Bailey AL, Schmidt LM, Hickman HD, Jayaraman A, et al. Hemagglutinin Receptor Binding Avidity Drives Influenza A Virus Antigenic Drift. Science. 2009;326: 734–736.

2. Li Y, Bostick DL, Sullivan CB, Myers JL, Griesemer SB, Stgeorge K, et al. Single hemagglutinin mutations that alter both antigenicity and receptor binding avidity influence influenza virus antigenic clustering. J Virol. 2013;87: 9904–9910.

3. Fonville JM, Wilks SH, James SL, Fox A, Ventresca M, Aban M, et al. Antibody landscapes after influenza virus infection or vaccination. Science. 2014;346: 7–9.

4. Bedford T, Suchard MA, Lemey P, Dudas G, Gregory V, Hay AJ, et al. Integrating influenza antigenic dynamics with molecular evolution. Elife. 2014;2014: e01914.

5. Hay JA, Minter A, Ainslie KEC, Lessler J, Yang B, Cummings DAT, et al. An open source tool to infer epidemiological and immunological dynamics from serological data: Serosolver. PLoS Comput Biol. 2020;16: e1007840.
