# Supplementary material for: Reconstructed influenza A/H3N2 infection histories reveal variation in incidence and antibody dynamics over the life course
Source: PLoS Biol. 2024 Nov 7;22(11):e3002864. doi: 10.1371/journal.pbio.3002864 (PMC11542844; doi:10.1371/journal.pbio.3002864)
Supplement: S2 Text — (DOCX) [file pbio.3002864.s033.docx]

**Reconstructed influenza A/H3N2 infection histories reveal variation in incidence and antibody dynamics over the life course**

**S2** **Text: Scenario analyses using simulation-recovery experiments**

**2.1 Overview**

The full *serosolver* model framework is complex, with many components and parameters to be estimated or fixed. A concern is therefore the identifiability of the estimated model parameters and the potential biases introduced by inappropriate assumptions for the fixed components. To assess the ability of the *serosolver* framework to accurately recover infection histories and antibody kinetics parameters, we simulated a dataset matching the dimensions of the Fluscape survey and performed extensive simulation-recovery experiments testing the robustness of the framework to each step of our inference pipeline. The aim of this analysis was to test how simplifying assumptions and model misspecification might bias our estimates relative to the known ground truth.

**2.2 Simulation settings**

We generated a simulated serosurvey of 1000 individuals of random ages uniformly sampled between 5 and 75 years. We simulated attack rates in 3-month time windows over a 46-year time period loosely based on estimates from the real data – per-quarter attack rates were randomly drawn from a log-normal distribution with mean 0.0375 and standard deviation 1.5 (on the natural scale). We assumed an attack rate of 0.6 in the first time period to represent the pandemic wave of H3N2 in 1968. Random infection histories for each individual were then simulated for each 3-month time period as Bernoulli trials with infection probability given by the simulated attack rates. Based on these infection histories, we then simulated each individual’s latent antibody kinetics using assumed model parameter values ([S5](#kix.iqw46j2amhxb) Table). To reflect the Fluscape serosurvey, we assumed that each individual had two serum samples taken at random times from the last 24 time periods of the simulation, with two titre measurements at each sample against 24 strains uniformly distributed across the simulation period. A crucial addition to the simulation is the inclusion of strain-specific measurement offsets – measurements against each strain were assumed to be shifted relative to the true titre, where these measurement shifts were normally distributed with mean 0 and standard deviation 0.5. We generated random measurement shifts for each measured strain drawn from this distribution. Finally, we used the same antigenic map as described in the main text Materials and Methods.

**2.3 Scenario analyses**

**2.3.1 Re-estimating the strain-specific measurement offsets**

The first step of the inference pipeline is to estimate the strain-specific measurement offset terms, where some strains have systematically higher or lower titres measured in the HI assay after accounting for differences in time-since-infection, exposure history and random measurement error. Following the same approach as outlined in [S1](#hgc4a5xm1cu) Text, we fitted the *serosolver* model to the simulated data to re-estimate the strain-specific measurement offsets. The recovered parameter estimates were close to those used to simulate the data, though there were some systematic biases ([S25](#ab5zgroxybe4) Fig). Most notably the total number of infections was overestimated, the antibody boosting parameters were underestimated and the cross-reactivity parameters were overestimated. The estimated measurement offset parameters were also biased but all in the right direction ([S26](#uq2eqq9g93e2) Fig). Relative to the prior ranges (uniform between -3 and 3), the parameter estimates were close to their true values, giving us confidence that our estimates for the measurement offset terms were still informative and could thus be used as fixed values for subsequent model fits.

**2.3.2 Correctly specified model**

Using the re-estimated strain-specific measurement offsets estimated from Section 2.3.1, we fit the *serosolver* model to the simulated data under the assumption that the model was correctly specified. This provides a sense check that the inference framework is able to accurately recover the antibody kinetics parameter estimates, attack rates and infection histories when the true generative model is known. 5 MCMC chains were run for 1,000,000 iterations with a 200,000 iteration burn in period. The model accurately re-estimated the true simulation parameter values ([S5](#kix.iqw46j2amhxb) Table), though with a slight underestimation of the long-term boosting parameter ([S27](#kix.o8jmxwhd4lj8) Fig).

**2.3.3 Fitting the model ignoring strain-specific measurement offsets**

To demonstrate the importance of considering the strain-specific measurement offsets, we compare the model estimates from Section 2.3.2 to results from fitting the model without accounting for the strain-specific measurement offsets, matching the workflow of the main text model. Both versions of the model were able to accurately re-estimate individual-level infection histories and fit to the antibody data well ([S27A-B](#kix.o8jmxwhd4lj8) and [S28A-B](#38holgtjhiy5) Figs). However, the version without the measurement offset term led to biased attack rate estimates in some time periods ([S28C](#38holgtjhiy5) Fig, e.g., 1969, 1975-1980, 1995-2000, 2005-2010). Furthermore, the version without the measurement offset terms led to greater bias in the estimated antibody kinetics parameters, particularly the observation error parameter, ε ([S28D](#38holgtjhiy5) Fig). Overall, these results demonstrate that attempting to account for strain-specific measurement biases, even if incompletely, leads to parameter estimates which are closer to the true values than if these biases are ignored.

**2.3.6 Misspecifying the antigenic map**

We do not know the strains which each individual was potentially exposed to, only the most likely antigenic cluster circulating in each time period. Thus, an individual’s infection history inferred using *serosolver* depends on the assumed strain an individual could be infected with in each time period, and how it contributes cross-reactive antibodies to their antibody profile. In theory, we might jointly estimate the antigenic map coordinates alongside the other model parameters, but at present this is computationally infeasible and thus we assume a fixed antigenic map for model fitting. As our estimates rely on the assumed position of each strain on the antigenic map, we performed sensitivity analyses to test how our estimates are affected by misspecifying the antigenic map.

First, we refit the model as described in Section 2.3.1 to the same simulated dataset, but instead of using the antigenic map used to simulate the data, we used an alternative antigenic map produced by Bedford et al for model fitting [1] (comparison in [S29](#ns7hysoxi2o4) Fig). As with the antigenic map used in the main text, to generate a single antigenic position representing each strain we fitted a cubic smoothing spline through the antigenic coordinates of all strains on the map (here, smoothing parameter = 0.8). This scenario tests the assumption that the antigenic map used for fitting does not match the antigenic map underlying the data generating process. Parameter estimates, infection histories and attack rates were all largely accurate despite using the wrong antigenic map, though the timing of some elevated attack rates were slightly off (e.g., 1973, 1992, 2007). There were some small biases in the long-term boosting, cross-reactivity and observation error parameters ([S30](#kix.j9ip83ic4qf7)D Fig). This suggests that although our quantitative estimates are affected by the assumed antigenic map, the overall trends are largely preserved.

Second, we generated an alternative simulated dataset using an antigenic map where antigenic evolution was assumed to follow punctuated jumps between clusters with all strains belonging to the same cluster having the same antigenic coordinates matching the first strain isolated from each cluster ([S29C](#ns7hysoxi2o4) Fig), and then refit the model described in Section 2.3.1 using the smoothed antigenic map rather than the clustered map used for the simulation. This scenario tests the impact of our assumption that sequential strains follow a smooth and continuous path through antigenic space despite the true data generating process following punctuated jumps between clusters. We used the estimates from Du et al. to determine the time periods of cluster dominance – Du et al. predicted which A/H3N2 antigenic cluster likely circulated in China in each time period based on HA sequence data [2]. For example, where Du et al. present a time range e.g., BE92 (1992-1995), we assumed that cluster circulated up to and not including the final time point in the range e.g., assume that BE92 circulated from January 1992 up to and including December 1994. We do not have cluster information after the PE09 cluster, and thus we assumed a new cluster emerged in 2013 (i.e., the PE09 cluster dominated for 4 years). Du et al. demonstrate that clusters are almost entirely dominant in the period in which they are circulating, though they rarely reach 100% frequency.

Parameter, infection history and attack rate estimates were mostly close to their true values despite misspecifying the antigenic map, with some differences in the estimated attack rates. Infection histories and model fits matched their true values well ([S31A and S31B](#nez0vwt9n37r) Fig), though the timing of elevated attack rates did not always align ([S31C](#nez0vwt9n37r) Fig). For example, the model missed the high attack rate in 1971, likely attributing these infections to the consecutive high attack rate periods from 1968. The high attack rates in 1992 and 2007 were also slightly misaligned, though the model was able to detect high incidence around those time periods. This misalignment is expected given how the antigenic map was misspecified. In the clustered map, we assumed that all strains in a cluster had the antigenic coordinates matching the first strain. However, in the smoothed map, each strain was instead assumed to be antigenically different from the previous one. Thus, when fitting with the smooth map, the model tends to attribute infections in a cluster to the first strain, as its antigenic coordinates are the closest to their true values used in the simulation. This tends to shift attack rates in those cluster periods earlier. Reassuringly, the model was still able to accurately recover the total number of infections in the simulation.

Some of the antibody kinetics parameters were slightly biased, with notable overestimation of the observation error parameter and waning rate, and slight underestimation of the long-term cross-reactivity parameter ([S31D](#nez0vwt9n37r) Fig). Bias in the waning rate parameter estimates is unsurprising given that attack rates were estimated to be slightly later than the truth for recent time periods which provide the model with information on these short-term kinetics. If infections are estimated later than their true timing, then a higher waning rate is required to reach the same low titres in a shorter time since infection. Bias in the observation error parameter is also unsurprising, as bias from model misspecification in the measurement offset term estimation stage (Section 2.3.1) becomes compounded when fitting the full model.

**References**

1. Bedford T, Suchard MA, Lemey P, Dudas G, Gregory V, Hay AJ, et al. Integrating influenza antigenic dynamics with molecular evolution. Elife. 2014;2014: e01914.

2. Du X, Dong L, Lan Y, Peng Y, Wu A, Zhang Y, et al. Mapping of H3N2 influenza antigenic evolution in China reveals a strategy for vaccine strain recommendation. Nat Commun. 2012;3: 709.
