# Supplementary material for: Reconstructed influenza A/H3N2 infection histories reveal variation in incidence and antibody dynamics over the life course
Source: PLoS Biol. 2024 Nov 7;22(11):e3002864. doi: 10.1371/journal.pbio.3002864 (PMC11542844; doi:10.1371/journal.pbio.3002864)
Supplement: Attachment — Submitted filename: reviewer_response.pdf [file pbio.3002864.s040.pdf]

Reconstructed influenza A/H3N2 infection histories reveal variation in incidence and antibody dynamics over the life course – Response to reviewer comments

Dear Dr. Roberts and Dr Vázquez Hernández,

We thank the reviewers and editor for their supportive comments and helpful suggestions for our manuscript. A detailed response to the points raised is attached below. Our responses are in blue text, with text changes to the manuscript highlighted.

Editor comments

1. Please supply the numerical values either in the a supplementary file or as a permanent DOI'd deposition for the following figures and tables:

   We have provided the raw data for all of the figures and tables as requested, other than Fig S13 and Fig S14 which are model schematics with mock parameter values and do not use any data. Most of these figures use posterior samples from fitting our main model, and thus use R functions transforming these draws (e.g., all summary statistics are reported as posterior credible intervals, means and medians using 1000 random posterior draws). All of the code to reproduce these figures from the original posterior draws is provided in the Git repo (Zenodo for final version), and the posterior draws themselves are also included. The original antibody titre data are included.

2. NOTE: the numerical data provided should include all replicates AND the way in which the plotted mean and errors were derived (it should not present only the mean/average values).

   We have provided the raw data, and in the case of model estimates, individual posterior draws rather than posterior medians/credible intervals.

3. Please cite the location of the data clearly in all relevant main and supplementary Figure legends, e.g. "The data underlying this Figure can be found in S1 Data" or "The data underlying this Figure can be found in https://doi.org/10.5281/zenodo.XXXXX"

   Links added as requested to all figures: https://doi.org/10.5281/zenodo.12795911

4. Please ensure that your Data Statement in the submission system accurately describes where your data can be found and is in final format, as it will be published as written there.

   The data statement now references the Zenodo repository instead of GitHub.

5. We thank you for providing the code used on Github. However, please note that we cannot accept sole deposition of code in GitHub, as this could be changed after publication. However, you can archive this version of your publicly available GitHub code to Zenodo. Once you do this, it will generate a DOI number, which you will need to provide in the Data Accessibility Statement (you are welcome to also provide the GitHub access information). See the process for doing this here: https://docs.github.com/en/repositories/archiving-a-github-repository/referencing-and-citing-content

The final version of the Git repository has been uploaded to Zenodo here: https://doi.org/10.5281/zenodo.12795911.

6. Please note that per journal policy, the model system/species (human) studied should be clearly stated in the abstract of your manuscript.

   We have updated the abstract:

   a. Although experimental and quantitative analyses have improved our understanding of the immunological processes defining an individual's antibody repertoire, how these within-host processes are linked to population-level influenza epidemiology **in humans** remains unclear.
   b. These estimated infection histories allowed us to reconstruct historical seasonal influenza patterns **in humans** and to investigate how influenza incidence varies over time, space and age in this population.

7. *Resubmission Checklist* When you are ready to resubmit your revised manuscript, please refer to this resubmission checklist: https://plos.io/Biology_Checklist

8. The authors should pay particular attention to R2's concerns about young children. The authors seem to have taken no account of maternal antibodies, and they should be more cautious about this conclusion, and discuss maternal antibodies as a limitation.

   We have added a sentence in the discussion regarding maternal antibodies. No one in the cohort was young enough to have any detectable maternal antibodies at the time of sampling, which typically wane within the first 9 months of life. A child will have antibody measurements reflective of their mother's antibody landscape during their early life, but after the first year any antibodies will have been acquired through the infant's own exposure history. The youngest individual in our dataset at the time of serum sampling is 6 years old, and thus we would have no signal from maternal antibodies in our dataset. The reason we are still able to estimate infections for individuals back to their time of birth, despite not having samples taken at that time, is due to the assumption that we know which strains circulated during which time periods – if an individual has high titers to the strain circulating in their first year of life, this provides information on their likely infection history with that strain. Although we estimate infection histories to time of birth, any maternal antibodies will have waned by the time serum samples were taken, and thus we can safely ignore maternal antibody kinetics.

   We have added some text to the limitations section on the antibody kinetics model:

   Finally, we did not model the kinetics of maternal antibodies, which typically wane within the first year of life (Li et al. 2023). The youngest individual in our dataset was 6 years old at the time of sampling, and thus all maternal antibodies would have waned by the time of observation and therefore did not contribute towards the observed antibody titres.

   However, we did not interpret the reviewer's comment to be about maternal antibodies, but rather about the presence of antibodies effective against strains which circulated before an individual was born (cross-reactivity to pre-birth strains) obtained through their own immune experience. We think the reviewer is concerned that because individuals have antibodies against these pre-birth strains, this might pull the timing of estimated infections earlier in our model. This is related to the reviewer's

request to clarify how cross-reactivity is accounted for in our model which we address below.

9. I also urge the authors to be more careful about language that is suggestive of accepting the null hypothesis (whether or not this acceptance is associated with a test): "not predicted by"; "no more variation"; "did not decline". We don't know if the estimated decline is real (nor would we be sure it didn't happen even if it were actually not observed at all in this particular study): better to say something like “with no clear association with distance”

   Thank you for highlighting this. We have softened our language throughout the paper as requested. We used “predicted by” in the statistical modelling sense, that knowledge of proximity between locations could explain some variation in attack rates.

Other changes

- We have made a small number of numerical changes to our annual attack rate estimates as we developed an improved approach. We now perform a data imputation step to augment infection states for individuals after their final serum sample when calculating per-year attack rates for more recent time periods – previously we needed to exclude individuals from the denominator if their last serum sample was partway through the year (e.g., if an individual’s final sample was in Q3-2015, then we cannot estimate their infection state in Q4-2015 based on their antibody titers and thus they cannot contribute towards the overall attack rate estimate). The updated approach is described in *Methods – Post-processing of infection history posteriors:*

We also carried out an additional data augmentation step when calculating annual attack rates for more recent time periods, as we are unable to infer infection states after the latest serum sample for each individual. For example, if an individual’s final serum sample is in Q3-2014 and they have been estimated to have no infections in Q1 or Q2, it is not known if the individual should contribute to the numerator and/or denominator when calculating the annual attack rate for 2014. However, because the model assumes that the per-time infection probability is Beta-distributed, it is straightforward to sample new infection states from the model prior to seeing any antibody data using the probability of infection given by (described in (Hay et al. 2020)):

$$P(Z_{i,j}=1|\boldsymbol{Z}_{-i,j},\alpha,\beta)=\frac{k_j+\alpha}{n_j+\alpha+\beta}$$

Where $\boldsymbol{Z}_{-i,j}$ gives the infection states of all individuals other than *i* at time *j*, α and β are parameters of the Beta prior (assumed to both be 1), k_j is the number of infections in $\boldsymbol{Z}_{-i,j}$ and *n* is the number of alive individuals at time point *j.* For every individual with an unknown infection state following their final serum sample, we resample a new infection state using the above formula for each posterior draw.

- We corrected a mistake in Figure S18.

Reviewer 1

In this study, the authors further developed their own software package to model antibody kinetics against antigenically different H3N2 viruses, and applied it to a large dataset including >60,000 serum hemagglutination inhibition (HI) titers from >1,000 participants around Guangzhou, China. They also compared the new dataset with their previous smaller dataset from Viet Nam. Interestingly, the authors successfully focused on the 'infection' history inference, by utilizing mostly 'vaccine-naïve' populations for the study cohort. By reconstructing life-time infection history at individual and at population levels from the serological data against a panel of historical H3N2 virus strains, the authors found that estimated attack rates could be much higher than values based on surveillance, presumably due to asymptomatic infection. Their model fits also estimated the HI titers at different timepoints for individuals, which enabled the authors to infer the time of the previous infection events and relationship between titers and infection probability. The correlations between HI titers and protection among different age groups suggests that non-HI immunity contributed differently in different age groups. The data interpretation by the authors is generally careful and thorough, with plenty of references cited. Although there are several limitations because these types of models are based on only seroconversion and simplified factors, the authors appropriately recognized and discussed such points.

We thank the reviewer for their positive comments and are glad to see that our main findings were well received.

Specific points:

- The details of the experimental conditions for the hemagglutinin inhibition assays should be described in the method section, not by just citing reference #41, since such information is important for readers to see if the data curation steps are scientifically appropriate.

We have added further information on the HI assay protocol to the methods:

Haemagglutination inhibition (HI) assays were performed for each sample to measure antibody titres against 20 A/H3N2 strains that circulated between 1968 and 2014 inclusive with approximately 2 year spacing between circulation years (A/Hong Kong/1968, A/England/1972, A/Victoria/1975, A/Texas/1977, A/Bangkok/1979, A/Philippines/1982, A/Mississippi/1985,A/Sichuan/1987, A/Beijing/1989, A/Beijing/1992, A/Wuhan/1995,A/Victoria/1998, A/Fujian/2000, A/Fujian/2002, A/California/2004, A/Brisbane/2007, A/Perth/2009, A/Victoria/2009, A/Texas/2012, A/Hong Kong/2014). The 50% tissue culture infectious dose (TCID50) for each virus was determined using Madin-Darby Canine Kidney (MDCK) cells, calculated using the Reed-Muench method. Blood samples were kept at 4°C on the day of collection until extracted sera was frozen at -80°C until testing. Sera were thawed and treated with receptor-destroying enzyme (RDE) derived from *Vibrio cholerae* to remove nonspecific inhibitors prior to incubation at 56ºC for 30 minutes, and then absorbed with Turkey red blood cells to remove substances which might lead to nonspecific agglutination. HI assays were conducted in 96-well microtiter plates with 0.5% turkey erythrocytes using four hemagglutination units. Sera from each individual's first and second sample were tested side-by-side on the same plate. Repeat titres were generated for all of the strains tested from the second serum sample on a separate plate (23,686 repeat measurements in total), but were not run for the first sample due to insufficient sample volume. Titres were tested in serial 2-fold dilutions from 1:10 to 1:1280, with the reciprocal of the highest dilution at which hemagglutination was inhibited recorded as the titer, and undetectable titres recorded as <1:10. The full laboratory protocol is described in (Manu 2002).

For all analyses, titres were transformed to a log2 scale (i.e., 2-fold dilutions), where $y=\log_2(D/5)$, giving log titres between 0 and 8 (undetectable titres were treated as a 0 log titre). Seroconversion between study visits was defined as a 4-fold rise in titre, equivalent to a ≥2 unit increase on the log scale. Seropositivity was defined as having a titre of ≥1:40 (log titre ≥ 3). Further details on laboratory testing have been described previously [41].

- Figure 1: A and B, and C could be presented with different color-coding to improve reader understanding, since they are different parameters. Also, the labels on the color scale in Figure 1C could be shown as percentages, to avoid confusion.

Thank you for this suggestion. We have changed the colors for Fig 1C and changed the label to “percentages”. We note that the editor had requested all of the raw data underlying each figure, so the sample sizes and number seroconverted for each bin are also now included in an accompanying Zenodo repository.

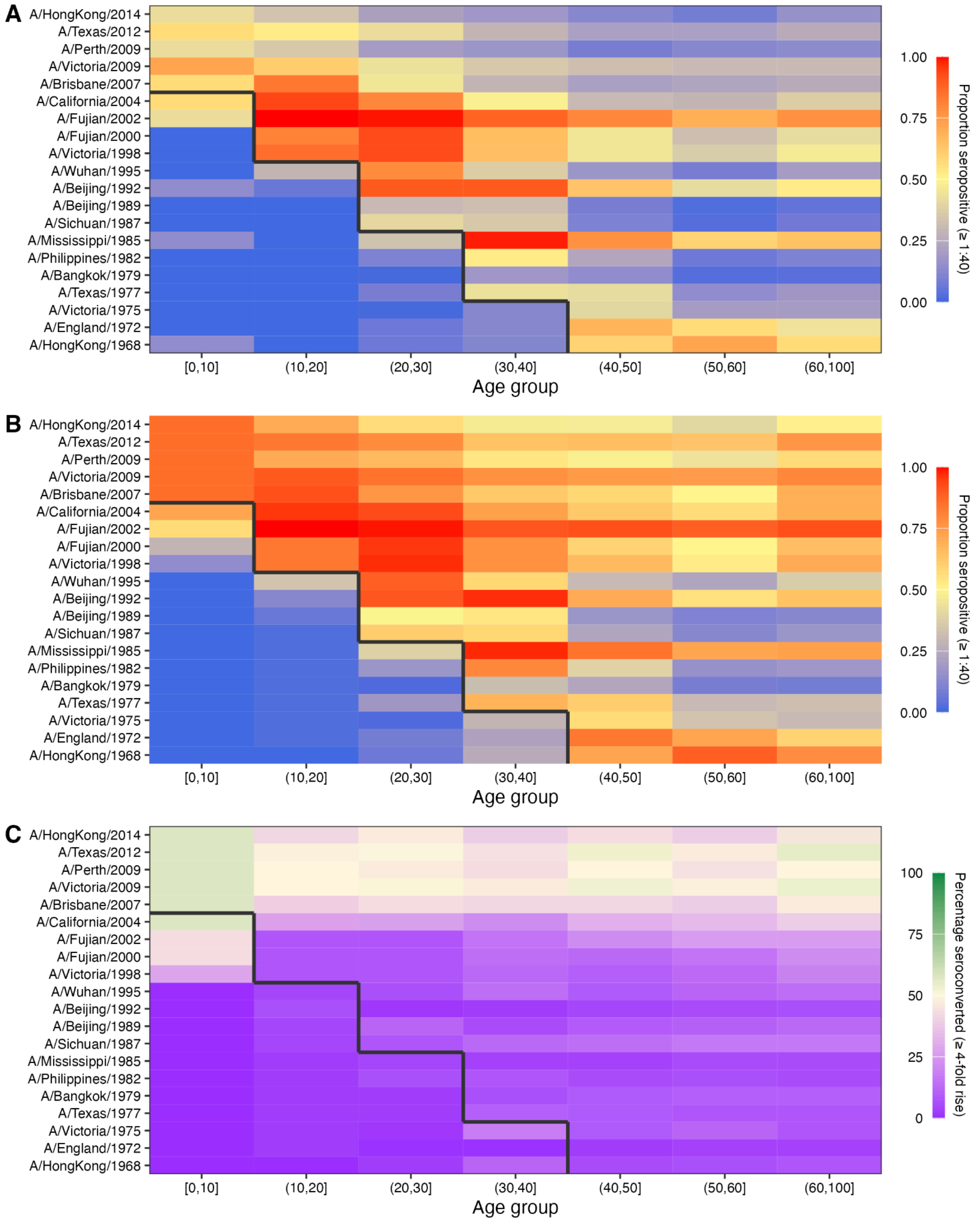
A
B
C
A/HongKong/2014
A/Texas/2012
A/Perth/2009
A/Victoria/2009
A/Brisbane/2007
A/California/2004
A/Fujian/2002
A/Fujian/2000
A/Victoria/1998
A/Wuhan/1995
A/Beijing/1992
A/Beijing/1989
A/Sichuan/1987
A/Mississippi/1985
A/Philippines/1982
A/Bangkok/1979
A/Texas/1977
A/Victoria/1975
A/England/1972
A/HongKong/1968
[0,10]
(10,20]
(20,30]
(30,40]
(40,50]
(50,60]
(60,100]
Age group
Proportion seropositive (≥ 1:40)
1.00
0.75
0.50
0.25
0.00
Percentage seroconverted (≥ 4-fold rise)
100
75
50
25
0

- Figure S6: Comparison of annual attack rates between the two cohorts. Some descriptions in the figure legend are highly subjective (e.g., remarkably similar, very different, etc.). This reviewer is wondering whehter the data could be statistically analyzed.

Thank you for highlighting this. To provide a more systematic analysis, we have updated Figure S6 to compare the proportion of posterior draws where the attack rate estimates for the Fluscape cohort was greater than that of the Ha Nam cohort. We now highlight years where more than 95% of posterior draws found a higher or lower attack rate in the Fluscape cohort (i.e., more than 95% probability). We also highlight years where between 25% and 75% of posterior draws find a higher or lower attack rate, indicating limited evidence for a difference in attack rate.

We have updated the figure caption as:

Figure S6: Comparison of annual attack rates using data from Ha Nam, Viet Nam and the Fluscape study in Guangzhou, China. Annual attack rates were defined here as the proportion of individuals who experienced at least one infection per year. Green shaded regions show time periods where >95% or <5% of posterior samples suggested a greater attack rate in the Fluscape cohort than Ha Nam, whereas yellow shaded regions show time periods where between 25% and 75% of posterior draws suggested a greater attack rate in the Fluscape cohort. Some time periods showed high uncertainty for the Ha Nam dataset, as few individuals in the sample were alive during that time (e.g., 1969-1980). Attack rate estimates were estimated to be higher in the Fluscape cohort from 2000 to 2003 inclusive with more than 95% posterior probability This might reflect a genuine different in A/H3N2 epidemiology during that time, but may also be partially driven by systematic biases in titre measurements to strains isolated during that time period – the fits to the Fluscape data include a positive offset term for titres against A/Fujian/2002, which leads to lower attack rate estimates in that time period, whereas fits to the Ha Nam data do not. The time period from 2010-2012 also shows higher attack rates in the Fluscape cohort but with similar relative patterns.

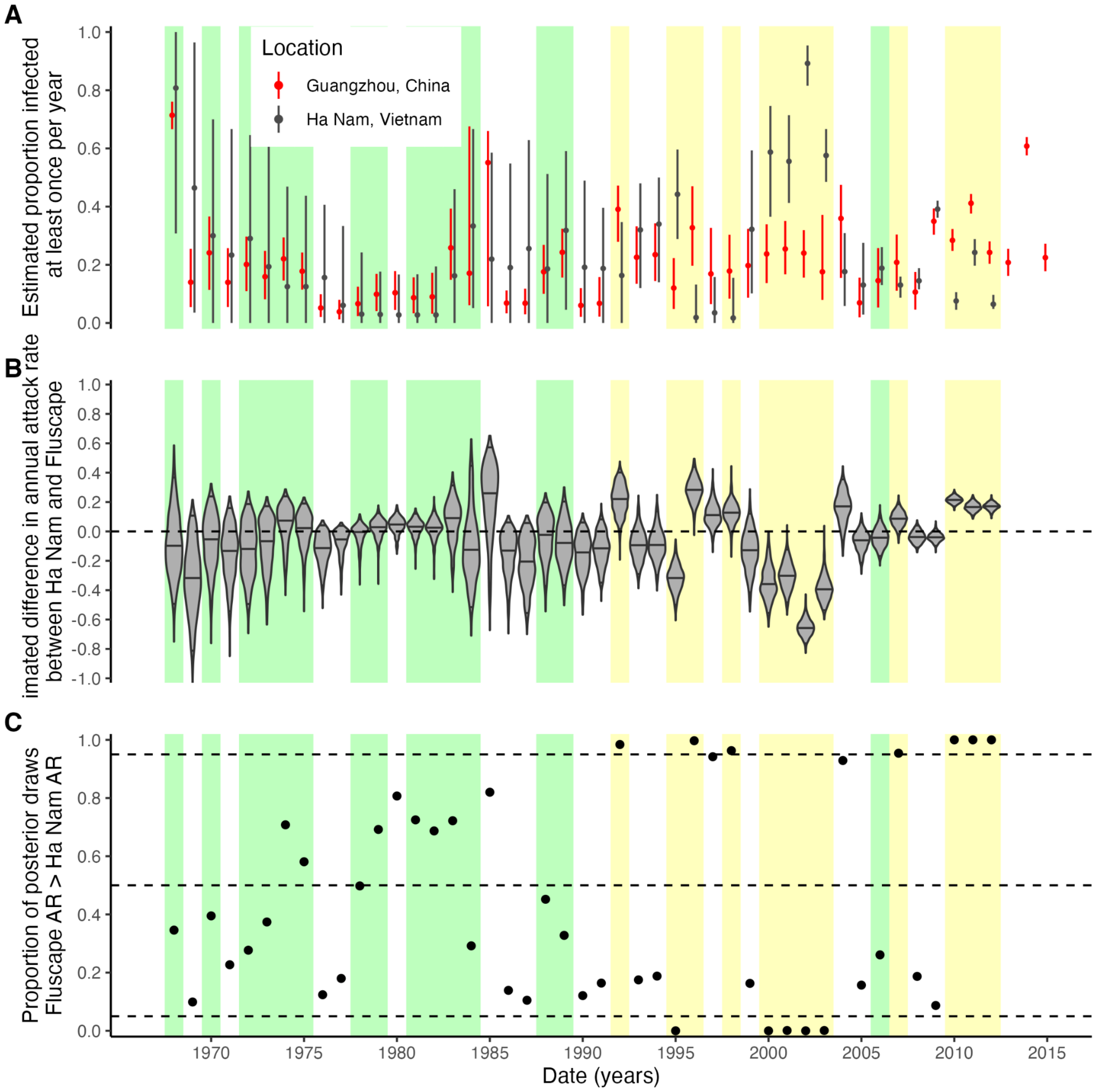

- Figure S15: "PE06" in the figure labels should be "PE09".

Corrected, thank you for catching this.

Reviewer #2:

In this manuscript by Hay et al., the authors attempt to reconstruct H3N2 infection histories using HI data from a large cohort in China. The authors analyze the relative attack rates of H3N2 and find an age-dependent risk, with more infections occurring in childhood and flattens to ~2/decade. There is an impressive amount of analyses performed to help form conclusions. Major concerns arise from basic information about study design, including samples and analysis of location, as well as concerns about the predicted infections without controlling for cross-reactive antibodies.

We have added further information on the sample collection and processing in the methods. The study design is described in full detail in a cohort profile publication, which we have made clearer in the “Cohort description – Fluscape” section. We have also added further context for our analyses based on location. We have clarified how our model accounts for cross-reactive antibodies.

Major Concerns

1. Figure 2A - Is influenza seasonal in this cohort? Relative to the Ha Nam cohort, can the differences in influenza seasonality, or lack thereof, explain similarities or differences in attack rates? Is the attack rate predicted to be higher in tropical/subtropical regions relative to regions with seasonal outbreaks?

Influenza incidence is seasonal in this cohort, though Guangzhou has a subtropical climate bordering on tropical. The two locations are fairly close together in latitude. This is a complex question, as although the literature supports a role for climate variables (which vary between subtropical and tropical locations) in determining the seasonality and timing of influenza incidence, it does not extend to measures of absolute incidence. Absolute incidence is very difficult to compare between settings due to differences in testing and reporting protocols, and thus the literature on this topic tends to focus on relative trends e.g., timing of peak incidence. A modelling study of influenza dynamics in Brazil compared to the USA and France did suggest that the effective reproductive number may be lower in tropical than temperate regions, but this does not necessarily extend to South East Asia (Chowell et al. 2010). This modelling study also found that the basic reproductive number was negatively correlated with latitude. We have added the following text to the Discussion:

The estimated annual attack rates were similar between the two cohorts, but not for all years. A possible driver of these differences is climate, despite these two locations being relatively close: Ha Nam, Viet Nam has a tropical climate with no clear influenza seasons [55], whereas Guangdong, China has a subtropical climate with clear peaks of influenza typically during the summer months, though with peaks in winter/spring in recent years [56–58]. It is possible that differences in climate and behavior partly explain differences in seasonal dynamics between the two locations [59–61], though past studies have been unable to reliably compare overall disease burden between locations due to differences in influenza case detection and reporting without further modeling [62]. Differences in influenza epidemiology between Ha Na, Viet Nam and Guangdong, China could be due to a combination of multiple factors including contact rates, climate effects on virus survival and behavior, immunological history, and dominance of different A/H3N2 strains over time.

2. The rationale for looking at location is unclear. The authors segment much their research based on location, which is really just the distance from the city center. Further clarification for these analyses is appreciated.

We agree that this rationale was not clear in our paper. This study follows our previous work where we found that including a random effect for location was significant in explaining variation in A/H3N2 antibody titers, after accounting for age and strain. This raises the hypothesis that exposure histories and incidence might significantly vary between locations at this small spatial scale. However, these previous analyses were based on antibody titres and not infections. Our model estimates allowed us to compare infection dynamics directly between locations.

We have added the following to the relevant results section:

"Previous analyses from this cohort found that antibody titres varied significantly between study locations after accounting for differences in demographics, suggesting that there may be differences in influenza A/H3N2 epidemiological dynamics between locations (Lessler et al. 2011). We sought to test the hypothesis that influenza A/H3N2 attack rates varied significantly between locations at this spatial scale using our augmented infection histories."

We should clarify that distance refers to euclidean distance between locations, not distance from the city centre. The hypothesis we are testing is if locations close to one another are more likely to exhibit similar attack rates than locations further apart. We did not find evidence to reject the null hypothesis that attack rates for locations close together are no more similar than locations further apart. We have revised the relevant results text:

"Fitting a spatial non-parametric correlation function–a model describing correlation of different spatial units over time as a function of their distance to each other– revealed high correlation in attack rates between locations which showed no clear association with increasing distance from one another (Fig S9D)."

3. The comment on page 15 about infection appears to happen shortly after birth and contradicts reference 43 - I do not think this conclusion can be made. It is clear that children can mount antibody responses to viruses prior to their birth, suggesting some level of cross-reactivity (figure 1). This could artificially move their predicted first infection up, when in reality it could be more in line with the finding in reference 43, which is longitudinal and looking for seroconversion in cohorts of relevant ages.

This is an important point. The model is able to explain these detectable pre-birth-strain titers as resulting from cross-reactivity following infection with strains circulating post birth – please see the response below for more detail. However, we do take the point that our model could be biased in how it accounts for cross-reactivity e.g., if the estimated cross-reactivity parameter is not strong enough to explain these high pre-birth strain titers, then the model might infer infections as close to the circulation time of those strains as possible, noting that the model cannot impute infections prior to birth.

Another relevant limitation is our assumption for the antigenic map which gives antigenic distances between strains in our model. We assumed a smooth path through antigenic space where the strain circulating in each timepoint was slightly antigenically different from the previous strain. We have found that this can lead to the timing of inferred infections "shrinking" towards certain time periods, whereas using an antigenic map with more punctuated antigenic changes over time leads to greater uncertainty in the precise timing of infections. Using a punctuated antigenic map may result in different estimates for the timing

of first infection. However, as discussed in *Supplementary Text 2 (2.3.6: Misspecifying the antigenic map)*, although we were able to fit this version of the model using simulated data we were unable to get reliable model convergence using the real data, and thus we cannot report these alternative estimates.

Nonetheless, we do agree that our estimates of these early life infections are likely less reliable than longitudinal cohort data as referenced, but we wished to report these estimates from our model for transparency. We have softened this statement on page 15:

We note that although these augmented infection histories suggest most individuals were infected soon after birth, our model requires a number of assumptions regarding cross-reactivity to pre-birth strains and antigenic distance between strains which limits our ability to draw this conclusion, particularly given contrasting previous findings from longitudinal serological studies in children [43].

We have added some text to the discussion paragraph on the strain coverage/antigenic map limitations on this point:

These assumptions about antigenic evolution, in addition to our simple way of modeling cross-reactive antibody boosting, might explain why our estimates for the age at first infection were much younger than found in serological data from longitudinal cohort studies of children, which are more reliable for estimating the precise timing of these early childhood infections [43]. They may also partially explain why the inferred seroresponse rates did not strictly align with timings of high incidence based on viral isolate data in Guangdong and Hong Kong for the same time period [42,46].

4. Unclear how the effects of crossreactive antibodies on attack rate estimates is being controlled.

The antibody kinetics model accounts for cross-reactivity following each infection event. If an individual is estimated to be infected with a strain, then the model computes a large boost to their antibody titer measured against that strain. At the same time, that infection is assumed to cause a boost to their antibody titer measurements against all other strains, where the degree of cross-reactive boosting is a linear function of the antigenic distance between the infecting strain and the measured strain (i.e., cross-reactive boosting is lower than homologous boosting). This is described in Figure S13.

The reviewer's question is specifically about how this cross-reactivity impacts the estimated attack rates. Because this is a Bayesian model, we are sampling from a distribution of possible infection histories which are consistent with the observed data. Thus, the model explores different possible infection histories – for example, a high titer measurement to strain A might be explained by a single infection with that strain (leading to a large boost), but it might also be explained by multiple infections with other, antigenically related strains which each contribute cross reactive antibodies (each leading to a small boost to the strain A titre). The estimated infection histories therefore account for this uncertainty, as with sparse antibody data we may not be able to discern these two infection histories. Hence, we do not present any results based on point estimates (i.e., a single estimate for the most plausible infection history). However, our antibody data are informative enough to estimate that some combinations of infections are more likely than others, which will be reflected in our estimates both for individual infection histories and population attack rates.

The attack rate estimates incorporate the uncertainty in individual infection histories by taking multiple samples from the posterior distribution, thus they reflect the possibility that

individual antibody measurements may be the result of homologous and/or cross-reactive antibodies.

We have added the following text to the *Methods – Summary of model* to clarify that uncertainty in infection histories due to cross-reactivity is accounted for when estimating population attack rates:

Note that the estimated attack rates and infection histories incorporate uncertainty in whether elevated titres against a particular strain result from infection with that strain and/or from cross-reactive antibodies from infection with a different, antigenically related strain. For example, a high titre measurement might be explained by a single infection with that strain (leading to a large boost) or by multiple infections with other, antigenically distant strains (leading to multiple small boosts). The antibody kinetics model accounts for both possibilities by including a homologous antibody boosting parameter and a model for cross-reactive antibody boosting as a function of antigenic distance (see below). This structure also allows us to estimate infection states during years from which we do not have a representative influenza strain in the HI panel. For example, in the time period 2010-2014 (Table 2), the model samples possible infection histories for that time period where elevated titres against A/Perth/2009, A/Victoria/2009, A/Texas/2012 and A/Hong Kong/2014 could reflect strain-specific antibody boosting from infections in 2010, 2012 and 2014, or cross-reactive antibodies from infection in 2011 and 2013. Hence, we do not present a single estimate for the most plausible infection history, but rather incorporate this uncertainty into the attack rate estimates by using multiple samples from the posterior for each individual's infection history.

Minor Concerns

1. Figure 4, it would be helpful to put the year of birth for each donor.

Updated as suggested.

2. Figure 4, the lab "probability that infection occurred" is misleading. This makes it sound certain. I would suggest "predicted probability that infection occurred."

Updated as suggested. We have also changed the y-axis label to HI titre to match Figure 5.

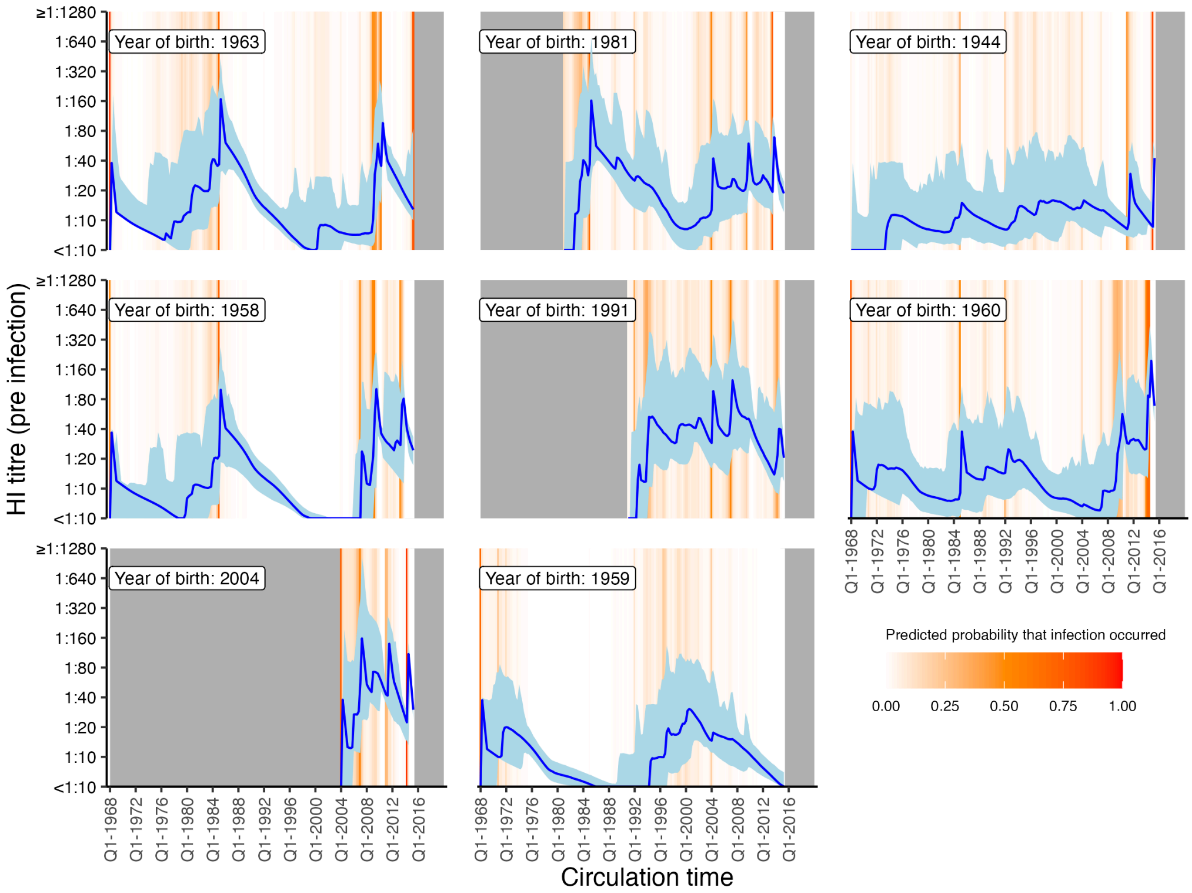

HI titre (pre infection)
≥1:1280
1:640
1:320
1:160
1:80
1:40
1:20
1:10
<1:10
Year of birth: 1963
Year of birth: 1981
Year of birth: 1944
Year of birth: 1958
Year of birth: 1991
Year of birth: 1960
Year of birth: 2004
Year of birth: 1959
Q1-1968
Q1-1972
Q1-1976
Q1-1980
Q1-1984
Q1-1988
Q1-1992
Q1-1996
Q1-2000
Q1-2004
Q1-2008
Q1-2012
Q1-2016
Circulation time
Predicted probability that infection occurred
0.00
0.25
0.50
0.75
1.00

3. A comment on the high CI shaded areas around HI titers of 7-8 in Figure 5 would be appreciated. I assume there were limited datapoints.

Yes, these are due to very small datapoints underpinning this part of the figure. We have added this to the figure legend:

Wide uncertainty intervals at higher titres reflect limited data as few individuals reach such high titres; the posterior median for number of individuals with HI titre of 1:640 or greater at the time of infection was less than 10 for all but the youngest age group.

4. Figure 5, log base what?

This is the log transform of the HI dilution factor used throughout the paper – log2(dilution/5). To make this result easier to compare to previous work, we instead label the x-axis with the original titre rather than log titre.

5. Figure 5, it would also be appreciated to add the relative risk at 40 HI titer for each graph.

Dashed line corresponding to the relative risk at a 40 HI titer added as requested.

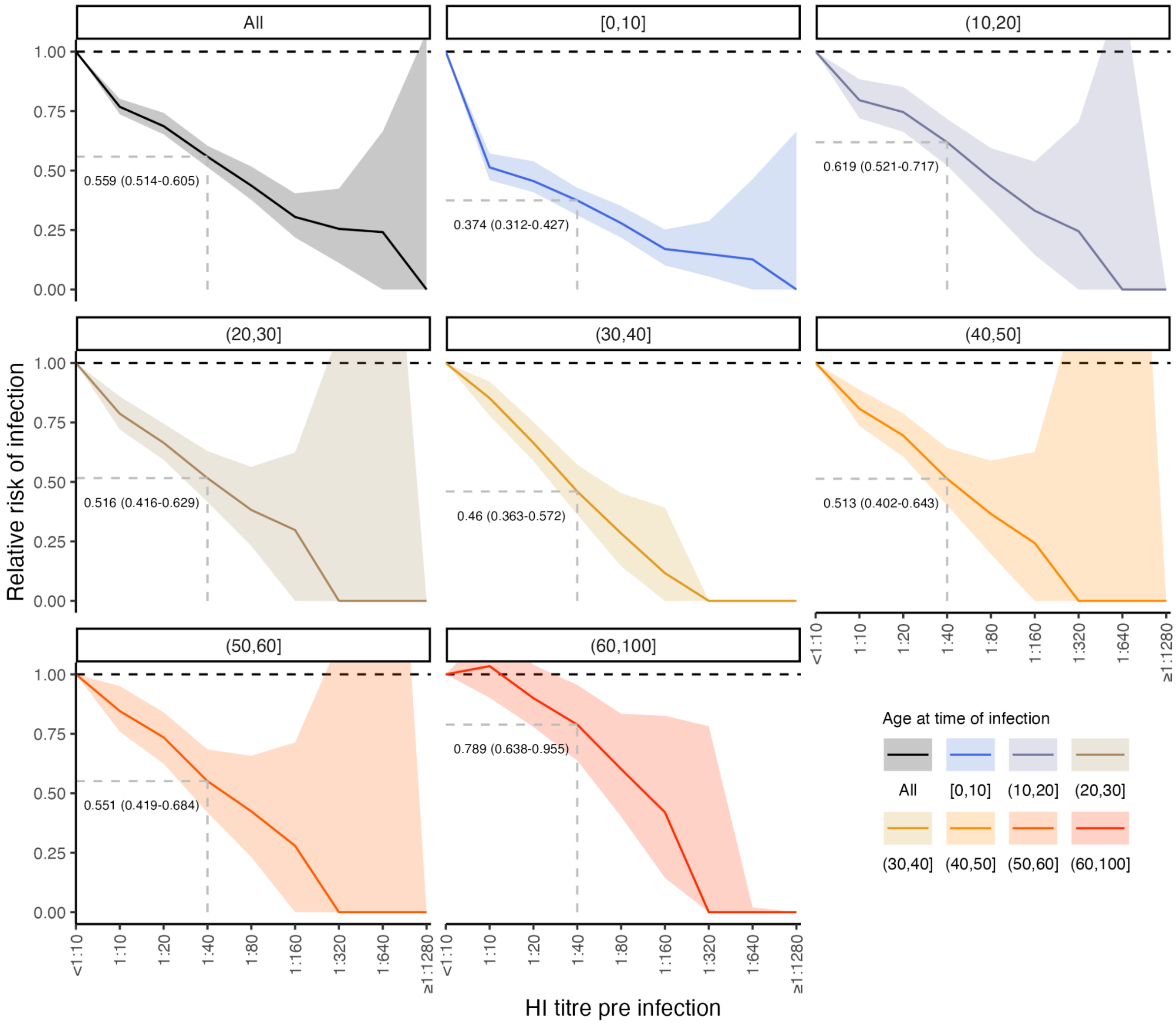

Reviewer #3:

Hay and colleagues leverage serum samples collected as a part of the Fluscape cohort study, perform hemagglutination inhibition (HI) assays across 20 antigenically unique viruses (1968-2014) and integrate these data into models that infer past infection events, define past attack rates at the population level across an age stratified cohort, and predict the likelihood of infection based on HI titer. Methodologies developed, adapted, and validated herein are predicted to be useful for understanding past population-level infection histories, current epidemiological trends and past or present susceptibility to infection. As the authors note, these approaches capture data that may otherwise be lost due to surveillance efforts that have largely relied upon symptomatic illness.

The manuscript is a tome, but is well written in language that is broadly understandable. I offer these suggestions to further solidify its conclusions or impact. I thank the authors for their descriptions and explanations of their models in clear and understandable language. The careful statements of chosen parameters, their meaning, and limitations and caveats were welcome.

Thank you for your positive comments.

Major:

The authors find a high frequency of infections within this cohort over the study window. Reinfections were also greater post 2008. This 2008-present time period corresponds to a point at which human H3N2 viruses developed a complex circulation history that involved multiple antigenically distinct clades. Given the assays and models are sensitive to antigenicity, could these observations i) be due to increased infections due to increased circulating antigenic diversity and ii) in part inflated due to the lack of representation of multiple clades (e.g. 3C.3a and its descendants) that circulated during the study window. While this may not be able to be addressed experimentally it could be discussed or modeled.

These are insightfulpoints that we have now addressed in the discussion. Your first suggestion is that our model might be capturing a genuine increase in infections due to the increased antigenic diversity during this time, which is certainly possible. The second suggestion is important, as our model does not account for co-circulation of antigenically varied strains (we must choose a single strain to represent a time period), and as you point out, our HI panel did not reflect antigenic diversity during this time period. If the strains which circulated were antigenically distant to the one we assumed in the model, then this could bias our infection estimates. However, this bias would likely have the opposite effect. If an individual was actually infected with a strain (strain A) which was antigenically distant to the one we assumed they were infected with (strain B), then their actual boost of antibodies to strain A would be lower than to strain B. Strain B antibodies would be lower, and thus because the model takes elevated titers as evidence for infection, lower titers means the model is less likely to infer infections.

It is not possible to model this with our current framework, but we have discussed these possibilities and highlight the limitation of our model in the revised Discussion:

This increased incidence during recent time periods could reflect the changing antigenic diversity of influenza A/H3N2 during this time, with the emergence of multiple antigenically distinct clades providing an increased force of infection from strains carrying different mutations leading to more frequent infections [52–54]. Our model is limited in capturing this changing epidemiology, as it cannot account for co-circulation of competing strains at the

same time, and our HI panel did not have strains representing these multiple clades. However, if individuals in this cohort were infected with strains that were not measured in the HI panel it is likely the model would underestimate infection incidence; antigenic mismatch between the infection strain and the measured strain would lead to lower observed titres, which will lead to a lower posterior probability of infection in the model.

Many of the results here align with previous observations. These observations lend support to this approach, its scalability and utility of the models employed. This work would be greatly enhanced if it had demonstrated predictive value. I do not know if the following can be addressed with current models and donor/sample information. It would be interesting if predictions could be made based on the first sample collection of the likelihood of infection before the second sample collection. Similarly, if known vaccinations during the study window could be identified. If these are not tractable, a bit of discussion of what data would need to be captured or models developed to infer population-level/age-group risk of infection.

For the first type of prediction, with this framework, we cannot predict the likelihood of infection using antibody data alone as this will depend on the population force of infection and the circulating strain. We can only estimate the relative risk of infection for different titre values assuming we know the circulating strain, which is demonstrated in Figure 5. We have added a sentence to the Discussion:

We note that our estimates are only of relative rather than absolute risk of infection; predicting the future probability of infection for an individual would additionally require knowing the force of infection (probability of exposure) as well as knowing which strain will circulate.

The suggestion to identify known vaccinations is interesting and is an analysis we have been working on a little since the initial submission. However, influenza vaccinations were only self-reported within large windows of time rather than based on precise vaccine records, which makes their use as true positives difficult. Nonetheless, hey provide an independent report of potential antibody boosting events to compare our inferred infection histories against. We have added new results to demonstrate that our model does accurately capture most of these self-reported vaccination events.

We refer to new Supplementary Figures S22 and S23 which show the timing of inferred infections (antibody boosts) against self-reported vaccination status, separating 62 correctly identified vaccinations from 28 missed vaccinations. For the 28 “missed” vaccinations, we note that antibody boosting to recent strains (antigenically closest to the vaccine strain) are either low or did not increase between samples, suggesting either recall error or no vaccine-induced antibody boosting.

New text in *Materials and Methods:*

Validation using self-reported vaccination

We validated our inference approach by comparing the model-predicted probability of infection to self-reported influenza vaccination. Vaccination coverage was low in this cohort (Table S1), and self-reported vaccination status is not a perfect reflection of true vaccination status. Furthermore, vaccination status was reported only within a window of time rather than on a specific date: individuals were asked if they had ever received an influenza vaccination and could give one of six answers: never vaccinated; in the same calendar year;

in the preceding calendar year; 2-5 years ago; 5+ years ago; or unsure. Individuals were also asked if they had been vaccinated since their last study visit. Where an individual reported vaccination in one of these time periods, we define the covered time period as the "vaccination window".

To assess the accuracy of our inference approach, we defined sensitivity as the proportion of vaccination windows +/- 3 months which contained at least one inferred infection with more than 25% posterior probability (i.e., we expect vaccination windows to contain inferred infections if our inference is accurate). However, a complication is that these windows are wide – an individual reporting vaccination 2-5 years ago has a 3-year wide vaccination window, and thus the individual is likely to have been infected during that time regardless of vaccination. Therefore to provide a comparable null simulation, we took each of the windows of time with reported vaccination and reassigned them to randomly chosen individuals at random times (e.g., if individual 1 reported vaccination between Q1-2013 and Q1-2015; the null simulation reassignment might move the window to individual 2 in Q2-1980 to Q2-1982, regardless of individual 2's vaccination status). We repeated this process 100 times to generate 95% uncertainty intervals for the null simulation. We then compared the proportion of vaccination windows which contained inferred infections based on reported vaccination to the null simulations. If the upper 95% uncertainty interval of the null simulation was lower than the proportion of vaccination windows with inferred infections using the real data, we took this as evidence that our model was more likely to infer antibody boosting events that coincided with self-reported vaccination than in randomly selected time periods.

New results text in *Inferring antibody kinetics and individual infection histories from antibody profiles*:

Although there were no virologically confirmed infections reported in the Fluscape study with which to validate our infection history inference, individuals did self-report influenza vaccination at each study visit, but only within a window of time rather than on a specific date (e.g., in the preceding calendar year; see Materials and Methods). We estimated antibody boosting events (infections) with >25% posterior probability for 68.9% of time windows in which individuals self-reported influenza vaccination (62 of 90 windows from 77 individuals; Figure S22&S23), compared to 43.1% (mean of null simulations; 95% quantiles: 32.8%-51.7%) of randomly selected time windows of the same duration from randomly selected individuals, suggesting that our model was more likely to infer antibody boosting events that coincided with self-reported vaccination than in randomly selected time periods. Vaccination windows in which no vaccination/infection was inferred tended to either have antibody boosts identified soon after the reported vaccination, suggesting either delayed boosting or recall error, or very low antibody titres to recent influenza strains, suggesting either inaccurate recall or no vaccine-induced antibody boosting (Figure S23).

New text in discussion:

Similarly, although our inference method did not distinguish between vaccination and infection, vaccination coverage in this cohort was very low and thus this assumption is unlikely to bias our infection history estimates substantially. In lieu of virologically confirmed influenza infections with which to validate our model, we used self-reported vaccination to approximate true antibody boosting events, finding that our model was more likely to impute antibody boosts during self-reported vaccination windows than in randomly selected time periods with no reported vaccination. Although this provides some support for the validity of our approach, self-reported vaccination status is not always an accurate measure of true vaccination status due to recall bias, and our model does not account for different antibody kinetics following infection and vaccination. Indeed, for most of the self-reported vaccinations where our model found limited evidence for an antibody boosting event showed limited

antibody responses against recent strains; both of these limitations make it difficult to evaluate the validity of our approach based on vaccination data alone.

Finally, we have added two new figures comparing the individual model-predicted probability of infection to time periods with reported influenza vaccination, alongside the observed antibody titre data for comparison:

<< Separate file >>

Figure S22: Comparison of observed antibody profiles, model-predicted infection states and self-reported vaccination status for accurately detected vaccinations. Left-hand column shows raw data as in Figure S20. Right-hand column shows the model-estimated posterior probability of infection (higher gray area suggested higher probability of infection) compared to self-reported vaccination states. Orange regions show time periods in which individuals reported having been vaccinated for influenza. Purple regions show time periods in which individuals reported no vaccination for influenza. Vertical dashed lines show the time of serum sample collection. Individuals were included in this plot (rather than Figure S23) if the posterior probability of infection during one of the orange time windows was >25%.

<< Separate file >>

Figure S23: Comparison of observed antibody profiles, model-predicted infection states and self-reported vaccination status for missed vaccinations. Left-hand column shows raw data as in Figure S20. Right-hand column shows the model-estimated posterior probability of infection (higher gray area suggested higher probability of infection) compared to self-reported vaccination states. Orange regions show time periods in which individuals reported having been vaccinated for influenza. Purple regions show time periods in which individuals reported no vaccination for influenza. Vertical dashed lines show the time of serum sample collection. Individuals were included in this plot (rather than Figure S22) if the posterior probability of infection during one of the orange time windows was <25%.

Minor:

Table 2: Can the authors describe how infections in 2011 and 2013 were inferred without using viruses from these years in their panel.

This is a result of how the model works: antibody titers are modeled as the culmination of multiple infections with different strains, accounting for homologous and cross-reactive boosting and waning. We have added an additional paragraph to *Methods – Summary of model* explaining how cross-reactivity is incorporated into the infection history/attack rate estimates to address this and another reviewer's comment:

Note that the estimated attack rates and infection histories incorporate uncertainty in whether elevated titres against a particular strain result from infection with that strain and/or from cross-reactive antibodies from infection with a different, antigenically related strain. For example, a high titre measurement might be explained by a single infection with that strain

(leading to a large boost) or by multiple infections with other, antigenically distant strains (leading to multiple small boosts). The antibody kinetics model accounts for both possibilities by including a homologous antibody boosting parameter and a model for cross-reactive antibody boosting as a function of antigenic distance (see below). This structure also allows us to estimate infection states during years from which we do not have a representative influenza strain in the HI panel. For example, in the time period 2010-2014 (Table 2), the model samples possible infection histories for that time period where elevated titres against A/Perth/2009, A/Victoria/2009, A/Texas/2012 and A/Hong Kong/2014 could reflect strain-specific antibody boosting from infections in 2010, 2012 and 2014, or cross-reactive antibodies from infection in 2011 and 2013. Hence, we do not present a single estimate for the most plausible infection history, but rather incorporate this uncertainty into the attack rate estimates by using multiple samples from the posterior for each individual's infection history.

Figure 2: Denote what the gray shading is for. I am assuming it corresponds to dates before a donor's birth.

This is correct. We have clarified this in the figure legend:

"The grey areas show time periods prior to each individual's birth."

Figure S3. What accounted for the higher HAI titers for visit 2 and perhaps broader serum antibody reactivity?

The main possibility is that increased incidence between visit 1 and visit 2 led to higher titers overall, which is reflected in our model estimates. We note that this increase in titers between study rounds has been reported previously in Yang et al. PLOS Pathogens 2020 (https://doi.org/10.1371/journal.ppat.1008635). Our model also estimated higher cross-reactive breadth in the short term following infection, and thus more recent infections would lead to increased antigenic breadth. However, it is possible that these differences reflect laboratory effects which we could not account for in our model. Although these are paired titres (i.e., visit 1 and visit 2 samples tested side-by-side at the same time), it is possible that there are unaccounted for laboratory effects (e.g., freeze-thaw reducing antibody concentrations in the earlier samples). We were not able to account for this here. We have added some text on this point to the limitations:

We performed paired tests of the serum samples from the two study visits to minimize batch effects, though it is possible that the increase in titres and broader reactivity between study rounds (Figure S2&S3) might reflect systematic measurement bias rather than increased recent infections as inferred by our model. For example, the delay between sample collection and testing for visit 1 samples might have led to some decline in antibody concentration within the samples.
